# Dual Ligand‐Enabled Nondirected C─H Olefination of (Hetero)Arenes for the Synthesis of Clickable Derivatives

**DOI:** 10.1002/chem.202600018

**Published:** 2026-02-05

**Authors:** Tommaso Braga, Maria Hergert, Manuel van Gemmeren

**Affiliations:** ^1^ Otto Diels‐Institut für Organische Chemie Christian‐Albrechts‐Universität zu Kiel Kiel Germany

**Keywords:** C─H activation, click chemistry, late‐stage functionalization, olefination, palladium catalysis

## Abstract

Herein, we present a highly efficient method for the late‐stage introduction of “clickable” alkyne moieties into different (hetero)arene scaffolds. A highly efficient dual ligand‐based palladium catalyst combining an N‐acylsulfonamide (NASA) ligand with an N‐heterocycle enables the introduction of acrylate moiety bearing a terminal alkyne via nondirected C─H activation, providing a versatile platform for subsequent bioconjugation or activity‐based protein profiling (ABPP) applications. The utility of the method is demonstrated through a broad substrate scope and the subsequent use of the obtained products in representative click reactions.

## Graphical abstract

We report an efficient method for late‐stage installation of “clickable” alkyne groups into diverse (hetero)arenes. Using a dual‐ligand palladium catalyst that combines an NASA ligand with an N‐heterocycle, an acrylate unit bearing a terminal alkyne can be introduced via nondirected C─H activation. This approach enables broad substrate scope and delivers products suitable for subsequent CuAAC reactions.

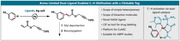

## 1 Introduction

**SCHEME 1:**
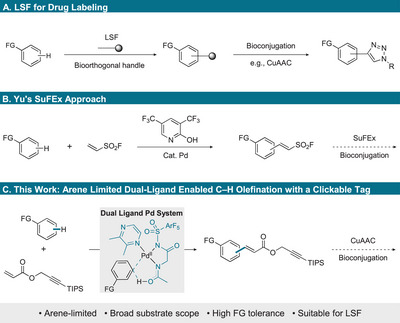
Bioorthogonal labeling strategies via nondirected C─H activation.

The chemical “tagging” of drugs or drug‐like molecules with azides or alkynes is widely used as a handle for bioorthognal labeling [1, 2] or activity‐based protein profiling (ABPP) [3, 4], a chemoproteomic technology for investigating metabolic processes. Activity‐based probes (ABPs) are designed with reactive warheads that covalently interact with the active site of a protein and can be tracked by suitable reporters such as fluorophores or biotin; however, it has been shown that bioorthogonal functional groups (FG) such as azides and alkynes are preferred for in cellulo applications [3, 5, 6, 7, 8]. In this context, copper‐catalyzed azide‐alkyne cycloaddition (CuAAC) [9, 10, 11], associated as click chemistry became a vital tool for drug labeling [12]. These tags are usually attached to the target compound by suitable prefunctionalization (e.g., alcohol or amine), which may necessitate a step‐intensive de novo synthesis. A more elegant route would be to introduce a tag through late‐stage C─H functionalization (LSF) [13, 14, 15, 16] that enables the use of the already available bioactive compound (Scheme 1A) [17]. Two general C─H activation approaches for LSF are distinguished: directed and nondirected C─H activation. Whereas directed pathways rely on the presence of specific FG to position the tag in proximity to the functional unit, nondirected methods offer complementary regioselectivity and broader applicability, as they do not require a directing group [18, 19]. Due to the often‐limited amounts of the bioactive compound available, a second required criterion for LSF includes arene‐limited reaction conditions [13]. Only a few studies have addressed the introduction of traceable tags into organic molecules through arene‐limited nondirected C─H activation. The group of Yu developed a C─H fluorosulfonyl vinylation of simple arenes and demonstrated the utility of their tag in follow‐up SuFEx chemistry (Scheme 1B) [20].

Analogously, the group of Wo and Yao proposed an “*alkyne‐containing acrylate*” in combination with nondirected arene C─H olefination as a highly promising strategy [12, 21, 22]. Subsequently, the same authors demonstrated the feasibility of this approach in principle, albeit with a narrow scope of very electron‐rich and thus activated arenes [23]. This led us to hypothesize that a more active catalyst system tailored specifically toward the *clickable* olefin reaction partner can enhance overall reactivity and thereby enable broader applicability (e.g., electron poor arenes), particularly for biologically active substances. In this regard, the aim of our research was to complement existing methods rather than simply improving conditions for already accessible substrates. These considerations motivated us to target the development of a widely applicable protocol. We envisioned to attain clickable drug conjugates by applying a catalyst system based on the cooperative action of two complementary ligands, an N‐heterocycle and an N‐acyl amino acid derivative with an alkyne‐containing acrylate as a reaction partner. The late‐stage C─H olefinated compounds would then be suitable for subsequent CuAAC transformations (Scheme 1C).

## 2 Results and Discussion

Our studies began with subjecting o‐xylene (**1a**) as a model substrate and acrylate **2** under the reaction conditions previously developed by our group for the nondirected olefination of arenes (Table 1, Entry 1) [22]. Product **3a** was obtained in a yield of only 29%. This observation raised the question why this particular acrylate performed poorly under the reaction conditions previously developed by our lab [22]. We therefore began monitoring the stability of acrylate **2**, which turned out to be a key parameter to identify optimal reaction conditions [24]. Since lowering the reaction temperature led to reduced decomposition of acrylate **2** (Δ2, Entries 2, 3), we adjusted the temperature at 70 °C and increased the reaction time to 48 h (Entry 4).

**TABLE 1:** Optimization of the reaction conditions.

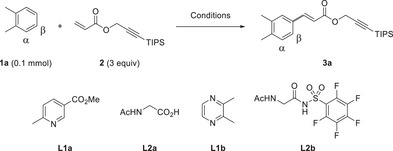					
Entry	Cond.	Solvent	Yield (%)	α:β ratio	Δ**2** (%)
1	A, 90 °C	HFIP	29	8:92	30
2	A, 80 °C	HFIP	27	7:93	30
3	A	HFIP	25	7:93	14
4	A, 48 h	HFIP	33	7:93	20
5	B	HFIP	51	3:97	13
6	B	TFE	54	3:97	13
7	B	TFE:DCE 2:8	58	2:98	12
8	B	TFE:CHCl_3_ 2:8	66	2:98	4
9	B^a^	TFE:CHCl_3_ 2:8	63	3:97	3

**Cond. A**: Pd(OAc)_2_ (10 mol%), **L1a** (20 mol%), **L2a** (30 mol%), AgOAc (2 equiv), solvent (1 mL), 70 °C, 24 h. **Cond. B**: Pd(OAc)_2_ (15 mol%), **L1b** (30 mol%), **L2b** (45 mol%), AgOAc (2 equiv), solvent (1 mL), 70 °C, 48 h.

^a^ 10 mol% Pd(OAc)_2_:**L1b**:**L2b** = 1:2:2, 0.6 mL solvent. Δ**2** = Amount of **2** not accounted for by remaining reagent or consumption toward product formation, i.e., decomposition of reagent **2**.

During an extensive ligand screening (see the Supporting Information for details) we discovered that N‐acylsulfonamide (NASA) ligands promote the reaction efficiently. In particular, the combination of 2,3‐dimethyl pyrazine (**L1b**) and the pentafluoroaryl‐NASA ligand **L2b** improved the reaction outcome to 51% yield (Entry 5, transition state model shown in Scheme 1C) [25]. The new catalyst system showed a good solvent compatibility, enabling the reaction to proceed in various solvents and solvent mixtures (Entries 6‐8 and Scheme S17 in the Supporting Information). The optimal solvent system was identified as a 2:8 mixture of trifluoroethanol (TFE) and chloroform, which afforded the product in 66% yield with minimal decomposition of acrylate **2** (Entry 8). These findings motivated us to conduct a more detailed comparison of solvents and reaction conditions and their influence on the decomposition of acrylate **2**, which allowed us to identify the major decomposition products (see Scheme S25 and discussion in the Supporting Information).

Interestingly, other ligand combinations exhibited lower activity in this reaction medium (see Scheme S19 in the Supporting Information), suggesting that NASA ligands may be particularly valuable for avoiding or reducing the need for polyfluorinated solvents such as HFIP (1,1,1,3,3,3‐hexafluoroisopropanol) [26]. Subsequent variations in stoichiometry and concentration enabled a reduction in catalyst loading and led to the formation of the product in 63% yield with an *α:β* ratio of 3:97 (Entry 9). Finally, we compared our results with the conditions developed by Yu et al. for the synthesis of β‐arylethenesulfonyl fluorides (giving products suitable for SuFEx transformations), using 3,5‐bis(trifluoromethyl)‐2(1H)‐pyridinone as ligand (see Scheme S26 in the Supporting Information) [20]. Notably, these conditions performed poorly for the alkyne containing acrylate used in this study, affording the desired product in only 18% yield. Conversely, our reaction conditions delivered suboptimal results (36% yield) when using ethenesulfonyl fluoride as a reaction partner. These findings confirm that the methods are indeed complementary. The olefin **2** used herein requires particularly mild reaction temperatures and solvents other than HFIP to prevent decomposition, while less sensitive olefins can be converted at harsher conditions to obtain increased yields.

**SCHEME 2:**
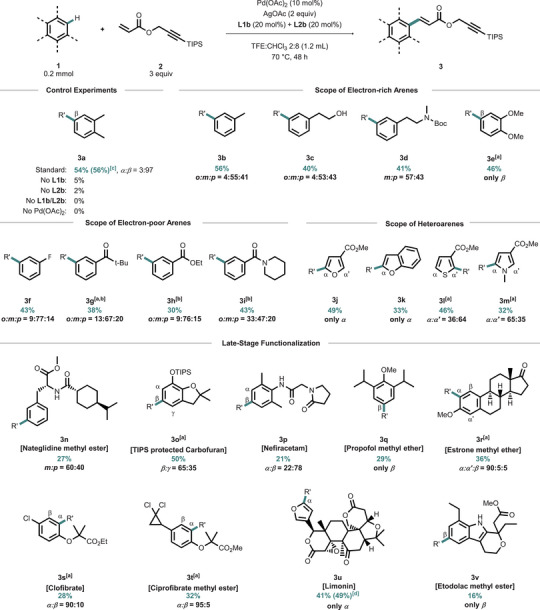
Substrate scope of simple and complex arenes. [a] Mixture of *E*:*Z* isomers [b] 15 mol% Pd(OAc)_2_, 80 °C [c] 3 mmol scale, 75 °C [d] 1 mmol scale, 75 °C.

With the final conditions in hand, we began our scope studies (Scheme 2) using a set of simple arenes, wherein electron‐rich substrates could be functionalized with good yields giving **3a**–**3e**. Monoalkylated substrates (products **3b**–**3d**) tended to preferred meta substitution, as expected based on our olefination studies with simple acrylates [22]. We were curios if free alcohols and amides would be tolerated, and we observed majorly functionalization in meta position yielding **3c** and **3d**. The products derived of disubstituted arenes, **3a** and **3e**, were also obtained in useful yields. We proceeded to study electron‐poor substrates and, with slightly increased catalyst loading, obtained **3f**–**3i** with moderate to good yields depending on the degree of the electron‐deficiency of the arene. Piperidine amide‐derived product **3i** showed a slightly higher formation of the ortho product (o:m:p = 33:47:20), presumably due to a competition between directed and nondirected functionalization [27]. We proceeded to investigate heteroarenes [28] as substrates. Two furane derived‐products (**3j**, **3k**), a thiophene methylester (**3l**) and a methyl protected pyrrole (**3m**) were obtained in useful yields, the sterically accessible position being the preferred site of functionalization. Having gained general information regarding the regioselectivity and functional group tolerance of our method, we focused our studies on the functionalization of medicinally and biologically relevant compounds. We began with the methyl ester of Nateglidine, an agent used against type 2 diabetes mellitus, yielding product **3n** in 27% with a preference for meta functionalization (60:40) [29, 30]. Carbofuran is a highly toxic insecticide for the protection of crops [31]. We used a TIPS‐protected analog, giving **3o** in 50% as a mixture of *β* (major) and *γ* (minor) functionalized product. The isomers were found to be separable by column chromatography. We were curious if aniline derived substrate Nefiracetam [32], a nootropic drug administered to people with cognitive issues would be tolerated, as we expect a potential catalyst coordination by the lewis‐basic sites in this substrate. Hence, we observed preferentially functionalization in the less hindered *β* position yielding **3p** in 21%.

**SCHEME 3:**
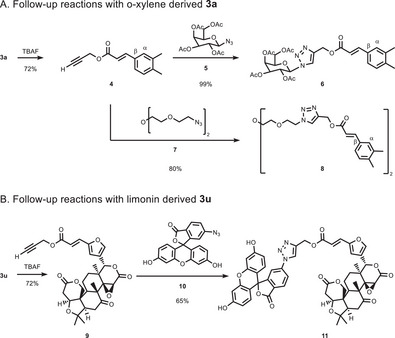
Follow‐up CuAAC reactions.

The comparably reactive substrate Propofol is one of the most commonly used anesthetics for pediatric surgery [33]. Its functionalized methyl ether **3q** was obtained in moderate yield as sole *β* regioisomer. Next, a tri‐substituted methyl ether derived from Estrone, a female estrogen was tested [34]. Its olefinated product **3r** was isolated as a mixture of regioisomers (36%), with a preferential functionalization in *α* position. We furthermore tested two antihyperlipidemia fibrates giving products **3s** and **3t** and for both substrates observed a preference for C─H activation in  ortho position to the methylpropanate, the electronically preferred position, obtaining similar yields of 28% and 32%, respectively. The 1,1‐dichloro cyclopropane moiety in product **3t** as well as the chloro substituent on the arene in **3s** stayed intact under the applied olefination conditions. The structurally complex substrate limonin, a tetracyclic triperpenoid, is found in high concentrations in the seeds of citrus fruits and has been shown to possess a variety of biological activities, including anticancer, antiinflammatory, and antiviral properties [35]. Its polycyclic structure features a carbonyl group, two lactone rings, an ether, and an epoxide. Using our method, a yield of 41% was obtained, and all FGs remained intact under the conditions employed. Product **3u** was isolated as a sole regioisomer (α). Another heteroarene ring is present in indole‐derived Etodolac. This nonsteroidal antiinflammatory drug was derivatized in the sterically most accessible *β* position yielding **3v** as sole isomer [36]. The use of our method in late‐stage functionalization underscored a high steric control in our olefination process, as well as a wide variability with respect to the electronic properties of the substrates and a tolerance for various FG. Notably, to date only few examples for nondirected late‐stage modifications of bioactive compounds toward bioorthogonal labeling have been described [23]. The moderate to good yields obtained with our method render the method highly useful, when viewed in the context of obviating the need for a multistep de novo synthesis [37].

To demonstrate the use of our products in subsequent labeling steps (Scheme 3), o‐xylene **1a** and limonin **1u** were first submitted to our method on a large scale (3 mmol and 1 mmol, respectively). For each example, the terminal alkyne was converted into a 1,4‐disubstituted 1,2,3‐triazole. O‐xylene‐derived product **3a** was deprotected in 72% yield and quantitatively transformed into the 1,4‐disubstituted product **6** as sole β‐isomer using tetra acetylated galactose azide **5**. Product **8**, derived of a twofold CuAAC reaction was obtained in 80% yield as a *β,β*‐isomer and is of special interest, since its benzene derivative (instead of having o‐xylene) showed cytotoxic, antiproliferative, and antimetastatic activity against melanoma cells [38]. We furthermore envisioned a rapid construction of a chemically tagged bioactive compound using our olefination conditions. Labeled limonin **3u** was deprotected with 72% yield and subsequently tagged with fluorescein azide **10** in a CuAAC reaction giving product **11** in 65% yield (23% over 3 steps from limonin).

## 3 Conclusion

Within this study we demonstrated a technique for the labeling of (hetero)arenes utilizing an alkyne modified acrylate which serves as a versatile handle for bioconjugation or ABPP via azide‐alkyne click reaction. In total, nine simple arenes, four heteroarenes, and nine complex arenes—including major bioactive compounds— have been demonstrated. The method exhibited good tolerance toward important FG, including esters, amides, lactones, and free alcohols. Although the catalyst nature inherently favors less hindered positions, both steric and electronic factors were found to influence the reaction, enabling novel substrate modifications and underscoring the value of LSF for drug labeling applications. As a proof of concept, we further demonstrated three examples of subsequent CuAAC reactions for the rapid construction of complex, labeled compounds. We strongly advocate that our catalytic system makes LSF an attractive tool for drug labeling, particularly for practitioners in the field of ABPP research.

## Conflicts of Interest

The authors declare no conflicts of interest.

## Supporting information




**Supporting File 1**: The authors have cited additional references within the Supporting Information [1, 2, 3, 4, 5, 6, 7, 8, 9, 10, 11, 12, 13, 14, 15, 16].
