# Supplementary material for: Dual Ligand‐Enabled Nondirected C─H Olefination of (Hetero)Arenes for the Synthesis of Clickable Derivatives
Source: Chemistry. 2026 Feb 5;32(15):e00018. doi: 10.1002/chem.202600018 (PMC13107500; doi:10.1002/chem.202600018)
Supplement: Supplementary file 1 — Supporting File 1: The authors have cited additional references within the Supporting Information [1, 2, 3, 4, 5, 6, 7, 8, 9, 10, 11, 12, 13, 14, 15, 16]. [file CHEM-32-e00018-s001.pdf]

Dual-Ligand Enabled Nondirected C–H Olefination of (Hetero)Arenes for the Synthesis of Clickable Derivatives

Tommaso Braga†, Maria Hergert† and Manuel van Gemmeren

Otto Diels-Institut für Organische Chemie, Christian-Albrechts-Universität zu Kiel, 24118 Kiel, Germany

Supporting Information

Table of Contents

General Experimental Methods 2

Optimization of the Reaction Conditions 4

Ligand synthesis 34

Starting Material Synthesis 38

Scope of the Reaction 44

Further Derivatization of Product Molecules 66

NMR Spectra 71

References 112

1. General Experimental Methods

Solvents, Reagents and Techniques

Unless otherwise noted, all reactions were carried out in oven-dried glassware (120°C). Reaction temperatures refer to the temperature of the aluminum-block surrounding the reaction vessel.
Commercially available chemicals were obtained from ABCR, Acros Organics, BLD-pharm, Alfa Aesar, Fluorochem, Sigma Aldrich or TCI Europe and used as received. The AgOAc utilized during these studies was purchased from ABCR. A strong dependency of the reaction on the quality and morphology of the AgOAc was observed, leading to varying results with AgOAc purchased from other suppliers. The AgOAc was stored in a glove box and batches for short term use were extracted when required.
1,1,1,3,3,3-Hexafluor-2-propanol (HFIP) was purchased from Fluorochem and directly used it in the reaction. Additional anhydrous solvents (<50 ppm water) were purchased from Fisher Scientific and stored over molecular sieves under inert atmosphere.

Chromatography

Analytical thin layer chromatography (TLC) was performed on silica gel ALUGRAM Xtra SIL G/UV_{254} plates (Macherey-Nagel), neutral plates (Merck). Compounds were visualized by ultraviolet light (254 nm or 366 nm) or by staining with $KMnO_4$ solution (1 g $KMnO_4$, 6 g K_2CO_3 and 0.1 g KOH in 100 mL of H_2O), bromocresol green solution (40 mg bromocresol green in 100 mL EtOH; addition of 0.1M (aq.) NaOH until the blue color appears in the solution) or phosphomolybdic acid (PMA) solution ($H_3[Mo_{12}PO_{40}]\cdot 12H_2O$ in Ethanol). Flash chromatography was performed on silica gel 60M (0.04-0.063 mm). Positive overpressure was applied. Automated flash chromatography was performed on a Biotage Isolera One system. Compounds were detected by a UV-detector.

Nuclear Magnetic Resonance (NMR) Spectroscopy

^{1}H, ^{13}C, and ^{19}F NMR spectra were recorded at room temperature on a Bruker AvanceNeo 500 or a Bruker Avance 600 device. Chemical shifts (δ) are given relative

to tetramethylsilane (TMS) and referenced to residual solvent signals as tabulated by Fulmer et al.[1] Chemical shifts are given with two decimal numbers (^{1}H) or one decimal number (^{13}C and ^{19}F). Data is reported in the following order: Chemical shift (multiplicity [s = singlet, d = doublet, t = triplet, q = quartet, quint = quintet, hept = septet, m = multiplet, br = broad signal], coupling constant (*J* [Hz]) and number of H-atoms). For the spectra of regioisomeric mixtures signals clearly assigned to a particular regioisomer are labelled with a superscript at the integration. The number of protons in such cases refers to the number of protons of the respective isomer. The absence of such an index indicates that the signals of all observed regioisomers overlap, the integration given corresponds to the number of protons in each isomer.
The ^{13}C-NMR spectra of mixtures are reported as observed. Due to the low signal intensity and potentially an overlap of signals, the number of signals can deviate from the hypothetical value, however, the signals of the major components are clearly recognizable in all cases and correspond to the literature values whenever the respective compounds are literature known. All NMR-spectra were processed using MestReNova program.

Mass Spectrometry (MS)

High resolution mass spectra (HRMS) were recorded on a Jeol AccuTOF using electron impact (EI) or a ThermoFisher Orbitrap spectrometer using electron spray ionization (ESI).

Gas Chromatography with Flame Ionization Detection (GC-FID)

GC-FID analysis was performed using an Agilent Technologies 7890B or 8860 instrument with an HP5 column (30 m, 0.32mm × 0.25 μm) and nitrogen as carrier gas.

Gas Chromatography with Mass Spectrometry (GC-MS)

GC-MS was performed on an Agilent Technologies 8890 system coupled to an Agilent Technologies 5977B mass detector (EI) and an HP-5MS column (30 m, 0.32mm × 0.25 μm). Helium was used as carrier gas.

2. Optimization of the Reaction Conditions

General procedure for the reactions carried out during the optimization of the reaction conditions

An oven dried 10 mL Schlenk tube was charged with Pd-source, monodentate ligand (**L1**), bidentate ligand (**L2**), Ag-source, *ortho*-xylene (**1a**) (10.6 mg, 0.100 mmol), 3-(triisopropylsilyl)prop-2-yn-1-yl acrylate (**2**), and the solvent. The reaction vessel was tightly sealed and placed into a preheated aluminum block with a tightly fitting recess on a magnetic stirrer set at 1000 rpm. The reaction was stirred at the set temperature and speed. After the indicated reaction time, the reaction mixture was allowed to cool to room temperature. 1,3,5-Trimethoxybenzene (16.8 mg, 0.100 mmol) was added and the reaction mixture was diluted with EtOAc (4 mL). An aliquot (300 μL) of the resulting solution was filtered over a short silica column using ethyl acetate as the eluent and the resulting sample was subjected to GC-FID analysis. The yields given during the optimization studies were determined by GC-FID analysis of the crude reaction mixture using 1,3,5-trimethoxybenzene as internal standard, unless otherwise stated.

Scheme S1: Starting conditions.

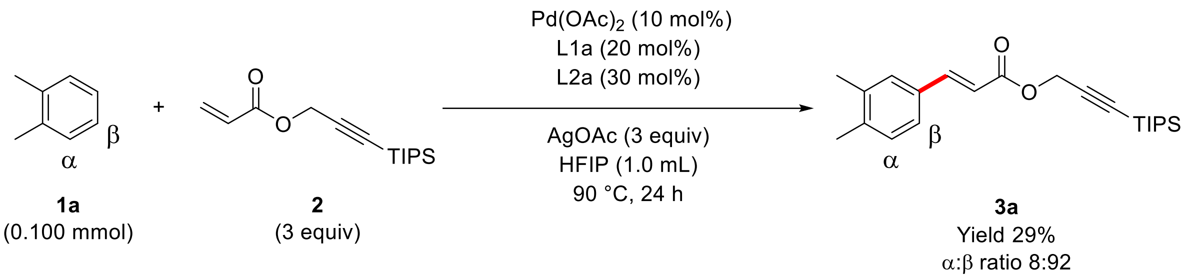

Note: the starting conditions were selected according to the previous findings of our group.[2]

Scheme S2: Preliminary time screening.

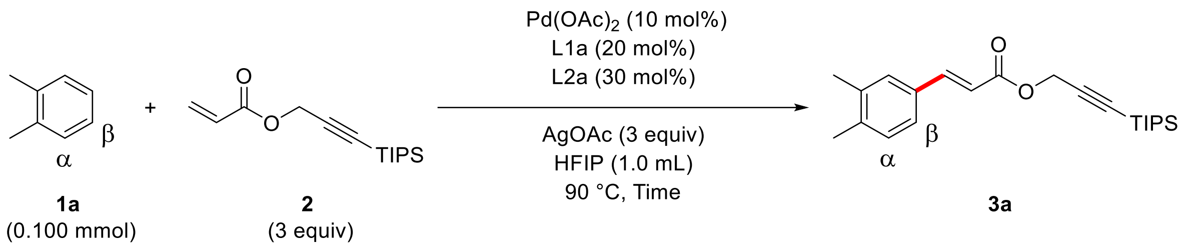

Entry	Time (h)	**1a** Conv. (%)	Yield (%)	α:β ratio
1.	6.5	38	13	10:90
2.	12	47	19	9:91
3.	18	55	29	8:92
4.	24	56	29	8:92
5.	48	60	26	8:92

Scheme S3: Preliminary temperature screening.

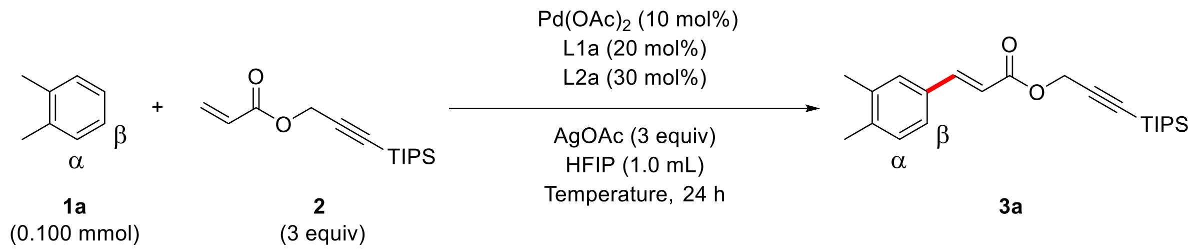

Entry	Temperature (°C)	**1a** Conv. (%)	Yield (%)	α:β ratio	**2** Decomp. (%)
1.	60	30	23	6:94	7
2.	70	41	25	7:93	14
3.	80	52	27	7:93	30
4.	90	56	29	8:92	30

Entry	Temperature (°C)	**2** theoretical amount (%)	**2** real amount (%)	**2** Decomp. (%)
1.	60	92	85	7
2.	70	92	78	14
3.	80	91	61	30
4.	90	90	60	30

Note: the stability of **2** (coupling partner) was introduced as an additional parameter to be monitored. The initial amount of **2** was precisely weighted. The remaining amount after the reaction was determined via GC-FID. The theoretical value describes the amount that would be expected to remain if **2** were consumed only for product formation, while the real amount refers to the actual remaining amount, determined by GC-FID. The difference between theoretical amount and real amount gives the percentage of decomposition (**2** Decomp.) reported in the table. In the next schemes only the **2** Decomp. (%) value is reported. More explanation regarding the decomposition of **2** is provided in **3-(Triisopropylsilyl)prop-2-yn-1-yl acrylate (2) decomposition test** chapter.

Scheme S4: Preliminary time screening at 60°C and 70°C.

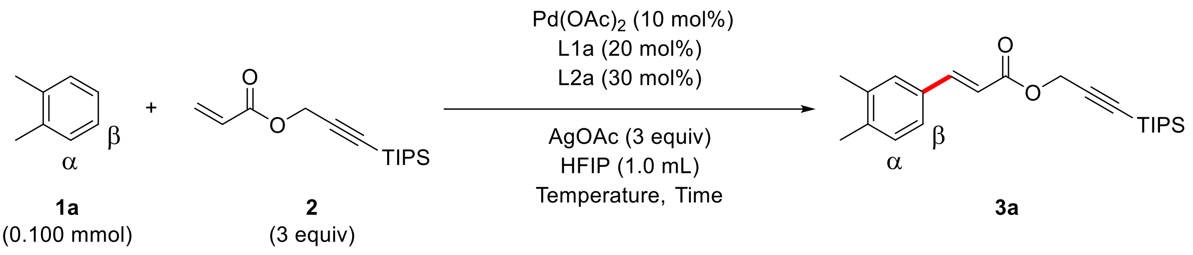

Entry	Temperature (°C)	Time (h)	Yield (%)	α:β ratio	**2** Decomp. (%)
1.	60	24	25	7:93	11
2.	60	48	27	7:93	13
3.	60	72	31	6:94	18
4.	70	24	27	7:93	14
5.	70	48	32	7:93	17
6.	70	72	32	6:94	22

Yields and ratios were determined by ^{1}H-NMR using 1,3,5-trimethoxybenzene as internal standard.

Scheme S5: Oxidant screening.

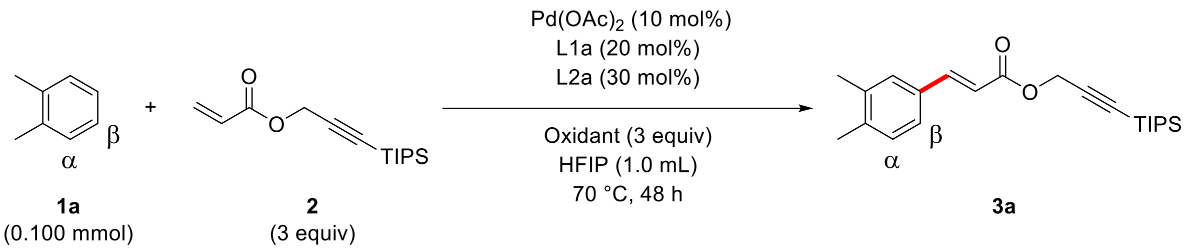

Entry	Oxidant	Yield (%)	α:β ratio	**2** Decomp. (%)
1.	AgOAc	31	6:94	17
2.	Ag_2CO_3	18	6:94	18
3.	$AgNO_3$	14	6:94	37
4.	Ag_3PO_4	7	14:86	6
5.	Ag_2O	9	11:89	85
6.	$Cu(OAc)_2$	-	-	-
7.	AgF	-	-	-
8.	AgOTf	-	-	-
9.	1,4-Benzoquinone	-	-	-

Yields and ratios were determined by ^{1}H-NMR using 1,3,5-trimethoxybenzene as internal standard.

Scheme S6: AgOAc amount screening.

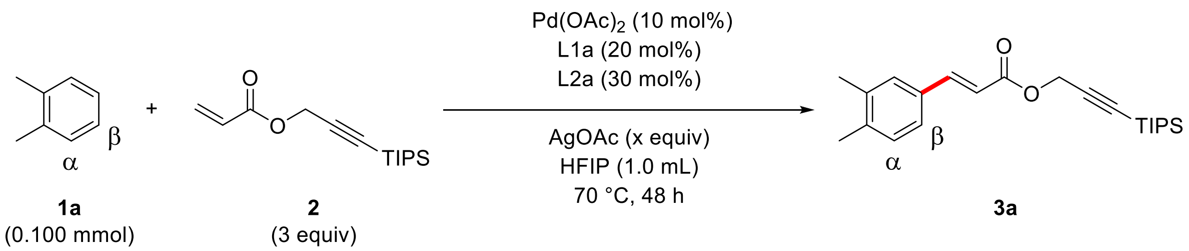

Entry	x	**1a** Conv. (%)	Yield (%)	α:β ratio
1.	1	32	21	7:93
2.	2	41	31	6:94
3.	3	51	30	5:95
4.	4	51	29	7:93

Scheme S7: 2 amount screening.

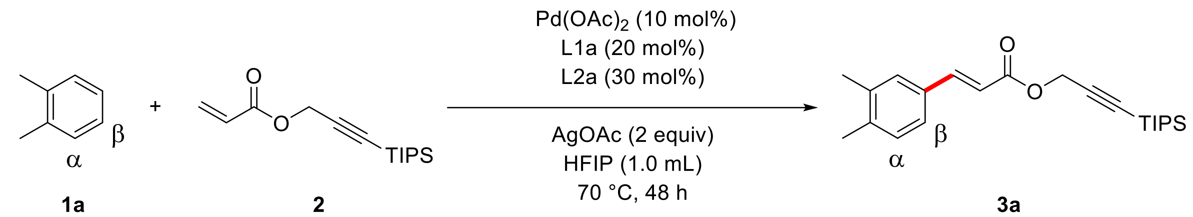

1a (0.100 mmol) + **2** (x equiv) → **3a**

Entry	x	**1a** Conv. (%)	Yield (%)	α:β ratio
1.	1	58	26	7:93
2.	2	52	29	6:94
3.	3	44	28	7:93
4.	4	36	24	7:93

Scheme S8: Bidentate ligand (**L2**) screening – Part 1.

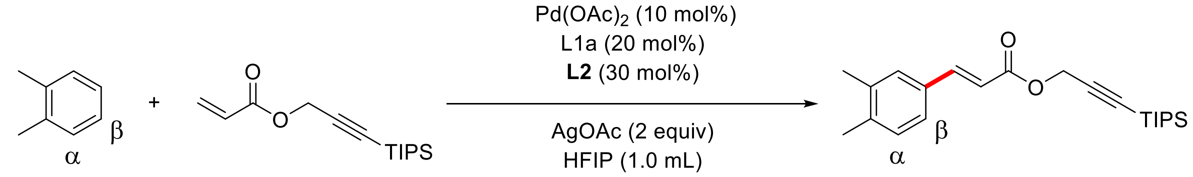

1a (0.100 mmol) **2** (3 equiv) **3a**

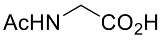

L2a

Conversion: 50%
Yield: 30%
Ratio (α:β): 7:93

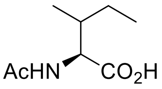

L2c

Conversion: 24%
Yield: 16%
Ratio (α:β): 11:89

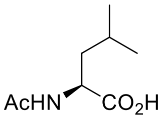

L2d

Conversion: 39%
Yield: 20%
Ratio (α:β): 9:91

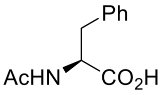

L2e

Conversion: 48%
Yield: 29%
Ratio (α:β): 4:96

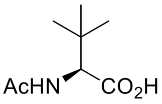

L2f

Conversion: 18%
Yield: 9%
Ratio (α:β): 18:82

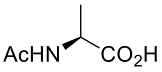

L2g

Conversion: 43%
Yield: 25%
Ratio (α:β): 7:93

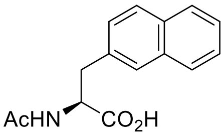

L2h

Conversion: 42%
Yield: 31%
Ratio (α:β): 4:96

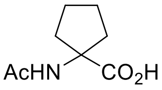

L2i

Conversion: 22%
Yield: 12%
Ratio (α:β): 17:83

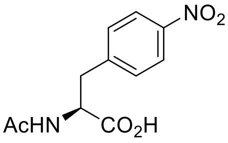

L2j

Conversion: 35%
Yield: 21%
Ratio (α:β): 8:92

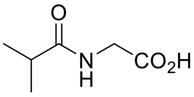

L2k

Conversion: 33%
Yield: 22%
Ratio (α:β): 9:91

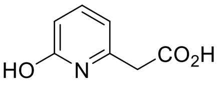

L2l

Conversion: 21%
Yield: 9%
Ratio (α:β): 7:93

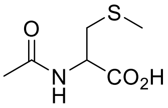

L2m

Conversion: 19%
Yield: 9%
Ratio (α:β): 15:85

Scheme S9: Bidentate ligand (**L2**) screening – Part 2.

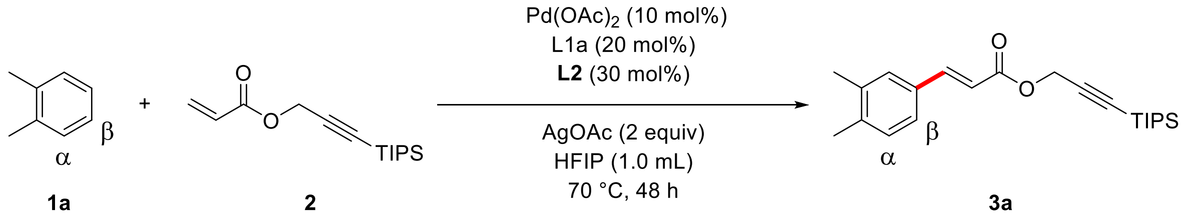

1a (0.100 mmol) | **2** (3 equiv) | **3a**

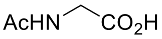

L2a

Conversion: 50%
Yield: 30%
Ratio (α:β): 7:93

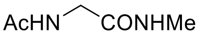

L2n

Conversion: 25%
Yield: 13%
Ratio (α:β): 17:83

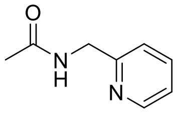

L2o

Conversion: 15%
Yield: 4%
Ratio (α:β): 0:100

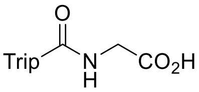

L2p

Conversion: 21%
Yield: 9%
Ratio (α:β): 7:93

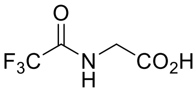

L2q

Conversion: 19%
Yield: 9%
Ratio (α:β): 15:85

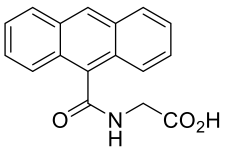

L2r

Conversion: 33%
Yield: 22%
Ratio (α:β): 9:91

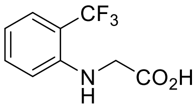

L2s

Conversion: 35%
Yield: 21%
Ratio (α:β): 8:92

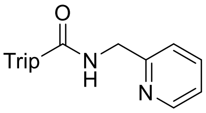

L2t

Conversion: 6%
Yield: -
Ratio (α:β): -

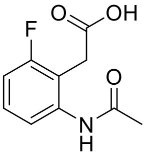

L2u

Conversion: 24%
Yield: 10%
Ratio (α:β): 18:82

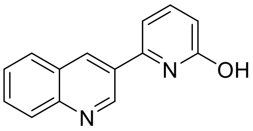

L2v

Conversion: 4%
Yield: -
Ratio (α:β): -

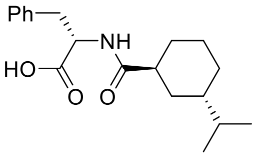

L2x

Conversion: 25%
Yield: 15%
Ratio (α:β): 8:92

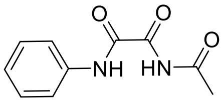

L2y

Conversion: 5%
Yield: -
Ratio (α:β): -

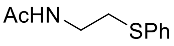

L2z

Conversion: 20%
Yield: 13%
Ratio (α:β): 16:84

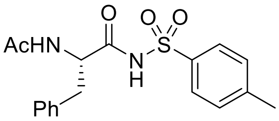

L2aa

Conversion: 12%
Yield: 5%
Ratio (α:β): 7:93

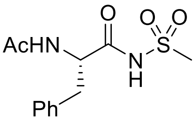

L2ab

Conversion: 13%
Yield: 7%
Ratio (α:β): 9:91

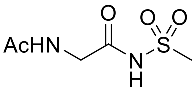

L2ac

Conversion: 36%
Yield: 24%
Ratio (α:β): 7:93

Scheme S10: Preliminary monodentate ligand (**L1**) screening.

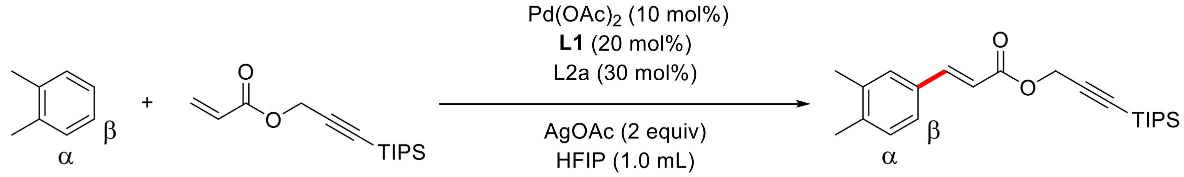

1a (0.100 mmol) | **2** (3 equiv) | **3a**

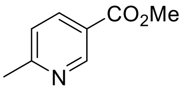

L1a

Conversion: 50%
Yield: 30%
Ratio (α:β): 7:93

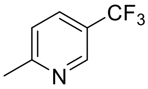

L1c

Conversion: 45%
Yield: 26%
Ratio (α:β): 5:95

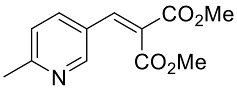

L1d

Conversion: 50%
Yield: 33%
Ratio (α:β): 6:94

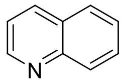

L1e

Conversion: 41%
Yield: 26%
Ratio (α:β): 7:93

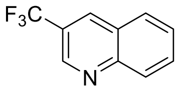

L1f

Conversion: 47%
Yield: 26%
Ratio (α:β): 7:93

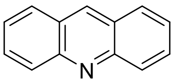

L1g

Conversion: 27%
Yield: 12%
Ratio (α:β): 4:96

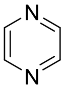

L1h

Conversion: 55%
Yield: 29%
Ratio (α:β): 14:86

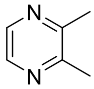

L1b

Conversion: 49%
Yield: 30%
Ratio (α:β): 6:94

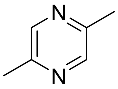

L1i

Conversion: 57%
Yield: 37%
Ratio (α:β): 7:93

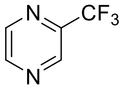

L1j

Conversion: 48%
Yield: 21%
Ratio (α:β): 11:89

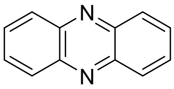

L1k

Conversion: 33%
Yield: 17%
Ratio (α:β): 4:96

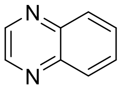

L1l

Conversion: 52%
Yield: 35%
Ratio (α:β): 7:93

Scheme S11: Preliminary catalyst loading screening.

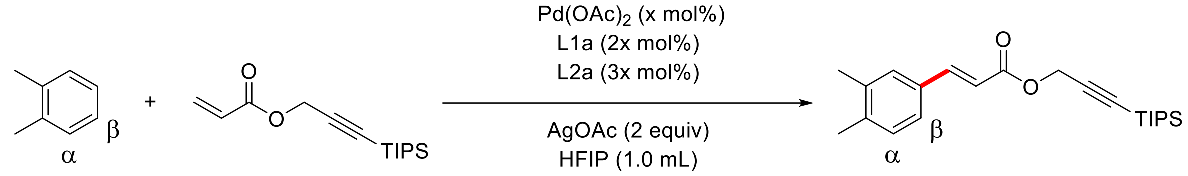

1a
(0.100 mmol)

2
(3 equiv)

3a

Entry	x	**1a** Conv. (%)	Yield (%)	α:β ratio
1.	10	50	30	6:94
2.	15	55	35	6:94
3.	20	60	35	6:94

Scheme S12: Bidentate ligand (**L2**) screening – Part 3.

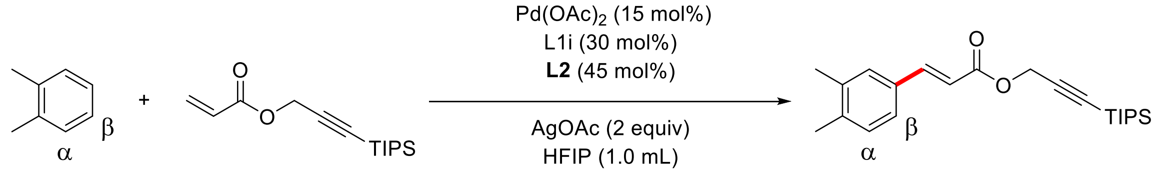

1a (0.100 mmol) | **2** (3 equiv) | **3a**

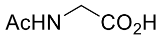

L2a

Conversion: 55%
Yield: 42%
Ratio (α:β): 5:95
2 Decomp.: 18%

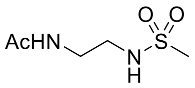

L2ac

Conversion: 20%
Yield: 14%
Ratio (α:β): 16:84
2 Decomp.: 36%

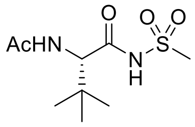

L2ad

Conversion: 24%
Yield: 13%
Ratio (α:β): 17:83
2 Decomp.: 40%

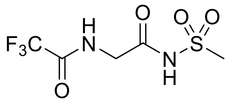

L2ae

Conversion: 32%
Yield: 21%
Ratio (α:β): 10:90
2 Decomp.: 37%

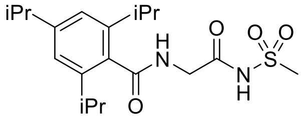

L2af

Yield: 11%
Ratio (α:β): 42:58
2 Decomp.: 23%

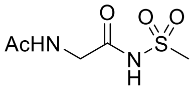

L2ag

Conversion: 57%
Yield: 46%
Ratio (α:β): 4:96
2 Decomp.: 25%

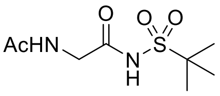

L2ah

Conversion: 27%
Yield: 24%
Ratio (α:β): 9:91
2 Decomp.: 26%

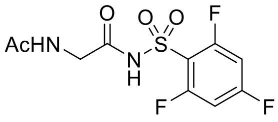

L2ai

Conversion: 60%
Yield: 47%
Ratio (α:β): 3:97
2 Decomp.: 15%

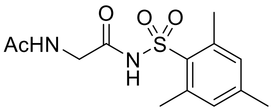

L2aj

Conversion: 41%
Yield: 37%
Ratio (α:β): 4:96
2 Decomp.: 23%

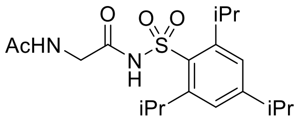

L2ak

Conversion: 16%
Yield: 14%
Ratio (α:β): 15:85
2 Decomp.: 28%

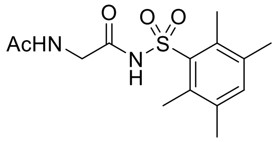

L2al

Conversion: 39%
Yield: 38%
Ratio (α:β): 4:96
2 Decomp.: 21%

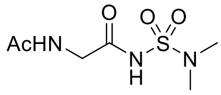

L2am

Conversion: 37%
Yield: 36%
Ratio (α:β): 5:95
2 Decomp.: 23%

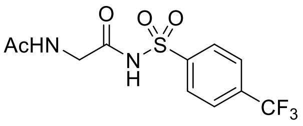

L2an

Conversion: 55%
Yield: 43%
Ratio (α:β): 5:95
2 Decomp.: 16%

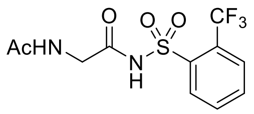

L2ao

Conversion: 46%
Yield: 39%
Ratio (α:β): 4:96
2 Decomp.: 16%

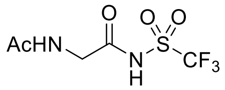

L2ap

Conversion: 56%
Yield: 47%
Ratio (α:β): 4:96
2 Decomp.: 18%

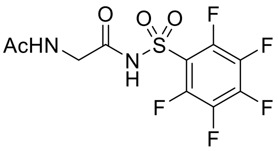

L2b

Conversion: 58%
Yield: 49%
Ratio (α:β): 3:97
2 Decomp.: 15%

Scheme S13: Monodentate ligand (**L1**) screening – Part 1.

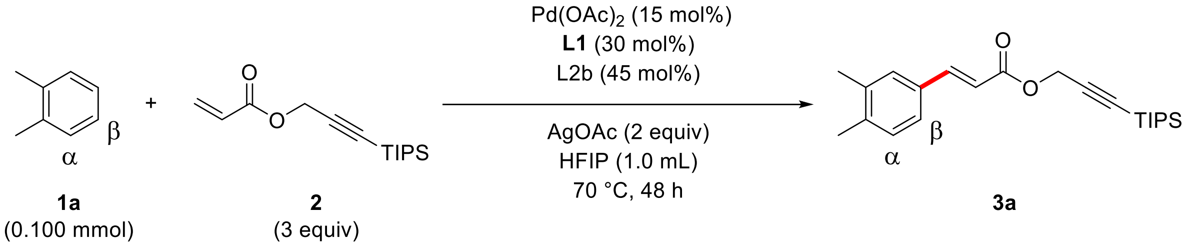

1a (0.100 mmol) | **2** (3 equiv) | **3a**

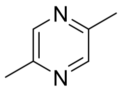

L1i

Conversion: 61%
Yield: 49%
Ratio (α:β): 3:97
2 Decomp.: 15%

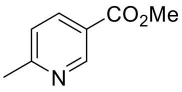

L1a

Conversion: 45%
Yield: 37%
Ratio (α:β): 5:95
2 Decomp.: 21%

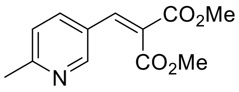

L1d

Conversion: 50%
Yield: 46%
Ratio (α:β): 4:96
2 Decomp.: 17%

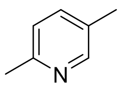

L1m

Conversion: 35%
Yield: 30%
Ratio (α:β): 5:95
2 Decomp.: 28%

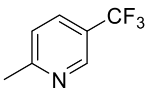

L1c

Conversion: 46%
Yield: 36%
Ratio (α:β): 4:96
2 Decomp.: 21%

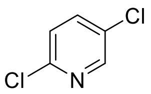

L1n

Conversion: 35%
Yield: 22%
Ratio (α:β): 4:96
2 Decomp.: 24%

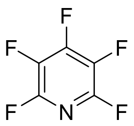

L1o

Conversion: 13%
Yield: 4%
Ratio (α:β): 0:100
2 Decomp.: 31%

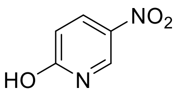

L1p

Conversion: 26%
Yield: 12%
Ratio (α:β): 0:100
2 Decomp.: 27%

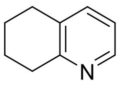

L1q

Conversion: 34%
Yield: 30%
Ratio (α:β): 5:95
2 Decomp.: 29%

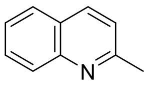

L1r

Conversion: 28%
Yield: 27%
Ratio (α:β): 2:98
2 Decomp.: 21%

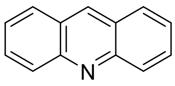

L1g

Conversion: 26%
Yield: 25%
Ratio (α:β): 3:97
2 Decomp.: 25%

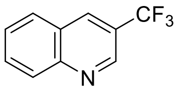

L1f

Conversion: 49%
Yield: 27%
Ratio (α:β): 5:95
2 Decomp.: 20%

Scheme S14: Monodentate ligand (**L1**) screening – Part 2.

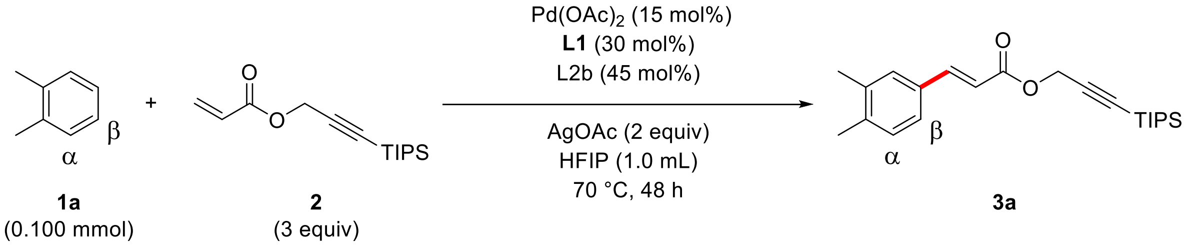

1a (0.100 mmol) + **2** (3 equiv) → **3a**

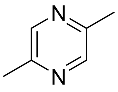

L1i

Conversion: 61%
Yield: 49%
Ratio (α:β): 3:97
2 Decomp.: 15%

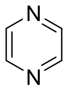

L1h

Conversion: 48%
Yield: 27%
Ratio (α:β): 9:91
2 Decomp.: 10%

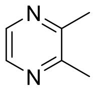

L1b

Conversion: 59%
Yield: 51%
Ratio (α:β): 3:97
2 Decomp.: 13%

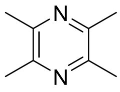

L1s

Conversion: 45%
Yield: 45%
Ratio (α:β): 2:98
2 Decomp.: 16%

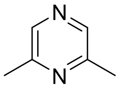

L1t

Conversion: 45%
Yield: 35%
Ratio (α:β): 7:93
2 Decomp.: 14%

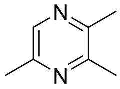

L1u

Conversion: 51%
Yield: 48%
Ratio (α:β): 3:97
2 Decomp.: 13%

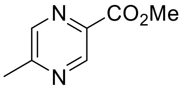

L1v

Conversion: 42%
Yield: 24%
Ratio (α:β): 2:98
2 Decomp.: 18%

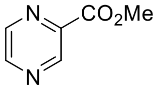

L1x

Conversion: 38%
Yield: 18%
Ratio (α:β): 4:96
2 Decomp.: 19%

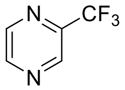

L1j

Conversion: 32%
Yield: 19%
Ratio (α:β): 7:93
2 Decomp.: 11%

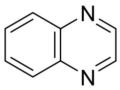

L1l

Conversion: 58%
Yield: 44%
Ratio (α:β): 4:96
2 Decomp.: 17%

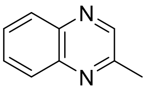

L1y

Conversion: 55%
Yield: 41%
Ratio (α:β): 3:97
2 Decomp.: 18%

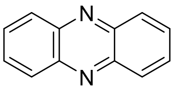

L1k

Conversion: 53%
Yield: 45%
Ratio (α:β): 2:98
2 Decomp.: 17%

Scheme S15: Monodentate ligand (**L1**) screening – Part 3.

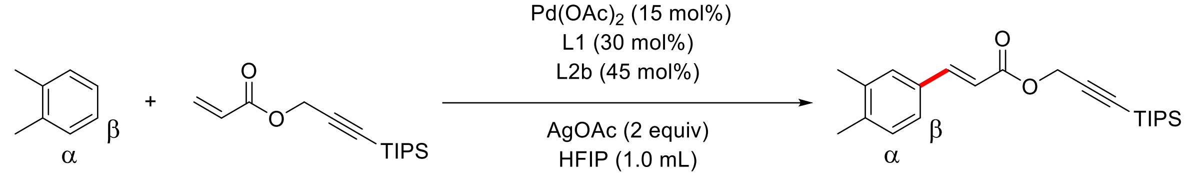

1a (0.100 mmol) | **2** (3 equiv) | **3a**

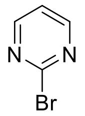

L1z

Conversion: 36%
Yield: 17%
Ratio (α:β): 7:93
2 Decomp.: 11%

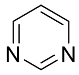

L1aa

Conversion: 53%
Yield: 24%
Ratio (α:β): 8:92
2 Decomp.: 18%

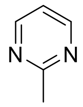

L1ab

Conversion: 57%
Yield: 36%
Ratio (α:β): 3:97
2 Decomp.: 13%

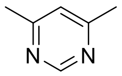

L1ac

Conversion: 61%
Yield: 39%
Ratio (α:β): 3:97
2 Decomp.: 14%

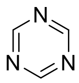

L1ad

Conversion: 20%
Yield: 10%
Ratio (α:β): 6:94
2 Decomp.: 4%

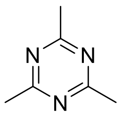

L1ae

Conversion: 39%
Yield: 25%
Ratio (α:β): 2:98
2 Decomp.: 16%

Scheme S16: Time and temperature screening with new catalyst.

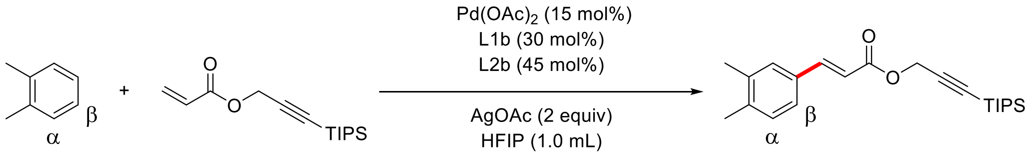

1a (0.100 mmol) | **2** (3 equiv) | **3a**

Entry	Temperature (°C)	Time (h)	**1a** Conv. (%)	Yield (%)	α:β ratio	**2** Decomp. (%)
1.	50	48	47	43	2:98	8
2.	50	72	50	44	2:98	11
3.	60	48	55	49	3:97	12
4.	60	72	56	48	3:97	14
5.	70	48	59	52	3:97	13
6.	70	72	59	49	3:97	16
7.	90	48	59	50	4:96	15
8.	90	72	56	50	4:96	15

Scheme S17: Solvent screening.

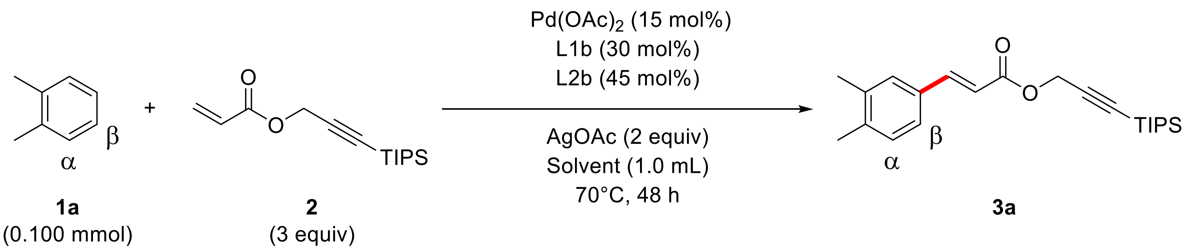

1a (0.100 mmol) + **2** (3 equiv) → **3a**

Entry	Solvent	**1a** Conv. (%)	Yield (%)	α:β ratio	**2** Decomp. (%)
1.	HFIP	59	52	3:97	13
2.	DCE	56	50	3:97	13
3.	TFE	75	54	3:97	13
4.	$CHCl_3$	41	36	3:97	4
5.	HFIP:DCE 9:1	67	46	3:97	12
6.	HFIP:DCE 7:3	69	52	2:98	12
7.	HFIP:DCE 1:1	75	57	2:98	12
8.	HFIP:DCE 3:7	75	59	2:98	11
9.	HFIP:DCE 2:8	71	62	2:98	8
10.	HFIP:DCE 15:85	67	59	2:98	7
11.	HFIP:DCE 1:9	64	57	2:98	8
12.	HFIP:DCE 5:95	57	56	3:97	5
13.	HFIP:TFE 1:1	59	48	3:97	18
14.	TFE:DCE 1:9	74	58	3:97	8
15.	TFE:DCE 2:8	70	58	2:98	12
16.	HFIP:$CHCl_3$ 1:1	70	55	2:98	15
17.	HFIP:$CHCl_3$ 2:8	73	63	2:98	4
18.	TFE:$CHCl_3$ 25:75	75	63	2:98	6
19.	TFE:$CHCl_3$ 2:8	71	66	2:98	4
20.	TFE:$CHCl_3$ 15:85	68	59	2:98	7
21.	TFE:$CHCl_3$ 1:9	62	56	2:98	7

HFIP: 1,1,1,3,3,3-Hexafluor-2-propanol; DCE: 1,2-Dichloroethane; TFE: 2,2,2-Trifluoroethanol

Scheme S18: Time and temperature screening.

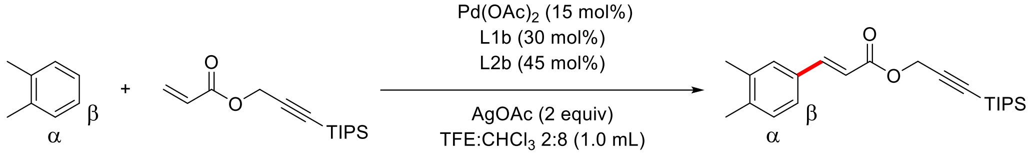

1a
(0.100 mmol)

2
(3 equiv)

3a

Entry	Temperature (°C)	Time (h)	**1a** Conv. (%)	Yield (%)	α:β ratio	**2** Decomp. (%)
1.	60	48	64	61	2:98	1
2.	60	72	64	64	2:98	3
3.	70	48	69	64	2:98	9
4.	70	72	71	64	2:98	10
5.	90	48	68	60	3:97	20
6.	100	48	64	55	3:97	26
7.	120	48	52	36	3:97	60

Scheme S19: Comparison with L1a and L2a.

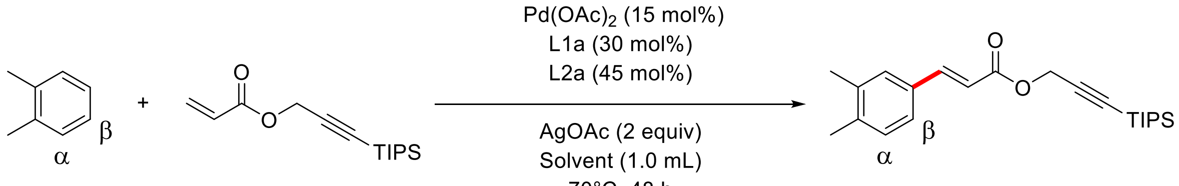

1a
(0.100 mmol)

2
(3 equiv)

3a

Entry	Solvent	**1a** Conv. (%)	Yield (%)	α:β ratio	**2** Decomp. (%)
1.	HFIP	51	33	6:94	20
2.	DCE	12	10	10:90	1
3.	HFIP:DCE 2:8	26	24	7:93	2
4.	TFE:$CHCl_3$ 2:8	29	28	6:94	2
5.	TFE:$CHCl_3$ 2:8 (72 h)	33	32	6:94	2

Scheme S20: Catalyst loading screening.

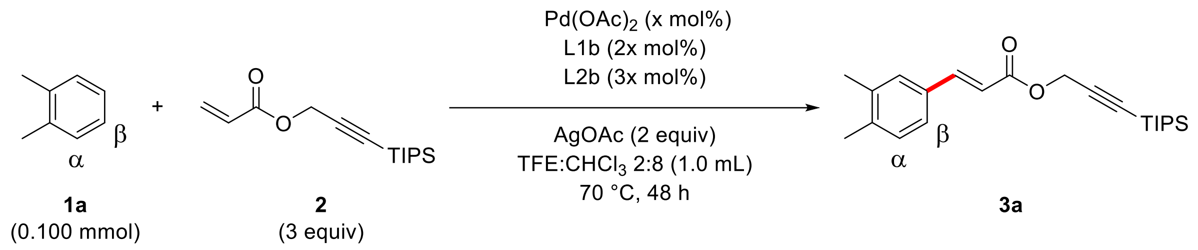

Entry	x	**1a** Conv. (%)	Yield (%)	α:β ratio	**2** Decomp. (%)
1.	15	69	64	2:98	9
2.	10	64	60	2:98	4
3.	5	41	40	2:98	0

Scheme S21: Stoichiometry variation screening.

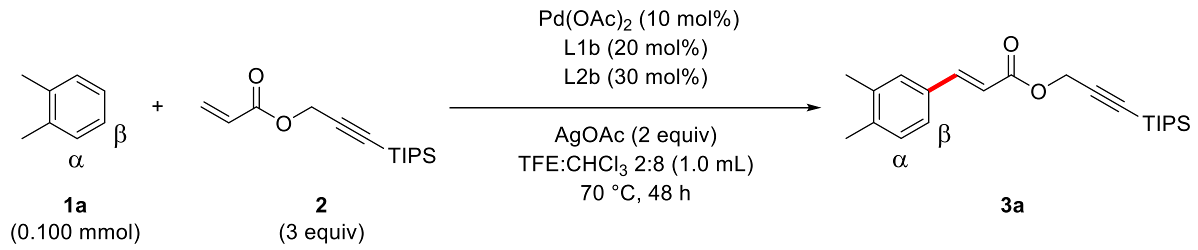

Entry	Variation	**1a** Conv. (%)	Yield (%)	α:β ratio	**2** Decomp. (%)
1.	No variations	64	60	2:98	4
2.	L2b (10 mol%)	55	52	2:98	0
3.	L2b (20 mol%)	60	60	2:98	0
4.	AgOAc (3 equiv)	61	59	2:98	3
5.	**2** (2 equiv)	57	56	2:98	4
6.	**2** (1.5 equiv.)	57	52	2:98	8

Scheme S22: Concentration screening.

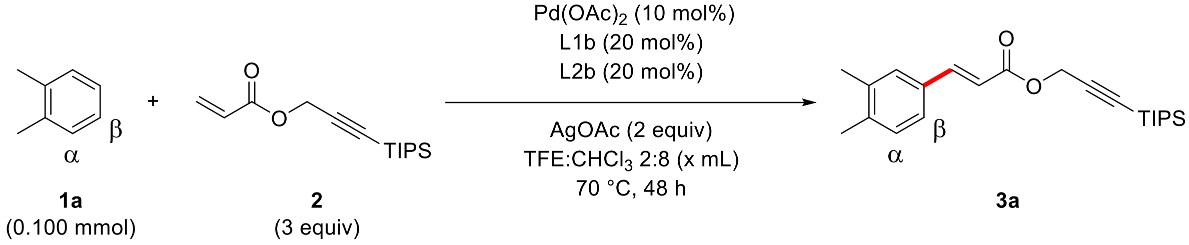

Entry	x	**1a** Conv. (%)	Yield (%)	α:β ratio	**2** Decomp. (%)
1.	0.4	60	55	3:97	2
2.	0.5	68	61	3:97	4
3.	0.6	66	63	3:97	3
4.	0.7	67	63	3:97	4
5.	0.8	67	61	3:97	3
6.	0.9	64	60	2:98	2
7.	1.0	64	59	2:98	2
8.	1.1	66	59	2:98	4
9.	1.2	57	54	2:98	2

Scheme S23: Catalyst and temperature screening for electron-poor substrates.

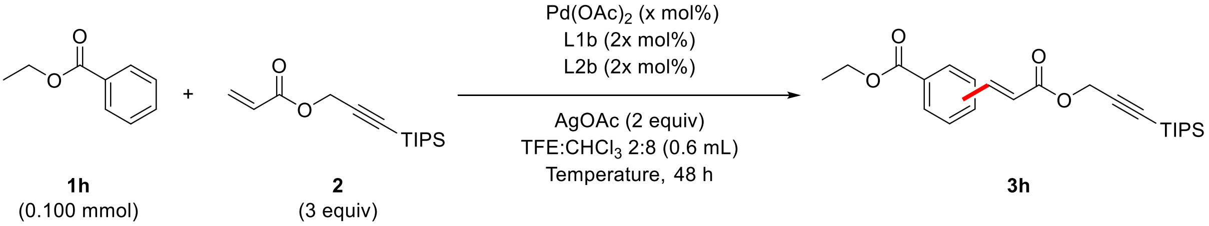

Entry	Temperature (°C)	x	Yield (%)
1.	70	10	16
2.	80	10	20
3.	90	10	23
4.	70	15	23
5.	80	15	35
6.	90	15	35

Yields and ratios were determined by ^{1}H-NMR using 1,3,5-trimethoxybenzene as internal standard.

Scheme S24: Control experiments.

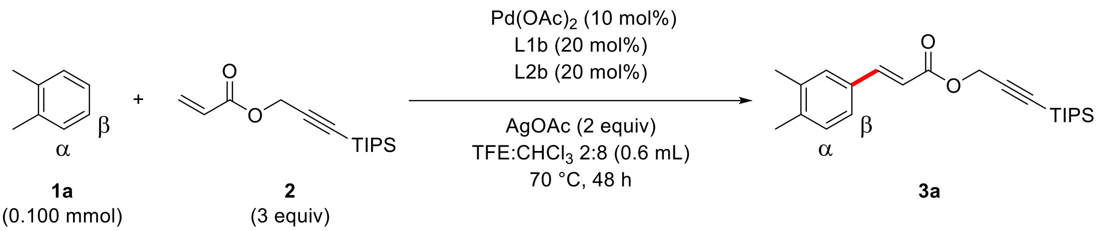

Entry	Variation	Yield (%)
1.	-	64
2.	No L1b	5
3.	No L2b	2
4.	No L1b and L2b	0
5.	No $Pd(OAc)_2$	0

Yields and ratios were determined by ^{1}H-NMR using 1,3,5-trimethoxybenzene as internal standard.

Investigations on the decomposition of 3-(Triisopropylsilyl)prop-2-yn-1-yl acrylate (2)

During the optimization campaign, we established that the decomposition of compound **2** is a critical factor influencing the efficiency of the reaction, showing a clear correlation between reaction yield and the extent of decomposition. Consequently, monitoring the decomposition of **2** was a fundamental part of the reaction optimization process. Minimizing or suppressing the decomposition of **2** proved to be essential for achieving optimal reaction conditions. Two key parameters were identified as major contributors to the decomposition of **2**: reaction temperature (as already shown in Schemes S13 and S18) and the solvent system employed. Notably, replacing HFIP with a TFE:$CHCl_3$ solvent system led to a significant reduction in the decomposition of **2** (Scheme S17).

On this basis, we aimed to elucidate the origin of this behavior by identifying the side products arising from the decomposition. A series of test reactions was conducted, and the reaction outcomes were analyzed by GC-MS. The corresponding reaction conditions, GC-MS chromatograms for each entry, extracted mass spectra of the peaks, and product assignments are shown below.

For comparison, the GC-MS spectrum of compound **2** used in these experiments is also provided. As evident from the chromatogram of compound **2**, signals with retention times of 11.8 and 4.1 min were already present as trace impurities in the starting material. Consequently, these components were not considered in the identification and assignment of

reaction-derived products. Furthermore, the compound with a retention time of 11.3 min (in Entries 1 and 2) was also excluded from the analysis, as it corresponds to 1,3,5-trimethoxybenzene, which was added after completion of the reaction as an internal standard.

Scheme S25: 3-(Triisopropylsilyl)prop-2-yn-1-yl acrylate (**2**) decomposition test.

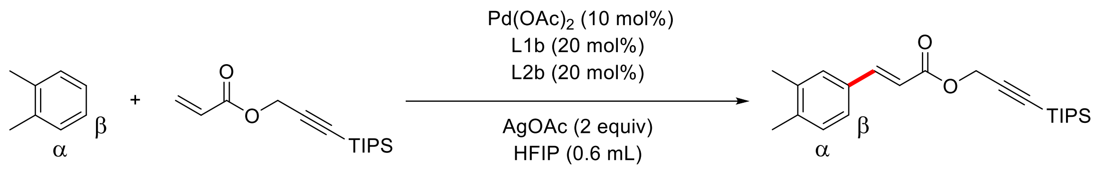

1a (0.100 mmol) **2** (3 equiv) **3a**

Entry	Variation
1.	TFE:$CHCl_3$ (0.6 mL)
2.	TFE:$CHCl_3$ (0.6 mL), 100 °C
3.	/
4.	No **1a**
5.	No AgOAc, ligands and **1a**
6.	No $Pd(OAc)_2$, ligands and **1a**

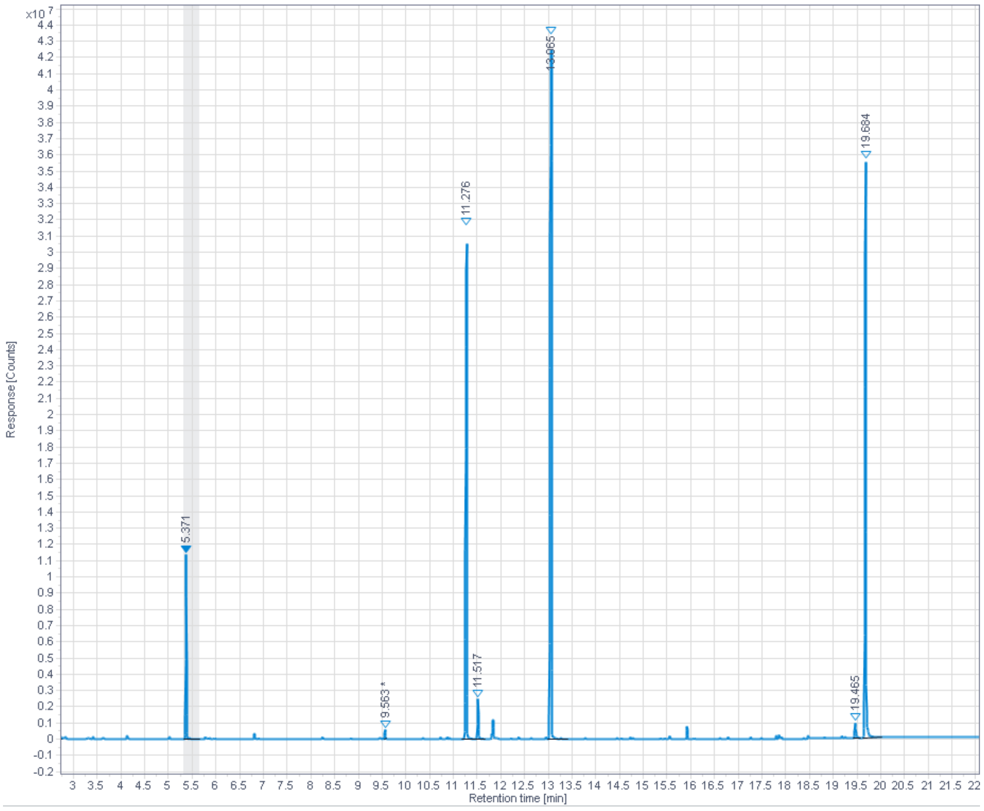

Figure 1. GC-MS chromatogram of Entry 1

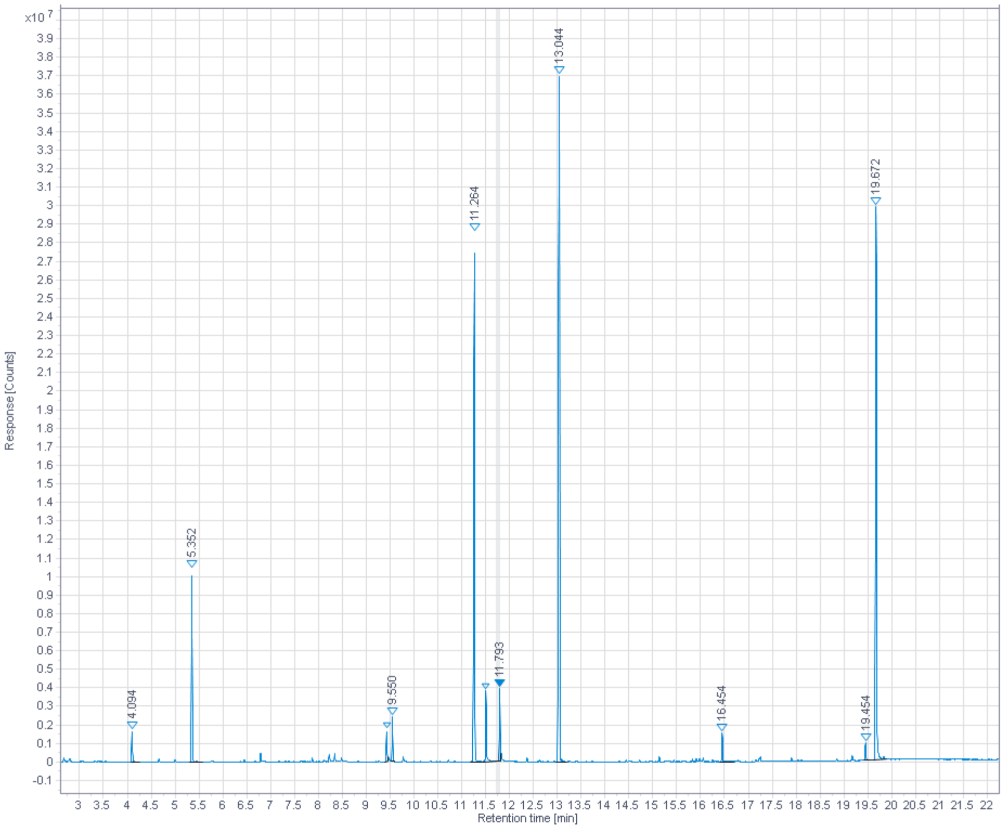

Figure 2. GC-MS chromatogram of Entry 2

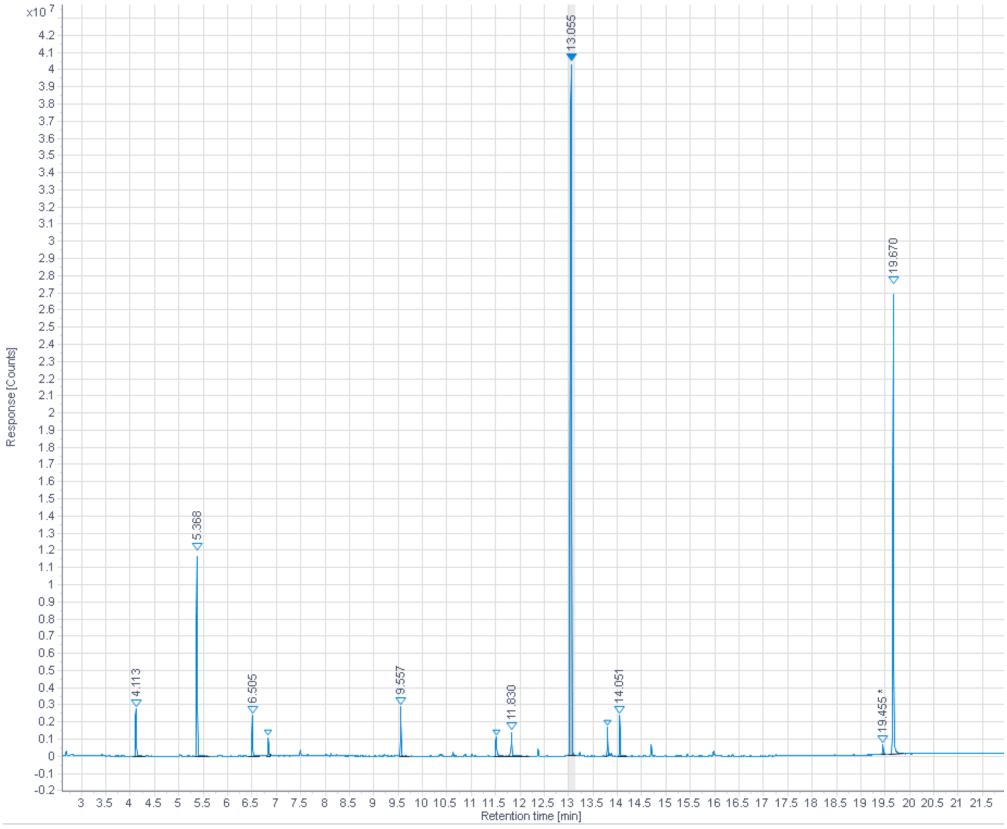

Figure 3. GC-MS chromatogram of Entry 3

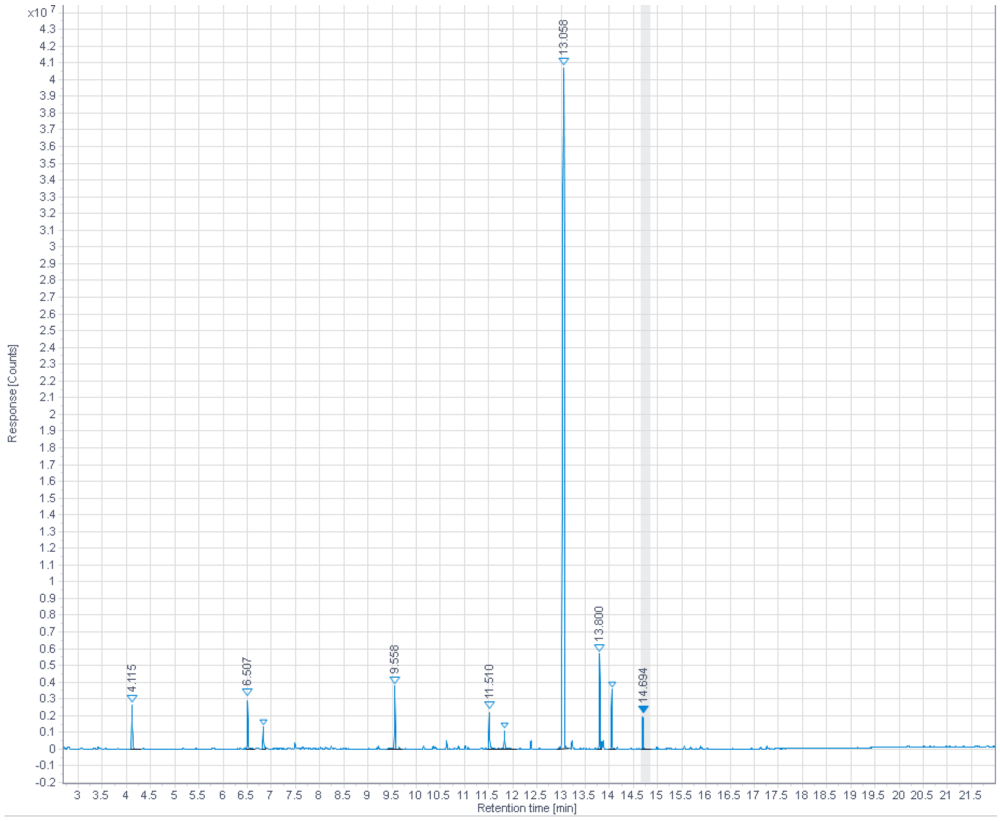

Figure 4. GC-MS chromatogram of Entry 4

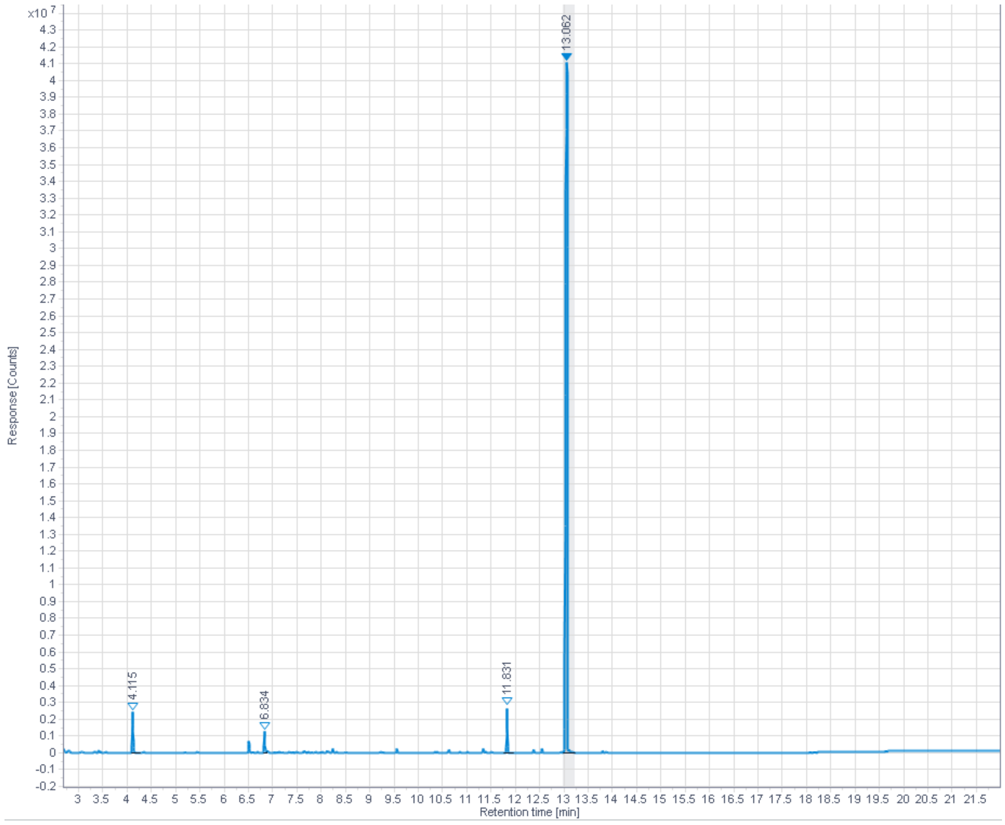

Figure 5. GC-MS chromatogram of Entry 5

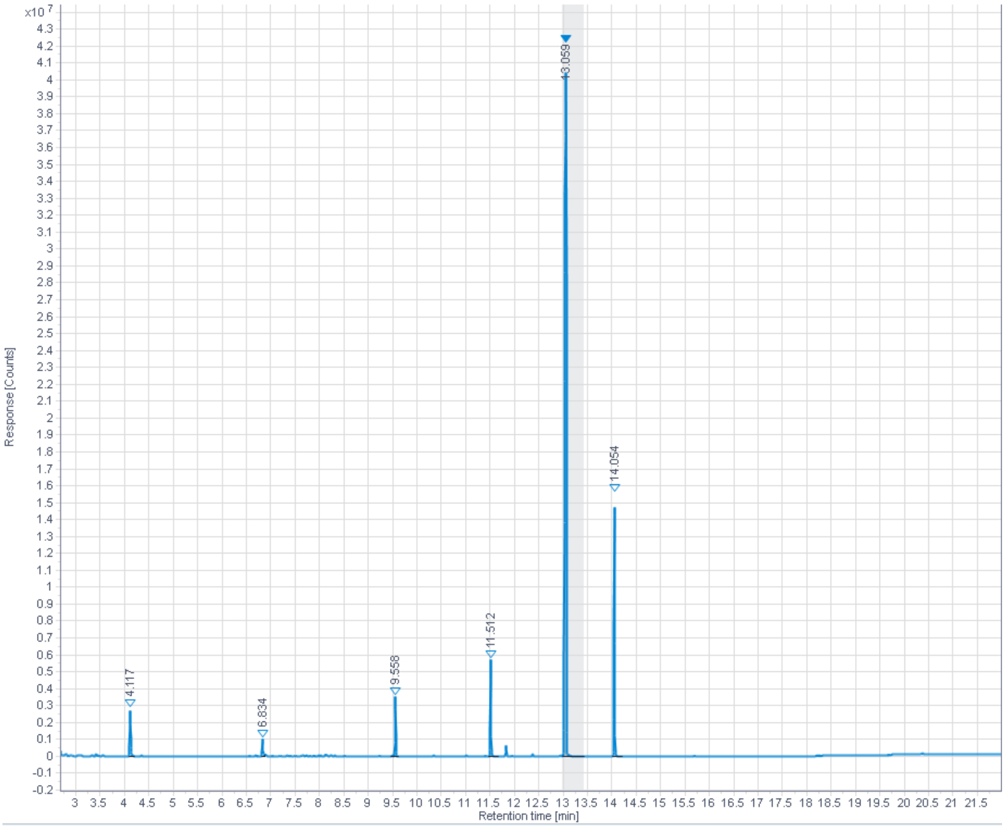

Figure 6. GC-MS chromatogram of Entry 6

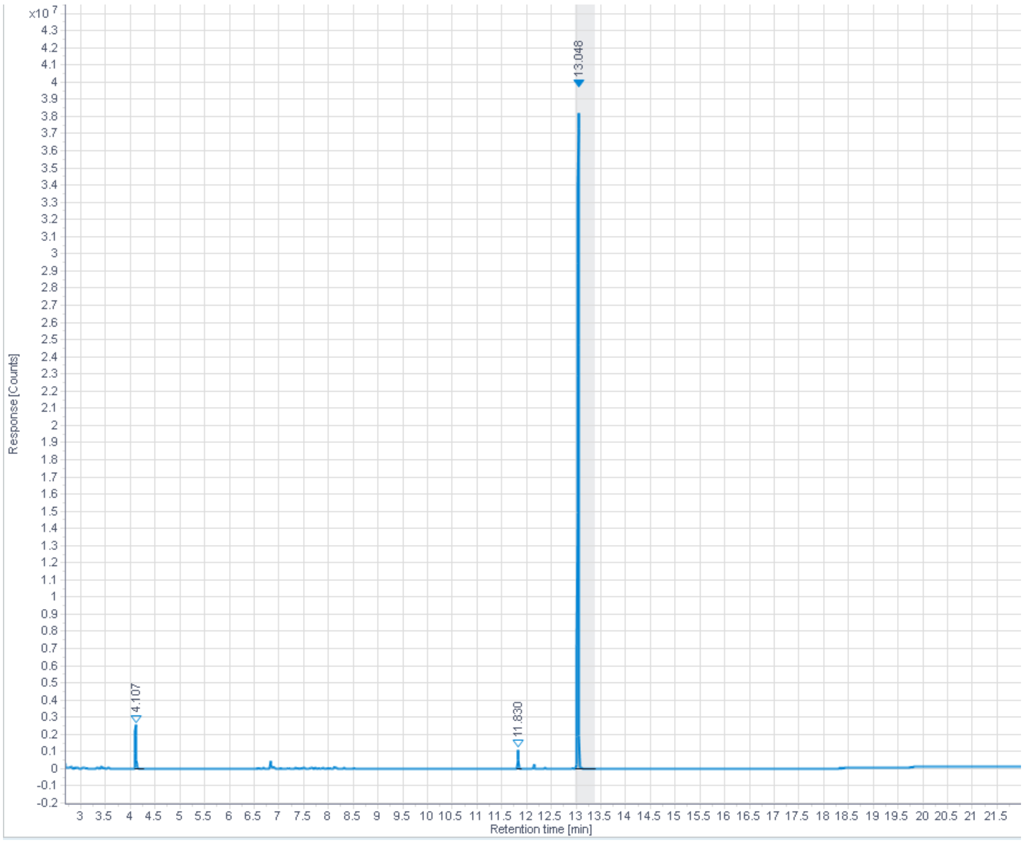

Figure 7. GC-MS chromatogram of compound **2**

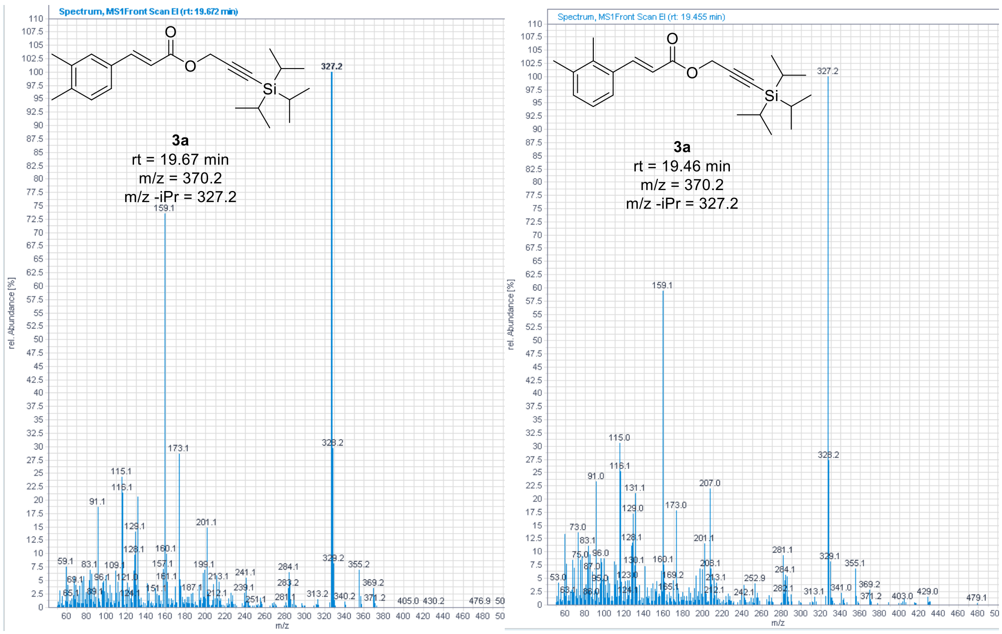

Figure 8. MS spectra of compound **3a**

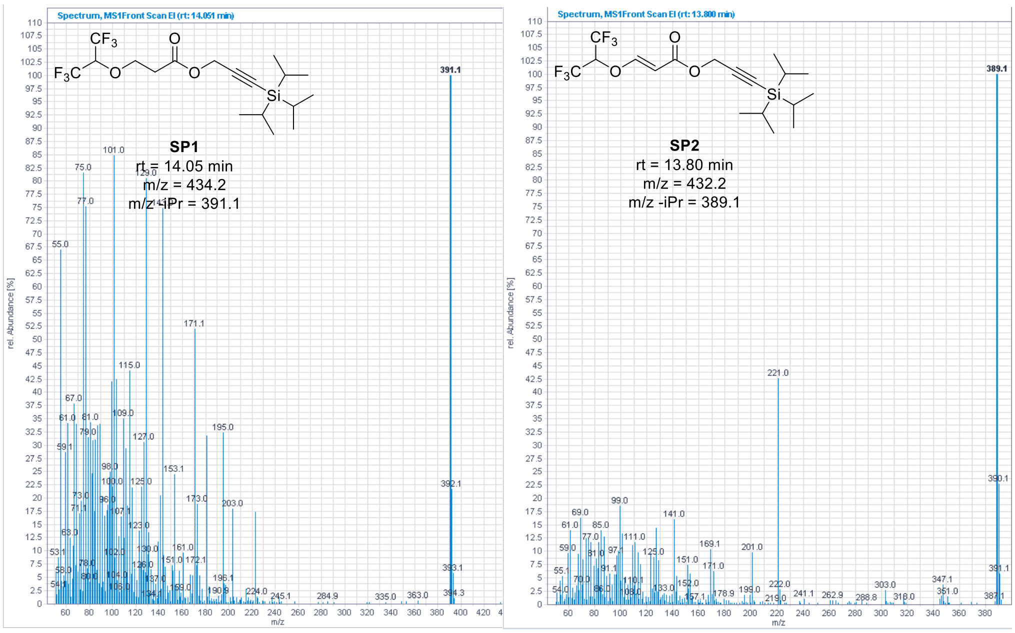

Figure 9. MS spectra of compounds **SP1** and **SP2**

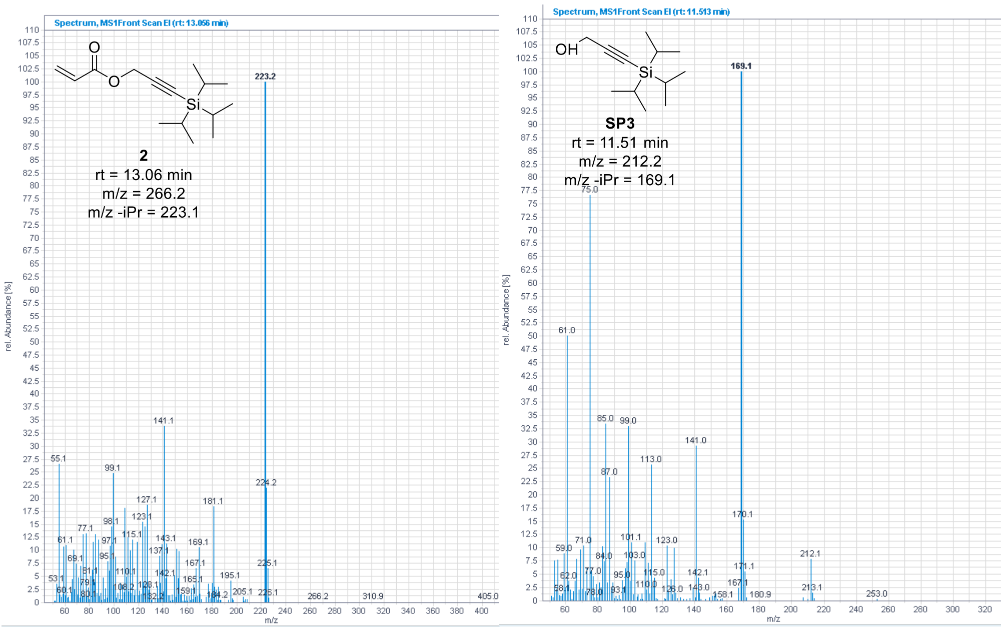

Figure 10. MS spectra of compounds **2** and **SP3**

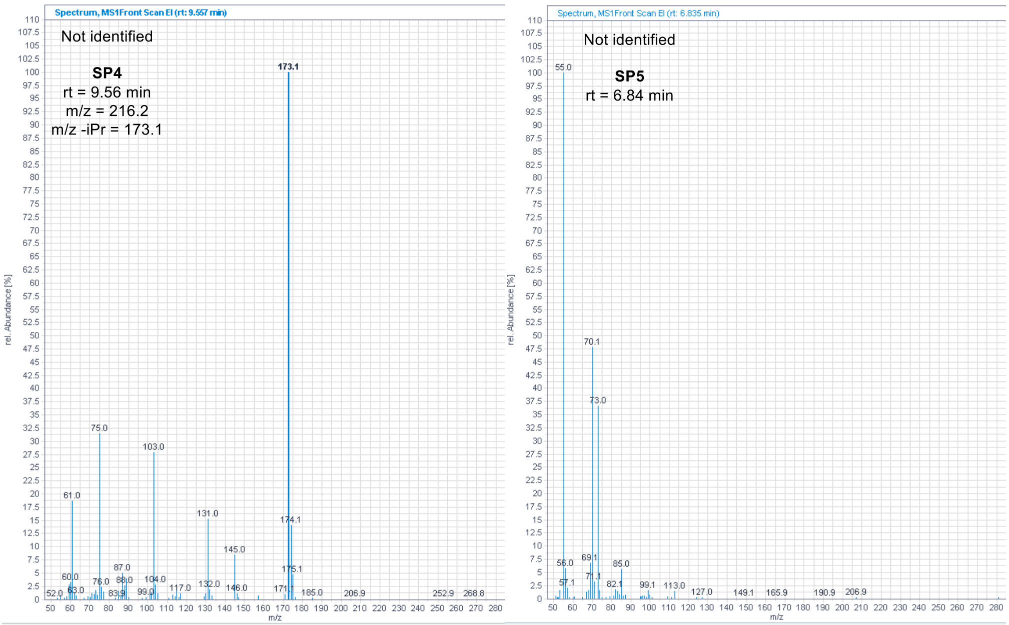

Figure 11. MS spectra of compounds **SP4** and **SP5**

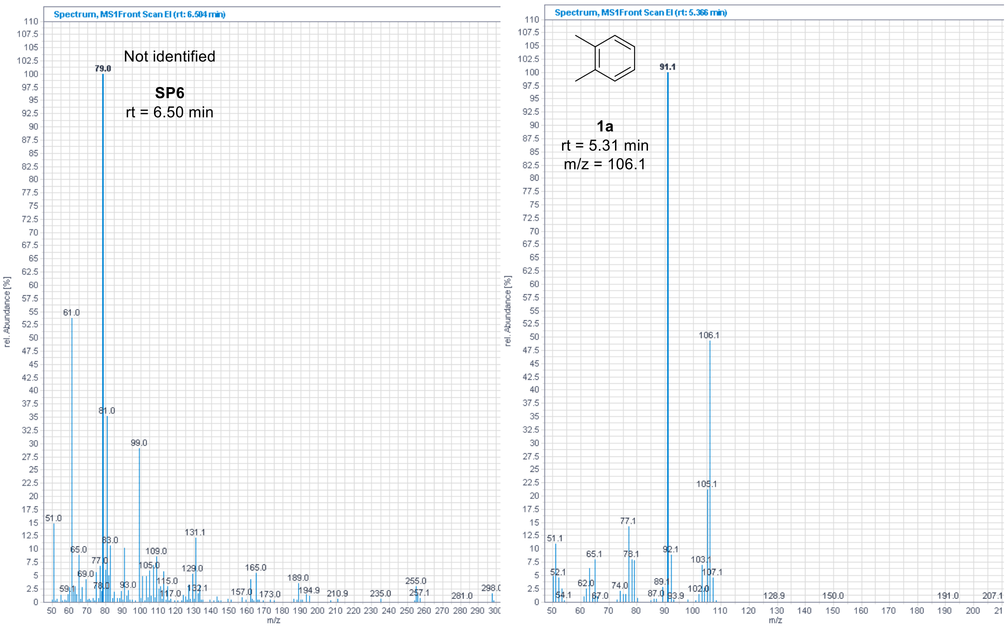

Figure 12. MS spectra of compounds **SP6** and **1a**

Additionally, we confirmed the structures of **SP1** and **SP2** by HRMS-ESI analysis of the crude mixture of entry 3.

For **SP1**: **HRMS (ESI+H$^+$) m/z:** Calculated for $C_{18}H_{29}F_6O_3Si_1$ 435.17119, Found 435.1784.

For **SP2**: **HRMS (ESI+H$^+$) m/z:** Calculated for $C_{18}H_{27}F_6O_3Si_1$ 433.15554, Found 433.1613.

Due to the low concentration of **SP3, SP4, SP5**, and **SP6** in the reaction mixture it was not possible to reliably determine a corresponding peak in the ESI spectrum. The structure of **SP3** was therefore confirmed by comparison with a sample of 3-(triisopropylsilyl)prop-2-yn-1-ol (**SP3**), synthesized via an alternative synthetic route. The chromatogram and MS spectrum of compound **SP3** are shown below for comparison.

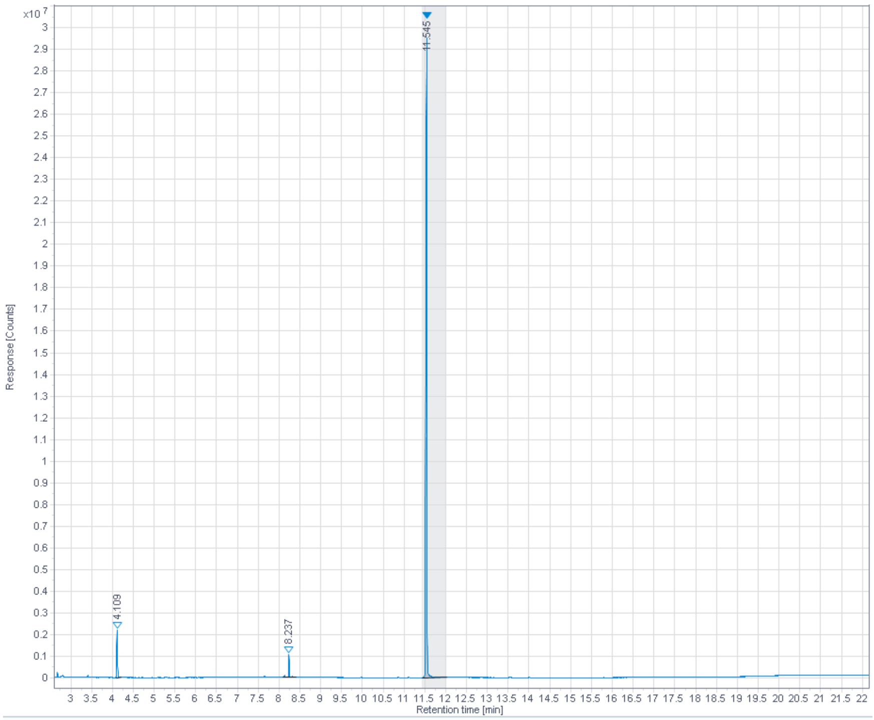

Figure 13. GC-MS chromatogram of compound **SP3**

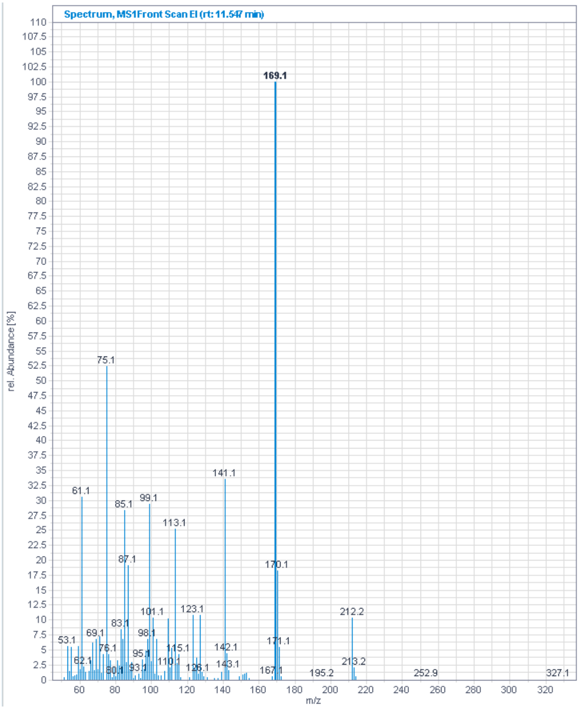

Figure 14. MS spectrum of compound **SP3**

From the results, it is evident that the reaction under the final conditions (Entry 1) resulted in significantly lower amounts of side products than the reaction conducted in HFIP (Entry 3). A temperature above 70 °C led to the formation of larger quantities as well as additional side products (Entry 2).

Across the tested conditions, the only side products consistently observed were **SP3**, the hydrolyzed alcohol, and **SP4**, which could not be characterized reliably.
In contrast, Entry 3 additionally showed the formation of several HFIP-derived side products, including those arising from Michael addition of HFIP to the double bond of compound **2** (**SP1**), **SP2**, supposedly formed by Pd-mediated addition of HFIP followed by β-hydride elimination or a Saegusa-Ito-like oxidation of **SP1**, as well as two unidentified species (**SP5** and **SP6**).
The comparison between Entry 3 and Entries 4–6 clearly indicates that the formation of **SP1** is triggered by the presence of AgOAc and HFIP, whereas **SP2** is generated only when AgOAc, Pd, and HFIP are all present. The side products **SP3** and **SP4** are always formed in the presence of AgOAc. Formation of **SP5** requires both HFIP and AgOAc, while **SP6** is observed exclusively when Pd and HFIP are combined.
Overall, these observations indicate that the improved efficiency observed under the final conditions arises from the greater stability of compound **2** in the TFE:$CHCl_3$ solvent system compared to HFIP and a fine tuning of the reaction temperature, resulting in an almost complete suppression of the reagent.

Comparison with reported systems

The comparison of our results with those reported by Yao *et al.* highlights the broader applicability of our method, particularly with respect to the functionalization of electron-poor substrates.[3] Notably, in their Supporting Information, the authors report that no product formation was observed when methyl benzoate was used as the substrate, whereas under our conditions the corresponding product could be isolated in 30% yield using ethyl benzoate as the starting material (compound **3h**). However, our catalytic system does not tolerate free phenolic moieties (see **list of poorly performing and failed entries**), while the method developed by Yao et al. is specifically optimized for this class of substrates. Overall, the two approaches can be regarded as complementary with respect to functional group tolerance.

To support our optimization efforts, we compared our results with the conditions developed by Yu *et al.* for the synthesis of β-arylethenesulfonyl fluorides suitable for SuFEx transformations, employing 3,5-bis(trifluoromethyl)-2(1H)-pyridinone as ligand in HFIP.[4] In addition, we explored whether ethenesulfonyl fluoride could serve as an alternative clickable partner under our reaction conditions.

Scheme S26: Comparison with already reported systems.

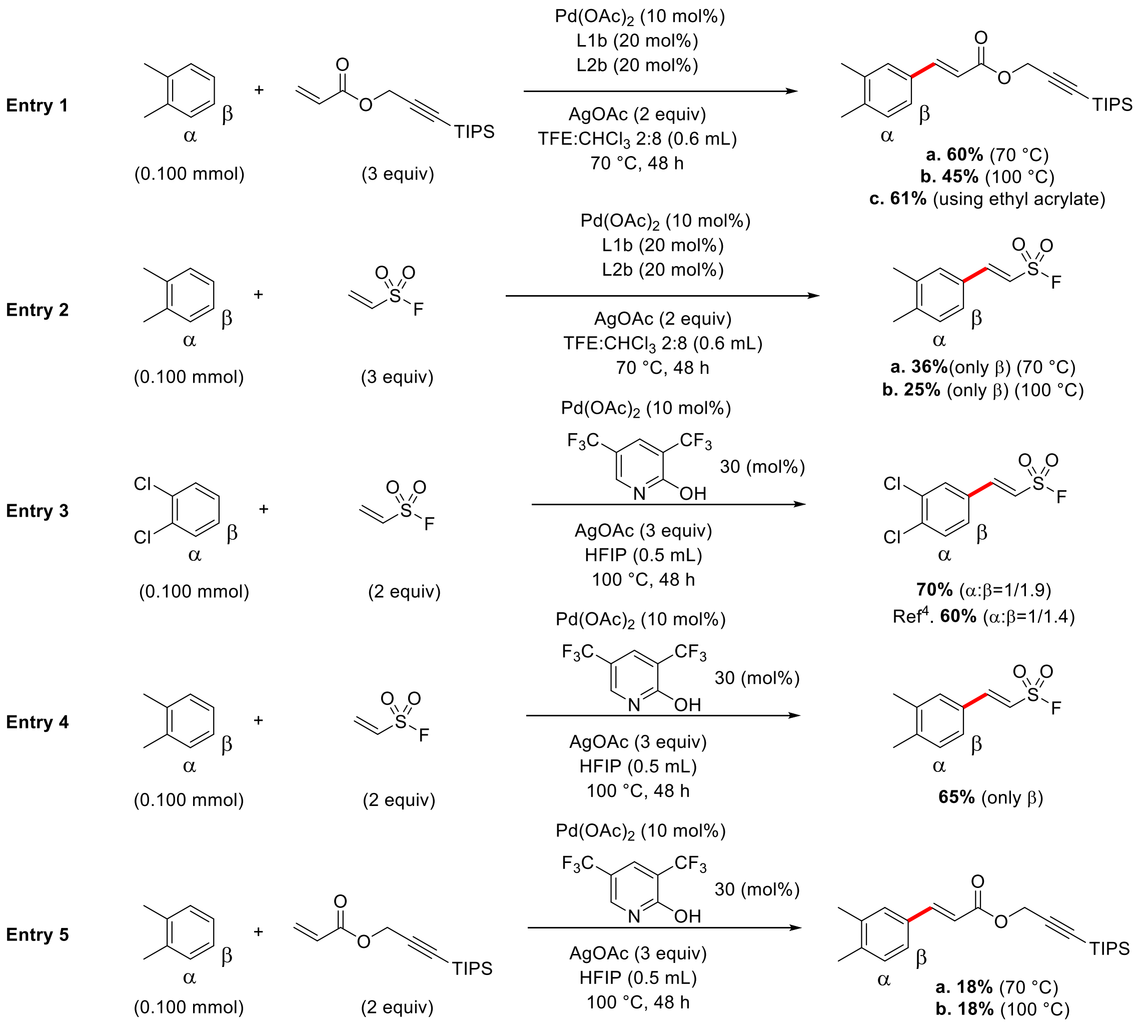

Yields were determined by ^{1}H-NMR using 1,3,5-trimethoxybenzene as internal standard.

Entry 1 summarizes the optimized reaction conditions (Entry 1a), along with the results obtained at elevated temperature (Entry 1b) and when ethyl acrylate was employed instead of the alkyne-containing acrylate (Entry 1c). As discussed above, mild conditions are essential to minimize acrylate decomposition and to achieve higher yields. Notably, the use of ethyl acrylate under the optimized conditions (Entry 1c) afforded a virtually identical yield as the alkyne-containing acrylate (Entry 1a). This result confirms that, under the optimized conditions, acrylate decomposition is no longer a limiting factor.

Under our optimized conditions, an NMR yield of 36% of the arylethenesulfonyl fluoride was obtained when using ethenesulfonyl fluoride as coupling partner (Entry 2a); increasing the reaction temperature led to a decreased yield (Entry 2b).

Entry 3 was performed to confirm the reproducibility of the results reported by Yu et al. with the chemicals and equipment available in our lab. We obtained slightly higher yields, which can be attributed to the prolonged reaction time (48 h instead of the 24 h used in literature). Note that a commercially available ligand, not the final optimized ligand is used to compare the methods. Using o-xylene as substrate with the commercially available ligand, the desired arylethenesulfonyl fluoride was obtained in 65% yield under Yu's conditions (Entry 4). Notably, using our alkyne-containing acrylate as olefin under Yu's conditions afforded the desired product in only 18% yield (Entry 5a-b).

A comparison of Entries 1 and 6 demonstrates that our catalytic system provides superior yields for the clickable olefin as reaction partner by operating under milder conditions and avoiding the use of HFIP. These results additionally indicate that other acrylic moieties are tolerated by our conditions; however, with olefins that are less-prone to decomposition, harsher reaction conditions can be applied and deliver increased yields.

3. Ligand Synthesis

Ligands **L1a-ae** and **L2a-o** were purchased from ABCR, Acros Organics, BLD-pharm, Alfa Aesar, Fluorochem, Sigma Aldrich, or TCI Europe.

Ligands **L2p-am** were available in our laboratory or were synthesized according to literature procedures.[5,6] The ^{1}H-NMR spectra of the isolated material matched with the ones reported in the literature.

Ligands **L2an-ap and L2b** were synthesized adapting a literature procedure[6], as follows: 4-(dimethylamino)pyridine (DMAP) (489 mg, 4.00 mmol, 2.0 equiv) was added to a suspension of 1-ethyl-3-(3-dimethylaminopropyl)carbodiimide hydrochloride (EDC·HCl) (499 mg, 2.40 mmol, 1.2 equiv) in CH_2Cl_2 (5 mL, 0.4 M). The mixture was stirred at room temperature until all the solids had dissolved. The mixture was cooled to 0°C, the corresponding acid (2.00 mmol) was added, followed by the corresponding sulfonamide (2.40 mmol, 1.2 equiv) and the mixture was stirred at 0°C for 4 hours, allowed to warm up to room temperature, and stirred overnight. The reaction was acidified with concentrated aq. HCl to reach a pH value of 1 and extracted with EtOAc (4 × 20 mL). The combined organic phases were dried over $MgSO_4$, filtered, and concentrated under reduced pressure. The crude product was purified by flash column chromatography or recrystallization.

2-acetamido-*N*-((perfluorophenyl)sulfonyl)acetamide (L2b)

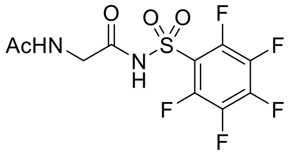

L2b

Ligand **L2b** was synthesized according to the general procedure using *N*-acetylglycine (234 mg, 2.00 mmol) and 2,3,4,5,6-pentafluorobenzenesulfonamide (600 mg, 2.40 mmol, 1.2 equiv) in dry CH_2Cl_2 (5 mL, 0.4 M). The crude product was purified by silica gel column chromatography using CH_2Cl_2:MeOH:HCO_2H 96:4:0.5 as the eluent and the target compound **L2b** was obtained as a colorless solid (485 mg, 1.40 mmol, 70%).

^{1}H-NMR (500 MHz, DMSO-d6): δ = 8.11 (t, J = 5.7 Hz, 1H), 3.79 (d, J = 5.7 Hz, 2H), 1.80 (s, 3H) ppm.
^{13}C-NMR (126 MHz, DMSO-d6): δ = 169.7, 169.6, 144.4 (m), 143.7 (m), 137.1 (m), 122.8, 42.3, 22.0 ppm.
^{19}F-NMR (471 MHz, DMSO-d6): δ = -136.5, -145.2, -159.7 ppm.
HRMS (ESI-neg) m/z: Calculated for $C_{10}H_6O_4N_2F_5S_1$ 344.99739, Found 344.99732.

2-acetamido-*N*-((4-(trifluoromethyl)phenyl)sulfonyl)acetamide (L2an):

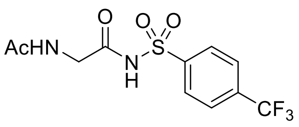

L2an

Ligand **L2an** was synthesized according to the general procedure using *N*-acetylglycine (234 mg, 2.00 mmol) and 4-(trifluoromethyl)benzenesulfonamide (495 mg, 2.20 mmol, 1.1 equiv) in dry CH_2Cl_2 (5 mL, 0.4 M). The crude product was purified by silica gel column chromatography using CH_2Cl_2:MeOH:HCO_2H 96:4:0.5 as the eluent and the target compound **L2an** was obtained as a colorless solid (292 mg, 900 µmol, 45%).

^{1}H-NMR (500 MHz, DMSO-d6): δ = 8.12 (d, J = 8.2 Hz, 2H), 8.07 (t, J = 5.8 Hz, 1H), 8.02 (d, J = 8.2 Hz, 2H), 3.76 (d, J = 5.8 Hz, 2H), 1.79 (s, 3H) ppm.
^{13}C-NMR (126 MHz, DMSO-d6): δ = 169.7, 169.0, 143.4, 133.0 (m), 128.5, 126.4 (m), 123.4 (m), 42.2, 22.2 ppm.
^{19}F-NMR (471 MHz, DMSO-d6): δ = -61.3 ppm.
HRMS (ESI-neg) m/z: Calculated for $C_{11}H_{12}O_4N_2F_3S$ 325.04644, Found 325.04616.

2-acetamido-*N*-((2-(trifluoromethyl)phenyl)sulfonyl)acetamide (L2ao):

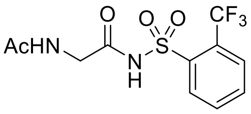

L2ao

Ligand **L2ao** was synthesized according to the general procedure using *N*-acetylglycine (234 mg, 2.00 mmol) and 2-(trifluoromethyl)benzenesulfonamide (495 mg, 2.20 mmol, 1.1 equiv) in dry CH_2Cl_2 (5 mL, 0.4 M). The crude product was purified by crystallization in EtOAc and the target compound **L2ao** was obtained as a colorless solid (573 mg, 1.76 mmol, 88%).

^{1}H-NMR (500 MHz, DMSO-d6): δ = 8.32 – 8.28 (m, 1H), 8.09 (t, J = 5.8 Hz, 1H), 8.01 (dd, J = 7.1, 2.1 Hz, 1H), 7.96 – 7.89 (m, 2H), 3.78 (d, J = 5.8 Hz, 2H), 1.79 (s, 3H) ppm.
^{13}C-NMR (126 MHz, DMSO-d6): δ = 169.7, 168.5, 137.3, 134.3, 133.1, 132.9, 128.4, 128.3, 126.2 (m), 122.5 (m), 42.03, 22.16 ppm.
^{19}F-NMR (471 MHz, DMSO-d6): δ = -55.8 ppm.
HRMS (ESI-neg) m/z: Calculated for $C_{11}H_{12}O_4N_2F_3S$ 325.04644, Found 325.04628.

2-acetamido-*N*-((trifluoromethyl)sulfonyl)acetamide (L2ap):

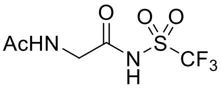

L2ap

Ligand **L2ap** was synthesized according to the general procedure using *N*-acetylglycine (234 mg, 2.00 mmol) and trifluoromethanesulfonamide (327.4 mg, 2.20 mmol, 1.1 equiv) in dry CH_2Cl_2 (5 mL, 0.4 M). The crude product was purified by silica gel column chromatography using CH_2Cl_2:MeOH:HCO_2H = 96:4:0.5 as the eluent and the target compound **L2ap** was obtained as a colorless solid (333 mg, 1.34 mmol, 67%).

^{1}H-NMR (500 MHz, DMSO-d6): δ = 7.90 (s, 1H), 3.65 (s, 2H), 1.83 (s, 3H) ppm.

^{13}C-NMR (126 MHz, DMSO-d6): δ = 172.61, 169.25, 44.24, 22.43 ppm.

^{19}F-NMR (471 MHz, DMSO-d6): δ = -76.8 ppm.

HRMS (ESI-neg) m/z: Calculated for $C_5H_8O_4N_2F_3S_1$ 249.01514, Found 249.01501.

4. Starting Material Synthesis

3-(Triisopropylsilyl)prop-2-yn-1-yl acrylate (2)

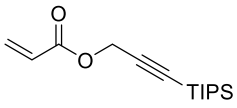

2

Compound **2** was synthetized adapting a literature procedure by Maeda et al.[7], as follows: *n*-BuLi 2.5 M in hexane (13.2 mL, 33.0 mmol, 1.1 equiv) was added dropwise to a solution of ethynyltriisopropylsilane (6.70 mL, 4.45 g, 30.0 mmol) in dry THF (60 mL) at −78 °C under a N_2 atmosphere. After stirring the solution for 0.5 h, paraformaldehyde (1.35 g, 45.0 mmol, 1.5 equiv) was added, the resulting mixture was allowed to warm to room temperature, and stirring was continued over-night. The suspension was cooled to 0 °C and acryloyl chloride (2.70 mL, 3.00 g, 33.4 mmol, 1.1 equiv) was added. The mixture was further stirred for 2 h, quenched with $NaHCO_3$ (15 mL) and extracted with CH_2Cl_2 (3 X 15 mL). After washing the solution with brine and water (30 mL), the combined organic layer was dried over $MgSO_4$ and the solvent removed under reduced pressure to obtain the crude product. The yellowish crude oil was purified by column chromatography using a gradient of 70:1 to 60:1 *n*-pentane/Et_2O as mobile phase to obtain **2** as a colorless oil (4.04 g, 15.2 mmol, 61%). The spectroscopic data were in agreement with the ones reported in literature.[7]

^{1}H-NMR (500 MHz, $CDCl_3$): δ = 6.45 (dd, *J* = 1.4, 17.3 Hz, 1H), 6.16 (dd, *J* = 7.3, 10.3 Hz, 1H), 5.87 (dd, *J* = 1.4, 10.3 Hz, 1H), 4.79 (s, 2H), 1.07 (m, 21H) ppm.
^{13}C-NMR (126 MHz, $CDCl_3$): δ = 165.5, 131.6, 128.0, 100.9, 88.7, 53.1, 18.7, 11.2 ppm.

***Tert*-butyl methyl(phenethyl)carbamate (1d)**

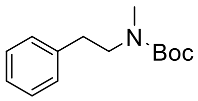

1d

Compound **1d** was synthetized following a literature procedure by Maguire et al.[8] and obtained as transparent oil (693 mg, 2.94 mmol, 98%). The spectroscopic data were in agreement with the ones reported in literature.[8]

^{1}H-NMR (600 MHz, $CDCl_3$): δ = 7.29 (t, *J* = 7.4 Hz, 2H), 7.24 – 7.14 (m, 3H), 3.42 (bs, 2H), 2.79 (m, 5H), 1.42 (m, 9H) ppm.
^{13}C-NMR (151 MHz, $CDCl_3$): δ = 155.7, 139.4, 129.0, 128.6, 126.4, 79.4, 51.0, 50.6, 34.7, 34.4, 34.3, 28.5 ppm.

(*R*)-methyl 2-(*trans*-4-isopropylcyclohexanecarboxamido)-3-phenylpropanoate (1n)

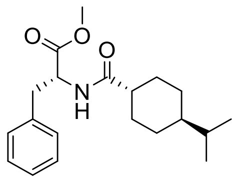

1n

Compound **1n** was synthetized following a modified literature procedure by Ritter et al.[9], as follows: (*R*)-2-(*trans*-4-isopropylcyclohexanecarboxamido)-3-phenylpropanoic acid (349 mg, 1.10 mmol) was dissolved in 3 mL DMF at rt and stirred until the solid was fully dissolved. To the mixture was added K_2CO_3 (300 mg, 2.20 mmol, 2 equiv) and methyl iodide (469 mg, 205 µL, 3.30 mmol, 3 equiv) and stirred for 24 h at rt. The reaction was diluted with ethyl acetate and washed several times with water and brine. The combined organic layer was dried over Na_2SO_4 and the resulting solution concentrated under reduced vacuum. After purification by flash chromatography (6:4 n-pentane/EtOAc) was the product obtained as a colorless solid (346 mg, 1.05 mmol, 95%). The spectroscopic data were in agreement with the ones reported in literature.[9]

^{1}H-NMR (600 MHz, MeOD): δ = 7.32 – 7.26 (m, 2H), 7.25 – 7.18 (m, 3H), 4.67 (dd, *J* = 9.3, 5.5 Hz, 1H), 3.71 (s, 3H), 3.18 (dd, *J* = 13.9, 5.5 Hz, 1H), 2.96 (dd, *J* = 13.9, 9.3 Hz, 1H), 2.12 (m, 1H), 1.85 – 1.73 (m, 3H), 1.67 (m, 1H), 1.47 – 1.26 (m, 3H), 1.08 – 0.96 (m, 3H), 0.89 (s, 3H), 0.89 (s, 3H) ppm.
^{13}C-NMR (151 MHz, MeOD): δ = 177.7, 172.3, 136.9, 128.8, 128.0, 126.4, 53.5, 51.2, 44.8, 43.4, 37.0, 32.7, 29.4, 29.1, 28.7, 28.6, 18.7 ppm.

((2,2-dimethyl-2,3-dihydrobenzofuran-7-yl)oxy)triisopropylsilane (1o)

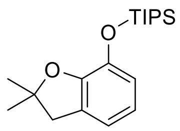

1o

Compound **1o** was synthetized adapting a literature procedure by Glorius et al.[10], as follows: 2,2-dimethyl-2,3-dihydrobenzofuran-7-ol (493 mg, 3.00 mmol) was dissolved in dry CH_2Cl_2 (11 mL) and the solution was cooled to 0°C. Triethylamine (1.08 mL, 788 mg, 7.80 mmol, 2.6 equiv) was added slowly and the mixture was stirred at 0°C for 0.5 h. Chlorotriisopropylsilane (0.830 mL, 755 mg, 3.90 mmol, 1.3 equiv) was added dropwise, the reaction mixture was allowed to warm to room temperature, and stirring was continued for 24 h. The reaction mixture was concentrated under reduced pressure and the crude product purified by column chromatography with *n*-pentane as eluent. Compound **1n** was obtained as yellow oil (960 mg, 3.00 mmol, >99%).

^{1}H-NMR (500 MHz, $CDCl_3$): δ = 6.78 – 6.60 (m, 3H), 2.99 (s, 2H), 1.45 (s, 6H), 1.33 – 1.20 (m, 3H), 1.10 (m, 18H) ppm.
^{13}C-NMR (126 MHz, $CDCl_3$): δ = 149.6, 140.4, 128.6, 120.0, 117.8, 86.5, 43.6, 28.4, 18.0, 13.0 ppm.
HRMS (ESI+NH_4^+) m/z: Calcd for $C_{19}H_{36}O_2N_1S_1$ 338.25098, Found 338.25073.

1,3-Diisopropyl-2-methoxybenzene (1q)

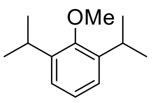

1q

Compound **1q** was synthetized following a literature procedure by Meek et al.[11] and obtained as a pale-yellow oil (326 mg, 1.70 mmol, 85%). The spectroscopic data were in agreement with the ones reported in literature.[11]

^{1}H-NMR (500 MHz, $CDCl_3$): δ = 7.14 – 7.06 (m, 3H), 3.75 (s, 3H), 3.35 (hept, *J* = 6.9 Hz, 2H), 1.25 (s, 6H), 1.24 (s, 6H) ppm.
^{13}C-NMR (151 MHz, $CDCl_3$): δ = 154.7, 141.8, 124.6, 124.2, 62.4, 26.6, 24.2 ppm.

Methyl 2-(4-(2,2-dichlorocyclopropyl)phenyl)-2-methylpropanoate (1t)

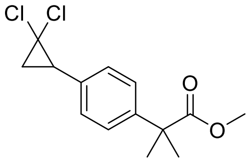

1t

Compound **1t** was synthetized following a literature procedure by Hu et al.[12] and obtained as a colorless solid (217 mg, 0.716 mmol, 99%). The spectroscopic data were in agreement with the ones reported in literature.[12]

^{1}H-NMR (600 MHz, $CDCl_3$): δ = 7.13 – 7.09 (m, 2H), 6.82 – 6.78 (m, 2H), 3.76 (s, 3H), 2.85 – 2.80 (m, 1H), 1.94 (dd, J = 10.7, 7.4 Hz, 1H), 1.77 (dd, J = 8.3, 7.4 Hz, 1H), 1.59 (s, 6H) ppm.
^{13}C-NMR (151 MHz, $CDCl_3$): δ = 174.9, 155.0, 129.8, 128.4, 118.9, 79.3, 61.0, 52.6, 35.0, 26.0, 25.5 ppm.

1-Azido-1-deoxy-2,3,4,6-tetra-O-acetyl-β-D-glucogalactose (5)

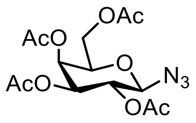

5

Compound **5** was synthetized following a literature procedure by Pringle et al.[13], and obtained as a colorless solid (715 mg, 1.90 mmol, 79%). The spectroscopic data were in agreement with the ones reported in literature.[13]

^{1}H-NMR (600 MHz, $CDCl_3$): δ = 5.42 (ddd, *J* = 3.4, 1.2, 0.4 Hz, 1H), 5.14 (dd, *J* = 10.3, 8.8 Hz, 0.4 Hz), 5.03 (dd, *J* = 10.4, 3.4 Hz, 1H), 4.59 (d, *J* = 8.7 Hz, 1H), 4.20 – 4.10 (m, 2H), 4.00 (ddd, *J* = 6.7, 6.3, 1.2 Hz, 1H), 2.15 (s, 3H), 2.07 (s, 3H), 2.04 (s, 3H), 1.97 (s, 3H) ppm.
^{13}C-NMR (151 MHz, $CDCl_3$): δ = 170.5, 170.2, 170.1, 169.5, 88.4, 73.0, 70.8, 68.2, 67.0, 61.3, 20.8, 20.7, 20.6 ppm.

1-azido-2-(2-(2-azidoethoxy)ethoxy)ethane (7)

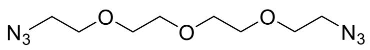

7

Compound **7** was synthetized following a literature procedure by Freitas et al.[14] and obtained as a yellow oil (218 mg, 0.890 mmol, 89%). The spectroscopic data were in agreement with the ones reported in literature.[14]

^{1}H-NMR (500 MHz, $CDCl_3$): δ = 3.70 – 3.65 (m, 12H), 3.41 – 3.37 (m, 4H) ppm.
^{13}C-NMR (126 MHz, $CDCl_3$): δ = 70.7, 70.0, 50.7 ppm.

6-azido-3',6'-dihydroxy-*3H*-spiro[isobenzofuran-1,9'-xanthen]-3-one (10)

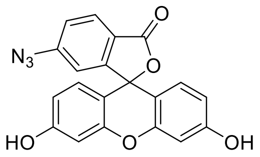

10

Compound **10** was synthetized following a literature procedure by Weber et al.[15], and obtained as an orange solid (477 mg, 1.30 mmol, 91%). The spectroscopic data were in agreement with the ones reported in literature.[15]

^{1}H-NMR (600 MHz, DMSO): δ = 10.15 (s, 2H), 7.99 (d, J = 8.2 Hz, 1H), 7.40 (dd, J = 8.2, 2.0 Hz, 1H), 7.03 (d, J = 2.0 Hz, 1H), 6.69 (d, J = 2.3 Hz, 2H), 6.62 – 6.55 (m, 4H) ppm.

^{13}C-NMR (151 MHz, DMSO): δ = 168.3, 160.0, 155.2, 152.3, 147.6, 129.6, 126.9, 123.2, 122.0, 114.7, 113.1, 109.8, 102.7, 83.1 ppm.

5. Scope of the Reaction

General procedure for the reactions carried out during the scope studies

An oven dried 10 mL Schlenk tube was charged with $Pd(OAc)_2$ (4.5 mg, 0.020 mmol, 10 mol%), monodentate ligand **L1b** (4.2 mg, 0.040 mmol, 20 mol%), bidentate ligand **L2b** (13.8 mg, 0.040 mmol, 20 mol%), AgOAc (66.8 mg, 0.400 mmol, 2 equiv), starting material **1** (0.200 mmol), 3-(triisopropylsilyl)prop-2-yn-1-yl acrylate **2** (159.8 mg, 0.6000 mmol, 3 equiv), and the solvent mixture TFE:$CHCl_3$ 2:8 (1.2 mL). The reaction vessel was tightly sealed and placed into a preheated aluminum block at 70°C with a tightly fitting recess on a magnetic stirrer set at 1000 rpm. The reaction mixture was stirred under these conditions for 48 hours, was allowed to cool to room temperature, filtered over Celite, and concentrated under reduced pressure. The crude product was purified by silica gel column chromatography.

Compounds **3g-i** were synthesized following the same procedure, but using $Pd(OAc)_2$ (6.9 mg, 0.030 mmol, 15 mol%), monodentate ligand **L1b** (6.3 mg, 0.060 mmol, 30 mol%), bidentate ligand **L2b** (20.7 mg, 0.060 mmol, 30 mol%), and conducting the reaction at 80°C.

The acrylate proton of each different product was used to calculate the regioisomeric ratios.

(*E*)-3-(triisopropylsilyl)prop-2-yn-1-yl 3-(3,4-dimethylphenyl)acrylate (3a-*α*), (*E*)-3-(triisopropylsilyl)prop-2-yn-1-yl 3-(2,3-dimethylphenyl)acrylate (3a-*β*)

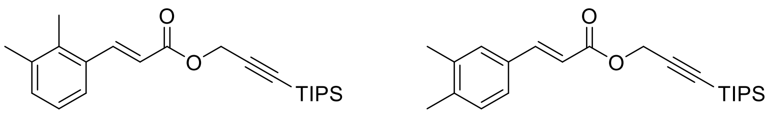

3a-*α*　**3a-*β***

According to the general procedure, compound **3a** was synthesized using *o*-xylene **1a** (21.2 mg, 0.200 mmol). The target compound was purified by column chromatography (*n*-pentane:CH_2Cl_2 9:1) and obtained as a transparent oil (39.7 mg, 0.107 mmol, 54%, α:*β* = 3:97).

The large scale reaction was performed in a 50 mL Schlenk tube using $Pd(OAc)_2$ (69.0 mg, 0.300 mmol, 10 mol%), monodentate ligand **L1b** (63.0 mg, 0.600 mmol, 20 mol%), bidentate ligand **L2b** (207 mg, 0.600 mmol, 20 mol%), AgOAc (1.002 g, 6.000 mmol, 2 equiv), *o*-xylene **1a** (318.5 mg, 3.000 mmol), 3-(triisopropylsilyl)prop-2-yn-1-yl acrylate **2** (2.397 g, 9.000 mmol, 3 equiv), and the solvent mixture TFE:$CHCl_3$ 2:8 (18 mL). The reaction mixture was stirred at the 75°C for 48 hours. The target compound was purified by column chromatography (*n*-pentane:CH_2Cl_2 9:1) and obtained as a transparent oil (621.5 mg, 1.677 mmol, 56%, α:*β* = 3:97).

^{1}H-NMR (500 MHz, $CDCl_3$): δ = 8.12 (d, *J* = 15.8 Hz, 1H$^{\alpha}$), 7.68 (d, *J* = 16.0 Hz, 1H$^{\beta}$), 7.40 (d, *J* = 7.8 Hz, 1H$^{\alpha}$), 7.31 (d, *J* = 1.9 Hz, 1H$^{\beta}$), 7.28 (dd, *J* = 7.7, 1.9 Hz, 1H$^{\beta}$), 7.19 (d, *J* = 7.4 Hz, 1H$^{\alpha}$), 7.15 (d, *J* = 7.7 Hz, 1H$^{\beta}$), 7.12 (m, 1H$^{\alpha}$), 6.43 (d, *J* = 16.0 Hz, 1H$^{\beta}$), 6.35 (d, *J* = 15.8 Hz, 1H$^{\alpha}$), 4.85 (s, 2H$^{\alpha}$), 4.84 (s, 2H$^{\beta}$), 2.33 (s, 3H$^{\alpha}$), 2.31 (s, 3H$^{\alpha}$), 2.28 (s, 6H$^{\beta}$), 1.09 (d, *J* = 1.9 Hz, 21H$^{\alpha+\beta}$) ppm.
^{13}C-NMR (126 MHz, $CDCl_3$): δ = 166.5$^{\beta}$, 145.9$^{\beta}$, 144.4$^{\alpha}$, 139.8$^{\beta}$, 137.3$^{\beta}$, 136.3$^{\alpha}$, 133.7$^{\alpha}$ 132.1$^{\beta}$, 131.8$^{\alpha}$, 130.3$^{\beta}$, 129.5$^{\beta}$, 126.0$^{\beta}$, 124.6$^{\alpha}$, 118.9$^{\alpha}$, 116.2$^{\beta}$, 101.3$^{\beta}$, 88.4$^{\beta}$, 53.0$^{\beta}$, 20.0$^{\beta}$, 19.9$^{\beta}$, 18.7$^{\alpha+\beta}$, 11.3$^{\alpha+\beta}$ ppm.
HRMS (ESI+H$^+$) m/z: Calculated for $C_{23}H_{34}O_2Si$ 371.23879, Found 371.23907.

(*E*)-3-(triisopropylsilyl)prop-2-yn-1-yl 3-(*o*-tolyl)acrylate (3b-*ortho*)
(*E*)-3-(triisopropylsilyl)prop-2-yn-1-yl 3-(*m*-tolyl)acrylate (3b-*meta*)
(*E*)-3-(triisopropylsilyl)prop-2-yn-1-yl 3-(*p*-tolyl)acrylate (3b-*para*)

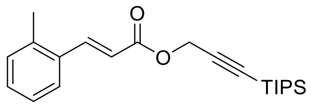

3b-*ortho*

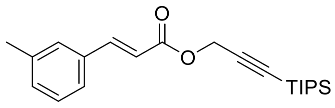

3b-*meta*

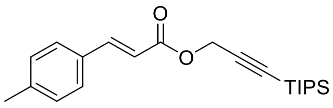

3b-*para*

According to the general procedure, compound **3b** was synthesized using toluene **1b** (18.4 mg, 0.200 mmol). The target compound was purified by column chromatography (*n*-pentane:CH_2Cl_2 9:1) and obtained as a transparent oil (40.0 mg, 0.112 mmol, 56%, *o:m:p* = 4:55:41).

^{1}H-NMR (600 MHz, $CDCl_3$): δ = 8.02 (d, *J* = 15.9 Hz, 1H^{o}), 7.70 (d, *J* = 16.0 Hz, 1H^{m+p}), 7.57 (d, *J* = 7.7 Hz, 1H^{o}), 7.43 (d, *J* = 8.4 Hz, 2H^{p}), 7.37 – 7.32 (m, 2H^{m}), 7.30 – 7.26

(m, 1H^{o+m}), 7.23 – 7.18 (m, 1H^{o+m}+2H^{p}), 6.46 (d, *J* = 16.0 Hz, 1H^{m}), 6.43 (d, *J* = 16.0 Hz, 1H^{p}), 6.40 (d, *J* = 15.9 Hz, 1H^{o}), 4.84 (m, 2H^{o+m+p}), 2.44 (s, 3H^{o}), 2.38 (m, 3H^{m+p}), 1.09 (m, 21H^{o+m+p}) ppm.

^{13}C-NMR (151 MHz, CDCl$_3$): δ = 166.5^{p}, 166.3^{m}, 145.9^{m}, 145.7^{p}, 143.3^{o}, 141.0^{p}, 138.7^{m}, 137.9^{o}, 134.4^{m}, 133.4^{o}, 131.7^{p}, 131.4^{m}, 131.0^{o}, 130.3^{o}, 129.8^{p}, 128.9^{m}, 128.3^{p}, 126.6^{o}, 126.5^{o}, 125.5^{m}, 118.5^{o}, 117.3^{m}, 116.4^{p}, 101.2^{p}, 101.2^{m}, 88.5^{m}, 88.5^{p}, 53.1^{m}, 53.0^{p}, 21.6^{p}, 21.5^{m}, 18.7^{o+m+p}, 11.3^{o+m+p} ppm.

HRMS (ESI+H$^+$) m/z: Calculated for $C_{22}H_{33}O_2Si$ 357.22443, Found 357.22392

(*E*)-3-(triisopropylsilyl)prop-2-yn-1-yl 3-(2-(2-hydroxyethyl)phenyl)acrylate (3c-*ortho*)

(*E*)-3-(triisopropylsilyl)prop-2-yn-1-yl 3-(3-(2-hydroxyethyl)phenyl)acrylate (3c-*meta*)

(*E*)-3-(triisopropylsilyl)prop-2-yn-1-yl 3-(4-(2-hydroxyethyl)phenyl)acrylate (3c-*para*)

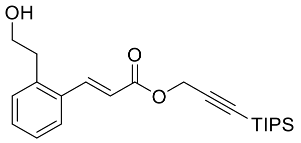

3c-*ortho*

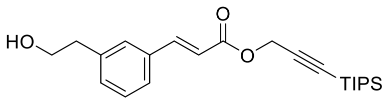

3c-*meta*

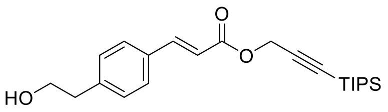

3c-*para*

According to the general procedure, compound **3c** was synthesized using 2-phenylethanol **1c** (24.4 mg, 0.200 mmol). The target compound was purified by column chromatography (Cy:EtOAc 8:2) and the starting material was separated by Kugelrohr distillation (85°C, 10 mbar) and obtained as a yellow oil (30.7 mg, 79.4 µmol, 40%, o:*m*:*p* = 4:53:43).

^{1}H-NMR (600 MHz, CDCl$_3$): δ = 8.06 (d, *J* = 15.8 Hz, 1H^{o}), 7.71 (m, 1H^{m+p}), 7.61 (d, *J* = 8.0 Hz, 1H^{o}), 7.49 (d, *J* = 7.9 Hz, 2H^{p}), 7.41 (m, 2H^{m}), 7.34 (m, 1H^{m+o}), 7.28 – 7.25 (m, 1H^{m}+2H^{o+p}), 6.46 (m, 1H^{o+m+p}), 4.84 (m, 2H^{o+m+p}), 3.88 (m, 2H^{o+m+p}), 2.89 (m, 2H^{o+m+p}), 1.10 – 1.08 (m, 21H^{o+m+p}) ppm.

^{13}C-NMR (151 MHz, CDCl$_3$): δ = 166.4^{p}, 166.3^{o+m}, 145.6^{m}, 145.4^{p}, 142.9^{o}, 141.6^{p}, 139.5^{m}, 134.7^{m}, 132.8^{p}, 131.3^{m}, 131.1^{o}, 130.5^{o}, 129.8^{p}, 129.3^{m}, 128.9^{m}, 128.6^{p}, 127.3^{o},

127.0^{o}, 126.5^{m}, 119.2^{o}, 117.6^{m}, 117.0^{p}, 101.2^{p}, 101.1^{o+m}, 88.6^{o+m+p}, 63.7^{o}, 63.6^{m}, 63.5^{p}, 53.1^{o+m}, 53.1^{p}, 39.2^{p}, 39.1^{m}, 36.5^{o}, 18.7^{o+m+p}, 11.2^{o+m+p} ppm.

HRMS (ESI+H^{+}) m/z: Calculated for $C_{23}H_{35}O_{3}Si$ 387.23500, Found 387.23408.

(*E*)-3-(triisopropylsilyl)prop-2-yn-1-yl 3-(3-(2-((*tert*-butoxycarbonyl)(methyl)amino)ethyl)phenyl)acrylate (3d-*meta*)
(*E*)-3-(triisopropylsilyl)prop-2-yn-1-yl 3-(4-(2-((*tert*-butoxycarbonyl)(methyl)amino)ethyl)phenyl)acrylate (3d-*para*)

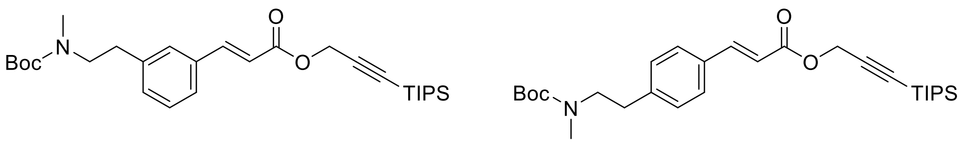

3d-*meta* **3d-*para***

According to the general procedure, compound **3d** was synthesized using tert-butyl methyl(phenethyl)carbamate **1d** (47.1 mg, 0.200 mmol). The target compound was purified by column chromatography (*n*-pentane:EtOAc 98:2 →95:5) and obtained as a yellow oil (40.5 mg, 81.0 µmol, 41%, *m:p* = 57:43).

^{1}H-NMR (600 MHz, CDCl$_3$): δ = 7.70 (d, *J* = 16.0 Hz, 1H^{m+p}), 7.46 (d, *J* = 8.0 Hz, 2H^{p}), 7.41 – 7.28 (m, 3H^{m}), 7.21 – 7.16 (m, 1H^{m} + 2H^{p}), 6.46 (d, *J* = 16 Hz, 1H^{m}), 6,43 (d, *J* = 16 Hz, 1H^{p}), 4.84 (s, 2H^{m}), 4.84 (s, 2H^{p}), 3.43 (bs, 2H^{m+p}), 2.89 – 2.74 (m, 5H^{m+p}), 1.55 – 1.30 (m, 9H^{m+p}), 1.17 – 1.01 (m, 21H^{m+p}) ppm.

^{13}C-NMR (151 MHz, CDCl$_3$): δ = 166.3^{m+p}, 166.2^{m+p}, 155.7^{m+p}, 145.6^{m+p}, 145.4^{m+p}, 142.3^{p}, 140.2^{m}, 134.6^{m}, 132.6^{p}, 131.2^{m}, 129.6^{p}, 129.2^{m}, 128.7^{m}, 128.5^{p}, 126.3^{m}, 117.5^{m}, 116.9^{p}, 101.2^{m+p}, 101.1^{m+p}, 88.5^{m+p}, 79.5^{m+p}, 53.0^{m+p}, 53.0^{m+p}, 50.8^{m+p}, 50.7 $^{m+p}$, 50.3^{m+p}, 34.9^{m+p}, 34.6^{m+p}, 34.5^{m+p}, 34.4^{m+p}, 34.2^{m+p}, 34.0^{m+p}, 28.6^{m+p}, 18.7^{m+p}, 11.2^{m+p} ppm.

HRMS (ESI+NH$_4^{+}$) m/z: Calculated for $C_{29}H_{45}O_{4}NSi$ 517.34561, Found 517.34454.

(*E*)-3-(triisopropylsilyl)prop-2-yn-1-yl 3-(3,4-dimethoxyphenyl)acrylate (3e-*β*-*E*)
(*Z*)-3-(triisopropylsilyl)prop-2-yn-1-yl 3-(3,4-dimethoxyphenyl)acrylate (3e-*β*-*Z*)

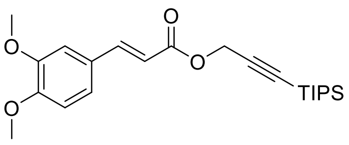
3e-β-*E*

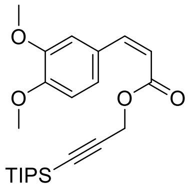
3e-β-*Z*

According to the general procedure, compound **3e** was synthesized using veratrole **1e** (27.6 mg, 0.200 mmol). The target compound was purified by column chromatography (Cy:CH_2Cl_2 6:4) and obtained as a transparent oil (36.9 mg, 91.7 µmol, 46%, *β* only, *E:Z* = 89:11).

^{1}H-NMR (600 MHz, $CDCl_3$): δ = 7.71 (d, *J* = 2.1 Hz, 1H^Z), 7.66 (d, *J* = 15.9 Hz, 1H^E), 7.21 (dd, *J* = 8.3, 2.1 Hz, 1H^Z), 7.10 (dd, *J* = 6.8, 2.0 Hz, 1H^E), 7.05 (d, *J* = 2.0 Hz, 1H^E), 6.87 – 6.81 (m, 1H^E + 2H^Z), 6.34 (d, *J* = 15.9 Hz, 1H^E), 5.85 (d, *J* = 12.9 Hz, 1H^Z), 4.83 (s, 2H^E), 4.77 (s, 2H^Z), 3.90 (m, 6H^{E+Z}), 1.10 – 1.04 (m, 21H^{E+Z}) ppm.
^{13}C-NMR (151 MHz, $CDCl_3$): δ = 166.5^E, 165.5^Z, 151.4^E, 150.4^Z, 149.3^E, 148.4^Z, 145.6^E, 144.8^Z, 127.6^Z, 127.4^E, 125.2^Z, 123.0^E, 116.1^Z, 115.1^E, 113.5^Z, 111.1^E, 110.4^Z, 109.7^E, 101.3^E, 101.2^Z, 88.4^E, 88.3^Z, 56.1^E, 56.0^E, 55.9^Z, 52.9^E, 52.7^Z, 18.7^{E+Z}, 11.2^{E+Z} ppm.
HRMS (ESI+H^+) m/z: Calculated for $C_{23}H_{35}O_4Si$ 403.22991, Found 403.22950.

(*E*)-3-(triisopropylsilyl)prop-2-yn-1-yl 3-(2-fluorophenyl)acrylate (3f-*ortho*)
(*E*)-3-(triisopropylsilyl)prop-2-yn-1-yl 3-(3-fluorophenyl)acrylate (3f-*meta*)
(*E*)-3-(triisopropylsilyl)prop-2-yn-1-yl 3-(4-fluorophenyl)acrylate (3f-*para*)

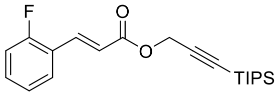
3f-*ortho*

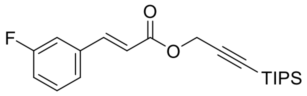
3f-*meta*

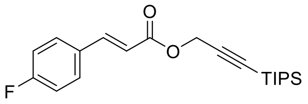
3f-*para*

According to the general procedure, compound **3f** was synthesized using fluorobenzene **1f** (19.2 mg, 0.200 mmol). The target compound was purified by column chromatography (*n*-pentane:CH_2Cl_2 8:2) and obtained as a transparent oil (31.1 mg, 86.3 µmol, 43%, *o:m:p* = 9:77:14).

^{1}H-NMR (600 MHz, CDCl$_3$): δ = 7.86 (d, *J* = 16.2 Hz, 1H^{m}), 7.68 (m, 1H^{o+p}), 7.54 (m, 1H^{m}+2H^{p}), 7.39 – 7.33 (m, 1H^{m+o}), 7.30 (d, *J* = 7.7 Hz, 1H^{o}), 7.23 (d, *J* = 9.9 Hz, 1H^{o}), 7.17 (td, *J* = 7.6, 1.2 Hz, 1H^{m}), 7.13 – 7.06 (m, 1H^{m+o}+2H^{p}), 6.58 (d, *J* = 16.2 Hz, 1H^{m}), 6.46 (d, *J* = 16.0 Hz, 1H^{o}), 6.40 (d, *J* = 16.0 Hz, 1H^{p}), 4.85 (m, *J* = 6.3 Hz, 3H^{m+o+p}), 1.08 (m, 21H^{m+o+p}) ppm.
^{13}C-NMR (151 MHz, CDCl$_3$): δ = 166.1^{m}, 162.4^{p}, 160.7^{o}, 144.3^{p}, 144.2^{o}, 138.3^{m}, 132.0^{m} (d, *J* = 8.7 Hz), 130.6^{o} (d, *J* = 8.2 Hz), 130.2^{p} (d, *J* = 8.2 Hz), 129.3^{m}, 124.6^{m} (d, *J* = 3.8 Hz), 124.3^{o}, 122.5^{m} (d, *J* = 11.4 Hz), 120.1^{m}, 118.9^{o}, 117.5^{o}, 117.4^{p}, 117.3^{p}, 116.5^{m}, 116.3^{m}, 116.2^{p}, 114.6^{o} (d, *J* = 22.3 Hz), 101.1^{m+o}, 100.9^{p}, 88.8^{p}, 88.7^{m+o}, 53.2^{m+o}, 53.1^{p}, 18.7^{m+o+p}, 11.3^{m+o+p} ppm.
^{19}F-NMR (471 MHz, CDCl$_3$): δ = -109.78^{p}, -112.93^{o}, -114.59^{m} ppm.
HRMS (EI) m/z: Calculated for $C_{21}H_{29}FO_2Si$ 360.19208, Found 360.19208.

(*E*)-3-(triisopropylsilyl)prop-2-yn-1-yl 3-(2-pivaloylphenyl)acrylate (3g-*ortho*)
(*E*)-3-(triisopropylsilyl)prop-2-yn-1-yl 3-(3-pivaloylphenyl)acrylate (3g-*meta*)
(*E*)-3-(triisopropylsilyl)prop-2-yn-1-yl 3-(4-pivaloylphenyl)acrylate (3g-*para*)
(*Z*)-3-(triisopropylsilyl)prop-2-yn-1-yl 3-(pivaloylphenyl)acrylate (3g-*Z-isomers*)

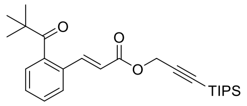

3g-*ortho*

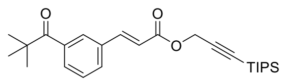

3g-*meta*

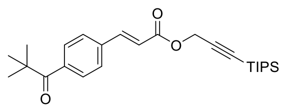

3g-*para*

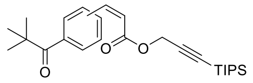

3g-*Z-isomers*

According to the general procedure, compound **3g** was synthesized using pivalophenone **1g** (32.4 mg, 0.200 mmol). The target compound was purified by column chromatography (*n*-pentane:CH_2Cl_2 6:4) and obtained as a yellow oil (32.7 mg, 76.6 µmol, 38%, *o:m:p:Z-isomers* = 12:62:19:7).

^{1}H-NMR (600 MHz, CDCl$_3$): δ = 7.80 (t, *J* = 1.8 Hz, 1H^{m}), 7.76 – 7.65 (m, 2H^{m}+3H^{p}+1H^{o}), 7.62 (m, 1H^{m}+1H^{o}), 7.58 – 7.52 (m, 2H^{p}+1H^{o}), 7.46 – 7.36 (m, 1H^{m}+2H^{o}+H^{Z}), 7.20 (dd, *J* = 6.6, 2.4 Hz, H^{Z}), 6.97 (d, *J* = 12.7 Hz, H^{Z}), 6.51 (m, 1H^{m}+1H^{p}), 6.40 (d, *J* = 15.9 Hz, 1H^{o}), 6.05 (d, *J* = 12.7 Hz, H^{Z}), 4.85 (s, 2H^{m}+2H^{p}), 4.81 (s, 2H^{o}), 4.76 (m, H^{Z}), 1.35 (m, 9H^{m}+9H^{p}+9H^{o}), 1.23 (s, H^{Z}), 1.10 – 1.06 (m, 21H$^{m+p+o+Z}$) ppm.

^{13}C-NMR (151 MHz, CDCl$_3$): δ = 214.1^{Z}, 208.8^{m+p}, 208.6^{o}, 166.0^{m}, 165.9^{p}, 165.5^{o}, 165.0^{Z}, 144.8^{m}, 144.3^{p}, 143.2^{Z}, 142.6^{o}, 142.3^{Z}, 140.1^{p}, 139.5^{m}, 136.6^{p}, 134.4^{m}, 131.1^{Z}, 130.2^{m}, 129.6^{o}, 129.6^{m}, 129.5^{o}, 129.2^{o}, 128.8^{m}, 128.6^{p}, 127.89^{p}, 127.85^{o}, 127.7^{m}, 126.8^{Z}, 125.5^{Z}, 120.2^{Z}, 119.8^{o}, 119.2^{m}, 118.6^{m}, 101.0^{m+o+p}, 100.9^{Z}, 88.8^{o}, 88.7^{m+p}, 88.6^{Z}, 53.2^{m+o+p}, 53.0^{Z}, 45.4^{Z}, 44.42^{m}, 44.38^{p}, 44.3^{o}, 28.1^{m+o+p}, 27.4^{Z}, 18.7$^{m+o+p+Z}$, 11.2$^{m+o+p+Z}$ ppm.

HRMS (ESI+H$^+$) m/z: Calculated for $C_{26}H_{39}O_3Si$ 427.26630, Found 427.26562.

(*E*)-ethyl 2-(3-oxo-3-((3-(triisopropylsilyl)prop-2-yn-1-yl)oxy)prop-1-en-1-yl)benzoate (3h-*ortho*)

(*E*)-ethyl 3-(3-oxo-3-((3-(triisopropylsilyl)prop-2-yn-1-yl)oxy)prop-1-en-1-yl)benzoate (3h-*meta*)

(*E*)-ethyl 4-(3-oxo-3-((3-(triisopropylsilyl)prop-2-yn-1-yl)oxy)prop-1-en-1-yl)benzoate (3h-*para*)

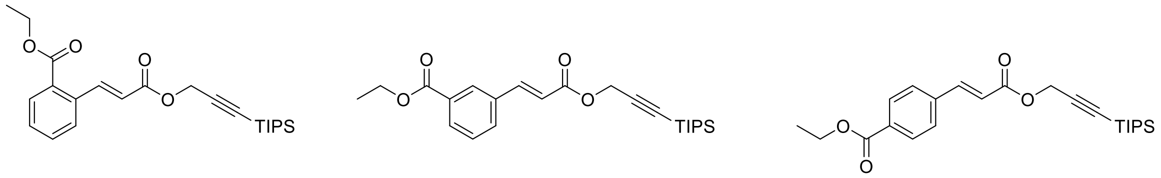

3h-*ortho* **3h-*meta*** **3h-*para***

According to the general procedure, compound **3h** was synthesized using ethyl benzoate **1h** (30.0 mg, 0.200 mmol). The target compound was purified by column chromatography (*n*-pentane:CH_2Cl_2 6:4) and obtained as a transparent oil (24.5 mg, 59.1 µmol, 30%, *o:m:p* = 9:76:15).

3

^{1}H-NMR (600 MHz, CDCl$_3$): δ = 8.47 (d, *J* = 15.9 Hz, 1H^{o}), 8.21 (t, *J* = 1.8 Hz, 1H^{m}), 8.06 (m, 1H^{m}+2H^{p}), 7.96 (dd, *J* = 7.8, 1.4, 1H^{o}), 7.74 (m, 1H^{m+p}), 7.70 (dt, *J* = 7.7, 1.2 Hz, 1H^{m}), 7.61 – 7.57 (m, 1H^{o}+2H^{p}), 7.52 (dt, *J* = 7.4, 1.4 1H^{o}), 7.47 (t, *J* = 7.8 Hz, 1H^{m}), 7.46 – 7.43 (m, 1H^{o}), 6.55 (m, 1H^{m+p}), 6.33 (d, *J* = 15.9 Hz, 1H^{o}), 4.85 (m, *J* = 1.7 Hz, 2H^{o+m+p}), 4.43 – 4.36 (m, 2H^{o+m+p}), 1.41 (m, 3H^{o+m+p}), 1.08 (m, 21H^{o+m+p}) ppm.

^{13}C-NMR (151 MHz, CDCl$_3$): δ = 167.0^{o}, 166.1^{m}, 165.9^{m}, 165.8^{p}, 145.0^{o}, 144.5^{m}, 144.3^{p}, 138.5^{m}, 136.1^{o}, 134.7^{m}, 134.5^{p}, 132.3^{m}, 132.1^{o}, 131.4^{m}, 130.9^{o}, 130.2^{p}, 129.6^{o}, 129.18^{m}, 129.15^{m}, 128.1^{p}, 128.0^{o}, 120.1^{o}, 119.8^{p}, 118.8^{m}, 101.1^{o}, 101.0^{m}, 100.9^{p}, 88.82^{p}, 88.75^{m}, 88.6^{o}, 61.6^{o}, 61.4^{m}, 61.4^{p}, 53.3^{p}, 53.2^{m}, 53.2^{p}, 18.7^{o+m+p}, 14.5^{o+m+p}, 11.2^{o+m+p} ppm.

HRMS (ESI+H$^+$) m/z: Calculated for $C_{24}H_{35}O_4Si$ 415.22991, Found 415.22906.

(*E*)-3-(triisopropylsilyl)prop-2-yn-1-yl 3-(2-(piperidine-1-carbonyl)phenyl)acrylate (3i-*ortho*)
(*E*)-3-(triisopropylsilyl)prop-2-yn-1-yl 3-(3-(piperidine-1-carbonyl)phenyl)acrylate (3i-*meta*)
(*E*)-3-(triisopropylsilyl)prop-2-yn-1-yl 3-(4-(piperidine-1-carbonyl)phenyl)acrylate (3i-*para*)

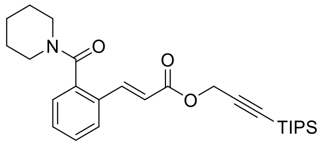

3i-*ortho*

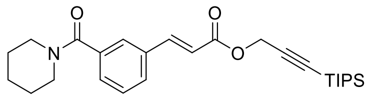

3i-*meta*

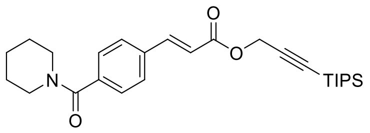

3i-*para*

According to the general procedure, compound **3i** was synthesized using benzoylpiperidine **1i** (37.9 mg, 0.200 mmol). The target compound was purified by column chromatography (Cy:EtOAc 7:3) and obtained as a transparent oil (39.1 mg, 86.2 µmol, 43%, *o:m:p* = 33:47:20).

^{1}H-NMR (600 MHz, $CDCl_3$): δ = 7.75 – 7.68 (m, $1H^{o+m+p}$), 7.66 – 7.64 (m, $1H^{o}$), 7.57 – 7.53 (m, $2H^{m+p}$), 7.44 – 7.37 (m, $2H^{o+m+p}$), 7.29 – 7.26 (m, $1H^{o}$), 6.53 – 6.41 (m, $1H^{o+m+p}$), 4.84 (m, $2H^{o+m+p}$) 3.71 (br, $2H^{o+m+p}$), 3.22 (br, $2H^{o+m+p}$), 1.68 (br, $4H^{o+m+p}$), 1.51 (br, $2H^{o+m+p}$), 1.07 (m, $21H^{o+m+p}$) ppm.
^{13}C-NMR (151 MHz, $CDCl_3$): δ = 169.6^{m+p}, 168.5^{o}, 166.0^{p+m}, 165.7^{o}, 144.7^{m}, 144.6^{p}, 142.1^{o}, 138.3^{p}, 137.8^{o}, 137.4^{m}, 135.4^{p}, 134.7^{o}, 130.9^{o}, 130.6^{m}, 129.3^{p}, 129.2^{m}, 129.1^{m}, 128.7^{m}, 128.3^{p}, 127.6^{o}, 126.9^{o}, 126.8^{o}, 126.5^{m}, 119.7^{o}, 118.6^{p}, 118.6^{m}, 101.0^{o+m+p}, 88.7^{m+p}, 88.6^{o}, 53.21^{m+p}, 53.16^{o}, $48.9^{\mathrm{m+p}}$, 48.2^{o}, $43.3^{\mathrm{m+p}}$, 42.9^{o}, $26.7^{\mathrm{m+p}}$, 26.4^{o}, 25.8^{o}, $25.7^{\mathrm{m+p}}$, $24.7^{\mathrm{m+p}}$, 24.6^{o}, 18.7^{o+m+p}, 11.2^{o+m+p} ppm.
HRMS (ESI+H^{+}) m/z: Calculated for $C_{27}H_{40}NO_3Si$ 454.27720, Found 454.27685.

(*E*)-methyl 5-(3-oxo-3-((3-(triisopropylsilyl)prop-2-yn-1-yl)oxy)prop-1-en-1-yl)furan-3-carboxylate (3j-*α*)

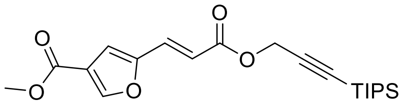

3j-*α*

According to the general procedure, compound **3j** was synthesized using methyl furan-3-carboxylate **1j** (25.0 mg, 0.200 mmol). The target compound was purified by column chromatography (*n*-pentane:EtOAc 98:2 → 95:5) and obtained as a yellow solid (37.9 mg, 97.0 µmol, 49%, only *α*).

^{1}H-NMR (600 MHz, $CDCl_3$): δ = 8.02 (s, 1H), 7.42 (d, *J* = 15.8, 1H), 6.92 (s, 1H), 6.40 (d, *J* = 15.8 Hz, 1H), 4.82 (s, 2H), 3.85 (s, 3H), 1.12 – 1.03 (m, 21H) ppm.
^{13}C-NMR (151 MHz, $CDCl_3$): δ = 165.6, 162.7, 151.7, 149.0, 130.9, 121.4, 117.2, 113.7, 100.8, 88.6, 53.1, 51.8, 18.5, 11.1 ppm.
HRMS (ESI+H^+) m/z: Calculated for $C_{21}H_{31}O_2Si$ 391.19353, Found 391.19240.

(*E*)-3-(triisopropylsilyl)prop-2-yn-1-yl 3-(benzofuran-2-yl)acrylate (3k-*α*)

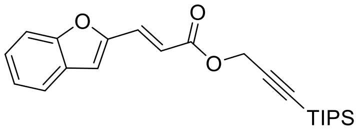

3k-*α*

According to the general procedure, compound **3k** was synthesized using benzofurane **1k** (23.5 mg, 0.200 mmol). The target compound was purified by column chromatography (*n*-pentane:methyl *tert*-butyl ether 99:1 → 90:10) followed by Kugelrohr distillation (4 mbar, 160°C to remove acrylate remaining in the sample), and obtained as a yellow solid (25.2 mg, 65.9 µmol, 33%, only *α*).

^{1}H-NMR (600 MHz, $CDCl_3$): δ = 7.60 – 7.58 (m, 2H), 7.48 (dq, *J* = 8.3, 0.9 Hz, 1H), 7.37 (ddd, *J* = 8.4, 7.2, 1.3 Hz, 1H), 7.28 – 7.22 (m, 1H), 6.96 (bs, 1H), 6.61 (dd, *J* = 15.7, 0.6 Hz, 1H), 4.86 (s, 2H), 1.14 – 1.03 (m, 21H) ppm.
^{13}C-NMR (151 MHz, $CDCl_3$): δ = 166.0, 155.8, 152.4, 132.3, 128.5, 126.7, 123.5, 122.0, 118.2, 112.0, 111.6, 101.0, 88.7, 53.3, 18.7, 11.3 ppm.
HRMS (ESI+NH_4^+) m/z: Calculated for $C_{23}H_{34}O_3NSi$ 400.23025, Found 400.22922.

(*E*)-methyl 5-(3-oxo-3-((3-(triisopropylsilyl)prop-2-yn-1-yl)oxy)prop-1-en-1-yl)thiophene-3-carboxylate (3l-*α*-*E*)
(*Z*)-methyl 5-(3-oxo-3-((3-(triisopropylsilyl)prop-2-yn-1-yl)oxy)prop-1-en-1-yl)thiophene-3-carboxylate (3l-*α*-*Z*)
(*E*)-methyl 2-(3-oxo-3-((3-(triisopropylsilyl)prop-2-yn-1-yl)oxy)prop-1-en-1-yl)thiophene-3-carboxylate (3l-*α´*-*E*)

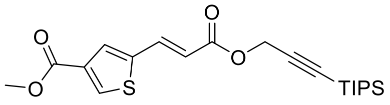

3l-α–***E***

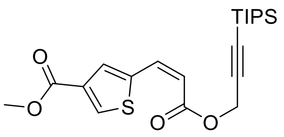

3l-α–***Z***

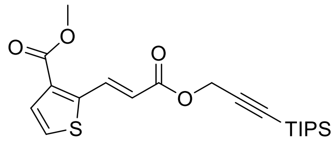

3l-α´-***E***

According to the general procedure, compound **3l** was synthesized using methyl thiophene-3-carboxylate **1l** (28.6 mg, 0.200 mmol). The target compound was purified by column chromatography (*n*-pentane:Et_2O 97:3 → 92:8) and obtained as a colorless solid (37.1 mg, 91.2 µmol, 46%, *α-E:α-Z:α´-E* = 33:3:64). The product was isolated in two different fractions, the first one containing 12.0 mg of product (*α-E:α-Z* = 91:9) and the second one containing 25.1 mg of product (*α´-E:α-E* = 94:6). Therefore, two different spectra are given.

Fraction 1 (*α-E:α-Z* = 91:9)

^{1}H-NMR (600 MHz, $CDCl_3$): δ = 8.64 (dd, *J* = 15.9, 0.8 Hz, $1H^{E\text{-}\alpha}$), 8.12 (dd, *J* = 12.8, 0.6 Hz, $1H^{Z\text{-}\alpha}$), 7.49 (d, *J* = 5.3 Hz, $1H^{Z\text{-}\alpha}$), 7.49 (d, *J* = 5.3 Hz, $1H^{E\text{-}\alpha}$),7.38 (dd, 5.4 Hz, 0.8 Hz, $1H^{Z\text{-}\alpha}$), 7.27 (dd, *J* = 5.3, 0.8 Hz, $1H^{E\text{-}\alpha}$), 6.39 (d, *J* = 15.9 Hz, $1H^{E\text{-}\alpha}$), 6.01 (d, *J* = 12.8 Hz, $1H^{Z\text{-}\alpha}$), 4.84 (s, $2H^{E\text{-}\alpha}$), 4.80 (s, $2H^{Z\text{-}\alpha}$), 3.90 (s, $3H^{E\text{-}\alpha}$), 3.88 (s, $3H^{Z\text{-}\alpha}$), 1.12 – 1.01 (m, $21H^{E+Z\text{-}\alpha}$) ppm.

^{13}C-NMR (151 MHz, $CDCl_3$): δ = $165.5^{E+Z\text{-}\alpha}$, $163.3^{E+Z\text{-}\alpha}$, $144.9^{E+Z\text{-}\alpha}$, $136.8^{E\text{-}\alpha}$, $134.7^{E\text{-}\alpha}$, $132.4^{E\text{-}\alpha}$, $130.8^{E\text{-}\alpha}$, $129.1^{Z\text{-}\alpha}$, $128.5^{Z\text{-}\alpha}$, $126.3^{E\text{-}\alpha}$, $120.2^{E\text{-}\alpha}$, $117.6^{Z\text{-}\alpha}$, $100.9^{E+Z\text{-}\alpha}$, $88.7^{E+Z\text{-}\alpha}$, $53.3^{E\text{-}\alpha}$, $53.0^{Z\text{-}\alpha}$, $52.2^{E+Z\text{-}\alpha}$, $18.7^{E+Z\text{-}\alpha}$, $11.2^{E+Z\text{-}\alpha}$ ppm.

Fraction 2 (*α´-E*:*α-E* = 94:6)

^{1}H-NMR (500 MHz, $CDCl_3$): δ = 8.33 (dd, *J* = 16.1, 0.8 Hz, 1H$^{\alpha}$), 8.15 (d, *J* = 3.4 Hz, 1H$^{\alpha}$), 8.10 (t, *J* = 0.9 Hz, 1H$^{\alpha'}$), 7.76 (dt, *J* = 15.8, 0.8 Hz, 1H$^{\alpha'}$), 7.65 (dt, 1.2, 0.6 Hz, 1H$^{\alpha'}$), 7.59 (dd, *J* = 3.4, 0.8 Hz, 1H$^{\alpha}$), 6.35 (d, *J* = 16.1 Hz, 1H$^{\alpha}$), 6.29 (d, *J* = 15.7 Hz, 1H$^{\alpha'}$), 4.83 (s, 2H$^{\alpha'+\alpha}$), 3.88 (s, 3H$^{\alpha'+\alpha}$), 1.08 (s, 21H$^{\alpha'+\alpha}$) ppm.

^{13}C-NMR (126 MHz, $CDCl_3$): δ = 165.5$^{\alpha'+\alpha}$, 162.5$^{\alpha'+\alpha}$, 139.8$^{\alpha'}$, 138.6$^{\alpha}$, 137.0$^{\alpha'+\alpha}$, 134.8$^{\alpha}$, 134.3$^{\alpha}$, 131.0$^{\alpha'+\alpha}$, 125.3$^{\alpha}$, 119.6$^{\alpha}$, 117.7$^{\alpha}$, 100.7$^{\alpha'+\alpha}$, 88.7$^{\alpha'+\alpha}$, 53.1$^{\alpha'+\alpha}$, 52.0$^{\alpha'+\alpha}$, 18.5$^{\alpha'+\alpha}$, 11.1$^{\alpha'+\alpha}$ ppm.

HRMS (ESI+NH_4^+) m/z: Calculated for $C_{21}H_{34}O_4N_1S_1Si_1$ 424.19723, Found 424.19650.

(*E*)-methyl 1-methyl-2-(3-oxo-3-((3-(triisopropylsilyl)prop-2-yn-1-yl)oxy)prop-1-en-1-yl)-*1H*-pyrrole-3-carboxylate (3m-*α-E*)

(*Z*)-methyl 1-methyl-2-(3-oxo-3-((3-(triisopropylsilyl)prop-2-yn-1-yl)oxy)prop-1-en-1-yl)-*1H*-pyrrole-3-carboxylate (3m-*α-Z*)

(*E*)-methyl 1-methyl-5-(3-oxo-3-((3-(triisopropylsilyl)prop-2-yn-1-yl)oxy)prop-1-en-1-yl)-*1H*-pyrrole-3-carboxylate (3m-*α´-E*)

(*Z*)-methyl 1-methyl-5-(3-oxo-3-(((triisopropylsilyl)ethynyl)oxy)prop-1-en-1-yl)-*1H*-pyrrole-3-carboxylate (3m-*α´-Z*)

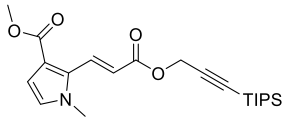

3m-α-*E*

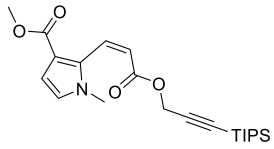

3m-α-*Z*

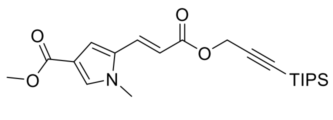

3m-α´-*E*

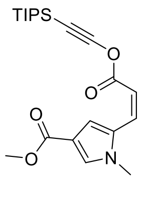

3m-α´-*Z*

According to the general procedure, compound **3m** was synthesized using methyl 1-methyl-1*H*-pyrrole-3-carboxylate **1m** (27.8 mg, 0.200 mmol). The target compound was purified by column chromatography (*n*-pentane:EtOAc:Et_3N 8:2:0.2) and obtained as a yellow oil (25.7 mg, 91.2 µmol, 32%, *α-E:α-Z:α´-E:α´-Z* = 54:11:31:4). The product was isolated in two different fractions, the first one containing 16.8 mg of product (*α-E:α-Z* = 83:17) and the second one containing 8.9 mg of product (*α´-E:α´-Z* = 90:10). Therefore, two different spectra are given.

Fraction 1 (*α-E:α-Z* = 83:17)

^{1}H-NMR (600 MHz, $CDCl_3$): δ = 8.22 (d, *J* = 16.5 Hz, 1H$^{E-\alpha}$), 7.20 (d, *J* = 12.0 Hz, 1H$^{Z-\alpha}$), 6.69 (d, *J* = 2.9 Hz, 1H$^{E-\alpha}$), 6.66 (d, *J* = 2.9 Hz, 1H$^{E-\alpha}$), 6.64 (d, *J* = 2.9 Hz, 1H$^{Z-\alpha}$), 6.60 (d, *J* = 2.9 Hz, 1H$^{Z-\alpha}$), 6.55 (d, *J* = 16.5 Hz, 1H$^{E-\alpha}$), 6.21 (d, *J* = 12.0 Hz, 1H$^{Z-\alpha}$), 4.84 (s, 2H$^{E-\alpha}$), 4.70 (s, 1H$^{Z-\alpha}$), 3.83 (s, 3H$^{E-\alpha}$), 3.77 (s, 3H$^{E-\alpha}$), 3.76 (s, 3H$^{Z-\alpha}$), 3.50 (s, 3H$^{Z-\alpha}$), 1.11 – 1.02 (m, 21H$^{E+Z-\alpha}$) ppm.
^{13}C-NMR (151 MHz, $CDCl_3$): δ = 166.5$^{E+Z-\alpha}$, 164.7$^{E+Z-\alpha}$, 134.8$^{Z-\alpha}$, 132.7$^{E-\alpha}$, 131.2$^{Z-\alpha}$, 126.76$^{Z-\alpha}$, 124.0$^{Z-\alpha}$, 122.5$^{Z-\alpha}$, 118.5$^{Z-\alpha}$, 117.9$^{E-\alpha}$, 112.1$^{E-\alpha}$, 110.3$^{Z-\alpha}$, 101.1$^{E+Z-\alpha}$, 88.3$^{E+Z-\alpha}$, 53.0$^{E-\alpha}$, 52.8$^{Z-\alpha}$, 51.3$^{E-\alpha}$, 51.0$^{Z-\alpha}$, 36.8$^{E-\alpha}$, 34.8$^{Z-\alpha}$, 18.5$^{E+Z-\alpha}$, 11.1$^{E+Z-\alpha}$ ppm.

Fraction 2 (*α´-E:α´-Z* = 90:10)
^{1}H-NMR (600 MHz, $CDCl_3$): δ = 8.16 (d, *J* = 16.1 Hz, 1H$^{E-\alpha'}$), 8.10 (d, *J* = 2.4 Hz, 1H$^{Z-\alpha'}$), 7.79 (d, *J* = 13.6 Hz, 1H$^{Z\ \alpha'}$), 7.26 (s, 1H$^{E-\alpha'}$), 7.23 (d, *J* = 2.4 Hz, 1H$^{Z-\alpha'}$), 6.95 (d, *J* = 2.4 Hz, 1H$^{E-\alpha'}$), 6.31 (d, *J* = 16.1 Hz, 1H$^{E-\alpha'}$), 5.80 (d, *J* = 12.9 Hz, 1H$^{Z-\alpha'}$), 4.81 (s, 2H$^{E-\alpha'}$), 4.76 (s, 1H$^{Z-\alpha'}$), 3.82 (s, 3H$^{E-\alpha'}$), 3.80 (s, 3H$^{Z-\alpha'}$), 3.69 (s, 3H$^{Z-\alpha'}$), 3.68 (s, 3H$^{E-\alpha'}$), 1.10 – 1.05 (m, 21H$^{E+Z-\alpha'}$) ppm.
^{13}C-NMR (151 MHz, $CDCl_3$): δ = 166.6$^{E+Z-\alpha'}$, 164.7$^{E+Z-\alpha'}$, 138.4$^{E-\alpha'}$, 137.1$^{Z-\alpha'}$, 129.4$^{E-\alpha'}$, 129.0$^{Z-\alpha'}$, 128.1$^{Z-\alpha'}$, 123.2$^{E-\alpha'}$, 120.9 $^{E-\alpha}$, 115.3$^{E-\alpha'}$, 114.7$^{Z-\alpha'}$, 112.9$^{Z-\alpha'}$, 101.4$^{E+Z-\alpha'}$, 87.9$^{E+Z-\alpha'}$, 52.6$^{E-\alpha'}$, 52.3 $^{Z-\alpha'}$, 51.1$^{E-\alpha'}$, 51.0$^{Z-\alpha'}$, 36.9$^{E+Z\ \alpha'}$, 18.5$^{E+Z-\alpha'}$, 11.1$^{E+Z\ \alpha'}$ ppm.

HRMS (ESI+NH_4^+) m/z: Calculated for $C_{22}H_{37}O_4N_2Si_1$ 421.25171, Found 421.25150.

(*E*)-3-(triisopropylsilyl)prop-2-yn-1-yl 3-(3-((*R*)-2-(*trans*-4-isopropylcyclohexanecarboxamido)-3-methoxy-3-oxopropyl)phenyl)acrylate (3n-*meta*)

(*E*)-3-(triisopropylsilyl)prop-2-yn-1-yl 3-(4-((*R*)-2-(*trans*-4-isopropylcyclohexanecarboxamido)-3-methoxy-3-oxopropyl)phenyl)acrylate (3n-*para*)

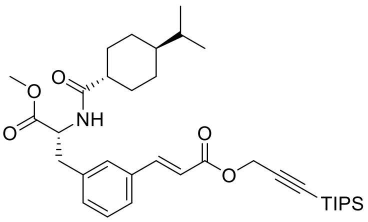

3n-*meta*

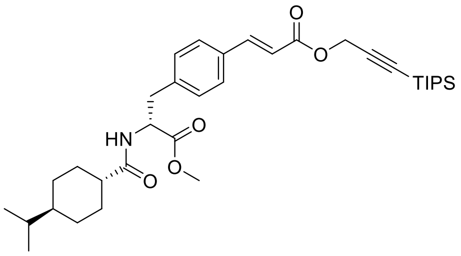

3n-*para*

According to the general procedure, compound **3n** was synthesized using (*R*)-methyl 2-(4-isopropylcyclohexanecarboxamido)-3-phenylpropanoate **1n** (66.2 mg, 0.200 mmol). The target compound was purified by column chromatography (*n*-pentane:EtOAc 90:10) and obtained as a transparent oil (32.6 mg, 54.7 µmol, 27%, *m:p* = 60:40).

^{1}H-NMR (600 MHz, MeOD): δ = 7.72 (d, *J* = 16.0 Hz, 1H^{m}), 7.72 (d, *J* = 16 Hz, 1H^{p}), 7.57 (d, *J* = 8 Hz, 1H^{p}), 7.53 – 7.47 (m, 2H^{m}), 7.36 (ddd, *J* = 7.5, 1.01 Hz, 1H^{m}), 7.31 – 7.26 (m, 1H^{m} + 2H^{p}), 6.58 (d, *J* = 16.1 Hz, 1H^{m}), 6.58 (d, *J* = 16.1 Hz, 1H^{p}), 4.87 – 4.86 (m, 2H^{m+p}), 3.73 (s, 3H^{m}), 3.72 (s, 3H^{p}), 3.29 – 3.18 (m, 1H^{m+p}), 3.04 – 2.94 (m, 1H^{m+p}), 2.15 – 2.06 (m, 1H^{m+p}), 1.84 – 1.71 (m, 3H^{m+p}), 1.70 – 1.61 (m, 1H^{m+p}), 1.47 – 1.18 (m, 3H^{m+p}), 1.16 – 1.04 (m, 21H^{m+p}), 1.05 – 0.95 (m, 3H^{m+p}), 0.88 (d, *J* = 6.8 Hz, 6H^{p}), 0.88 (d, *J* = 6.8 Hz, 6H^{m}) ppm.

^{13}C-NMR (151 MHz, MeOD): δ = 179.1^{m+p}, 179.1^{m+p}, 173.5^{m+p}, 173.4^{m+p}, 167.6^{m+p}, 167.5^{m+p}, 146.8^{m}, 146.6^{p}, 141.4^{p}, 139.4^{m}, 135.7^{m}, 134.3^{p}, 132.6^{m}, 131.0^{p}, 130.2^{m}, 130.1^{m}, 129.4^{p}, 128.0^{p}, 118.4^{m}, 117.9^{p}, 103.1^{m+p}, 103.1^{m+p}, 88.6^{m+p}, 88.6 $^{m+p}$, 54.6^{m+p}, 54.5^{m+p}, 53.6^{m+p}, 53.5^{m+p}, 52.7^{m+p}, 46.2^{m+p}, 46.2^{m+p}, 44.7^{m+p}, 38.2^{m+p}, 38.1^{m+p}, 34.1^{m+p}, 30.9^{m+p}, 30.8^{m+p}, 30.6^{m+p}, 30.5^{m+p}, 30.1^{m+p}, 30.0^{m+p}, 30.0^{m+p}, 20.2^{m+p}, 20.1^{m+p}, 19.0^{m+p}, 12.3^{m+p} ppm.

HRMS (ESI+NH_4^+) m/z: Calculated for $C_{35}H_{57}O_5N_2Si$ 613.40313, Found 613.40155.

(*E*)-3-(triisopropylsilyl)prop-2-yn-1-yl 3-(2,2-dimethyl-7-((triisopropylsilyl)oxy)-2,3-dihydrobenzofuran-4-yl)acrylate (3o-*α*-*E*)
(*Z*)-3-(triisopropylsilyl)prop-2-yn-1-yl 3-(2,2-dimethyl-7-((triisopropylsilyl)oxy)-2,3-dihydrobenzofuran-4-yl)acrylate (3o-*α*-*Z*)
(*E*)-3-(triisopropylsilyl)prop-2-yn-1-yl 3-(2,2-dimethyl-7-((triisopropylsilyl)oxy)-2,3-dihydrobenzofuran-5-yl)acrylate (3o-*β*-*E*)
(*Z*)-3-(triisopropylsilyl)prop-2-yn-1-yl 3-(2,2-dimethyl-7-((triisopropylsilyl)oxy)-2,3-dihydrobenzofuran-5-yl)acrylate (3o-*β*-Z)

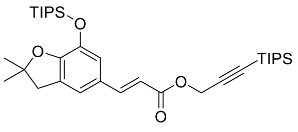

3o-*β*-*E*

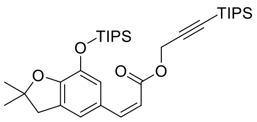

3o-*β*-*Z*

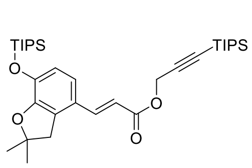

3o-*γ*-*E*

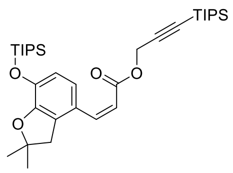

3o-*γ*-*Z*

According to the general procedure, compound **3o** was synthesized using ((2,2-dimethyl-2,3-dihydrobenzofuran-7-yl)oxy)triisopropylsilane **1o** (64.1 mg, 0.200 mmol). The target compound was purified by column chromatography (*n*-pentane/Et_2O 99:1 → 95:5) and obtained as a yellow oil (58.5 mg, 100 µmol, 50%, *β-E:β-Z:γ-E:γ-Z* = 54:11:31:4). The product was isolated in two different fractions, the first one containing 17.3 mg of product (*γ-E:γ-Z* = 89:11) and the second one containing 41.2 mg of product (*β-E:β-Z* = 96:4). Therefore, two different spectra are given.

Fraction 1 (*γ-E:γ-Z* = 89:11)

^{1}H-NMR (600 MHz, $CDCl_3$): δ = 7.62 (d, *J* = 16.2 Hz, 1H$^{E\text{-}\gamma}$), 7.31 (d, *J* = 9.1 Hz, 1H$^{Z\text{-}\gamma}$), 6.94 (d, *J* = 8.6 Hz, 1H$^{E\text{-}\gamma}$), 6.78 (d, *J* = 12.6 Hz, 1H$^{Z\text{-}\gamma}$), 6.73 (d, *J* = 8.4 Hz, 1H$^{E\text{-}\gamma}$), 6.69 (d, *J* = 8.3 Hz, 1H$^{Z\text{-}\gamma}$), 6.23 (d, *J* = 16.1 Hz, 1H$^{E\text{-}\gamma}$), 5.87 (d, *J* = 12.6 Hz, 1H$^{Z\text{-}\gamma}$), 4.83 (s, 2H$^{E\text{-}\gamma}$), 4.75 (s, 1H$^{Z\text{-}\gamma}$), 3.15 (s, 2H$^{E\text{-}\gamma}$), 2.98 (s, 1H$^{Z\text{-}\gamma}$), 1.48 (s, 6H$^{E\text{-}\gamma}$), 1.45 (s, 6H$^{Z\text{-}\gamma}$), 1.34 – 1.22 (m, 4H$^{E+Z\text{-}\gamma}$), 1.14 – 1.01 (m, 38H$^{E+Z\text{-}\gamma}$) ppm.

^{13}C-NMR (151 MHz, $CDCl_3$): δ = 166.7$^{E\text{-}\gamma}$, 165.6$^{Z\text{-}\gamma}$, 149.6$^{E\text{-}\gamma}$, 148.7$^{Z\text{-}\gamma}$, 143.9$^{E\text{-}\gamma}$, 142.7$^{E\text{-}\gamma}$, 141.6$^{Z\text{-}\gamma}$, 141.6$^{Z\text{-}\gamma}$, 129.3$^{Z\text{-}\gamma}$, 128.8$^{E\text{-}\gamma}$, 124.6$^{Z\text{-}\gamma}$, 124.4$^{E\text{-}\gamma}$, 122.0$^{Z\text{-}\gamma}$, 120.9$^{E\text{-}\gamma}$, 120.7$^{E\text{-}\gamma}$, 120.0$^{Z\text{-}\gamma}$, 117.2$^{Z\text{-}\gamma}$, 115.8$^{E\text{-}\gamma}$, 101.3$^{E\text{-}\gamma}$, 101.2$^{Z\text{-}\gamma}$, 88.4$^{E\text{-}\gamma}$, 88.3$^{Z\text{-}\gamma}$, 87.1$^{E\text{-}\gamma}$, 86.7$^{Z\text{-}\gamma}$, 52.93$^{E\text{-}\gamma}$, 52.70$^{Z\text{-}\gamma}$, 43.42$^{E\text{-}\gamma}$, 42.88$^{Z\text{-}\gamma}$, 29.85$^{Z\text{-}\gamma}$, 28.6$^{E\text{-}\gamma}$, 18.7$^{E+Z\text{-}\gamma}$, 18.0$^{E+Z\text{-}\gamma}$, 13.0$^{E+Z\text{-}\gamma}$, 11.3$^{E+Z\text{-}\gamma}$ ppm.

Fraction 2 (*β-E:β-Z* = 96:4)

^{1}H-NMR (500 MHz, $CDCl_3$): δ = 7.59 (d, *J* = 15.9 Hz, 1$H^{E\text{-}\beta}$), 7.49 (s, 1$H^{Z\text{-}\beta}$), 7.09 (s, 1$H^{Z\beta'}$), 6.97 (s, 1$H^{E\text{-}\beta}$), 6.91 (s, 1$H^{E\text{-}\beta}$), 6.74 (d, *J* = 12.9 Hz, 1$H^{Z\text{-}\beta}$), 6.23 (d, *J* = 15.9 Hz, 1$H^{E\text{-}\beta}$), 5.75 (d, *J* = 12.9 Hz, 1$H^{Z\text{-}\beta}$), 4.83 (s, 2$H^{E\text{-}\beta}$), 4.76 (s, 2$H^{Z\text{-}\beta}$), 3,01 (s, 2$H^{E\text{-}\beta}$) 1.47 (s, 6$H^{E+Z\text{-}\beta}$), 1.33 – 1.21 (m, 4$H^{E+Z\text{-}\beta}$), 1.15 – 1.03 (m, 38$H^{E+Z\text{-}\beta}$) ppm.

^{13}C-NMR (151 MHz, $CDCl_3$): δ = $166.8^{E\text{-}\beta}$, $165.6^{Z\text{-}\beta}$, $152.2^{E\text{-}\beta}$, $151.4^{Z\text{-}\beta}$, $146.3^{E\text{-}\beta}$, $145.4^{Z\text{-}\beta}$, $140.6^{E\text{-}\beta}$, $139.6^{Z\text{-}\beta}$, $129.3^{E\text{-}\beta}$, $128.5^{Z\text{-}\beta}$, $127.2^{E\text{-}\beta}$, $124.1^{Z\text{-}\beta}$, $121.1^{Z\text{-}\beta}$, $120.7^{E\text{-}\beta}$, $118.5^{E\text{-}\beta}$, $114.5^{Z\text{-}\beta}$, $113.7^{E\text{-}\beta}$, $101.5^{E+Z\text{-}\beta}$, $88.3^{E\text{-}\beta}$, $88.1^{E\text{-}\beta}$, $87.8^{Z\text{-}\beta}$, $52.8^{E\text{-}\beta}$, $52.6^{Z\text{-}\beta}$, $43.3^{Z\text{-}\beta}$, $43.1^{E\text{-}\beta}$, $29.9^{Z\text{-}\beta}$, $28.4^{E\text{-}\beta}$, $18.7^{E+Z\text{-}\beta}$, $18.0^{E+Z\text{-}\beta}$, $12.9^{E+Z\text{-}\beta}$, $11.3^{E+Z\text{-}\beta}$ ppm.

HRMS (ESI+NH_4^+) m/z: Calculated for $C_{34}H_{60}O_4N_1Si_2$ 602.40554, Found 602.40554.

(*E*)-3-(triisopropylsilyl)prop-2-yn-1-yl 3-(2,4-dimethyl-3-(2-(2-oxopyrrolidin-1-yl)acetamido)phenyl)acrylate (3p-*α*)

(*E*)-3-(triisopropylsilyl)prop-2-yn-1-yl 3-(3,5-dimethyl-4-(2-(2-oxopyrrolidin-1-yl)acetamido)phenyl)acrylate (3p-*β*)

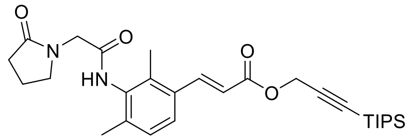

3p-*α*

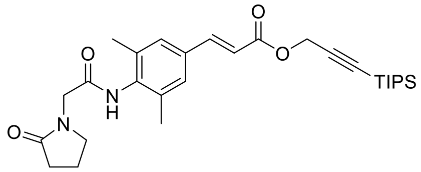

3p-*β*

According to the general procedure, compound **3p** was synthesized using Nefiracetam **1p** (49.3 mg, 0.200 mmol). The target compound was purified by column chromatography (EtOAc) and obtained as a yellow oil (20.9 mg, 40.9 µmol, 21%, *α:β* = 22:78).

^{1}H-NMR (600 MHz, $CDCl_3$): δ = 7.98 (d, *J* = 15.8 Hz, 1H^{α}), 7.90 (m, 1$H^{\alpha+\beta}$), 7.62 (d, *J* = 16.0 Hz, 1H^{β}), 7.42 (d, *J* = 8.0 Hz, 1H^{α}), 7.23 (s, 2H^{β}), 7.09 (d, *J* = 8.0 Hz, 1H^{α}), 6.40 (d, *J* = 16.0 Hz, 1H^{β}), 6.32 (d, *J* = 15.8 Hz, 1H^{α}), 4.83 (m, 2$H^{\alpha+\beta}$), 4.11 (m, 2$H^{\alpha+\beta}$), 3.61 (m, 2$H^{\alpha+\beta}$), 2.47 (m, 2$H^{\alpha+\beta}$), 2.23 (m, 6$H^{\alpha+\beta}$), 2.17 – 2.11 (m, 2$H^{\alpha+\beta}$), 1.08 (m, 21$H^{\alpha+\beta}$) ppm.

^{13}C-NMR (151 MHz, $CDCl_3$): δ = $176.6^{\alpha+\beta}$, 167.3^{α}, 167.1^{β}, $166.2^{\alpha+\beta}$, 145.1^{β}, 143.4^{α}, 137.8^{α}, 135.8^{β}, 135.6^{β}, 135.3^{α}, 134.2^{α}, 133.3^{β}, 132.5^{α}, 128.4^{α}, 128.2^{β}, 126.0^{α}, 118.7^{α}, 117.5^{β}, $101.1^{\alpha+\beta}$, 88.6^{α}, 88.6^{β}, $53.1^{\alpha+\beta}$, 48.94^{β}, 48.85^{α}, 48.11^{β}, 48.05^{α}, $30.5^{\alpha+\beta}$, $18.7^{\alpha+\beta}$, $18.6^{\alpha+\beta}$, $18.4^{\alpha+\beta}$, $11.2^{\alpha+\beta}$ ppm.

HRMS (ESI+H^+) m/z: Calculated for $C_{29}H_{43}O_4N_2Si$ 511.29866, Found 511.29793.

(*E*)-3-(triisopropylsilyl)prop-2-yn-1-yl 3-(3,5-diisopropyl-4-methoxyphenyl)acrylate (3q-*β*)

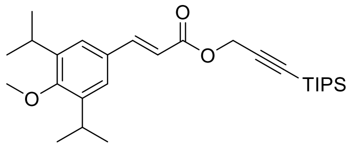

3q-*β*

According to the general procedure, compound **3q** was synthesized using 1,3-diisopropyl-2-methoxybenzene **1q** (38.7 mg, 0.200 mmol). The target compound was purified by column chromatography (*n*-pentane:Et_2O 99:1 → 94:6) and obtained as a yellow oil (26.8 mg, 58.7 µmol, 29%, only *β*).

^{1}H-NMR (600 MHz, $CDCl_3$): δ = 7.70 (d, *J* = 16.0 Hz, 1H), 7.29 (s, 2H), 6.40 (d, *J* = 16.0 Hz, 1H), 4.85 (s, 2H), 3.75 (s, 3H), 3.33 (hept, *J* = 6.9 Hz, 2H), 1.25 (s, 6H), 1.24 (s, 6H), 1.09 (m, 21H) ppm.
^{13}C-NMR (151 MHz, $CDCl_3$): δ = 166.3, 156.9, 145.9, 142.5, 130.5, 124.4, 115.8, 101.1, 88.3, 62.3, 52.8, 26.5, 23.9, 18.5, 11.1 ppm.
HRMS (EI) m/z: Calculated for $C_{28}H_{44}O_3Si_1$ 456.30597, Found 456.30573.

(*E*)-3-(triisopropylsilyl)prop-2-yn-1-yl 3-((7a*S*,10a*S*,11a*S*)-3-methoxy-10a-methyl-10-oxo-6,6a,7,7a,8,9,10,10a,11,11a-decahydro-*5H*-cyclopenta[b]phenanthren-2-yl)acrylate (3r-*α*-*E*)
(*Z*)-3-(triisopropylsilyl)prop-2-yn-1-yl 3-((7a*S*,10a*S*,11a*S*)-3-methoxy-10a-methyl-10-oxo-6,6a,7,7a,8,9,10,10a,11,11a-decahydro-*5H*-cyclopenta[b]phenanthren-2-yl)acrylate (3r-*α*-*Z*)
(*E*)-3-(triisopropylsilyl)prop-2-yn-1-yl 3-((7a*S*,10a*S*,11a*S*)-3-methoxy-10a-methyl-10-oxo-6,6a,7,7a,8,9,10,10a,11,11a-decahydro-*5H*-cyclopenta[b]phenanthren-1-yl)acrylate (3r-*β*-*E*)
(*E*)-3-(triisopropylsilyl)prop-2-yn-1-yl 3-((7a*S*,10a*S*,11a*S*)-3-methoxy-10a-methyl-10-oxo-6,6a,7,7a,8,9,10,10a,11,11a-decahydro-*5H*-cyclopenta[b]phenanthren-4-yl)acrylate (3r-*α´*-*E*)

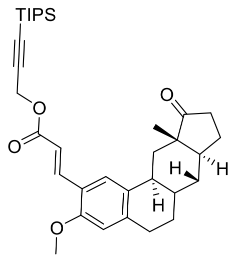
3r-*α*-*E*

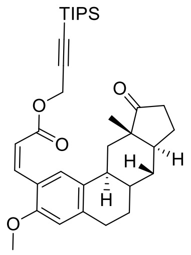
3r-*α*-*Z*

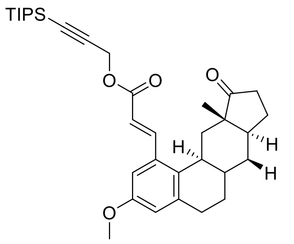
3r-*β*-*E*

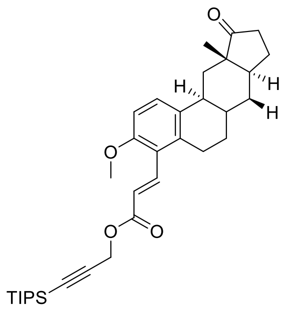
3r-*α´*-*E*

According to the general procedure, compound **3r** was synthesized using methyl ether Estrone **1r** (56.8 mg, 0.200 mmol). The target compound was purified by column chromatography (*n*-pentane:Et_2O 96:4 → 80:20) and obtained as a yellow oil (39.0 mg, 58.7 µmol, 36%, *α-E*+*α-Z*:*α’-E*:*β-E* = 90:5:5).

^{1}H-NMR (600 MHz, $CDCl_3$): δ = 7.98 (d, *J* = 16.2 Hz, 1H$^{E\text{-}\alpha}$), 7.92 (d, *J* = 16.2 Hz, 1H$^{E\text{-}\alpha'}$), 7.67 (s, 1H$^{Z\text{-}\alpha}$), 7.46 (dd, *J* = 15.8, 0.8 Hz, 1H$^{E\text{-}\beta}$), 7.43 (s, 1H$^{E\text{-}\alpha}$), 7.18 (d, *J* = 11.9 Hz, 1H$^{Z\text{-}\alpha}$), 6.98 (s, 1H$^{E\text{-}\beta}$), 6.69 (d, *J* = 16.1 Hz, 1H$^{E\text{-}\alpha'}$), 6.64 (s, 1H$^{E\text{-}\alpha}$), 6.59 (s, 1H$^{Z\text{-}\alpha}$), 6.54 (d, *J* = 16.1 Hz, 1H$^{E\text{-}\alpha}$), 6.12 (dd, *J* = 15.8, 0.7 Hz, 1H$^{E\text{-}\beta}$), 5.96 (d, *J* = 12.6 Hz, 1H$^{Z\text{-}\alpha}$), 4.85 (s, 2H$^{E\text{-}\alpha'+\beta}$), 4.83 (s, 2H$^{E\text{-}\alpha}$), 4.80 (s, 2H$^{E\text{-}\alpha'+\beta}$), 4.73 (d, *J* = 1.3 Hz, 2H$^{Z\text{-}\alpha}$), 3.86 (s, 3H$^{E\text{-}\alpha+\alpha'+\beta}$), 3.80 (s, 3H$^{Z\text{-}\alpha}$), 2.99 – 2.87 (m, 2H$^{E\text{-}\alpha+\alpha'+\beta+Z\text{-}\alpha}$), 2.56 – 2.40 (m, 2H$^{E\text{-}\alpha+\alpha'+\beta+Z\text{-}\alpha}$), 2.29 – 2.22 (m, 1H$^{E\text{-}\alpha+\alpha'+\beta+Z\text{-}\alpha}$), 2.20 – 2.10 (m, 1H$^{E\text{-}\alpha+\alpha'+\beta+Z\text{-}\alpha}$), 2.09 – 1.94 (m, $^{E\text{-}\alpha+\alpha'+\beta+Z\text{-}\alpha}$), 1.68 – 1.40 (m, 6H$^{E\text{-}\alpha+\alpha'+\beta+Z\text{-}\alpha}$), 1.11 – 1.02 (m, 21H$^{E\text{-}\alpha+\alpha'+\beta+Z\text{-}\alpha}$), 0.91 (d, *J* = 3.6 Hz, 3H$^{E\text{-}\alpha+\alpha'+\beta+Z\text{-}\alpha}$) ppm.

^{13}C-NMR (151 MHz, CDCl$_3$): δ = $220.8^{E+Z-\alpha}$, $167.0^{E+Z-\alpha}$, $156.7^{E+Z-\alpha}$, $155.5^{Z-\alpha}$, $141.5^{E-\alpha}$, $141.0^{E-\alpha}$, $140.5^{Z-\alpha}$, $132.2^{E-\alpha}$, $128.3^{Z-\alpha}$, $126.4^{E+Z-\alpha}$, $121.0^{E+Z-\alpha}$, $118.1^{Z-\alpha}$, $116.9^{E-\alpha}$, $111.6^{E-\alpha}$, $110.8^{Z-\alpha}$, $101.5^{E+Z-\alpha}$, $88.3^{E+Z-\alpha}$, $55.6^{E+Z-\alpha}$, $52.9^{E-\alpha}$, $52.6^{Z-\alpha}$, $50.5^{E+Z-\alpha}$, $48.1^{E+Z-\alpha}$, $44.1^{Z-\alpha}$, $43.9^{E-\alpha}$, $38.4^{E+Z-\alpha}$, $36.0^{E+Z-\alpha}$, $31.6^{E+Z-\alpha}$, $30.1^{E+Z-\alpha}$, $26.5^{E+Z-\alpha}$, $26.0^{E+Z-\alpha}$, $21.7^{E+Z-\alpha}$, $18.7^{E+Z-\alpha}$, $14.0^{E+Z-\alpha}$, $11.3^{E+Z-\alpha}$ ppm.
HRMS (ESI+NH$_4^+$) m/z: Calculated for $C_{34}H_{52}O_4N_1Si_1$ 566.36601, Found 566.36511.

(*E*)-3-(triisopropylsilyl)prop-2-yn-1-yl 3-(5-chloro-2-((1-ethoxy-2-methyl-1-oxopropan-2-yl)oxy)phenyl)acrylate (3s-*α-E*)
(*Z*)-3-(triisopropylsilyl)prop-2-yn-1-yl 3-(5-chloro-2-((1-ethoxy-2-methyl-1-oxopropan-2-yl)oxy)phenyl)acrylate (3s-*α-Z*)
(*E*)-3-(triisopropylsilyl)prop-2-yn-1-yl 3-(2-chloro-5-((1-ethoxy-2-methyl-1-oxopropan-2-yl)oxy)phenyl)acrylate (3s-*β-E*)
(*Z*)-3-(triisopropylsilyl)prop-2-yn-1-yl 3-(2-chloro-5-((1-ethoxy-2-methyl-1-oxopropan-2-yl)oxy)phenyl)acrylate (3s-*β-Z*)

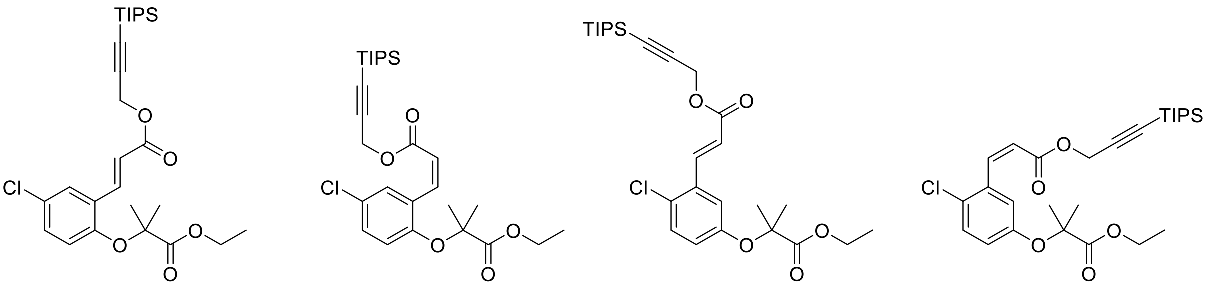

3s-*α-E* **3s-*α-Z*** **3s-*β-E*** **3s-*β-Z***

According to the general procedure, compound **3s** was synthesized using Clofibrate **1s** (48.5 mg, 0.200 mmol). The target compound was purified by column chromatography (Cy:CH$_2$Cl$_2$ 6:4) and obtained as a yellow oil (27.9 mg, 55.0 µmol, 28%, *α-E:α-Z:β-E:β-Z* = 62:28:8:2).

^{1}H-NMR (600 MHz, CDCl$_3$): δ = 8.05 (d, J = 16.0 Hz, 1H$^{E-\beta}$), 8.00 (d, J = 16.2 Hz, 1H$^{E-\alpha}$), 7.55 (d, J = 2.6 Hz, 1H$^{Z-\alpha}$), 7.51 (d, J = 2.7 Hz, 1H$^{E-\alpha}$), 7.27 (d, 1H$^{E-\beta}$), 7.19 (dd, J = 8.9, 2.7 Hz, 1H$^{E-\alpha}$), 7.16 – 7.12 (m, 1H$^{E-\beta}$+2H$^{Z-\alpha}$), 7.08 (d, J = 12.3 Hz, 1H$^{Z-\beta}$), 6.81 (dd, J = 8.8, 2.6 Hz, 1H$^{E-\beta}$), 6.71 – 6.67 (m, 1H$^{E-\beta}$+1H$^{Z-\alpha}$), 6.48 (d, J = 16.2 Hz, 1H$^{E-\alpha}$), 6.37 (d, J = 16.0 Hz, 1H$^{E-\beta}$), 6.09 (d, J = 12.3 Hz, 1H$^{Z-\beta}$), 6.02 (d, J = 12.5 Hz, 1H$^{Z-\alpha}$), 4.84 (m, 2H$^{E-\beta}$+2H$^{E-\alpha}$), 4.73 (s, 2H$^{Z-\alpha}$), 4.72 (s, 2H$^{Z-\beta}$), 4.23 (m, 2H$^{E-\alpha+Z-\alpha+E-\beta+Z-\beta}$), 1.63 (s, 6H$^{E-\alpha}$), 1.60 (s, 6H$^{E-\beta}$), 1.58 (s, 6H$^{Z-\alpha}$), 1.56 (s, 6H$^{Z-\beta}$), 1.27 – 1.24 (m, 3H$^{E-\alpha+Z-\alpha+E-\beta+Z-\beta}$), 1.10 – 1.04 (m, 21H$^{E-\alpha+Z-\alpha+E-\beta+Z-\beta}$) ppm.

^{13}C-NMR (151 MHz, $CDCl_3$): δ = 174.0$^{Z-\alpha}$, 173.9$^{E-\beta}$, 173.8$^{E-\alpha}$, 166.1$^{E-\alpha}$, 165.7$^{E-\beta}$, 165.0$^{Z-\alpha}$, 154.5$^{E-\beta}$, 153.1$^{E-\alpha}$, 152.2$^{Z-\alpha}$, 141.3$^{E-\beta}$, 139.6$^{E-\alpha}$, 133.2$^{E-\beta}$, 130.7$^{Z-\alpha}$, 130.6$^{E-\alpha+E-\beta}$, 129.6$^{Z-\alpha}$, 128.9$^{Z-\alpha}$, 128.3$^{E-\beta}$, 128.0$^{E-\alpha}$, 127.7$^{E-\alpha}$, 127.4$^{E-\alpha}$, 126.7$^{Z-\alpha}$, 122.2$^{E-\beta}$, 120.3$^{Z-\alpha}$, 120.2$^{E-\beta}$, 119.2$^{E-\alpha}$, 118.7$^{E-\alpha}$, 118.4$^{Z-\alpha}$, 118.3$^{E-\beta}$, 101.1$^{E-\alpha}$, 101.0$^{E-\beta}$, 100.9$^{Z-\alpha}$, 88.8$^{E-\beta}$, 88.6$^{E-\alpha}$, 88.5$^{Z-\alpha}$, 80.5$^{E-\alpha}$, 80.4$^{Z-\alpha}$, 79.9$^{E-\beta}$, 61.9$^{E-\alpha}$, 61.82$^{E-\beta}$, 61.75$^{Z-\alpha}$, 53.3$^{E-\beta}$, 53.2$^{E-\alpha}$, 52.9$^{Z-\alpha}$, 25.5$^{E-\alpha+E-\beta+Z-\alpha}$, 18.7 $^{E-\alpha+E-\beta+Z-\alpha}$, 14.3$^{E-\beta}$, 14.2$^{E-\alpha+Z-\alpha}$, 11.2 $^{E-\alpha+E-\beta+Z-\alpha}$ ppm.

HRMS (ESI+H$^+$) m/z: Calculated for $C_{27}H_{40}O_5ClSi$ 507.23281, Found 507.23197.

(*E*)-3-(triisopropylsilyl)prop-2-yn-1-yl 3-(5-(2,2-dichlorocyclopropyl)-2-((1-methoxy-2-methyl-1-oxopropan-2-yl)oxy)phenyl)acrylate (3t-*α-E*)

(*Z*)-3-(triisopropylsilyl)prop-2-yn-1-yl 3-(5-(2,2-dichlorocyclopropyl)-2-((1-methoxy-2-methyl-1-oxopropan-2-yl)oxy)phenyl)acrylate (3t-*α-Z*)

(*E*)-3-(triisopropylsilyl)prop-2-yn-1-yl 3-(2-(2,2-dichlorocyclopropyl)-5-((1-methoxy-2-methyl-1-oxopropan-2-yl)oxy)phenyl)acrylate (3t-*β-E*)

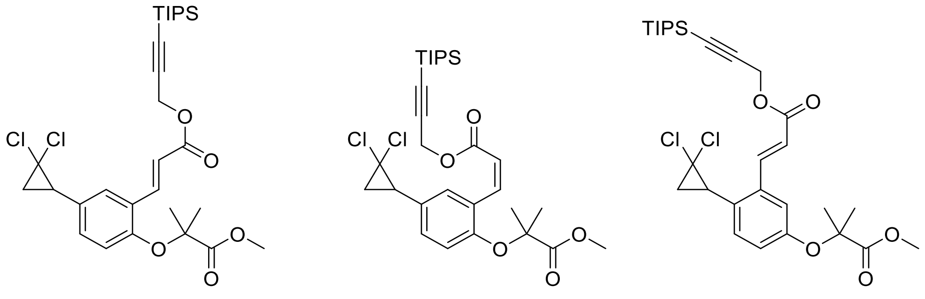

3t-*α-E* **3t-*α-Z*** **3t-*β-E***

According to the general procedure, compound **3t** was synthesized using Ciprofibrate Methyl-ester **1t** (60.6 mg, 0.200 mmol). The target compound was purified by column chromatography (Cy:CH_2Cl_2 6:4) and obtained as a transparent oil (36.6 mg, 64.5 µmol, 32%, *α-E:α-Z:β-E* = 78:17:5).

^{1}H-NMR (600 MHz, $CDCl_3$): δ = 8.10 (d, *J* = 15.9 Hz, 1H$^{E-\beta}$), 8.05 (d, *J* = 16.2 Hz, 1H$^{E-\alpha}$), 7.47 (d, *J* = 2.3 Hz, 1H$^{Z-\alpha}$), 7.41 (d, *J* = 2.2 Hz, 1H$^{E-\alpha}$), 7.21 (d, *J* = 12.5 Hz, 1H$^{Z-\alpha}$), 7.16 (d, *J* = 2.6 Hz, 1H$^{E-\beta}$), 7.13 (dd, *J* = 8.6, 2.4 Hz, 1H$^{E-\alpha}$), 7.09 (dd, *J* = 8.6, 2.4 Hz, 1H$^{Z-\alpha}$), 6.99 (d, *J* = 8.5 Hz, 1H$^{E-\beta}$), 6.78 (dd, *J* = 8.5, 2.6 Hz, 1H$^{E-\beta}$), 6.67 (m, 1H$^{E-\alpha+Z-\alpha}$), 6.51 (d, *J* = 16.2 Hz, 1H$^{E-\alpha}$), 6.41 (d, *J* = 15.9 Hz, 1H$^{E-\beta}$), 6.01 (d, *J* = 12.5 Hz, 1H$^{Z-\alpha}$), 4.87 (s, 2H$^{E-\beta}$), 4.84 (s, 2H$^{E-\alpha}$), 4.72 (s, 2H$^{Z-\alpha}$), 3.76 (s, 3H$^{E-\alpha+E-\beta}$), 3.75 (s, 3H$^{Z-\alpha}$), 2.83 (m, 1H$^{E-\alpha+Z-\alpha+E-\beta}$), 2.03 (dd, *J* = 10.5, 7.3 Hz, 1H$^{E-\beta}$), 1.97 (dd, *J* = 10.7, 7.5 Hz, 1H$^{E-\alpha}$), 1.92 (dd, *J* = 10.7, 7.4 Hz, 1H$^{Z-\alpha}$), 1.79 (m, 1H$^{E-\alpha+Z-\alpha+E-\beta}$), 1.64 (s, 6H$^{E-\alpha}$), 1.61 (s, 6H$^{E-\beta}$), 1.57 (s, 6H$^{Z-\alpha}$), 1.08 (m, 21H$^{E-\alpha+E-\beta}$), 1.05 (m, 21H$^{Z-\alpha}$) ppm.

^{13}C-NMR (151 MHz, CDCl$_3$): δ = 174.7$^{Z-α}$, 174.5$^{E-α}$, 166.4$^{E-α}$, 165.9$^{E-β}$, 165.2$^{Z-α}$, 155.3$^{E-β}$, 153.9$^{E-α}$, 152.9$^{Z-α}$, 142.4$^{E-β}$, 140.7$^{E-α}$, 140.6$^{Z-α}$, 131.5$^{E-α}$, 130.5$^{Z-α}$, 130.0$^{E-β}$, 129.0$^{E-α}$, 128.4$^{E-α}$, 127.7$^{Z-α}$, 127.1$^{Z-α}$, 126.0$^{E-α}$, 120.3$^{E-β}$, 119.9$^{E-β}$, 119.5$^{Z-α}$, 118.4$^{E-α}$, 117.2$^{E-β}$, 117.0$^{E-α}$, 116.7$^{Z-α}$, 101.2$^{E-α}$, 101.1$^{Z-α}$, 101.0$^{E-β}$, 88.5$^{E-α+E-β}$, 88.4$^{Z-α}$, 80.2$^{E-α}$, 80.1$^{Z-α}$, 79.6$^{E-β}$, 60.9$^{Z-α}$, 60.7$^{E-α}$, 60.5$^{E-β}$, 53.3$^{E-β}$, 53.1$^{E-α+Z-α}$, 52.8$^{E-α+E-β}$, 52.7$^{Z-α}$, 34.9$^{Z-α}$, 34.8$^{E-α+E-β}$, 26.0$^{E-α+ Z-α+E-β}$, 25.5$^{E-α+ Z-α+E-β}$, 18.7$^{E-α+ Z-α+E-β}$, 11.2$^{E-α+ Z-α+E-β}$ ppm.
HRMS (ESI+H$^+$) m/z: Calculated for $C_{29}H_{41}O_5Cl_2Si$ 567.20948, Found 567.20853.

(*E*)-3-(triisopropylsilyl)prop-2-yn-1-yl 3-(4-((4a*S*,6a*R*,8a*R*,8b*R*,9a*S*,12*S*,12a*S*,14b*R*)-6,6,8a,12a-tetramethyl-3,8,10-trioxohexadecahydrooxireno[2,3-d]pyrano[4',3':3,3*a*]isobenzofuro[5,4-*f*]isochromen-12-yl)furan-2-yl)acrylate (3u-*α*)

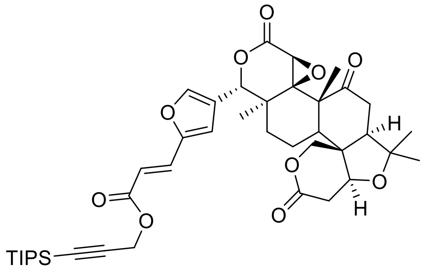

3u-*α*

According to the general procedure, compound **3u** was synthesized using Limonin **1u** (94.1 mg, 0.200 mmol). The target compound was purified by column chromatography (*n*-pentane:EtOAc 9:1 → 6:4) and obtained as a yellow solid (60.3 mg, 82.0 µmol, 41%, *α* only). The large scale reaction was performed in a 25 mL Schlenk tube using Pd(OAc)$_2$ (22.5 mg, 0.100 mmol, 10 mol%), monodentate ligand **L1b** (21.6 mg, 0.200 mmol, 20 mol%), bidentate ligand **L2b** (69.2 mg, 0.200 mmol, 20 mol%), AgOAc (333.0 mg, 2.000 mmol, 2 equiv), Limonin **1u** (470.5 mg, 1.000 mmol), 3-(triisopropylsilyl)prop-2-yn-1-yl acrylate **2** (799.4 mg, 3.000 mmol, 3 equiv), and the solvent mixture TFE:CHCl$_3$ 2:8 (6 mL). The reaction was stirred at 75°C for 72 hours. The target compound was purified by column chromatography (*n*-pentane:EtOAc 9:1 → 4:6) and obtained as a yellow solid (359 mg, 488 µmol, 49%).

^{1}H-NMR (600 MHz, CDCl$_3$): δ = 7.46 (s, 1H), 7.38 (d, *J* = 15.8 Hz, 1H), 6.55 (s, 1H), 6.34 (d, *J* = 15.6 Hz, 1H), 5.44 (s, 1H), 4.81 (s, 2H), 4.76 (d, *J* = 13.3 Hz, 1H), 4.46 (d, *J* = 13.1 Hz, 1H), 4.0 (bs, 2H), 2.96 (dd, *J* = 16.7, 3.8 Hz, 1H), 2.86 (dd, *J* = 15.8, 14.5 Hz, 1H), 2.67 (dd, *J* = 16.7, 1.9 Hz, 1H), 2.54 (dd, *J* = 12.5, 2.9 Hz, 1H), 2.45 (dd, *J* =

14.6, 3.3 Hz, 1H), 2.23 (dd, *J* = 15.9, 3.4 Hz, 1H), 1.94 – 1.86 (m, 1H), 1.84 – 1.75 (m, 2H), 1.57 – 1.48 (m, 1H), 1.28 (bs, 3H), 1.16 (bs, 3H), 1.15 (bs, 3H), 1.06 (m, 24H) ppm.

^{13}C-NMR (151 MHz, CDCl$_3$): δ = 206.2, 169.2, 166.4, 165.9, 151.4, 143.1, 131.2, 122.9, 116.5, 114.2, 100.9, 88.7, 80.5, 79.3, 77.6, 65.7, 65.5, 60.7, 53.9, 53.2, 51.5, 48.3, 46.1, 38.0, 36.5, 35.8, 31.0, 30.3, 21.5, 20.8, 19.0, 18.7, 17.7, 11.2.

HRMS (ESI+H$^+$) m/z: Calculated for $C_{41}H_{55}O_{10}Si_1$ 735.35590, Found 735.35519.

(*E*)-3-(triisopropylsilyl)prop-2-yn-1-yl 3-(1,8-diethyl-1-(2-methoxy-2-oxoethyl)-1,3,4,9-tetrahydropyrano[3,4-*b*]indol-6-yl)acrylate (3v-*β*)

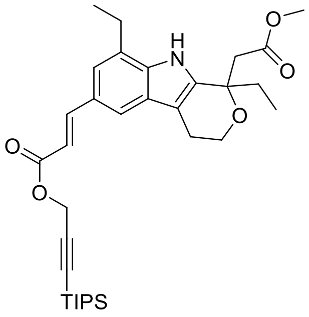

3v-*β*

According to the general procedure, compound **3v** was synthesized using Etodolac **1v** (60.3 mg, 0.200 mmol). The target compound was purified by column chromatography (cy:CH_2Cl_2 6:4) and obtained as a colorless solid (17.7 mg, 31.3 µmol, 16%, *β* only).

^{1}H-NMR (600 MHz, CDCl$_3$): δ = 9.33 (s, 1H), 7.86 (d, *J* = 15.9 Hz, 1H), 7.54 (d, *J* = 1.5 Hz, 1H), 7.25 (d, *J* = 1.5 Hz, 1H), 6.45 (d, *J* = 15.9 Hz, 1H), 4.85 (s, 2H), 4.05 (dt, *J* = 11.4, 4.9 Hz, 1H), 3.94 (ddd, *J* = 11.4, 7.4, 4.4 Hz, 1H), 3.73 (s, 3H), 3.03 (d, *J* = 16.8 Hz, 1H), 2.93 (d, *J* = 16.8 Hz, 1H), 2.91 – 2.86 (m, 2H), 2.86 – 2.73 (m, 2H), 2.15 (dq, *J* = 14.6, 7.3 Hz, 1H), 1.99 (dq, *J* = 14.6, 7.3 Hz, 1H), 1.39 (t, *J* = 7.6 Hz, 3H), 1.09 (m, 21H), 0.83 (t, *J* = 7.4 Hz, 3H) ppm.

^{13}C-NMR (151 MHz, CDCl$_3$): δ = 173.6, 167.0, 147.9, 137.4, 136.2, 127.3, 126.7, 126.4, 120.2, 118.2, 113.7, 109.5, 101.6, 88.2, 74.6, 60.6, 52.8, 52.2, 42.8, 30.6, 24.3, 22.4, 18.7, 13.7, 11.3, 7.7 ppm.

HRMS (ESI+H$^+$) m/z: Calculated for $C_{33}H_{48}O_5N_1Si_1$ 566.32963, Found 566.32891.

List of Poorly Performing and Failed Entries

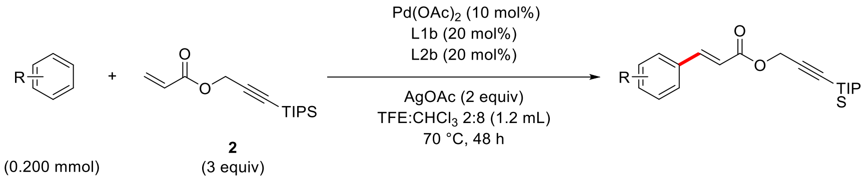

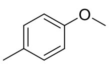

NMR-Yield 40%

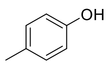

Decomposition

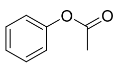

NMR-Yield 19%

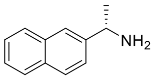

No reaction

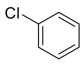

NMR-Yield 22%

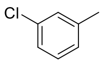

NMR-Yield 27%

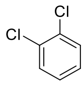

NMR-Yield 6%

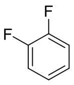

NMR-Yield 22%

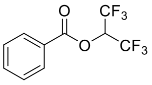

Isolated Yield 12%

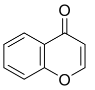

Olefination of the double bond

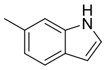

Decomposition

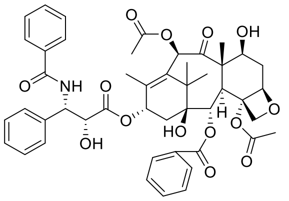

Isolated Yield 20%
complex mixture

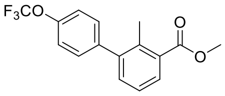

NMR-Yield 40%
challenging purification

Yields were determined by ^{1}H-NMR using dibromomethane as internal standard.

6. Further Derivatization of Product Molecules

(*E*)-prop-2-yn-1-yl-3-(3,4-dimethylphenyl)acrylate (4)

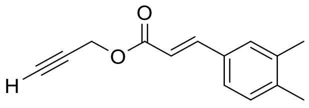

4

Compound **4** was synthetized adapting a literature procedure by Reissig et al.[14], as follows: compound **3a** (589 mg, 1.59 mmol) was dissolved in dry THF (16 mL), a solution of TBAF (1 M in THF, 4.77 mL, 4.77 mmol, 3 equiv) was added and the mixture was stirred for 1 h at rt under a N_2 atmosphere. The reaction mixture was filtered over Celite and concentrated under reduced pressure. The brown solid obtained as crude product was purified by column chromatography (*n*-pentane:EtOAc 98:2) and the target compound was obtained as colorless solid (246 mg, 1.15 mmol, 72%).

^{1}H-NMR (600 MHz, $CDCl_3$): δ = 8.13 (d, *J* = 15.8, 1H$^{\alpha}$), 7.70 (d, *J* = 16 Hz, 1H$^{\beta}$), 7.31 (s, 1H$^{\beta}$), 7.28 (dd, *J* = 7.80, 1.70 Hz, 1H$^{\beta}$), 7.15 (d, *J* = 7.80 Hz, 1H$^{\beta}$), 6.41 (d, *J* = 16 Hz, 1H$^{\beta}$), 6.34 (d, *J* = 15.8, 1H$^{\alpha}$), 4.82 (d, *J* = 2.50 Hz, 2H$^{\alpha}$), 4.81 (d, *J* = 2.50 Hz, 2H$^{\beta}$), 2.50 (t, *J* = 2.50 Hz, 1H$^{\beta}$), 2.33 (s, 3H$^{\alpha}$), 2.32 (s, 3H$^{\alpha}$), 2.29 (s, 3H$^{\beta}$), 2.28 (s, 3H$^{\beta}$) ppm.
^{13}C-NMR (151 MHz, $CDCl_3$): δ = 166.3, 146.2, 139.8, 137.2, 131.9, 130.2, 129.4, 125.8, 115.7, 77.9, 74.8, 51.9, 19.8, 19.7 ppm.
HRMS (ESI+H$^+$) m/z: Calculated for $C_{14}H_{15}O_2$ 215.10666, Found 215.10666.

(2*R*,3*S*,4*S*,5*R*,6*R*)-2-(acetoxymethyl)-6-(4-((((*E*)-3-(3,4-dimethylphenyl)acryloyl)oxy)methyl)-*1H*-1,2,3-triazol-1-yl)tetrahydro-*2H*-pyran-3,4,5-triyl triacetate (6)

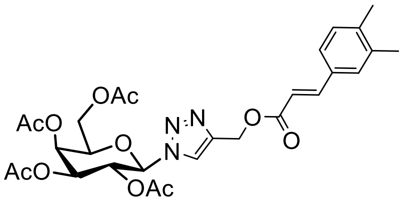

6

Compound **6** was synthetized adapting a literature procedure by Freitas et al.[12], as follows: compound **5** (74.7 mg, 0.200 mmol) and compound **4** (47.1 mg, 220 µmol, 1.1 equiv) were transferred into a 10 mL Schlenk tube. Copper(II) sulfate tetrahydrate (18.5 mg, 80.0 µmol, 0.4 equiv) and sodium ascorbate (8.0 mg, 40 µmol, 0.2 equiv) were added and all components were dissolved in a H_2O/CH_2Cl_2 mixture (1:1 , 2.5 mL). The reaction mixture was stirred at rt for 1 h, diluted with CH_2Cl_2 (5 mL), washed with water and brine (15 + 15 mL), and dried over $MgSO_4$. The solvent was evaporated under reduced pressure and the crude product was purified by column chromatography (EtOAc). The target compound was obtained as a colorless solid (116.5 mg, 198.0 µmol, 99%, only *β* isomer).

^{1}H-NMR (600 MHz, $CDCl_3$): δ = 7.95 (s, 1H), 7.67 (d, *J* = 16.0 Hz, 1H), 7.28 (s, 1H), 7.25 (dd, *J* = 7.8, 1.9 Hz, 1H), 7.12 (d, *J* = 7.8 Hz, 1H), 6.40 (d, *J* = 16.0 Hz, 1H), 5.85 (d, *J* = 9.3 Hz, 1H), 5.57 – 5.53 (m, 2H), 5.38 (dd, *J* = 12.9, 0.5 Hz, 1H), 5.31 (dd, *J* = 12.9, 0.5 Hz, 1H), 5.25 (dd, *J* = 10.3, 3.4 Hz, 1H), 4.25 – 4.17 (m, 2H), 4.16 – 4.11 (m, 1H), 2.26 (s, 3H), 2.26 (s, 3H), 2.22 (s, 3H), 2.03 (s, 3H), 2.00 (s, 3H), 1.87 (s, 3H) ppm.

^{13}C-NMR (151 MHz, $CDCl_3$): δ = 170.4, 170.1, 169.9, 169.1, 167.0, 146.0, 143.9, 139.8, 137.3, 132.0, 130.3, 129.5, 125.9, 122.4, 116.1, 86.4, 74.2, 70.9, 68.0, 66.9, 61.3, 57.5, 20.8, 20.7, 20.6, 20.3, 19.9, 19.8 ppm.

HRMS (ESI+H$^+$) m/z: Calculated for $C_{28}H_{34}O_{11}N_3$ 588.21879, Found 588.21869.

(2*E*,2'*E*)-(1,1'-(((oxybis(ethane-2,1-diyl))bis(oxy))bis(ethane-2,1-diyl))bis(1*H*-1,2,3-triazole-4,1-diyl))bis(methylene) bis(3-(3,4-dimethylphenyl)acrylate) (8)

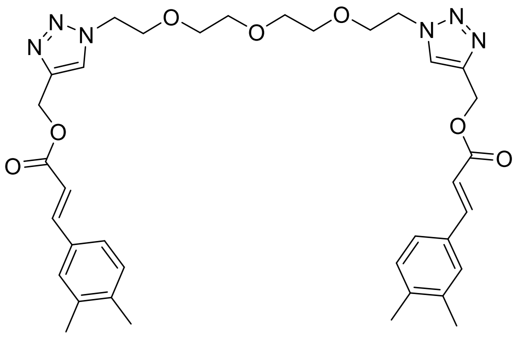

8

Compound **8** was synthetized adapting a literature procedure Freitas et al.[12], as follows: compound **7** (48.9 mg, 0.200 mmol) and compound **4** (94.3 mg, 0.440 mmol, 2.2 equiv) were transferred into a 10 mL Schlenk tube. Copper(II) sulfate tetrahydrate (18.5 mg, 80.0 µmol, 0.4 equiv) and sodium ascorbate (8.0 mg, 40 µmol, 0.2 equiv) were added and all components were dissolved in a H_2O/CH_2Cl_2 mixture (1:1, 2.5 mL). The reaction mixture was stirred at rt for 1 h, diluted with CH_2Cl_2 (5 mL), washed with water and brine (15 + 15 mL), and dried over $MgSO_4$. The solvent was evaporated under reduced pressure and the crude product purified by column chromatography (*n*-pentane:MeOH 8:2). The target compound was obtained as a transparent oil (106.6 mg, 158.4 µmol, 80%, only (*β,β*) product).

^{1}H-NMR (600 MHz, $CDCl_3$): δ = 7.81 (s, 2H), 7.63 (d, *J* = 16.0 Hz, 2H), 7.24 (d, *J* = 2.0 Hz, 2H), 7.21 (dd, *J* = 7.8, 2.0 Hz, 2H), 7.10 (d, *J* = 7.8 Hz, 2H), 6.36 (d, *J* = 16.0 Hz, 2H), 5.32 (s, 4H), 4.51 (t, *J* = 5.1 Hz, 4H), 3.84 (t, *J* = 5.1 Hz, 4H), 3.58 – 3.51 (m, 8H), 2.24 (s+s, 12H) ppm.

^{13}C-NMR (151 MHz, $CDCl_3$): δ = 166.9, 145.7, 142.9, 139.7, 137.2, 131.9, 130.2, 129.3, 125.72, 125.0, 116.2, 70.4, 70.3, 69.3, 57.6, 50.2, 19.8, 19.7 ppm.

HRMS (ESI+H$^+$) m/z: Calculated for $C_{36}H_{44}N_6O_7$ 673.33442, Found 673.33430.

(*E*)-prop-2-yn-1-yl 3-(4-((4a*S*,6a*R*,8a*R*,8b*R*,9a*S*,12*S*,12a*S*,14b*R*)-6,6,8a,12a-tetramethyl-3,8,10-trioxohexadecahydrooxireno[2,3-*d*]pyrano[4',3':3,3*a*]isobenzofuro[5,4-*f*]isochromen-12-yl)furan-2-yl)acrylate (9)

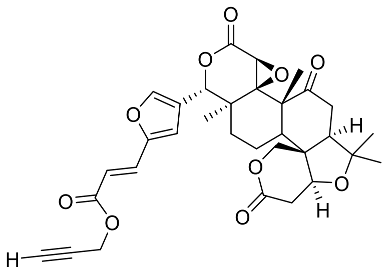

9

Compound **3u** (297 mg, 0.404 mmol) was dissolved in dry THF (15 mL) and cooled to 0°C. A solution of TBAF (1 M in THF, 0.48 mL, 0.480 mmol, 1.2 equiv) was added under vigorous stirring, keeping the temperature at 0°C. After 25 min the reaction was quenched with NH_4Cl (5 mL) and diluted with EtOAc (40 mL). The aqueous phase was extracted with EtOAc (5 x 50 mL), the combined organic phases were dried over Na_2SO_4 and concentrated under reduced pressure, and the resulting crude product was purified by column chromatography using an eluent mixture of *n*-pentane/EtOAc (6:4). Product **9** was obtained as colorless solid (167 mg, 0.289 mmol, 72%).

^{1}H-NMR (600 MHz, $CDCl_3$): δ = 7.46 (s, 1H), 7.40 (d, *J* = 15.8 Hz, 2H), 6.56 (s, 1H), 6.34 (d, *J* = 15.8 Hz, 2H), 5.45 (s, 1H), 4.79 (d, *J* = 2.5 Hz, 2H), 4.76 (d, *J* = 13.3 Hz, 1H), 4.46 (d, *J* = 12.4 Hz, 1H), 4.03 (s, 2H), 2.98 (dd, *J* = 16.8, 3.8 Hz, 1H), 2.86 (dd, *J* = 15.9, 14.5 Hz, 1H), 2.68 (dd, *J* = 16.7, 2.0 Hz, 1H), 2.54 (dd, *J* = 12.5, 2.9 Hz, 1H), 2.50 (t, *J* = 2.5 Hz, 1H), 2.47 (dd, *J* = 14.5, 3.4 Hz, 1H), 2.22 (dd, *J* = 15.9, 3.4 Hz, 1H), 1.95 – 1.86 (m, 1H), 1.86 – 1.75 (m, 2H), 1.57 – 1.49 (m, 2H), 1.29 (s, 3H), 1.18 (s, 3H), 1.16 (s, 3H), 1.07 (s, 3H) ppm.
^{13}C-NMR (151 MHz, $CDCl_3$): δ = 206.0, 169.0, 166.2, 165.8, 151.2, 143.1, 131.4, 122.8, 116.0, 114.3, 80.3, 79.1, 77.6, 77.4, 75.0, 65.5, 65.3, 60.6, 53.8, 52.1, 51.3, 48.1, 45.9, 37.9, 36.4, 35.6, 30.9, 30.2, 21.4, 20.7, 18.9, 17.6 ppm.
HRMS (ESI+NH_4^+) m/z: Calculated for $C_{32}H_{38}N_1O_{10}$ 596.24902, Found 596.24878.

(*E*)-(1-(3',6'-dihydroxy-3-oxo-*3H*-spiro[isobenzofuran-1,9'-xanthen]-6-yl)-*1H*-1,2,3-triazol-4-yl)methyl 3-(4-((4a*S*,6a*R*,8a*R*,8b*R*,9a*S*,12*S*,12a*S*,14b*R*)-6,6,8a,12a-tetramethyl-3,8,10-trioxohexadecahydrooxireno[2,3-*d*]pyrano[4',3':3,3a]isobenzofuro[5,4-*f*]isochromen-12-yl)furan-2-yl)acrylate (11)

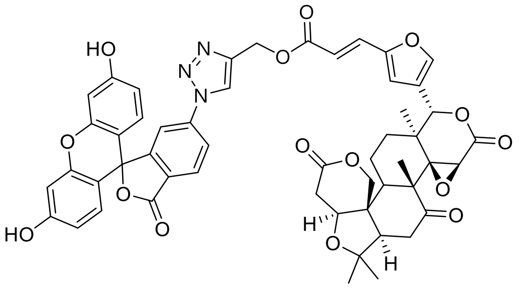

11

Substrate **9** (17.7 mg, 30.6 µmol) and azide **10** (17.2 mg, 46.0 µmol, 1.5 equiv) were charged in an oven dried 10 mL Schlenk tube. Copper(II) sulfate tetrahydrate (3.10 mg, 12.4 µmol, 0.4 equiv) and sodium ascorbate (1.20 mg, 6.06 µmol, 0.2 equiv) were added from a freshly prepared stock solution and all components were dissolved in a H_2O/DMSO mixture (1:1, 0.4 mL). The reaction mixture was stirred at 60°C for 24 h, diluted with EtOAc (5 mL) and the solvent was evaporated under reduced pressure. The crude compound was further dried over a nitrogen flow until a solid formed. Purification by column chromatography (CH_2Cl_2:EtOAc:MeOH:HCOOH 4:4:0.5:0.5) gave the target compound as an orange solid (19 mg, 20 µmol, 65%).

^{1}H-NMR (600 MHz, DMSO): δ = 10.29 (s, 2H), 9.18 (s, 1H), 8.49 (dd, *J* = 8.4, 1.9 Hz, 1H), 8.35 (dd, *J* = 8.4, 0.6 Hz, 1H), 8.03 (s, 2H), 7.59 (d, *J* = 15.8 Hz, 1H), 7.16 (s, 1H), 6.85 – 6.79 (m, 4H), 6.70 (dd, *J* = 8.7, 2.4 Hz, 2H), 6.38 (d, *J* = 15.9 Hz, 1H), 5.62 (s, 1H), 5.43 (s, 2H), 5.06 (d, *J* = 13.1 Hz, 1H), 4.62 (d, *J* = 13.0 Hz, 1H), 4.27 (s, 1H), 4.24 (d, *J* = 3.8 Hz, 1H), 3.26 (t, *J* = 15.3 Hz, 1H), 2.90 (d, *J* = 16.3 Hz, 1H), 2.76 (dd, *J* = 16.6, 4.0 Hz, 1H), 2.66 (m, 1H), 2.61 – 2.57 (m, 1H), 2.41 (dd, *J* = 14.8, 3.3 Hz, 1H), 1.98 (d, *J* = 7.0 Hz, 1H), 1.85 (dd, *J* = 12.7, 8.1 Hz, 2H), 1.42 (m, 1H), 1.32 (s, 3H), 1.22 (s, 3H), 1.16 (s, 3H), 1.14 (s, 3H) ppm.

^{13}C-NMR (151 MHz, DMSO): δ = 208.5, 170.7, 168.1, 167.6, 165.9, 160.2, 152.4 150.7, 145.1, 144.1, 142.0, 132.1, 130.1, 129.7, 129.1, 127.3, 126.4, 123.9, 123.6, 122.2, 116.6, 115.2, 113.2, 109.5, 102.8, 80.0, 78.9, 77.5, 67.2, 65.3, 58.4, 57.5, 55.3, 54.2, 50.7, 46.9, 45.7, 40.5, 38.1, 36.7, 36.2, 35.6, 31.7, 30.2, 29.5, 27.1, 25.6, 22.6, 21.9, 20.0, 18.0, 17.5 ppm.

HRMS (ESI+NH_4^+) m/z: Calculated for $C_{52}H_{46}N_3O_{15}$ 952.29234, Found 952.29156.

7. NMR Spectra

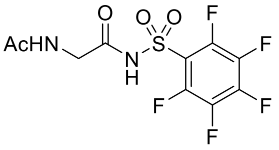

L2b

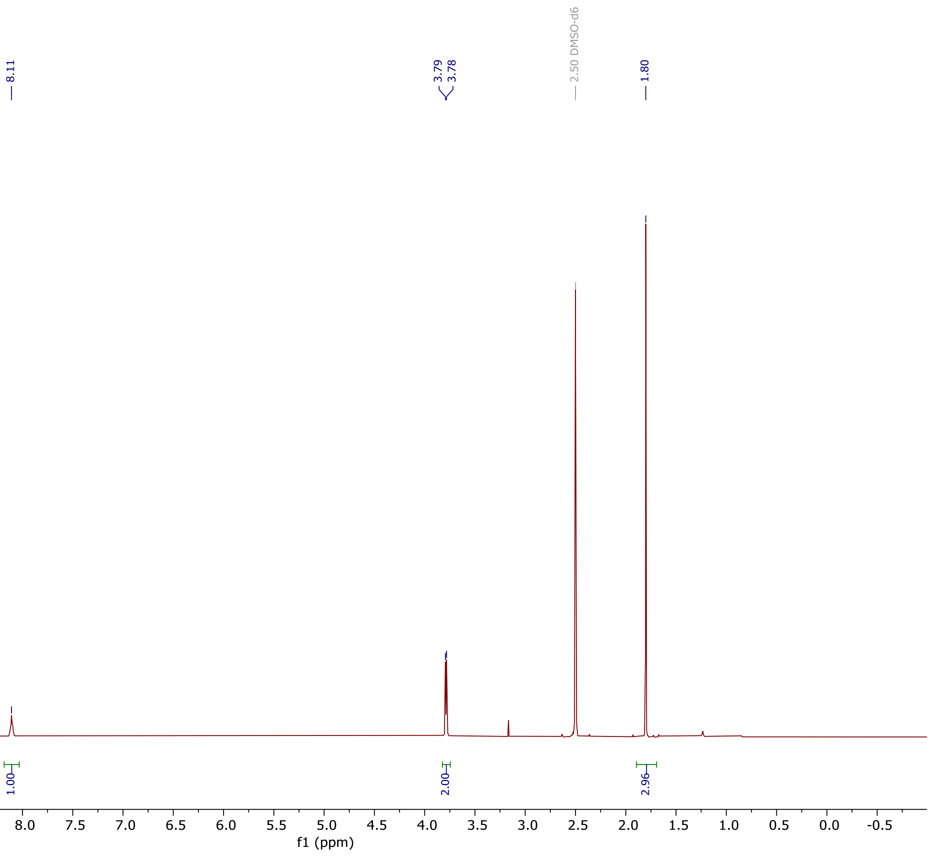

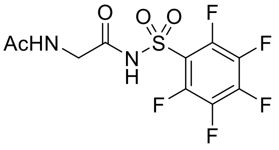

L2b

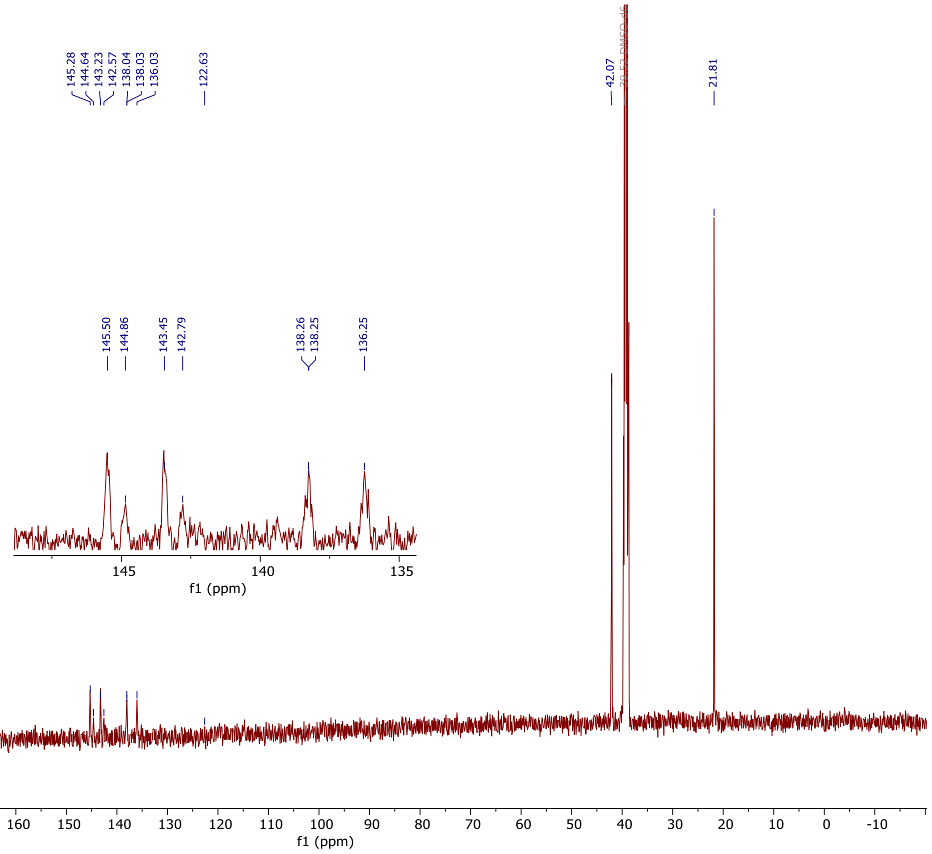

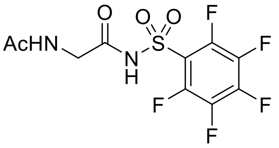

L2b

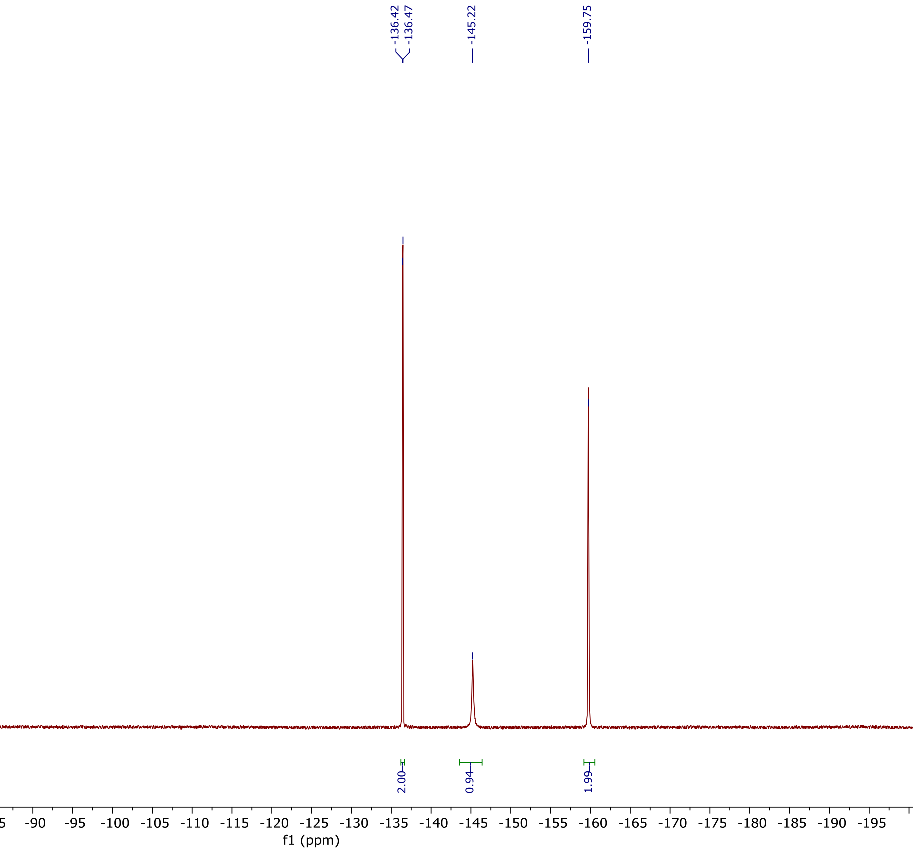
-136.42
-136.47
-145.22
-159.75
2.00
0.94
1.99
-50 -55 -60 -65 -70 -75 -80 -85 -90 -95 -100 -105 -110 -115 -120 -125 -130 -135 -140 -145 -150 -155 -160 -165 -170 -175 -180 -185 -190 -195
f1 (ppm)

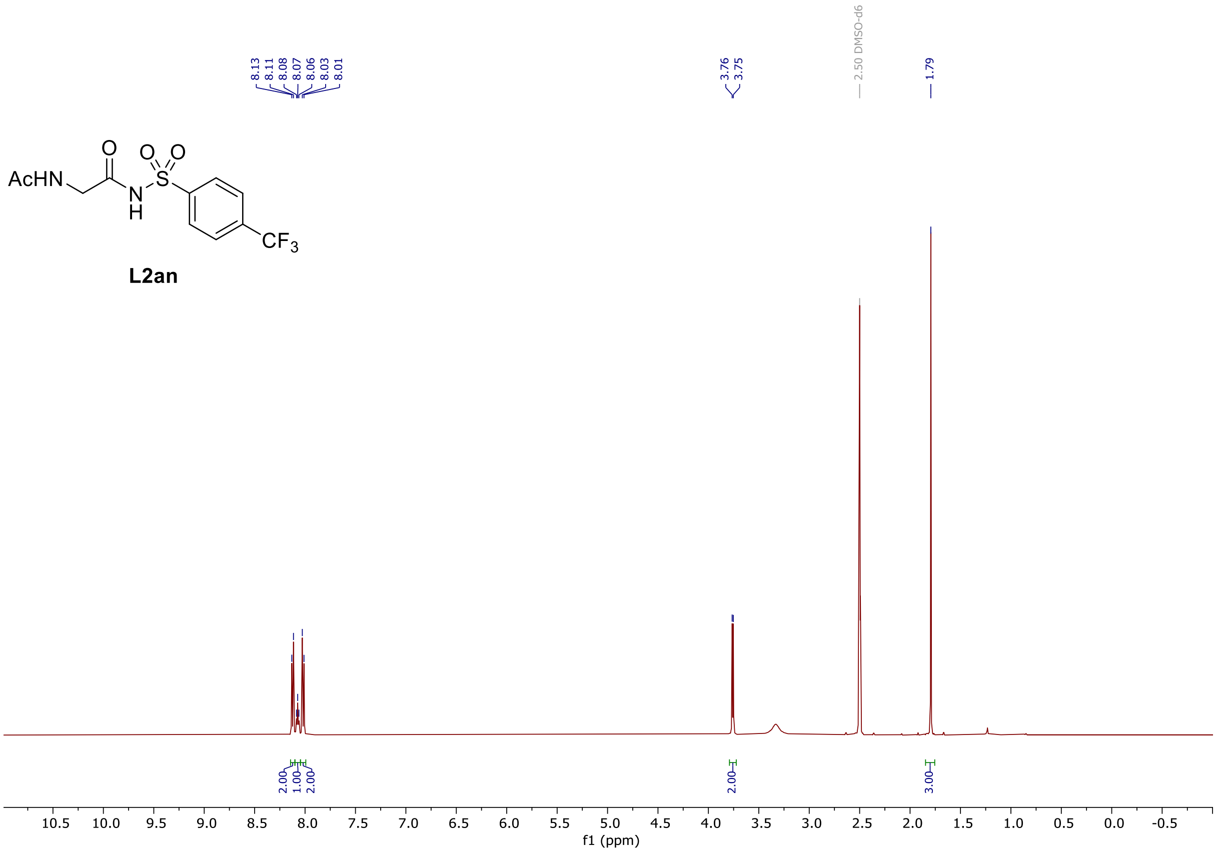
8.13
8.11
8.08
8.07
8.06
8.03
8.01
3.76
3.75
2.50 DMSO-d6
1.79
AcHN
O
O
O
N
S
H
CF3
L2an
2.00
1.00
2.00
2.00
3.00
f1 (ppm)

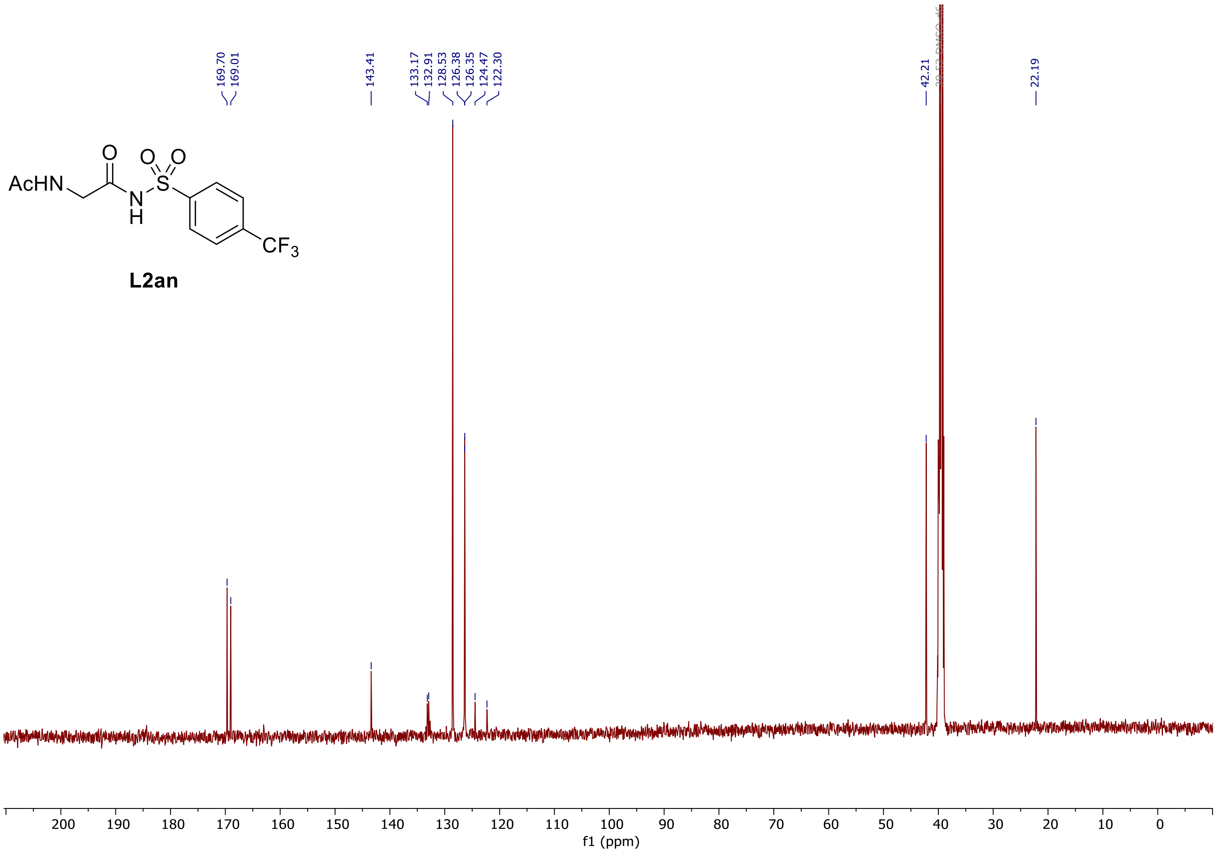
169.70
169.01
143.41
133.17
132.91
128.53
126.38
126.35
124.47
122.30
42.21
22.19
AcHN
O
O
O
N
S
H
CF3
L2an
f1 (ppm)

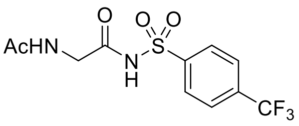

L2an

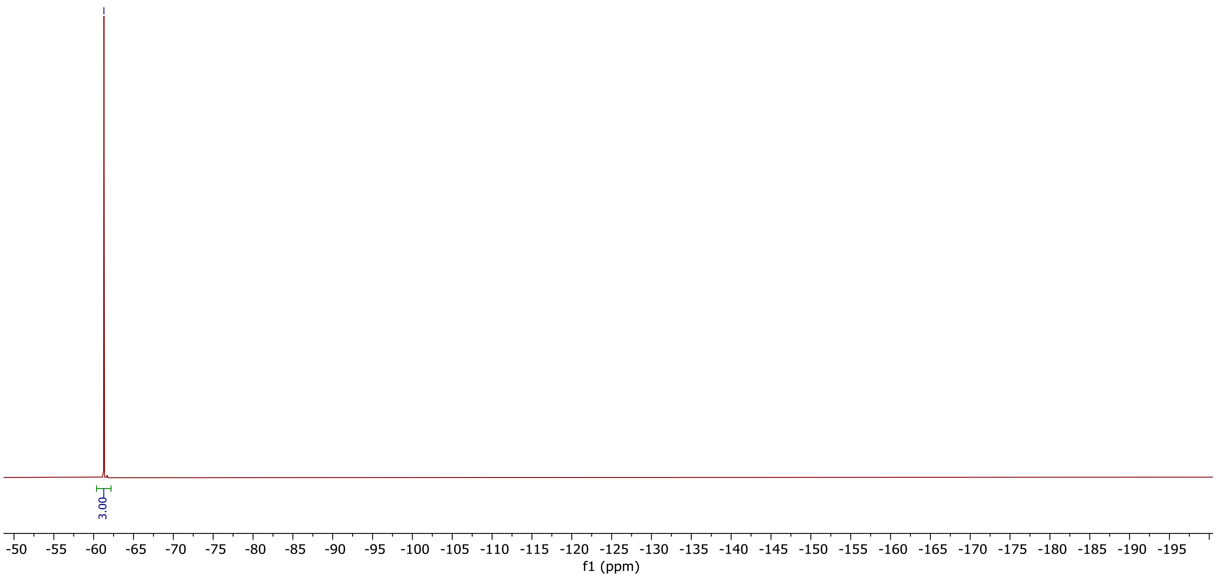
-61.28
3.00
-50 -55 -60 -65 -70 -75 -80 -85 -90 -95 -100 -105 -110 -115 -120 -125 -130 -135 -140 -145 -150 -155 -160 -165 -170 -175 -180 -185 -190 -195
f1 (ppm)

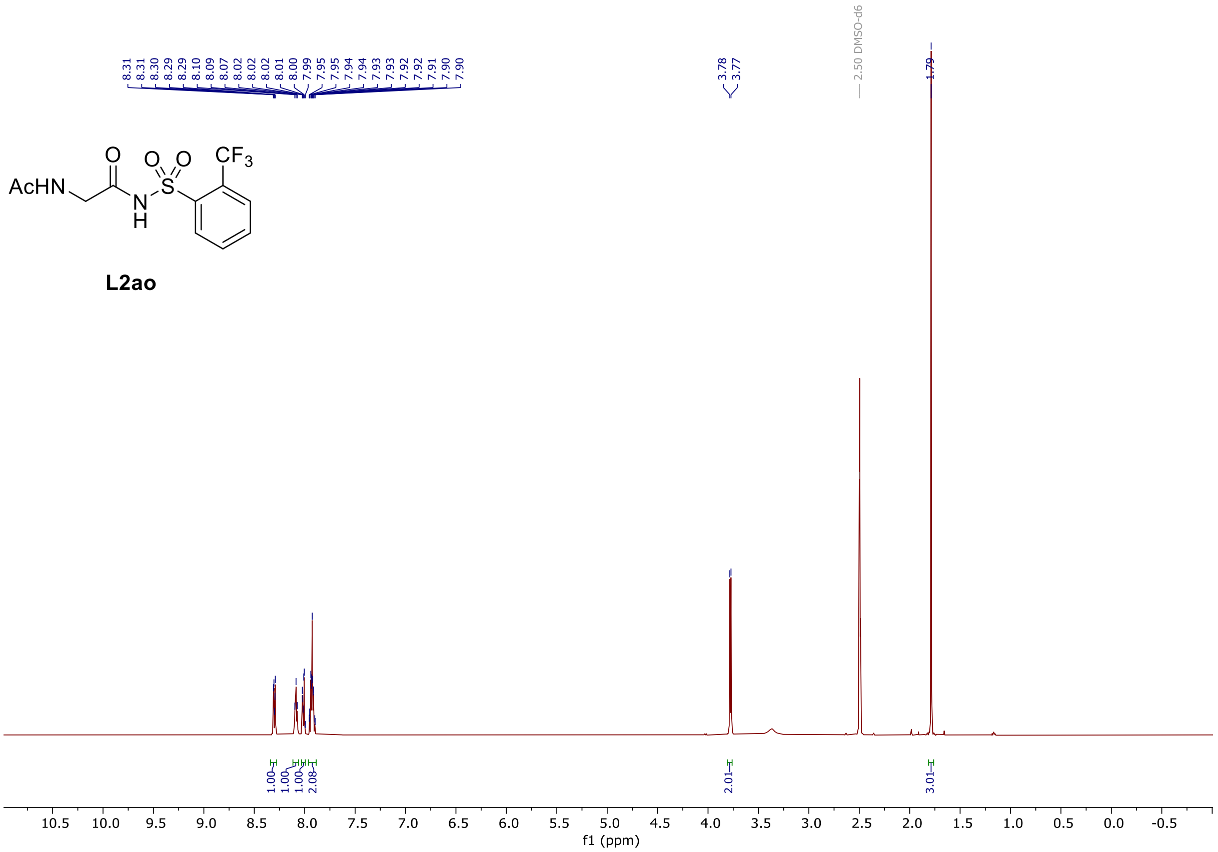
8.31 8.31 8.30 8.29 8.29 8.10 8.09 8.07 8.02 8.02 8.02 8.01 8.00 7.99 7.95 7.95 7.94 7.94 7.93 7.93 7.92 7.92 7.91 7.90 7.90
3.78 3.77
2.50 DMSO-d6
1.79
AcHN
O
O O
S
CF3
N
H
L2ao
1.00
1.00
1.00
2.08
2.01
3.01
f1 (ppm)

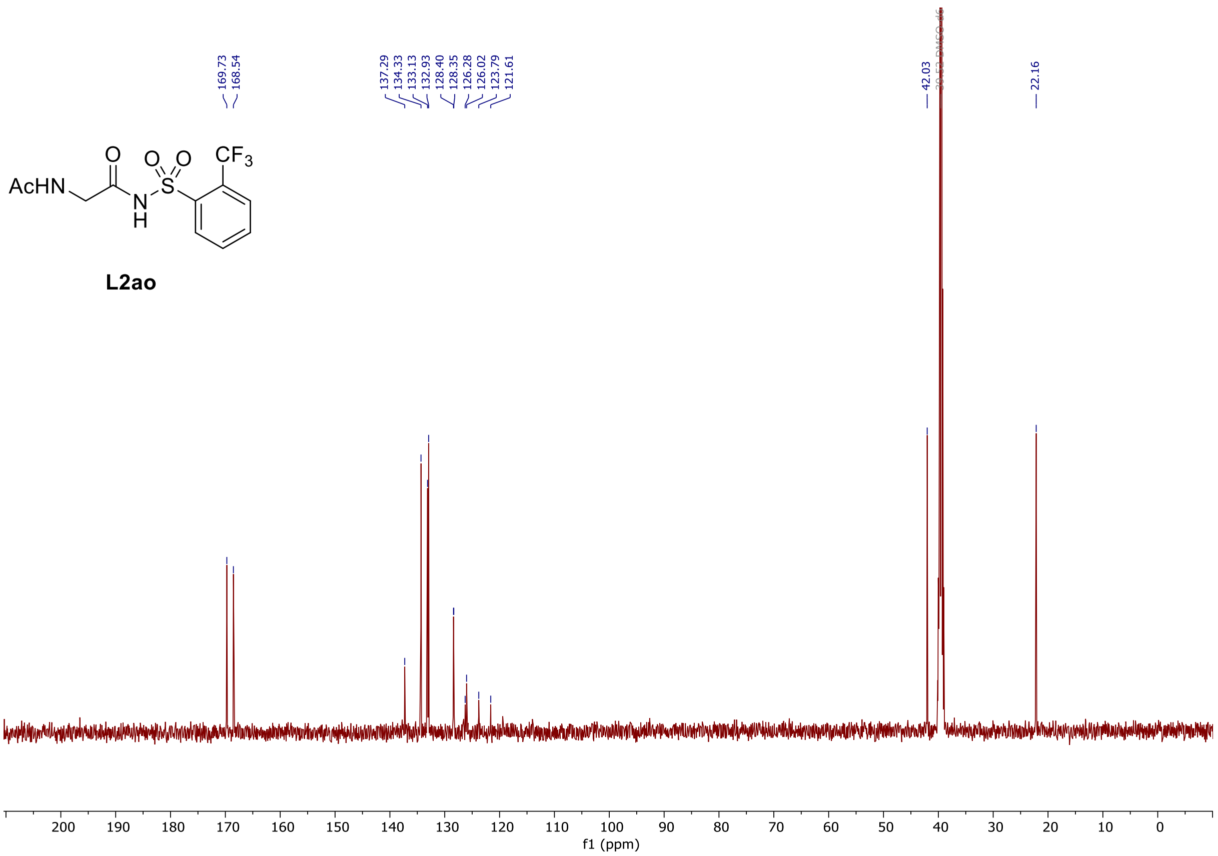
169.73 168.54
137.29 134.33 133.13 132.93 128.40 128.35 126.28 126.02 123.79 121.61
42.03
22.16
AcHN
O
O O
S
CF3
N
H
L2ao
f1 (ppm)

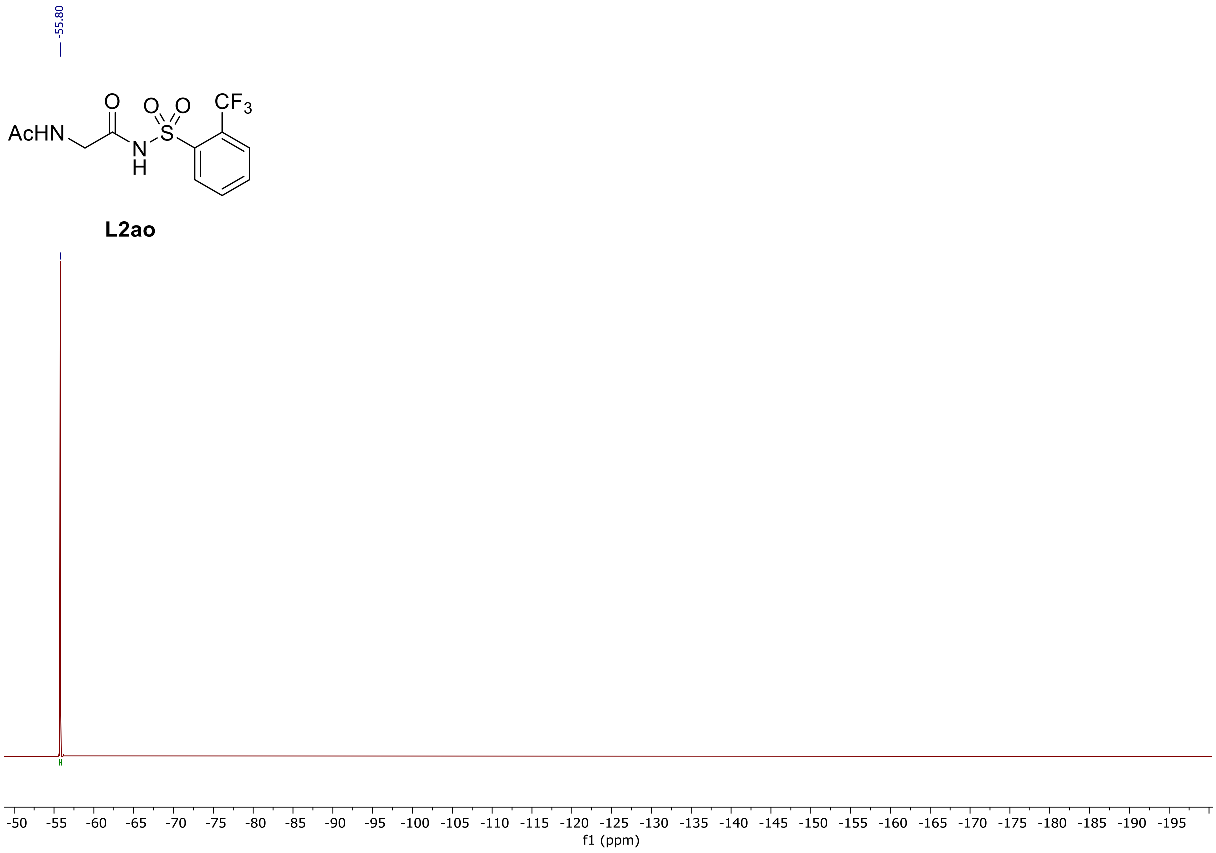

-55.80
AcHN
O
O
O
S
N
H
CF3
-50
-55
-60
-65
-70
-75
-80
-85
-90
-95
-100
-105
-110
-115
-120
-125
-130
-135
-140
-145
-150
-155
-160
-165
-170
-175
-180
-185
-190
-195
f1 (ppm)

L2ao

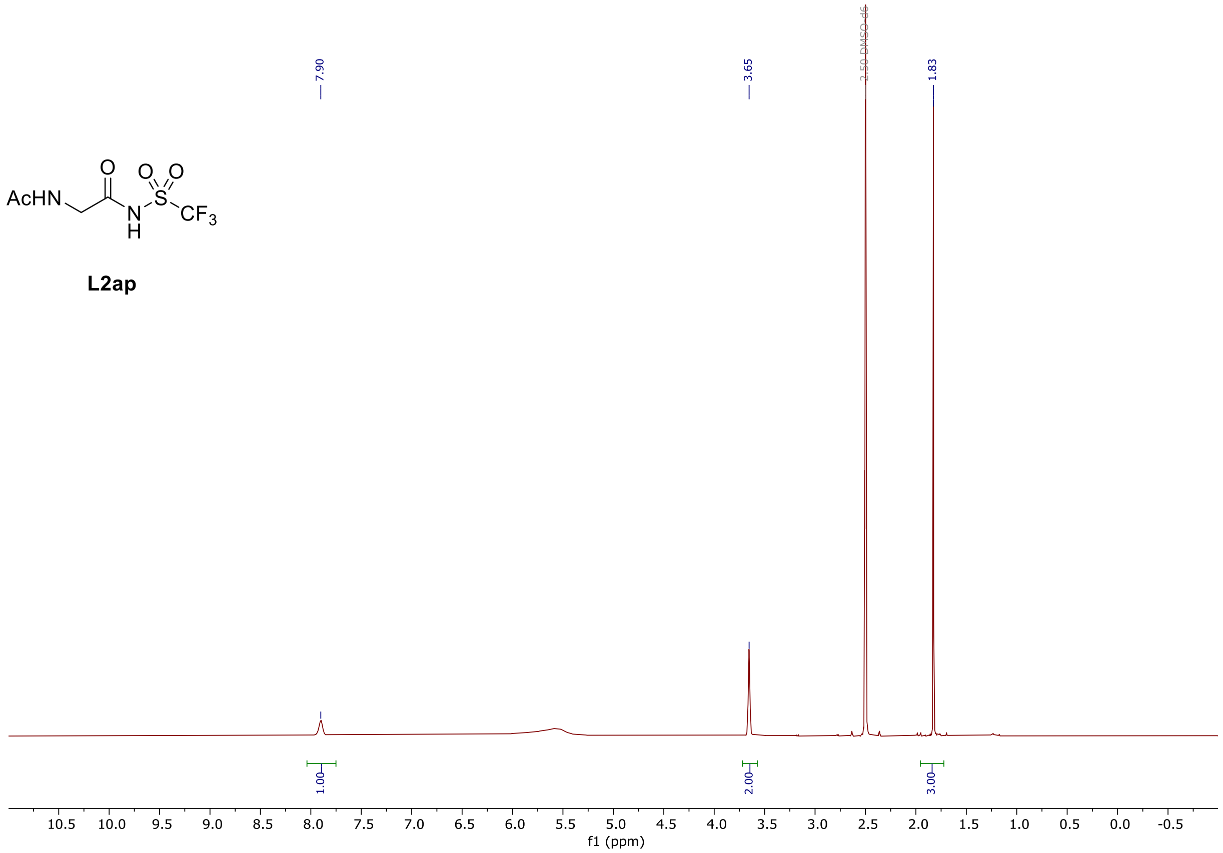
AcHN
O
O O
S
N
H
CF3
L2ap
7.90
3.65
2.50 DMSO-d6
1.83
1.00
2.00
3.00
10.5 10.0 9.5 9.0 8.5 8.0 7.5 7.0 6.5 6.0 5.5 5.0 4.5 4.0 3.5 3.0 2.5 2.0 1.5 1.0 0.5 0.0 -0.5
f1 (ppm)

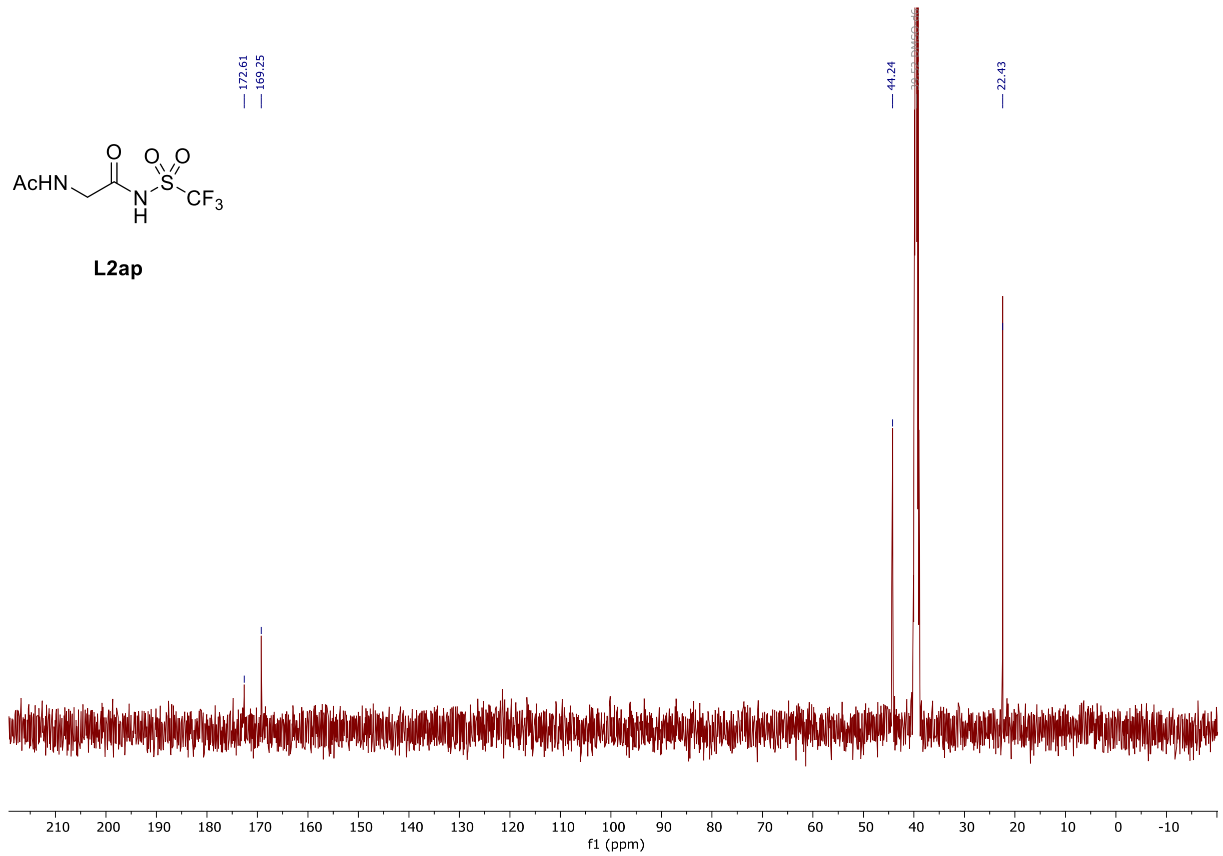
AcHN
O
O O
S
N
H
CF3
L2ap
172.61
169.25
44.24
22.43
210 200 190 180 170 160 150 140 130 120 110 100 90 80 70 60 50 40 30 20 10 0 -10
f1 (ppm)

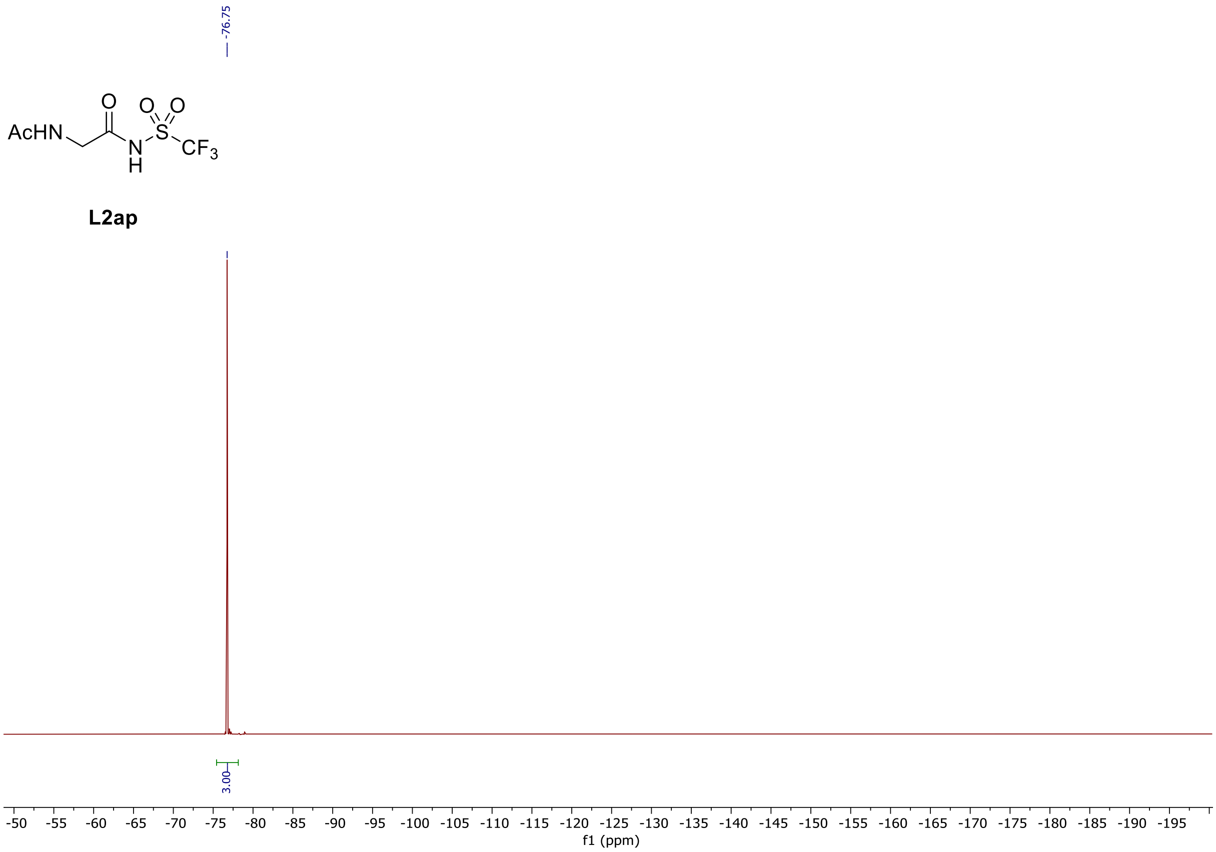
-76.75
AcHN
O
O
O
S
N
H
CF3
L2ap
3.00
-50 -55 -60 -65 -70 -75 -80 -85 -90 -95 -100 -105 -110 -115 -120 -125 -130 -135 -140 -145 -150 -155 -160 -165 -170 -175 -180 -185 -190 -195
f1 (ppm)

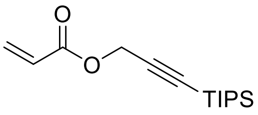
O
O
TIPS

2

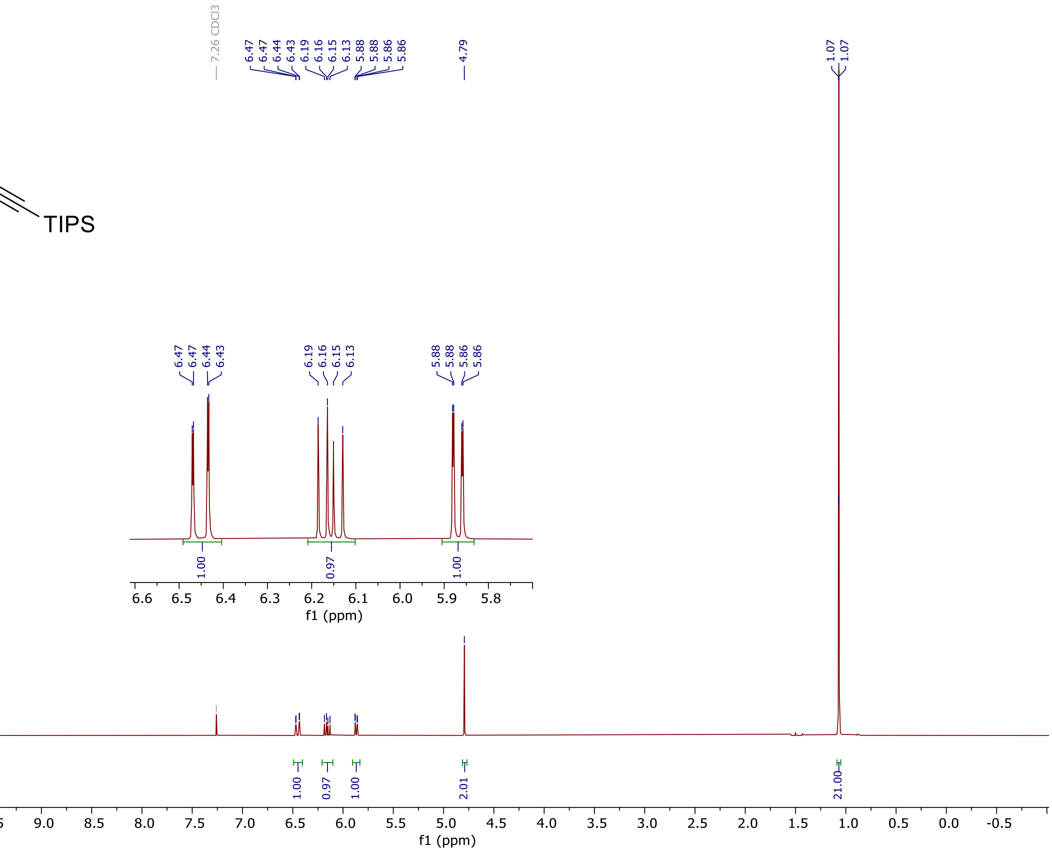
7.26 CDCl3
6.47
6.47
6.44
6.43
6.19
6.16
6.15
6.13
5.88
5.88
5.86
5.86
4.79
1.07
1.07
1.00
0.97
1.00
2.01
21.00
f1 (ppm)

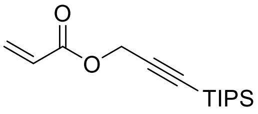
O
O
TIPS

2

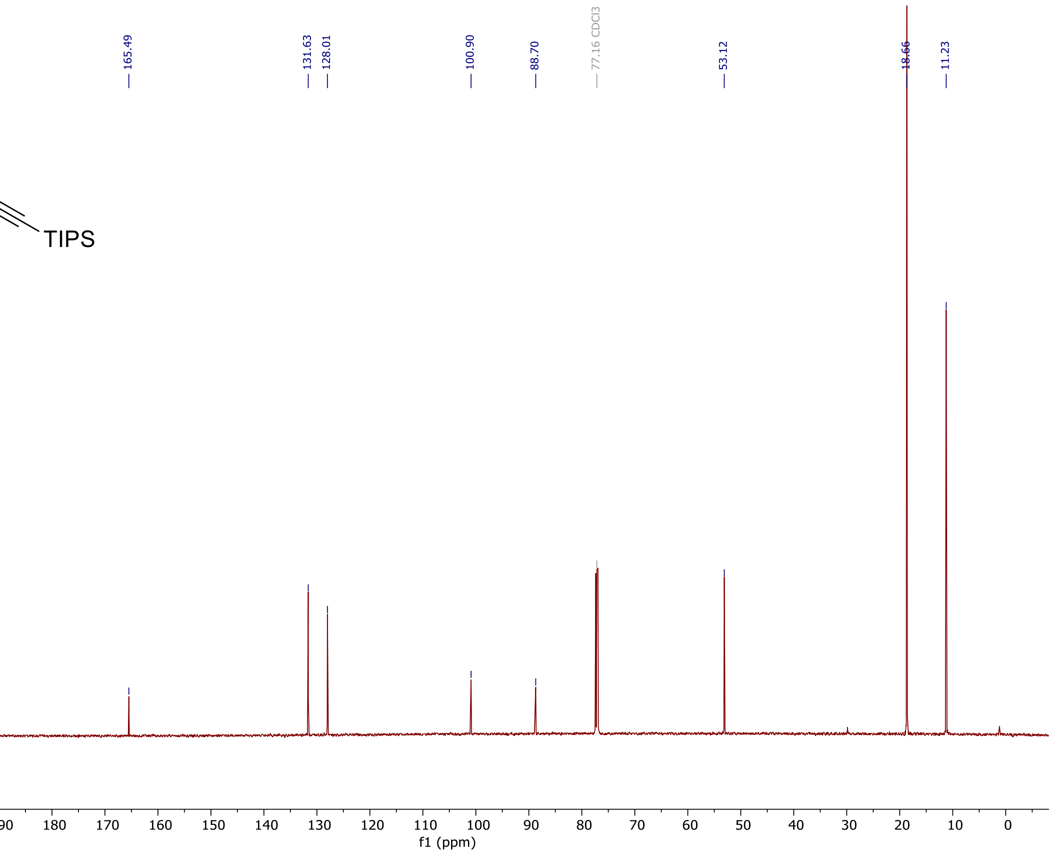
165.49
131.63
128.01
100.90
88.70
77.16 CDCl3
53.12
18.66
11.23
f1 (ppm)

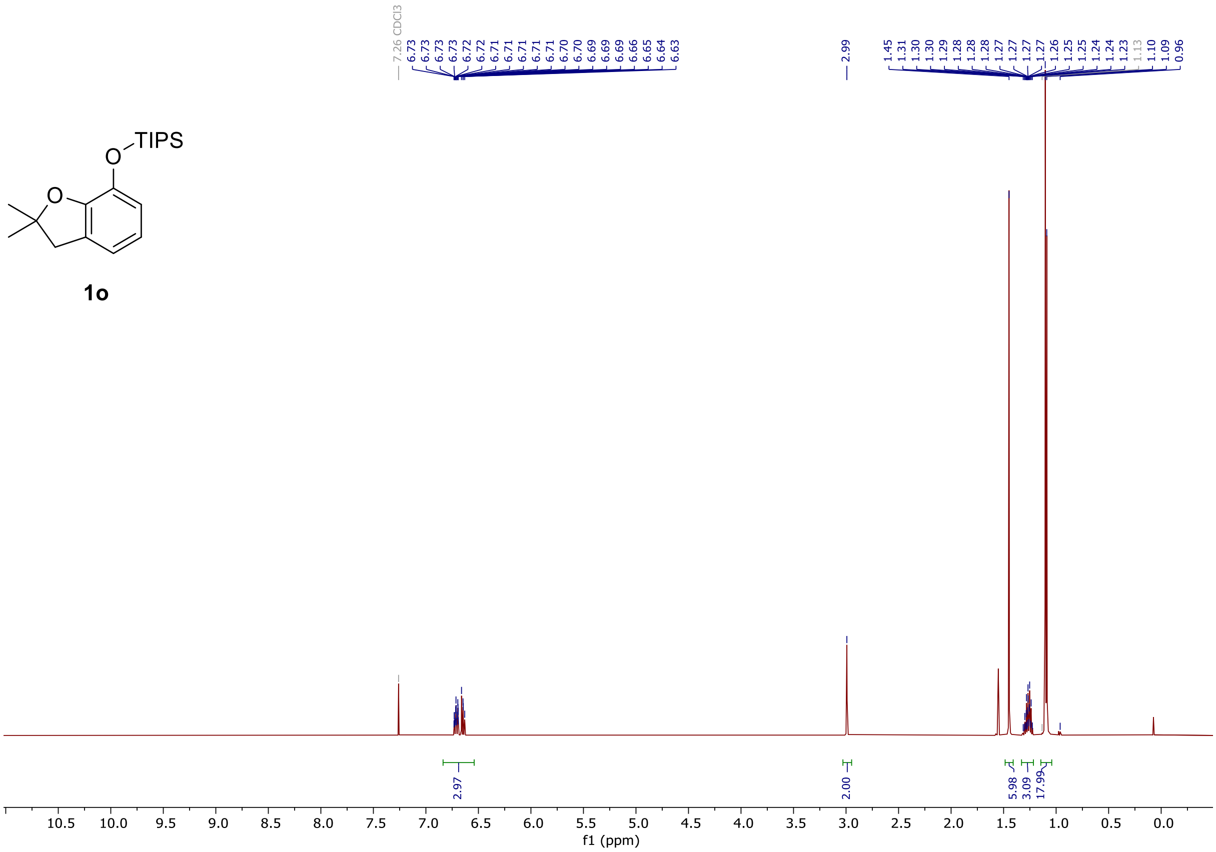

O-TIPS
1o
7.26 CDCl3
6.73
6.73
6.73
6.72
6.72
6.71
6.71
6.71
6.71
6.70
6.69
6.69
6.69
6.66
6.65
6.64
6.63
2.99
1.45
1.31
1.30
1.30
1.29
1.28
1.28
1.27
1.27
1.27
1.27
1.26
1.25
1.25
1.24
1.24
1.23
1.13
1.10
1.09
0.96
2.97
2.00
5.98
3.09
17.99
f1 (ppm)

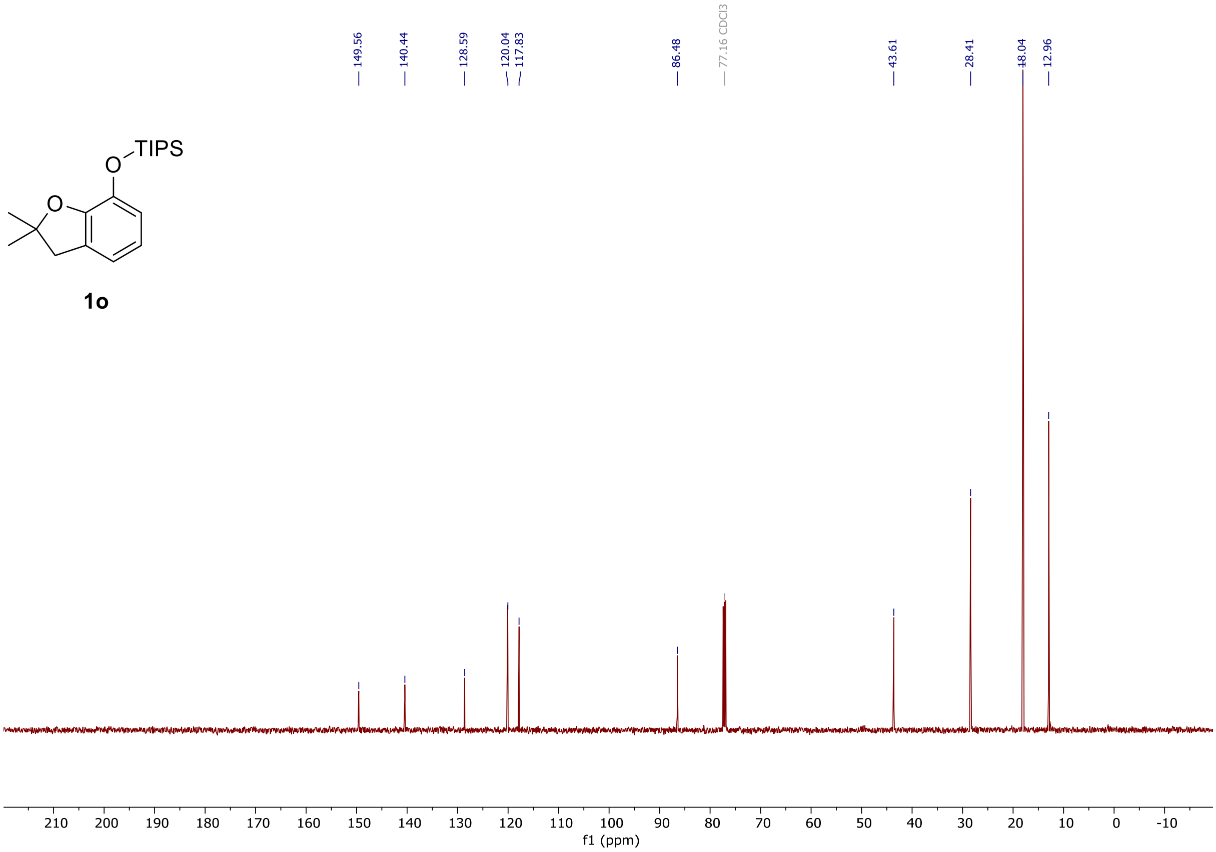

O-TIPS
1o
149.56
140.44
128.59
120.04
117.83
86.48
77.16 CDCl3
43.61
28.41
18.04
12.96
f1 (ppm)

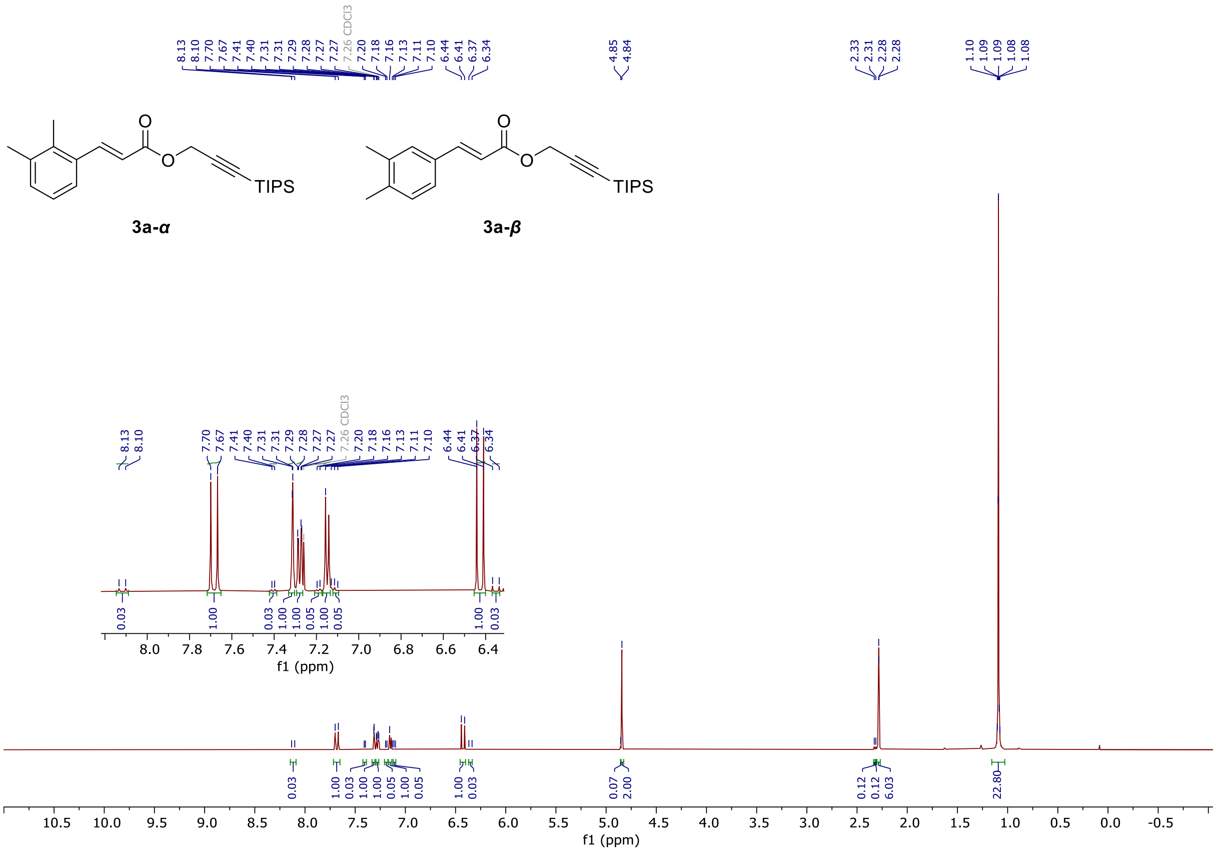
8.13 8.10 7.70 7.67 7.41 7.40 7.31 7.31 7.29 7.28 7.27 7.27 7.26 CDCl3 7.20 7.18 7.16 7.13 7.11 7.10 6.44 6.41 6.37 6.34
4.85 4.84
2.33 2.31 2.28 2.28
1.10 1.09 1.08 1.08
O
O
TIPS
3a-α
3a-β
0.03 1.00 0.03 1.00 1.00 0.05 1.00 0.05 1.00 0.03
0.07 2.00
0.12 0.12 6.03
22.80
f1 (ppm)

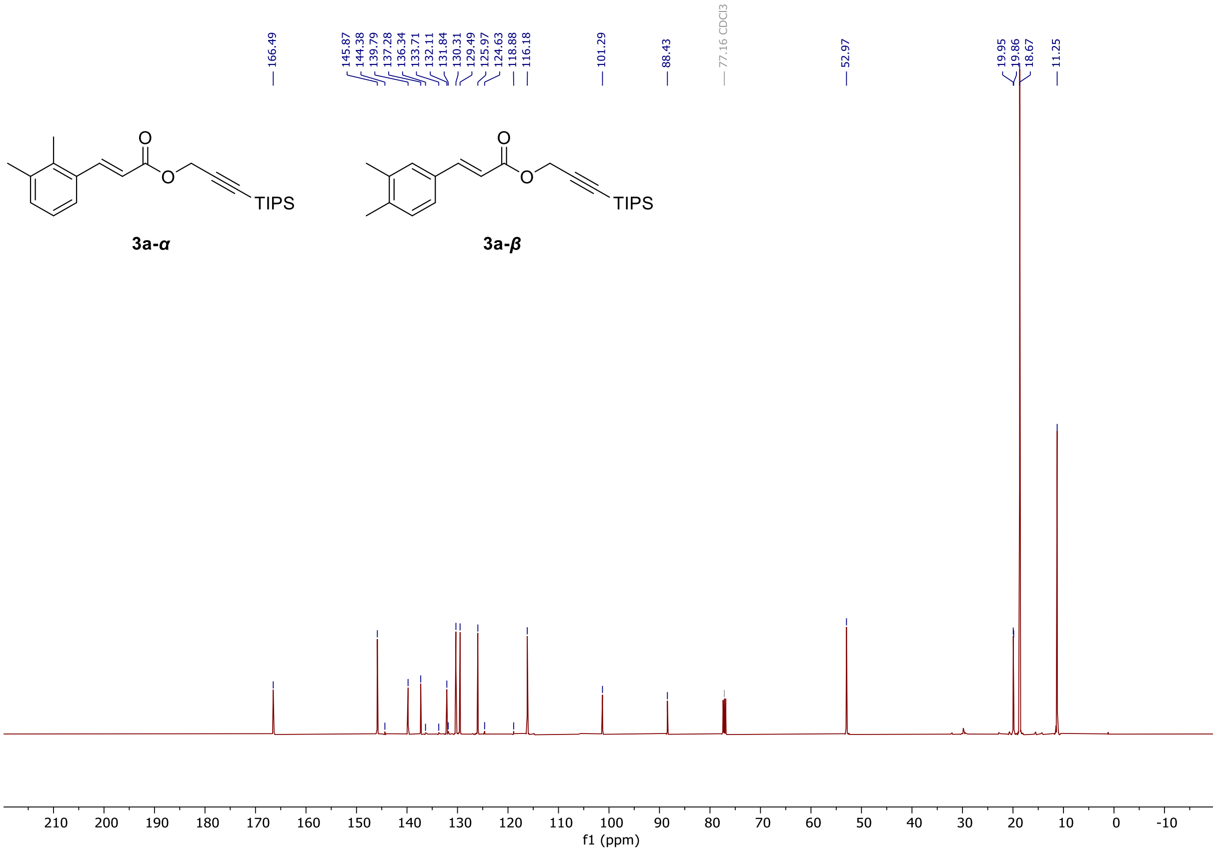
166.49
145.87 144.38 139.79 137.28 136.34 133.71 132.11 131.84 130.31 129.49 125.97 124.63 118.88 116.18
101.29
88.43
77.16 CDCl3
52.97
19.95 19.86 18.67
11.25
O
O
TIPS
3a-α
3a-β
f1 (ppm)

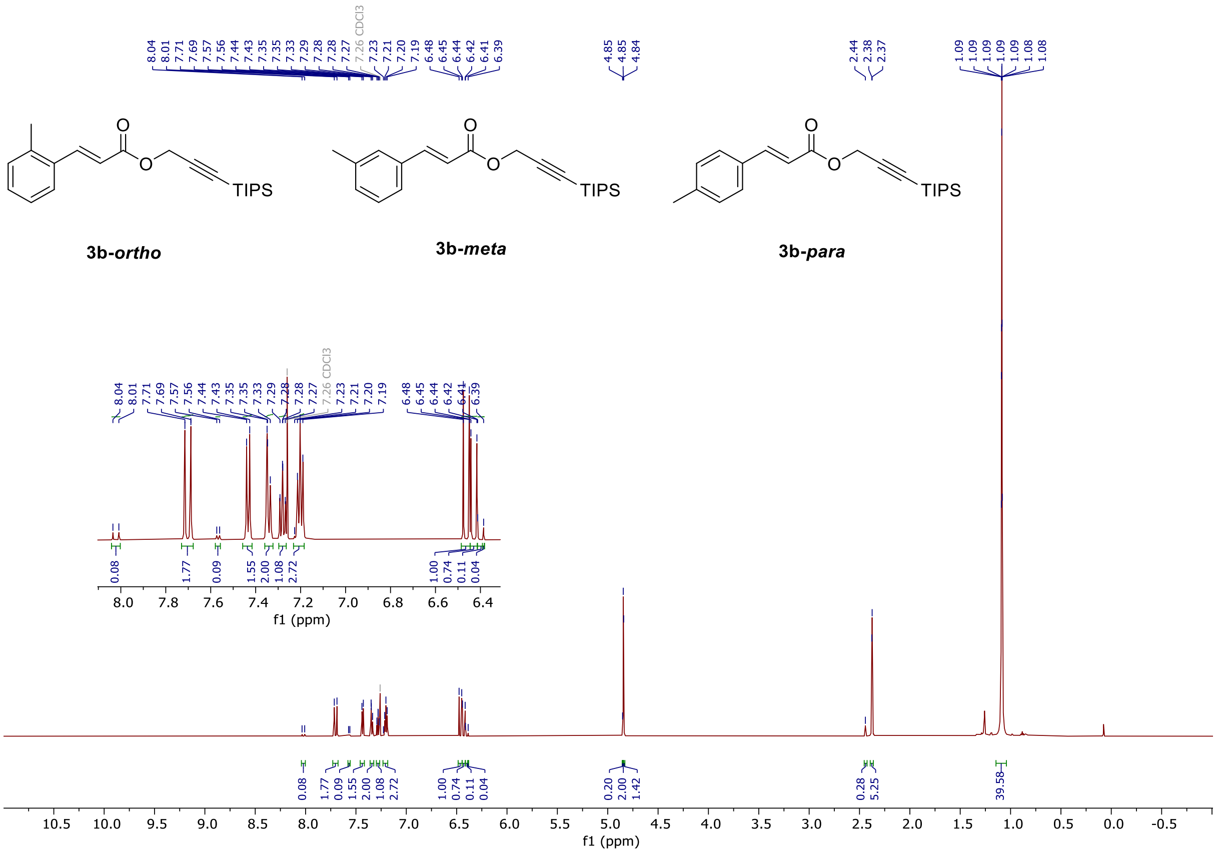
TIPS
3b-*ortho*
3b-*meta*
3b-*para*
f1 (ppm)

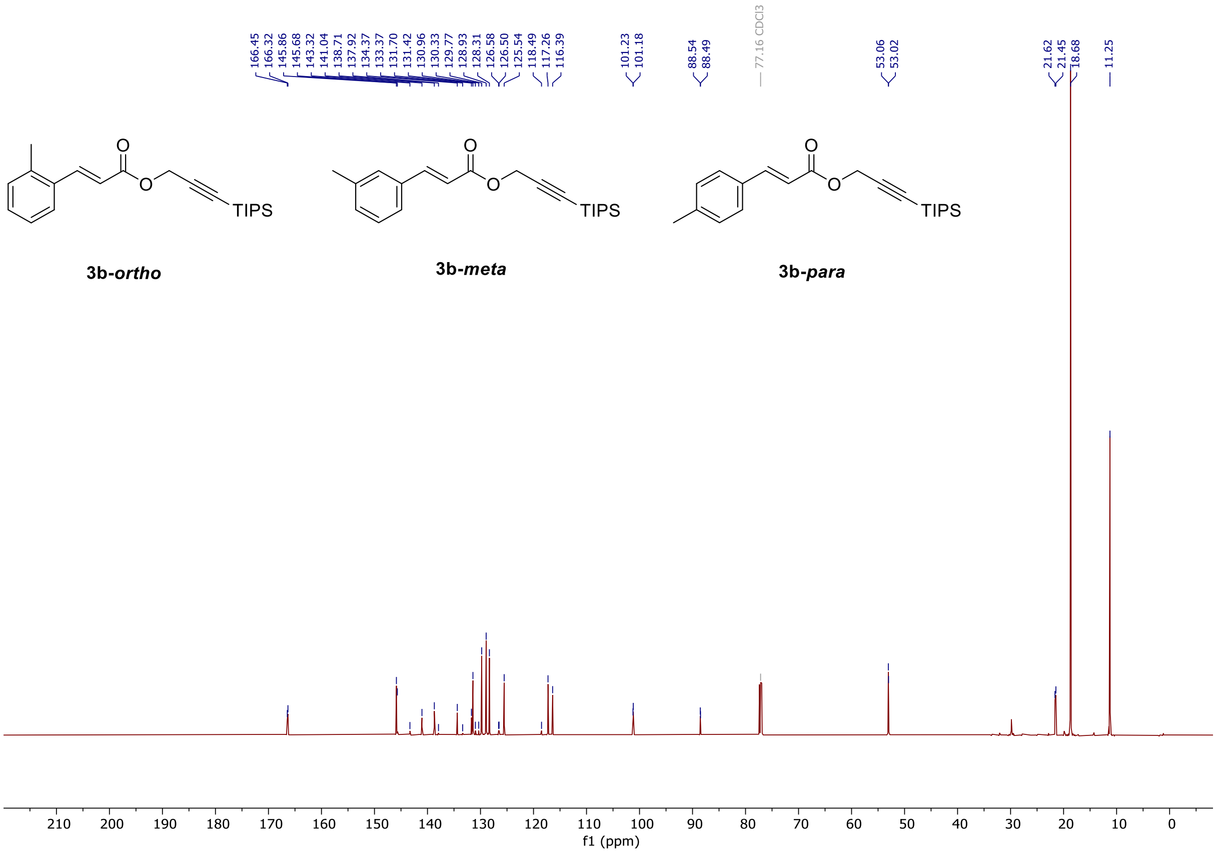
TIPS
3b-*ortho*
3b-*meta*
3b-*para*
f1 (ppm)

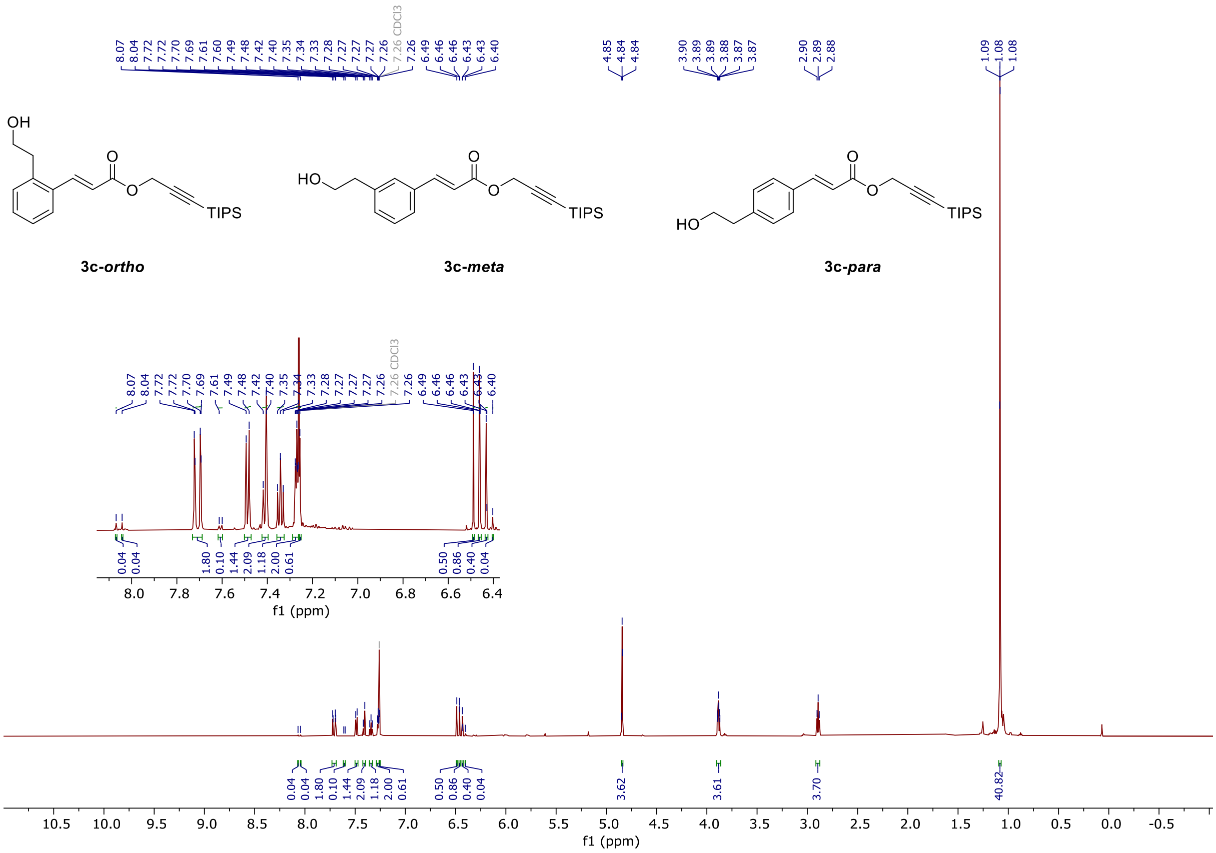
3c-ortho
3c-meta
3c-para
f1 (ppm)

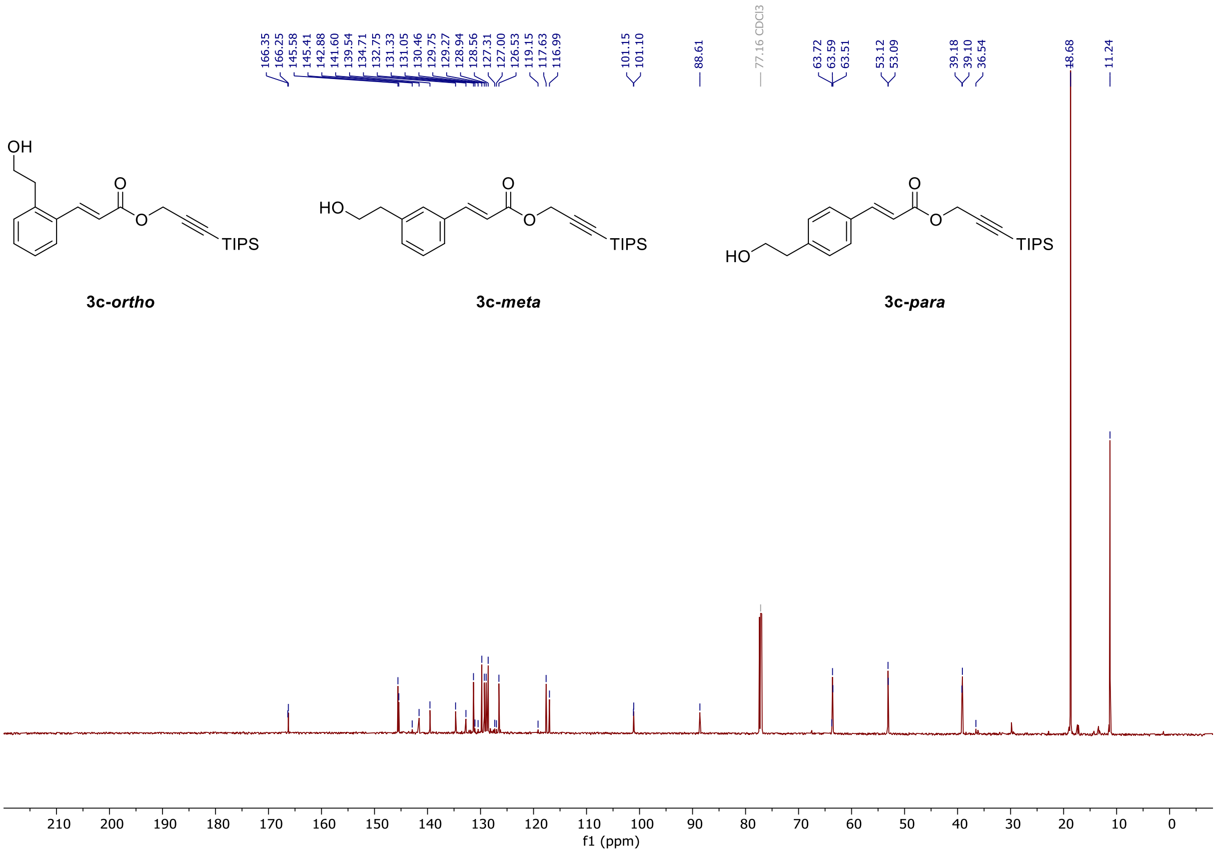
3c-ortho
3c-meta
3c-para
f1 (ppm)

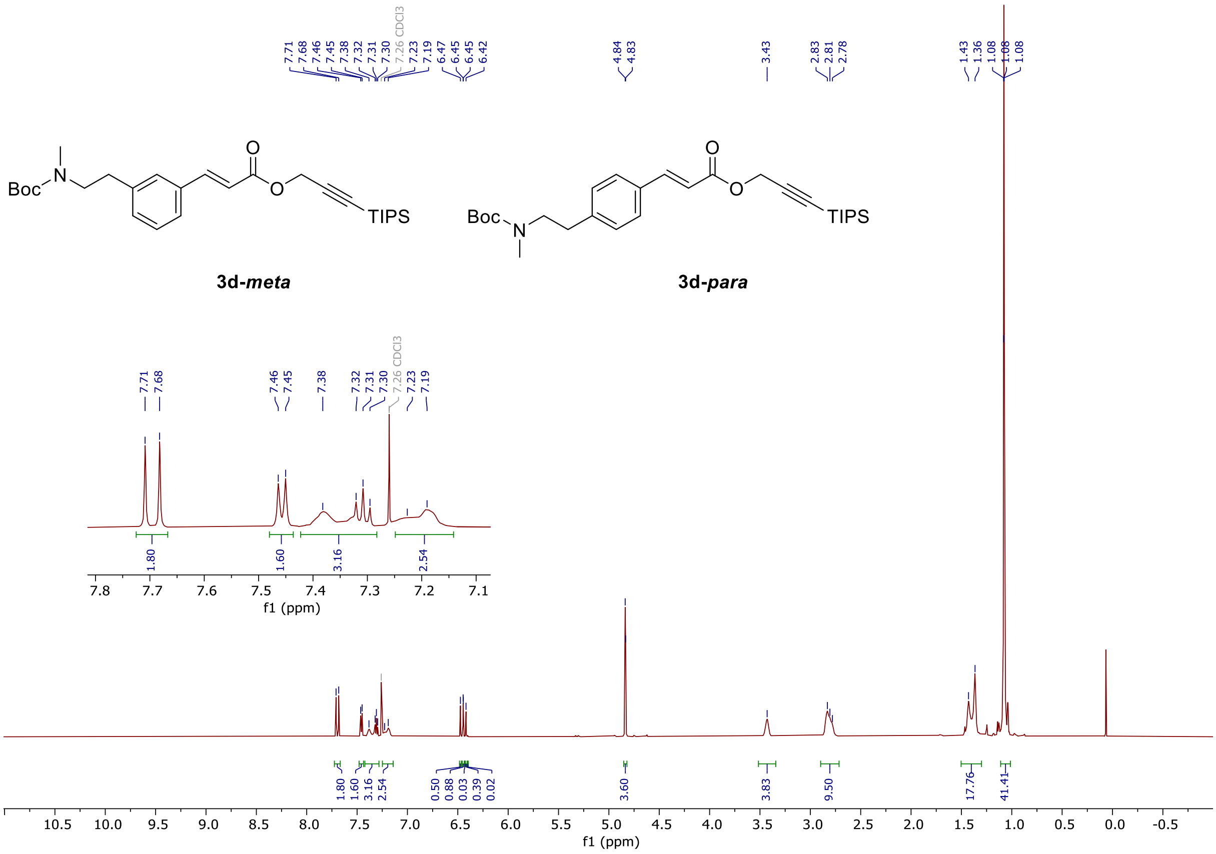
7.71 7.68 7.46 7.45 7.38 7.32 7.31 7.30 7.26 CDCl3 7.23 7.19 6.47 6.45 6.45 6.42
4.84 4.83
3.43
2.83 2.81 2.78
1.43 1.36 1.08 1.08 1.08
Boc N
O
O
TIPS
3d-*meta*
3d-*para*
1.80 1.60 3.16 2.54
7.8 7.7 7.6 7.5 7.4 7.3 7.2 7.1
f1 (ppm)
1.80 1.60 3.16 2.54 0.50 0.88 0.03 0.39 0.02 3.60 3.83 9.50 17.76 41.41
10.5 10.0 9.5 9.0 8.5 8.0 7.5 7.0 6.5 6.0 5.5 5.0 4.5 4.0 3.5 3.0 2.5 2.0 1.5 1.0 0.5 0.0 -0.5
f1 (ppm)

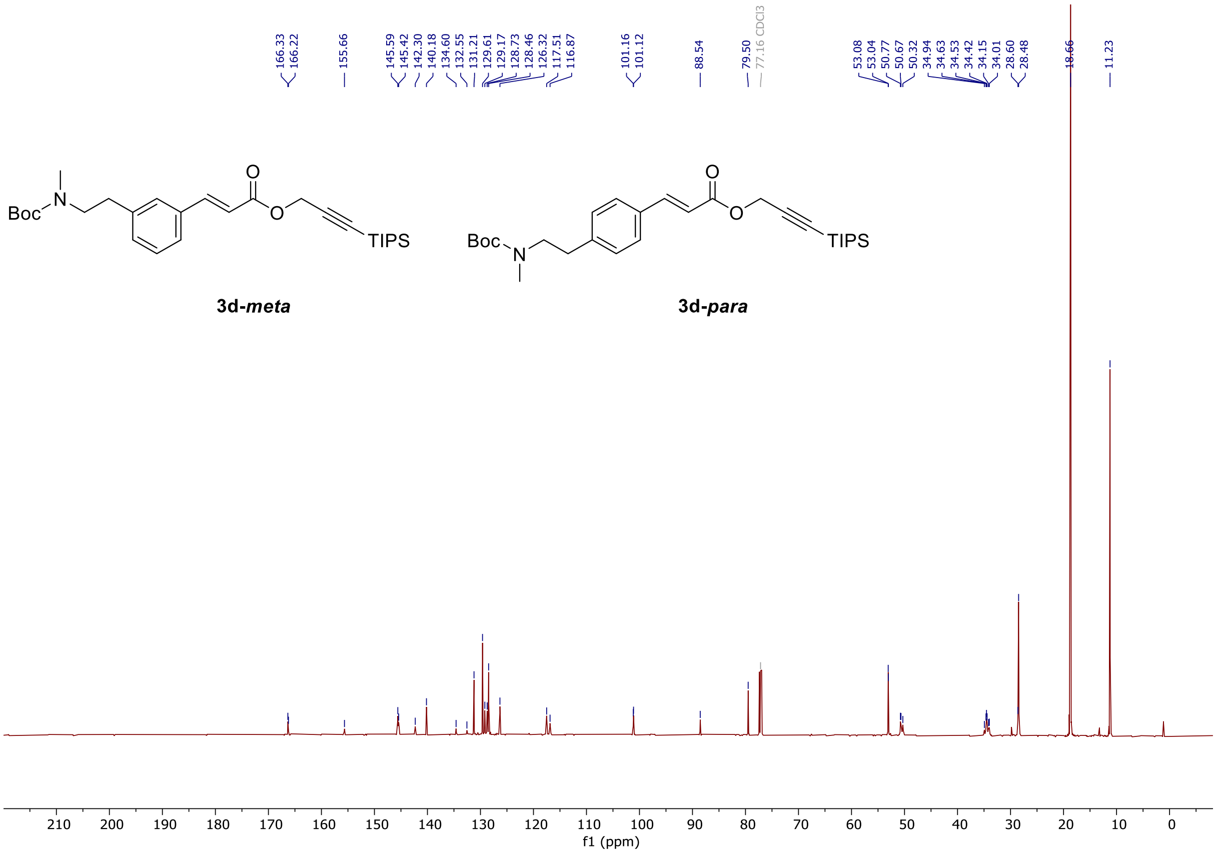
166.33 166.22
155.66
145.59 145.42 142.30 140.18 134.60 132.55 131.21 129.61 129.17 128.73 128.46 126.32 117.51 116.87
101.16 101.12
88.54
79.50 77.16 CDCl3
53.08 53.04 50.77 50.67 50.32 34.94 34.63 34.53 34.42 34.15 34.01 28.60 28.48
18.66
11.23
Boc N
O
O
TIPS
3d-*meta*
3d-*para*
210 200 190 180 170 160 150 140 130 120 110 100 90 80 70 60 50 40 30 20 10 0
f1 (ppm)

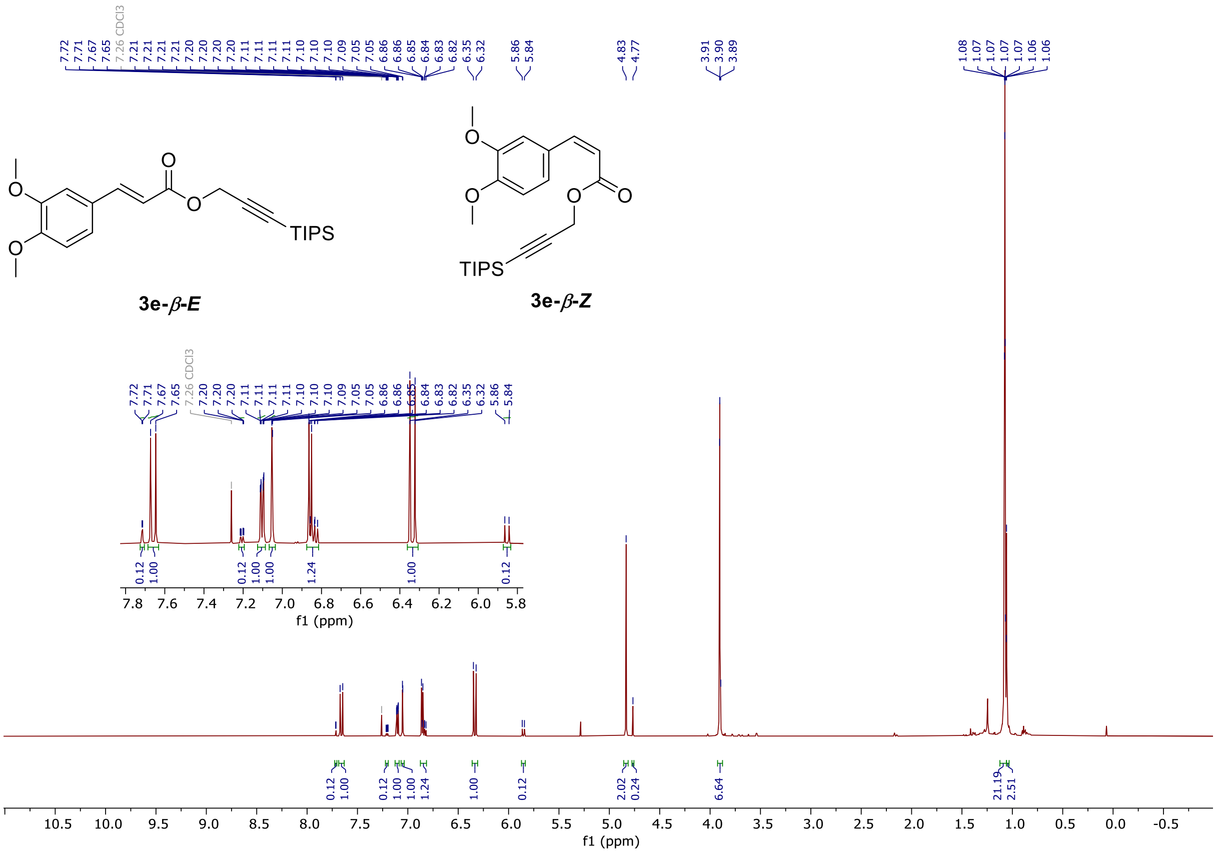
TIPS
3e-β-E
TIPS
3e-β-Z
f1 (ppm)
f1 (ppm)

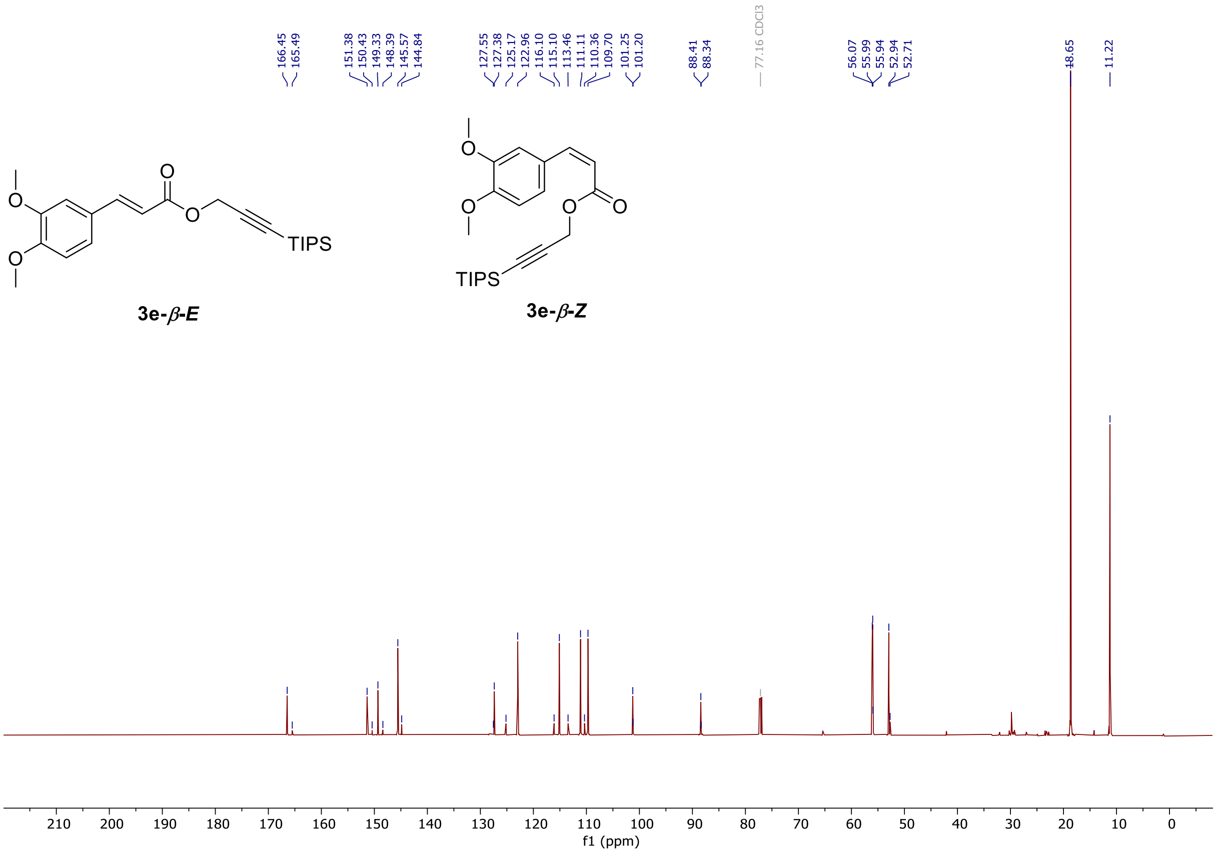
TIPS
3e-β-E
TIPS
3e-β-Z
f1 (ppm)

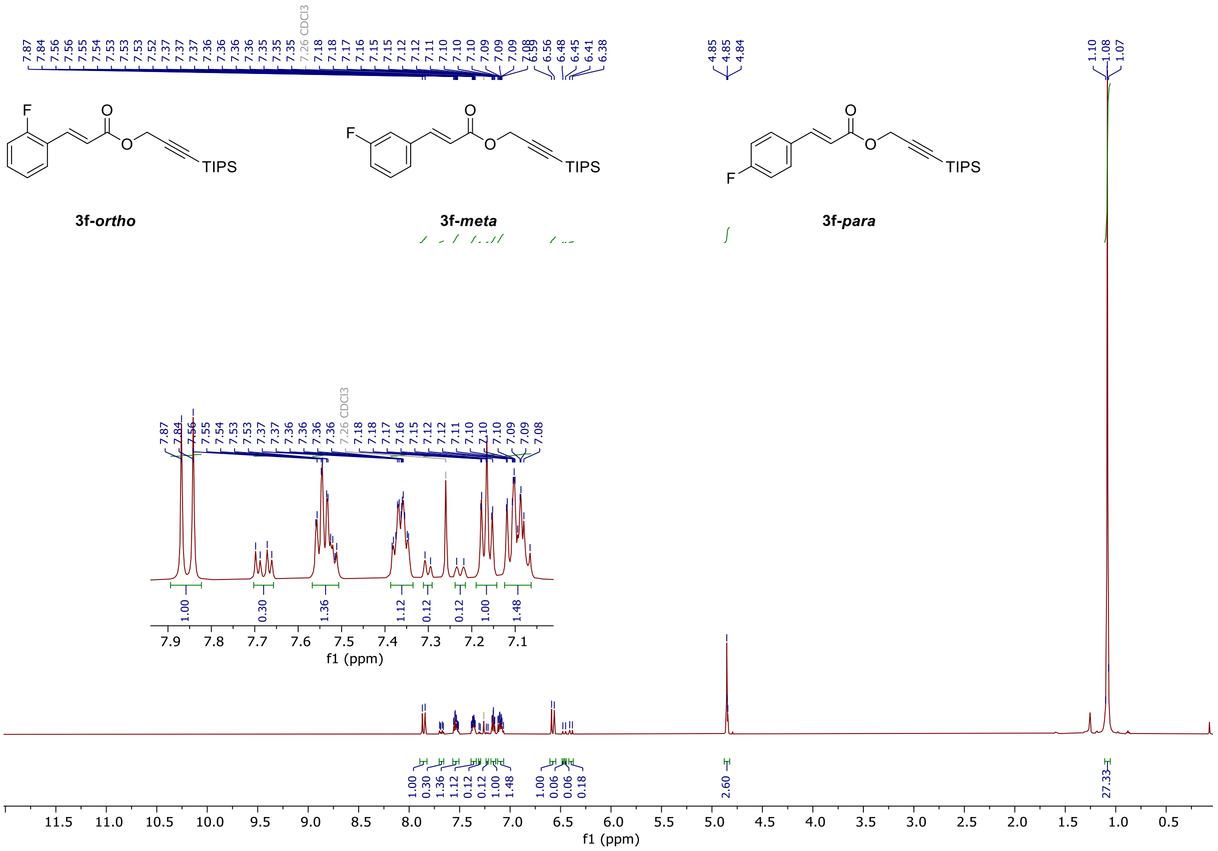
3f-ortho
3f-meta
3f-para
TIPS
f1 (ppm)

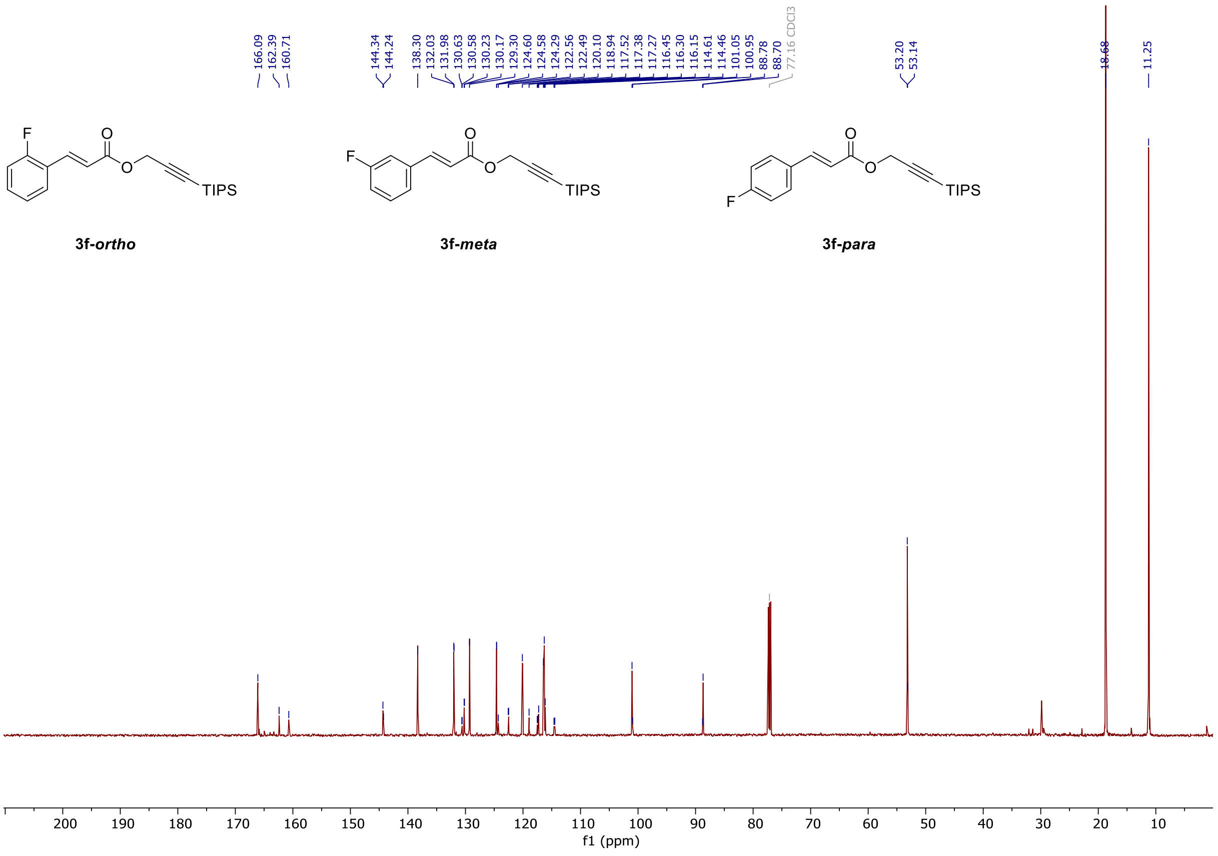
3f-ortho
3f-meta
3f-para
TIPS
f1 (ppm)

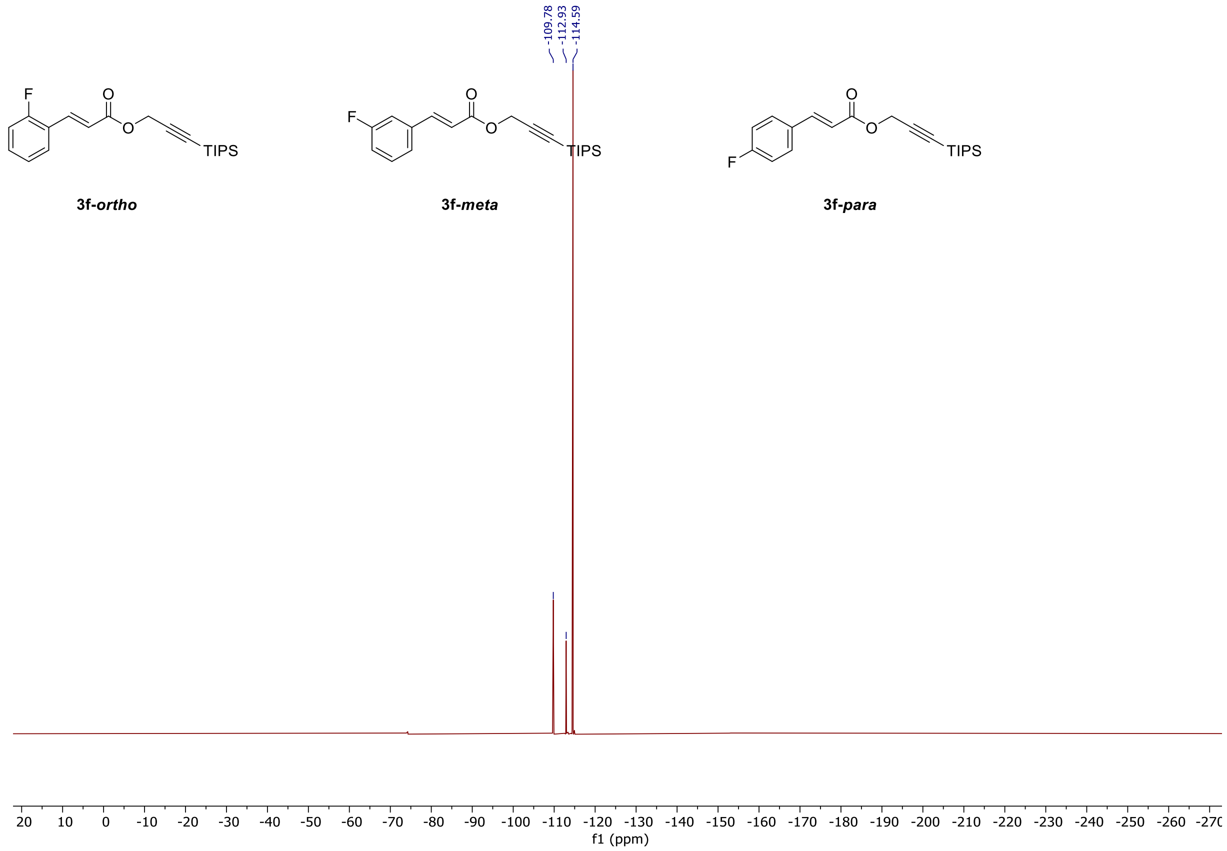
-109.78
-112.93
-114.59
F
O
O
TIPS
3f-*ortho*
3f-*meta*
3f-*para*
f1 (ppm)

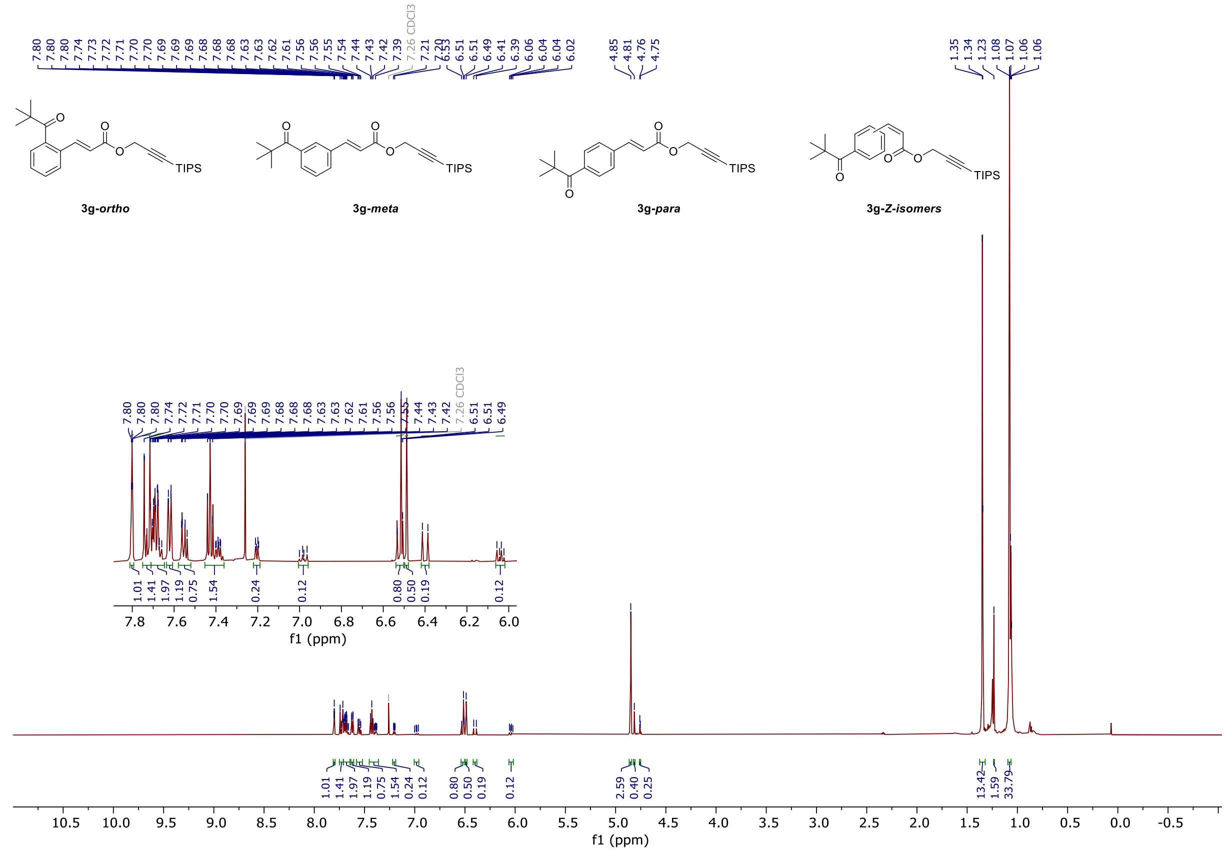
7.80 7.80 7.80 7.74 7.73 7.72 7.71 7.70 7.70 7.69 7.69 7.69 7.68 7.68 7.68 7.63 7.63 7.62 7.61 7.56 7.56 7.55 7.54 7.44 7.43 7.42 7.39 7.26 CDCl3 7.21 7.20 6.53 6.51 6.51 6.49 6.41 6.39 6.06 6.04 6.04 6.02
4.85 4.81 4.76 4.75
1.35 1.34 1.23 1.08 1.07 1.06 1.06
O
TIPS
3g-*ortho*
3g-*meta*
3g-*para*
3g-*Z-isomers*
f1 (ppm)

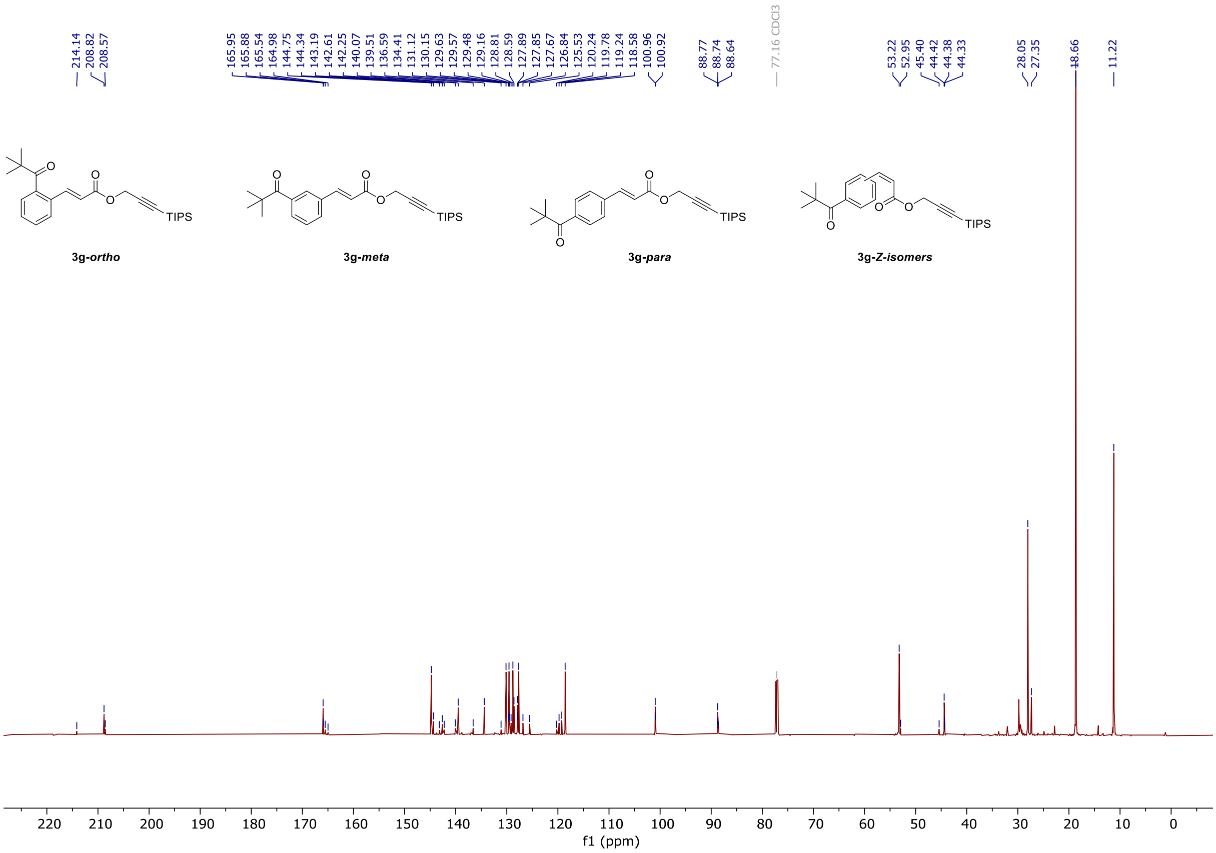
214.14
208.82
208.57
165.95
165.88
165.54
164.98
144.75
144.34
143.19
142.61
142.25
140.07
139.51
136.59
134.41
131.12
130.15
129.63
129.57
129.48
129.16
128.81
128.59
127.89
127.85
127.67
126.84
125.53
120.24
119.78
119.24
118.58
100.96
100.92
88.77
88.74
88.64
77.16 CDCl3
53.22
52.95
45.40
44.42
44.38
44.33
28.05
27.35
18.66
11.22
TIPS
3g-*ortho*
3g-*meta*
3g-*para*
3g-*Z-isomers*
220 210 200 190 180 170 160 150 140 130 120 110 100 90 80 70 60 50 40 30 20 10 0
f1 (ppm)

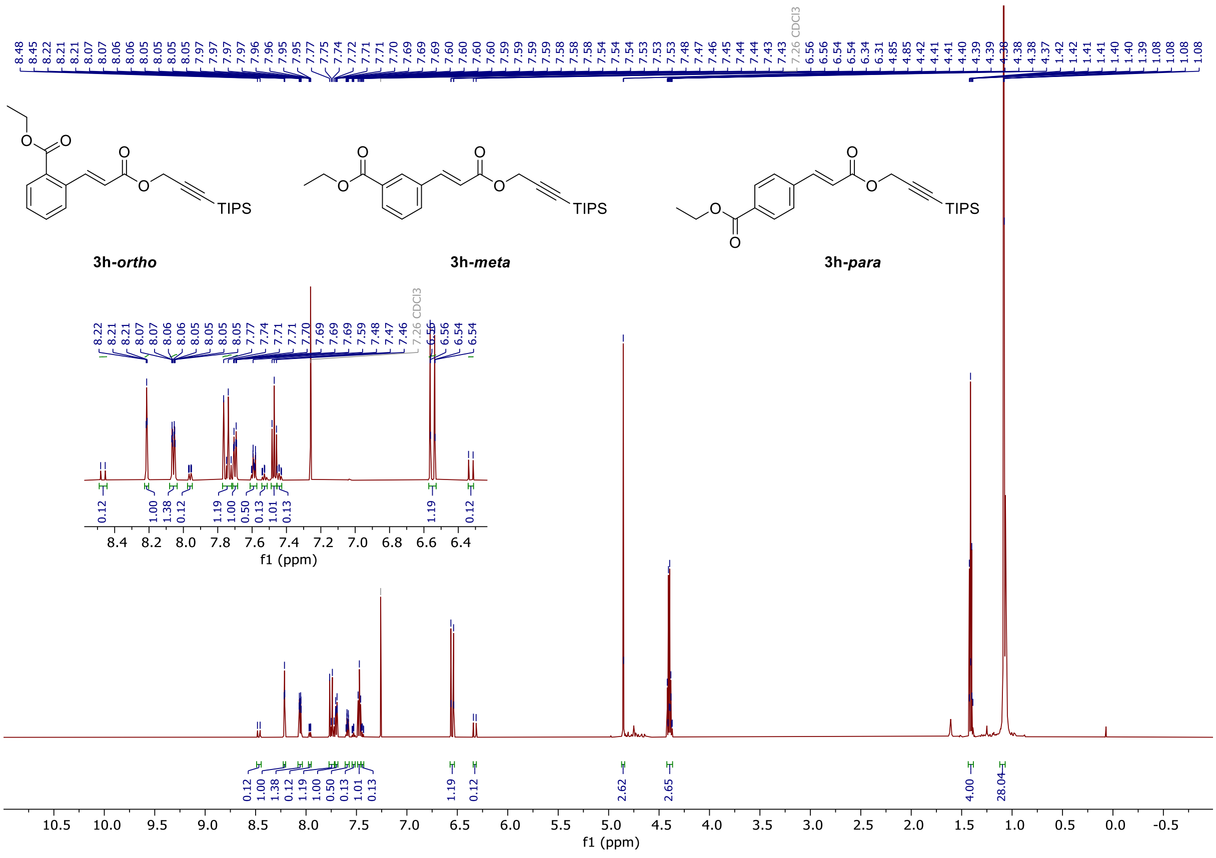
8.48
8.45
8.22
8.21
8.21
8.07
8.07
8.06
8.06
8.05
8.05
8.05
8.05
7.97
7.97
7.97
7.97
7.96
7.96
7.95
7.95
7.77
7.75
7.74
7.72
7.71
7.71
7.70
7.69
7.69
7.69
7.60
7.60
7.60
7.59
7.59
7.59
7.58
7.58
7.54
7.54
7.53
7.53
7.53
7.48
7.47
7.46
7.45
7.44
7.43
7.43
7.26 CDCl3
6.56
6.56
6.54
6.54
6.34
6.31
4.85
4.42
4.41
4.41
4.40
4.39
4.39
4.38
4.38
4.37
1.42
1.42
1.41
1.41
1.40
1.39
1.08
1.08
1.08
1.08
TIPS
3h-*ortho*
3h-*meta*
3h-*para*
8.22
8.21
8.21
8.07
8.07
8.06
8.06
8.05
8.05
8.05
8.05
7.77
7.74
7.71
7.71
7.70
7.69
7.69
7.59
7.48
7.47
7.46
7.26 CDCl3
6.56
6.56
6.54
6.54
0.12
1.00
1.38
0.12
1.19
1.00
0.50
0.13
1.01
0.13
1.19
0.12
8.4 8.2 8.0 7.8 7.6 7.4 7.2 7.0 6.8 6.6 6.4
f1 (ppm)
0.12
1.00
1.38
0.12
1.19
1.00
0.50
0.13
1.01
0.13
1.19
0.12
2.62
2.65
4.00
28.04
10.5 10.0 9.5 9.0 8.5 8.0 7.5 7.0 6.5 6.0 5.5 5.0 4.5 4.0 3.5 3.0 2.5 2.0 1.5 1.0 0.5 0.0 -0.5
f1 (ppm)

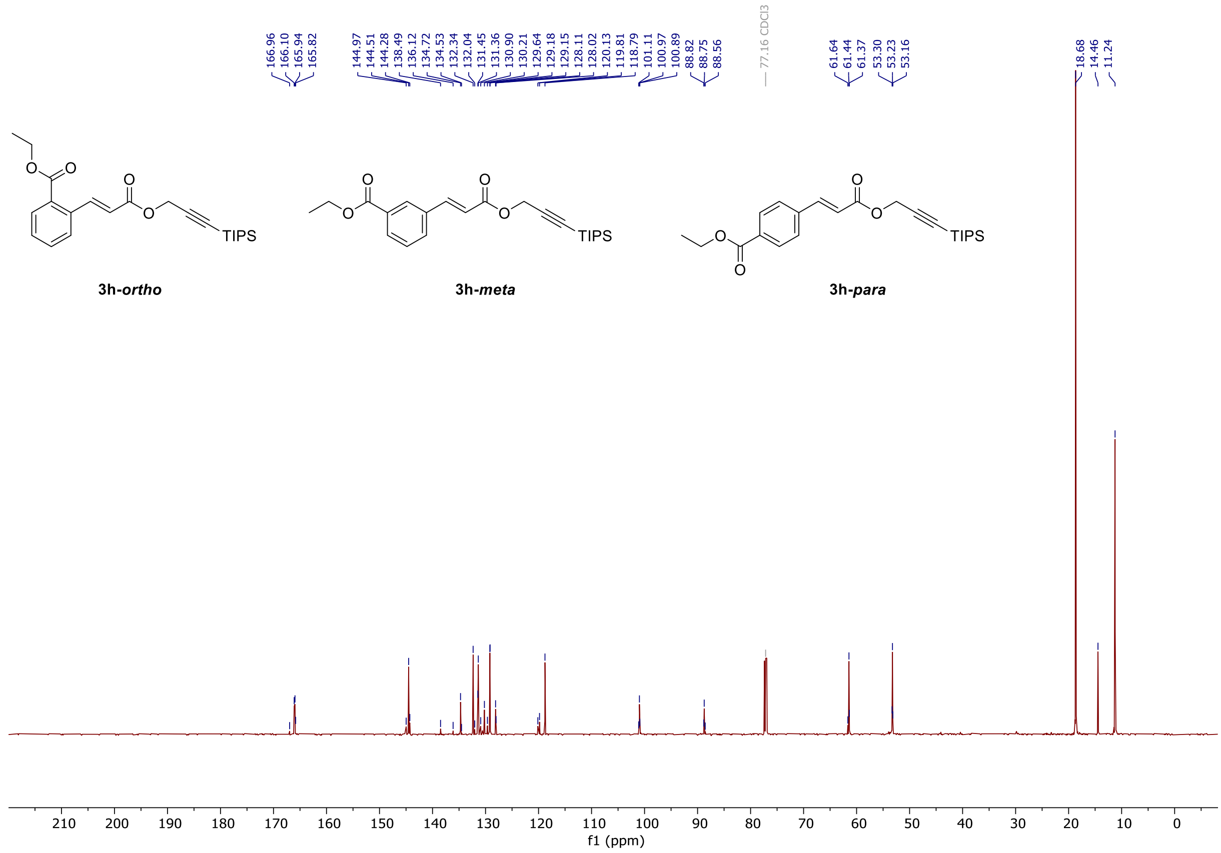
3h-*ortho*
3h-*meta*
3h-*para*
TIPS
f1 (ppm)

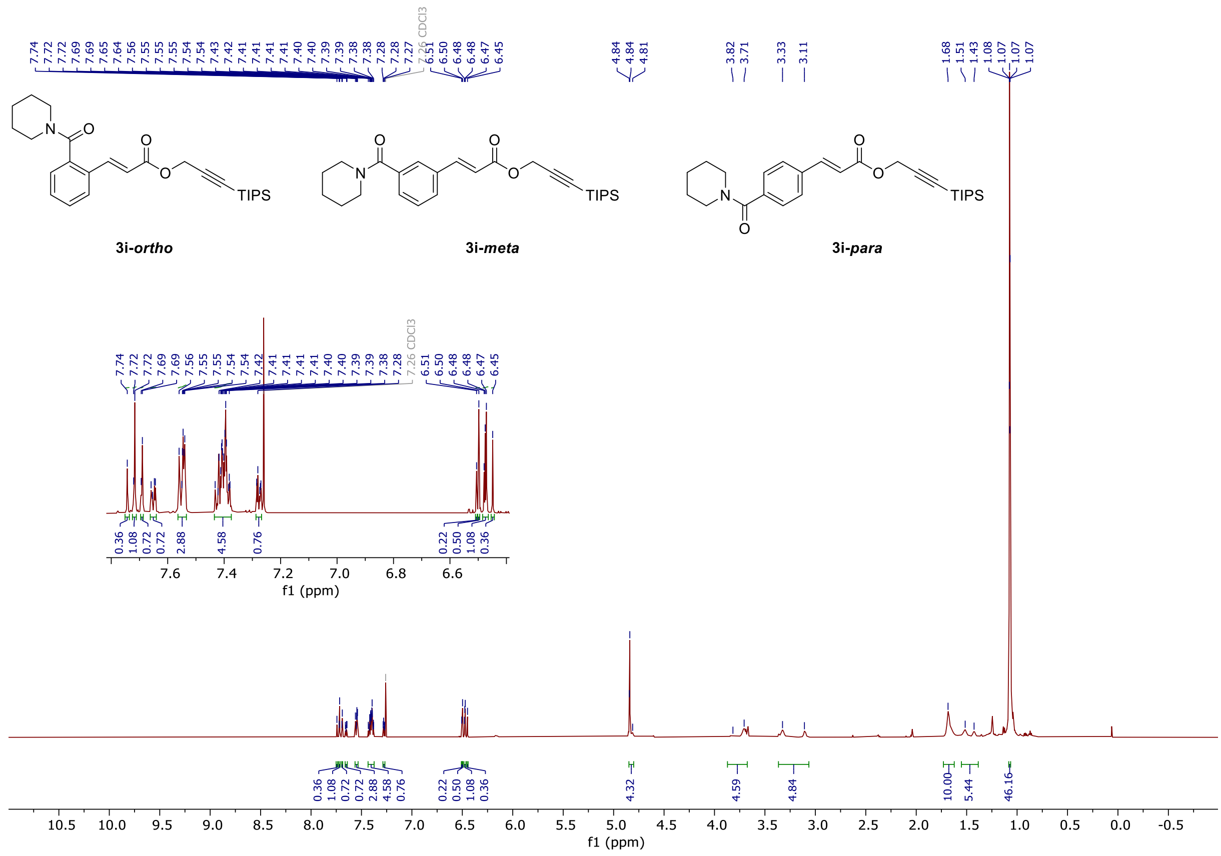
3i-*ortho*
3i-*meta*
3i-*para*
TIPS
f1 (ppm)

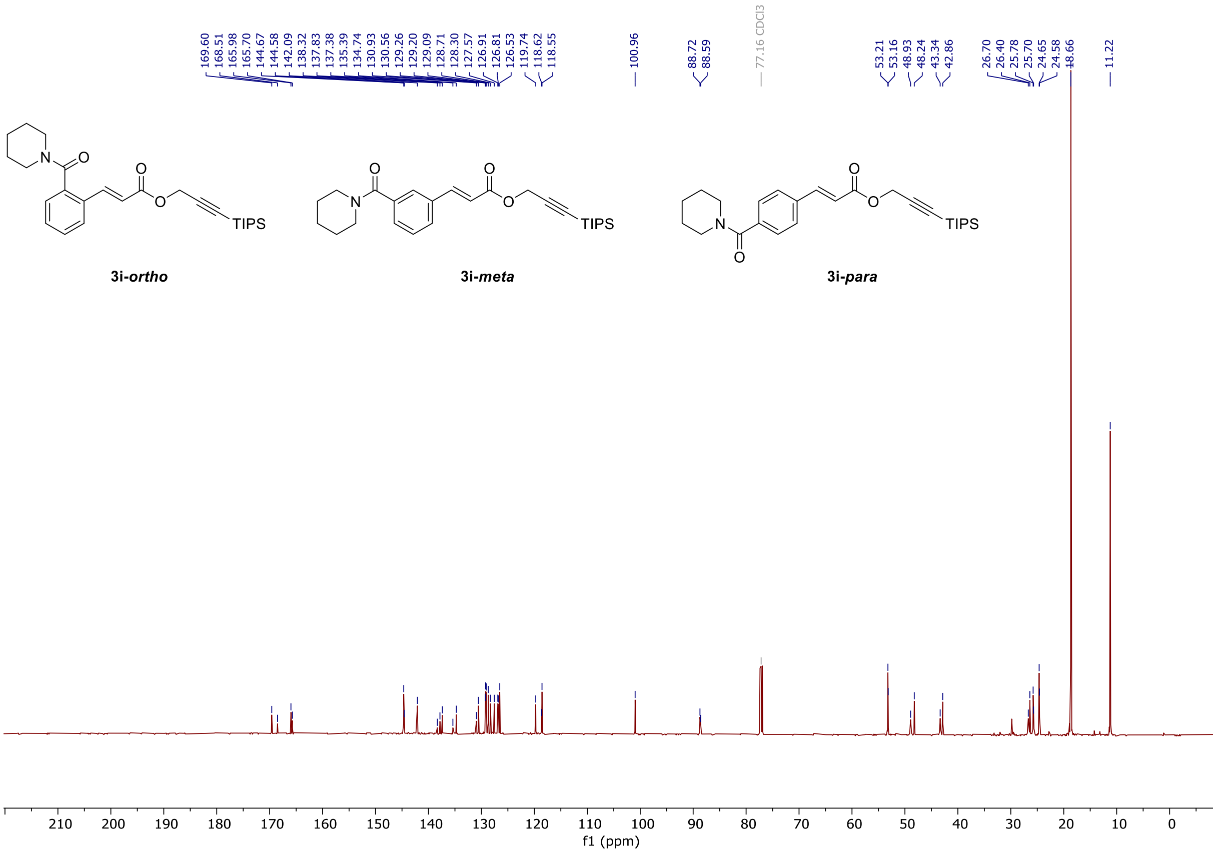
169.60
168.51
165.98
165.70
144.67
144.58
142.09
138.32
137.83
137.38
135.39
134.74
130.93
130.56
129.26
129.20
129.09
128.71
128.30
127.57
126.91
126.81
126.53
119.74
118.62
118.55
100.96
88.72
88.59
77.16 CDCl3
53.21
53.16
48.93
48.24
43.34
42.86
26.70
26.40
25.78
25.70
24.65
24.58
18.66
11.22
N
O
O
O
TIPS
3i-ortho
3i-meta
3i-para
210 200 190 180 170 160 150 140 130 120 110 100 90 80 70 60 50 40 30 20 10 0
f1 (ppm)

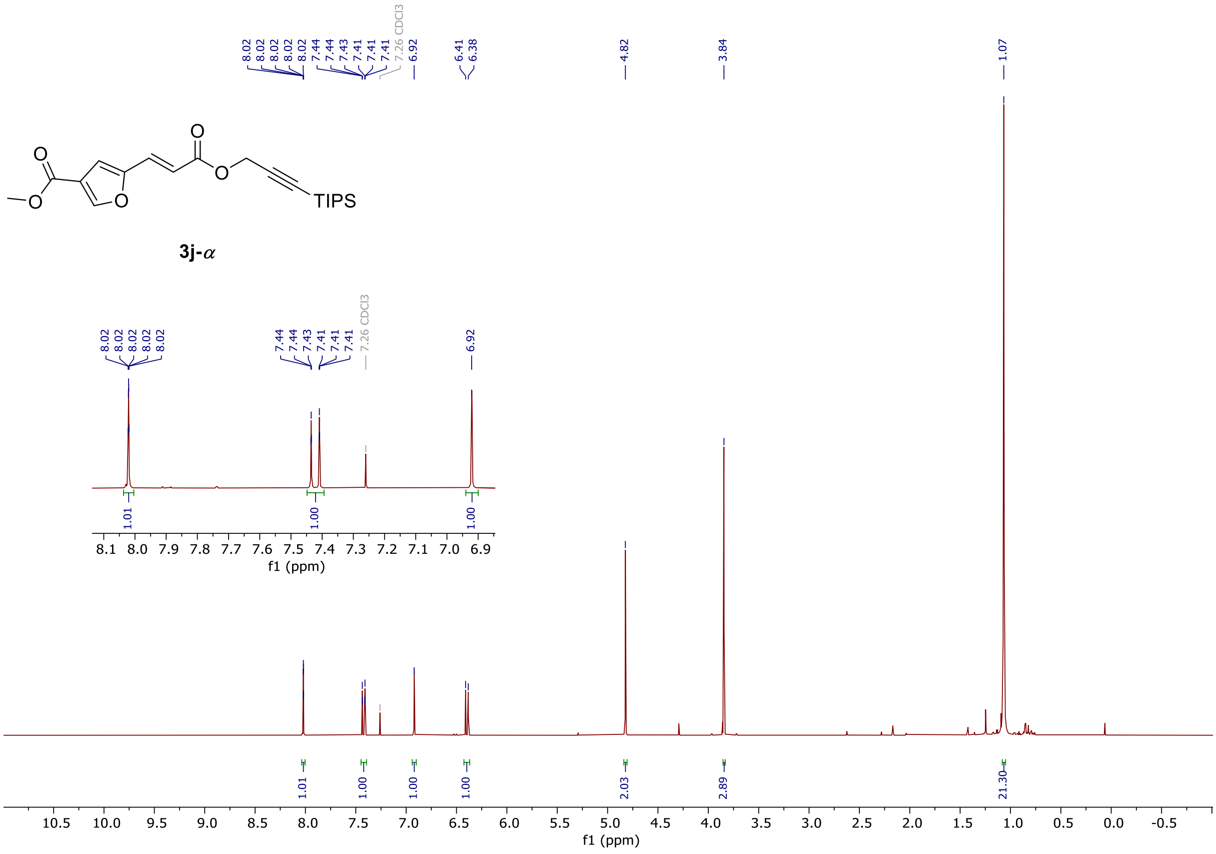
8.02
8.02
8.02
8.02
8.02
7.44
7.44
7.43
7.41
7.41
7.41
7.26 CDCl3
6.92
6.41
6.38
4.82
3.84
1.07
O
TIPS
3j-α
1.01
1.00
1.00
8.1 8.0 7.9 7.8 7.7 7.6 7.5 7.4 7.3 7.2 7.1 7.0 6.9
f1 (ppm)
1.01
1.00
1.00
1.00
2.03
2.89
21.30
10.5 10.0 9.5 9.0 8.5 8.0 7.5 7.0 6.5 6.0 5.5 5.0 4.5 4.0 3.5 3.0 2.5 2.0 1.5 1.0 0.5 0.0 -0.5
f1 (ppm)

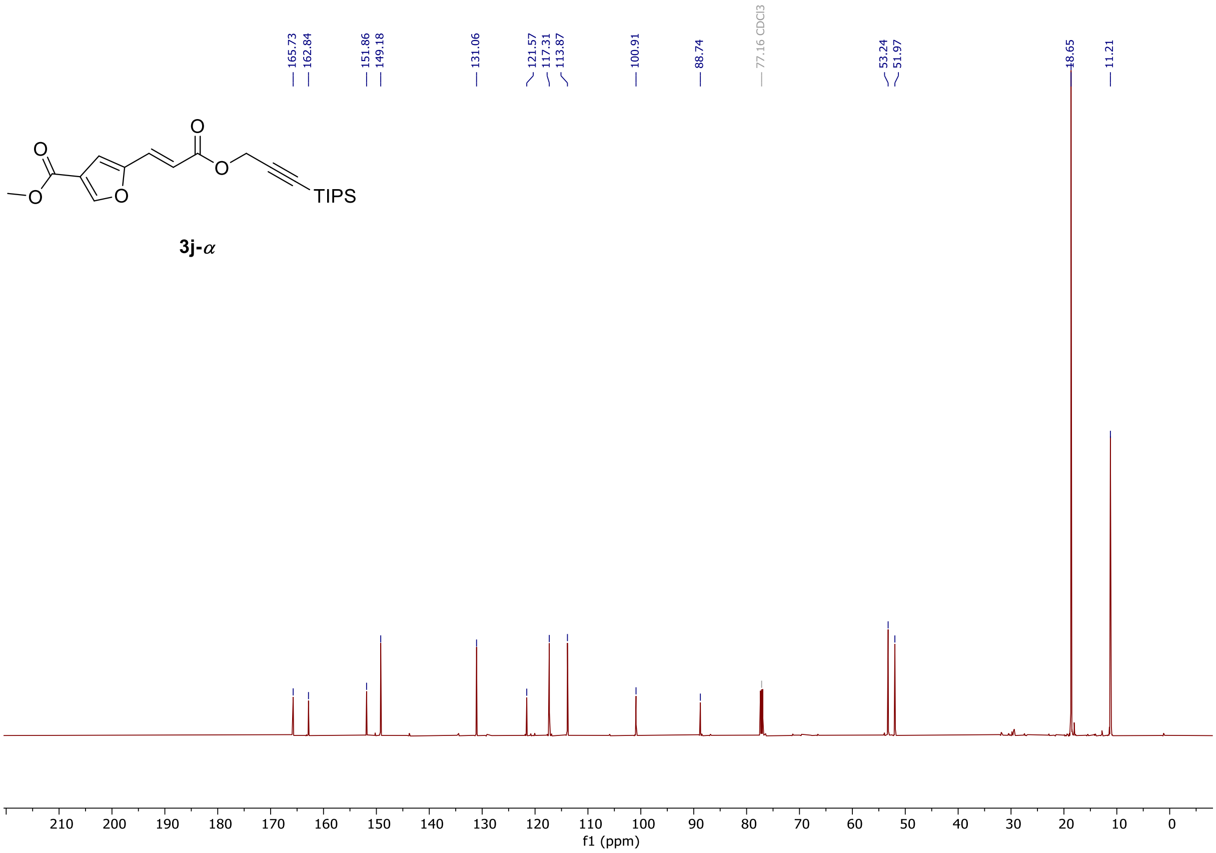
TIPS
3j-α
77.16 CDCl3
f1 (ppm)

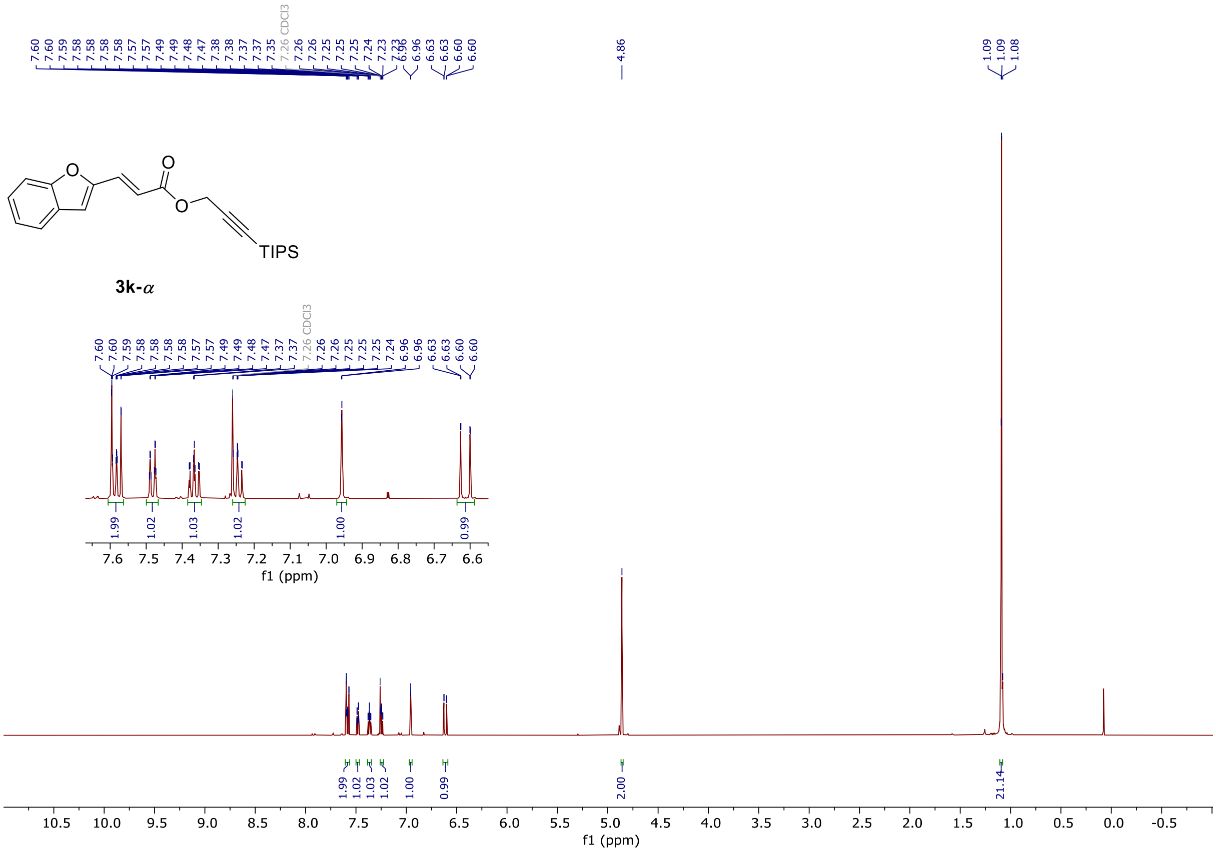
TIPS
3k-α
7.26 CDCl3
f1 (ppm)

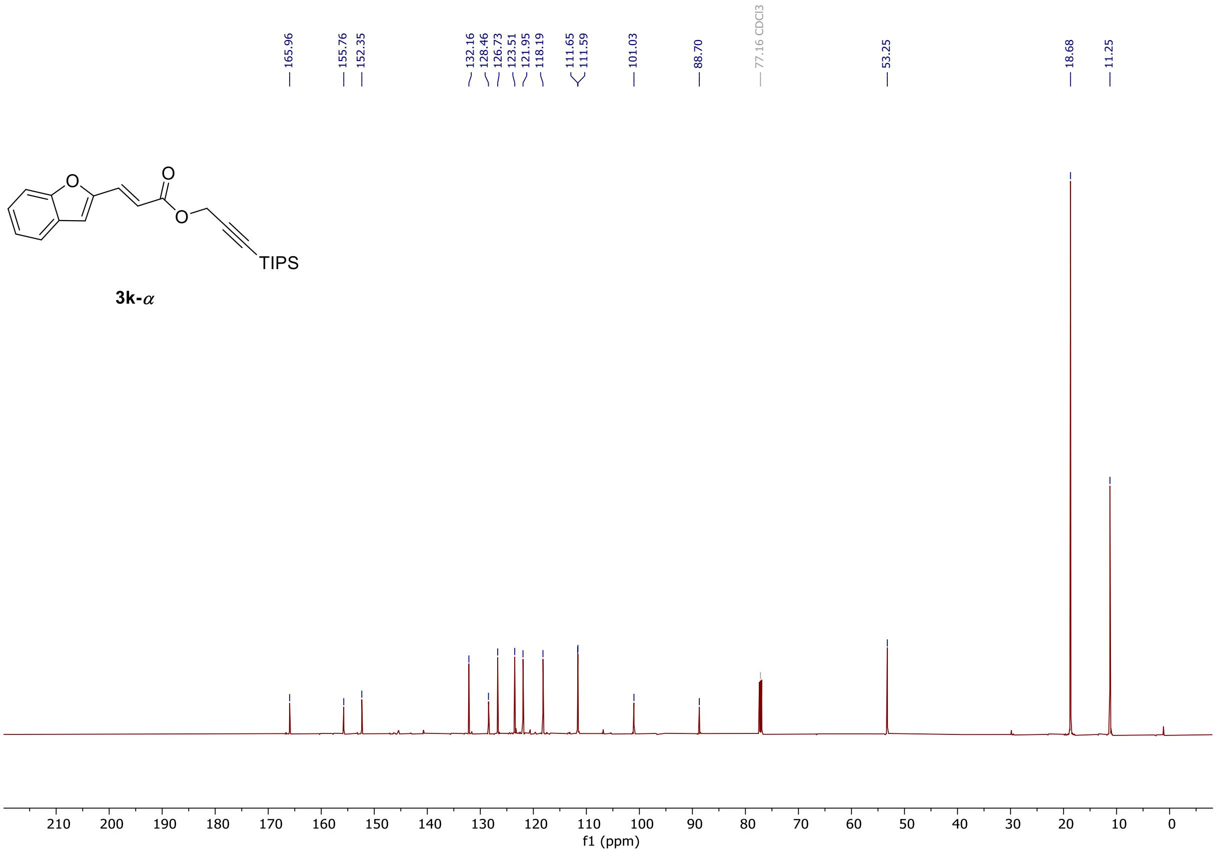
165.96
155.76
152.35
132.16
128.46
126.73
123.51
121.95
118.19
111.65
111.59
101.03
88.70
77.16 CDCl3
53.25
18.68
11.25
O
O
O
TIPS
3k-α
210 200 190 180 170 160 150 140 130 120 110 100 90 80 70 60 50 40 30 20 10 0
f1 (ppm)

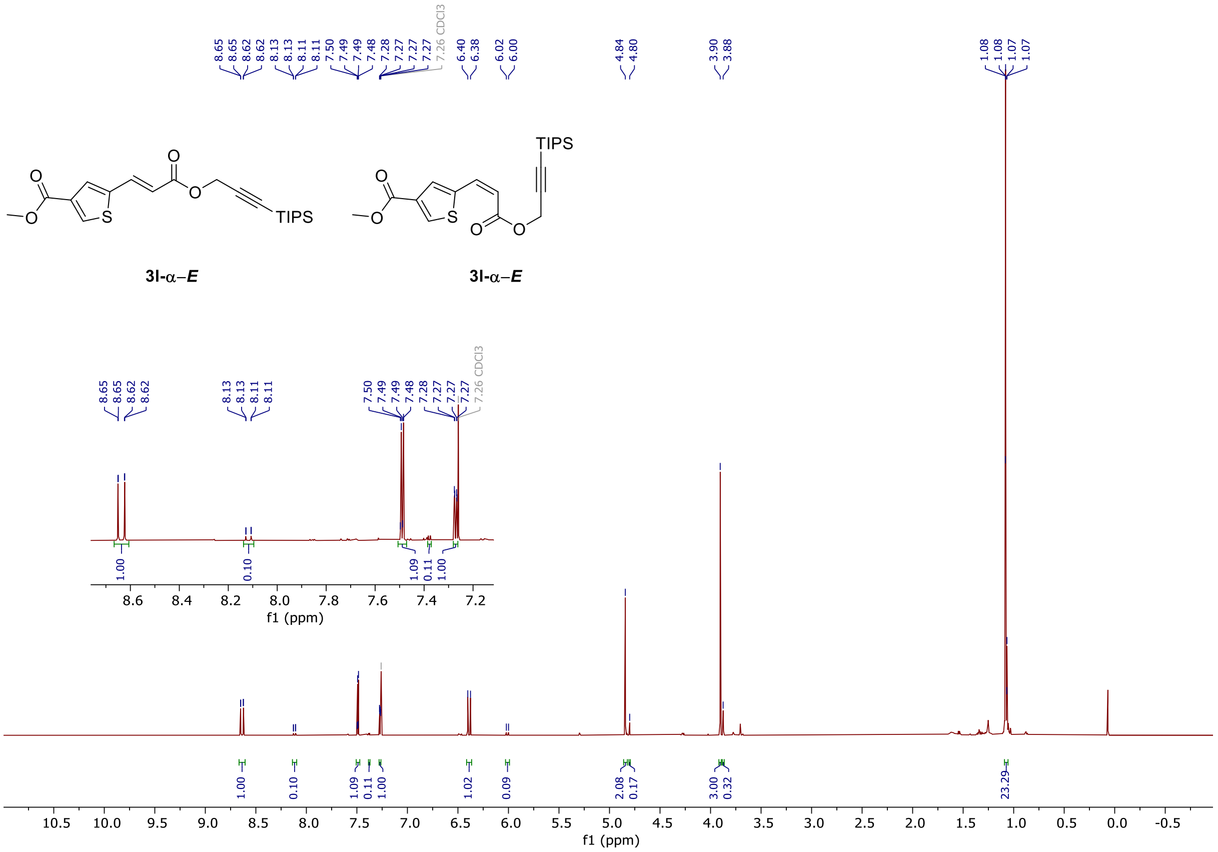
8.65
8.65
8.62
8.62
8.13
8.13
8.11
8.11
7.50
7.49
7.49
7.48
7.28
7.27
7.27
7.27
7.26 CDCl3
6.40
6.38
6.02
6.00
4.84
4.80
3.90
3.88
1.08
1.08
1.07
1.07
TIPS
S
3l-α–E
3l-α–E
1.00
0.10
1.09
0.11
1.00
8.6 8.4 8.2 8.0 7.8 7.6 7.4 7.2
f1 (ppm)
1.00
0.10
1.09
0.11
1.00
1.02
0.09
2.08
0.17
3.00
0.32
23.29
10.5 10.0 9.5 9.0 8.5 8.0 7.5 7.0 6.5 6.0 5.5 5.0 4.5 4.0 3.5 3.0 2.5 2.0 1.5 1.0 0.5 0.0 -0.5
f1 (ppm)

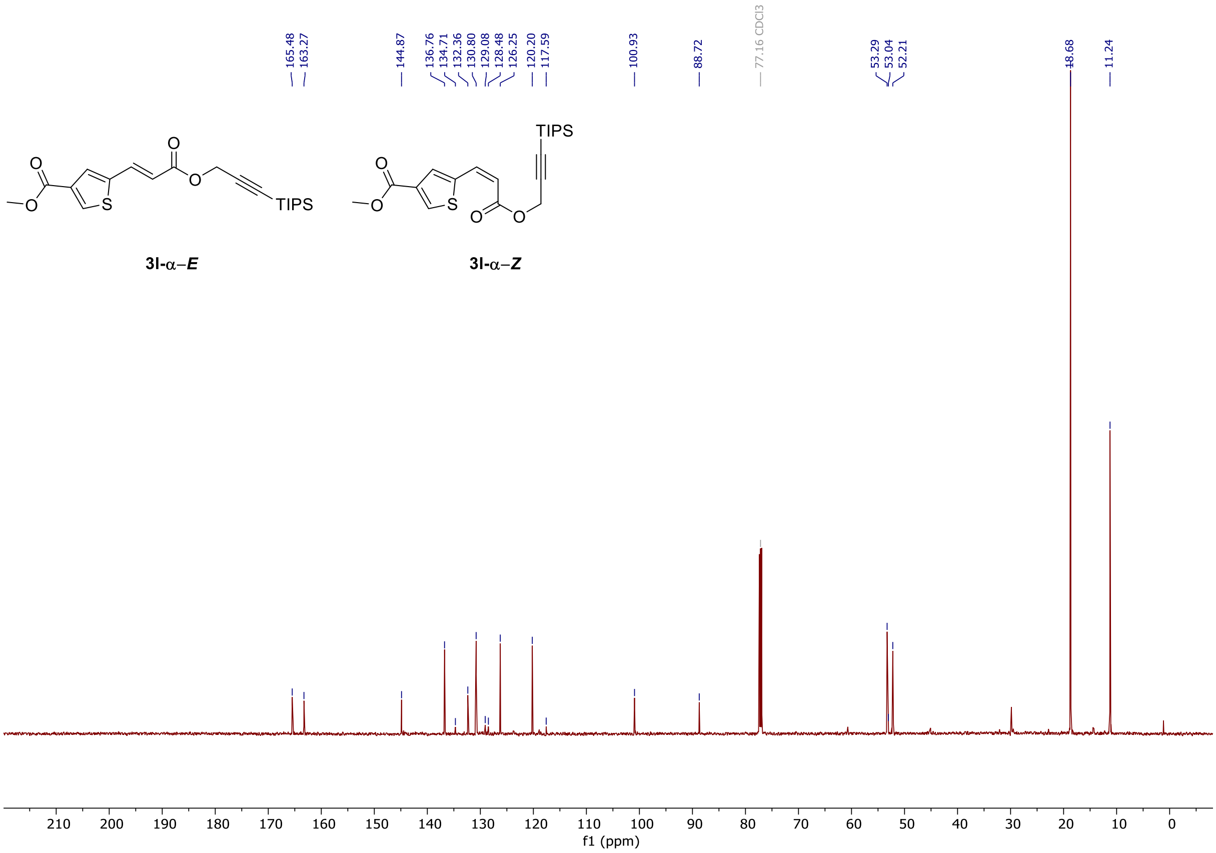
165.48
163.27
144.87
136.76
134.71
132.36
130.80
129.08
128.48
126.25
120.20
117.59
100.93
88.72
77.16 CDCl3
53.29
53.04
52.21
18.68
11.24
TIPS
3l-α–E
3l-α–Z
f1 (ppm)

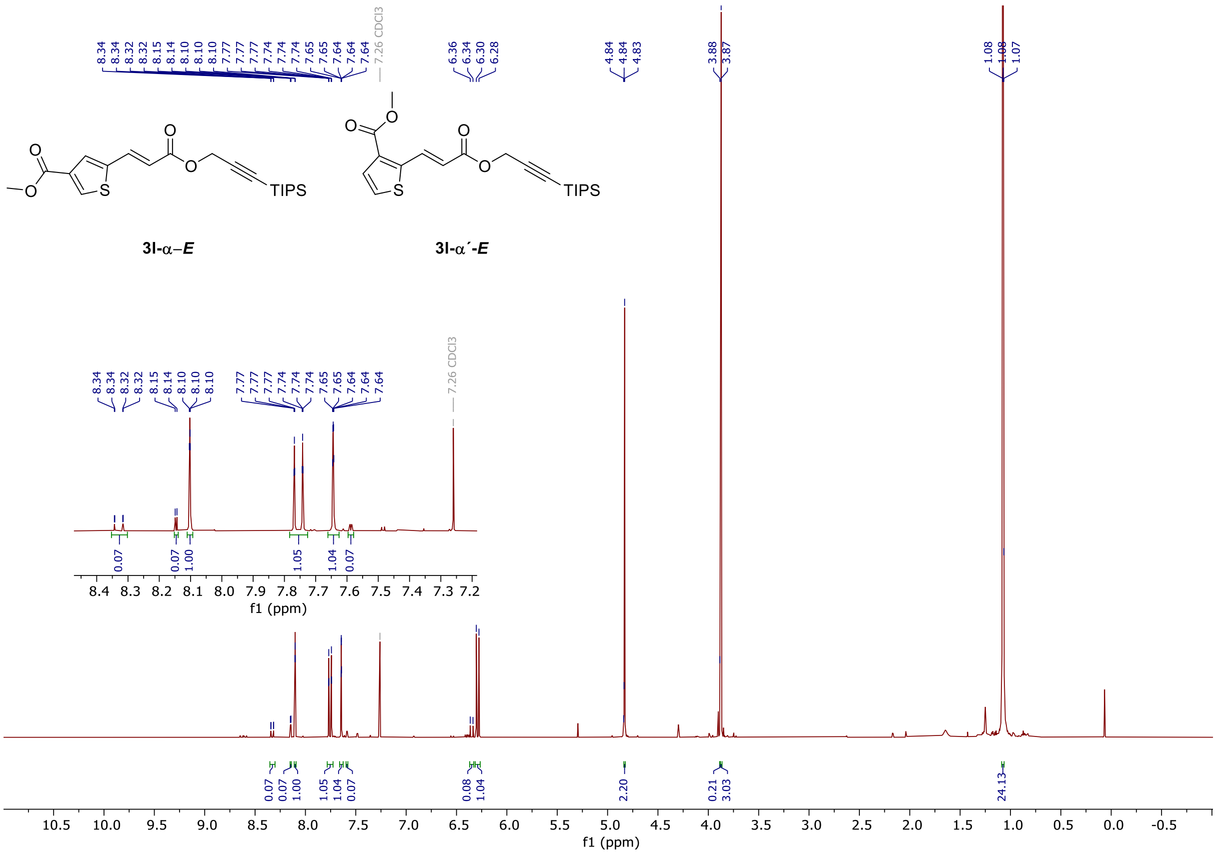
8.34
8.34
8.32
8.32
8.15
8.14
8.10
8.10
8.10
8.10
7.77
7.77
7.77
7.74
7.74
7.74
7.65
7.64
7.64
7.64
7.26 CDCl3
6.36
6.34
6.30
6.28
4.84
4.84
4.83
3.88
3.87
1.08
1.08
1.07
TIPS
3l-α–E
3l-α´-E
0.07
0.07
1.00
1.05
1.04
0.07
0.08
1.04
2.20
0.21
3.03
24.13
f1 (ppm)

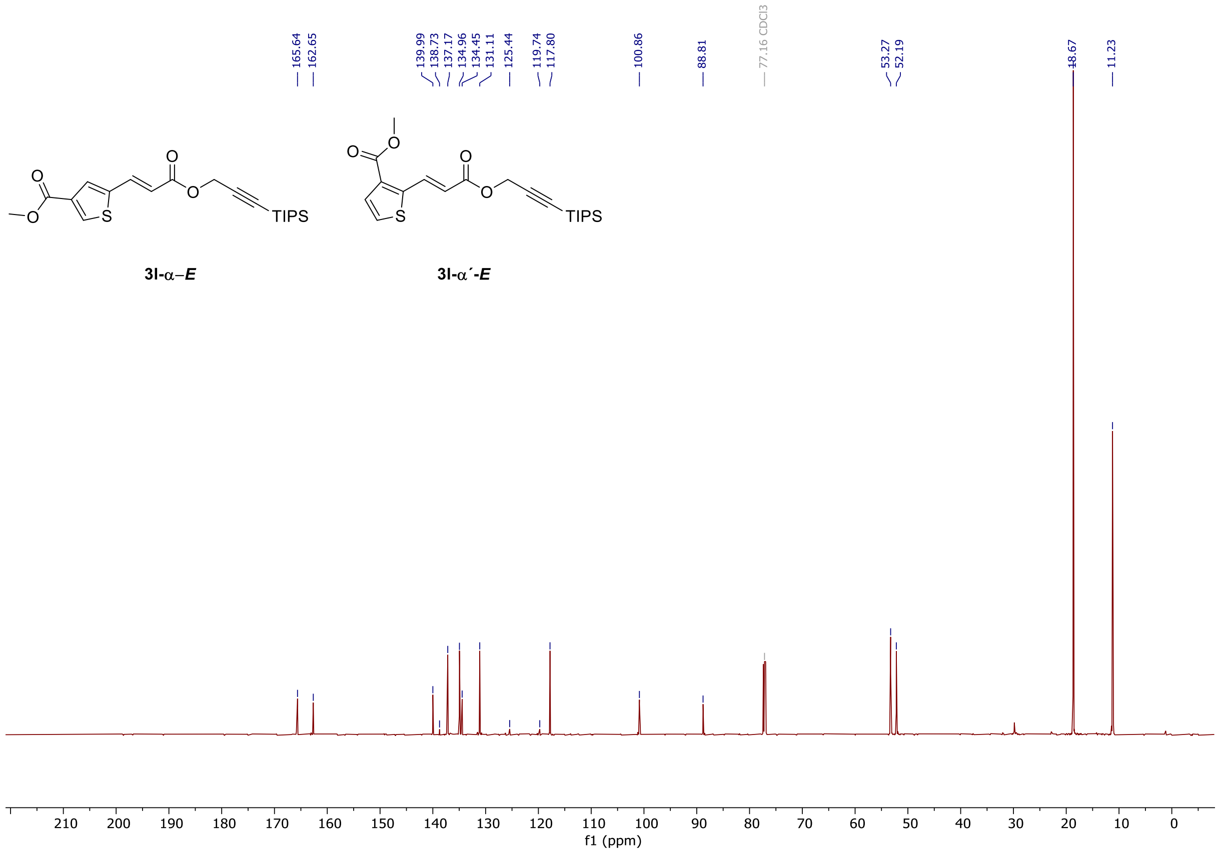
165.64
162.65
139.99
138.73
137.17
134.96
134.45
131.11
125.44
119.74
117.80
100.86
88.81
77.16 CDCl3
53.27
52.19
18.67
11.23
TIPS
TIPS
3l-α–E
3l-α´-E
f1 (ppm)

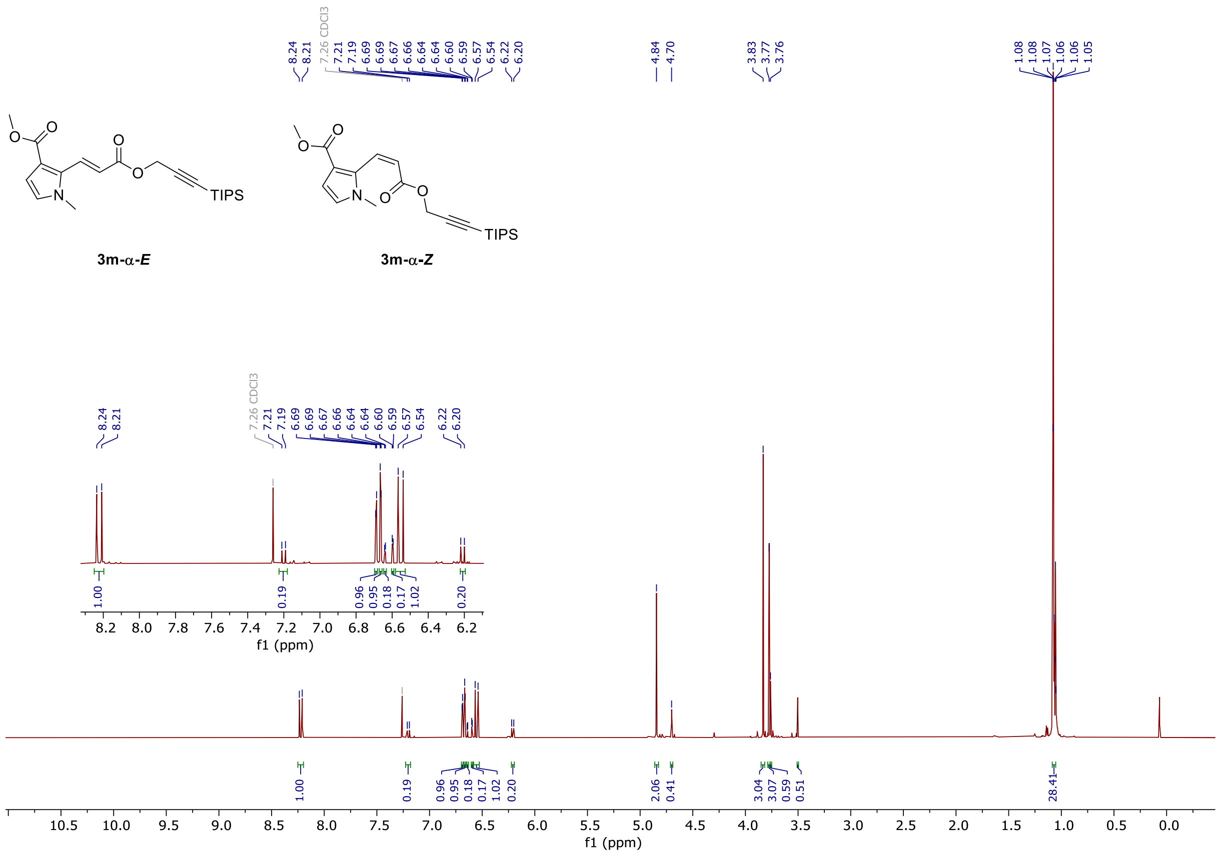
8.24
8.21
7.26 CDCl3
7.21
7.19
6.69
6.69
6.67
6.66
6.64
6.64
6.60
6.59
6.57
6.54
6.22
6.20
4.84
4.70
3.83
3.77
3.76
1.08
1.08
1.07
1.06
1.06
1.05
TIPS
TIPS
3m-α-E
3m-α-Z
1.00
0.19
0.96
0.95
0.18
0.17
1.02
0.20
2.06
0.41
3.04
3.07
0.59
0.51
28.41
f1 (ppm)

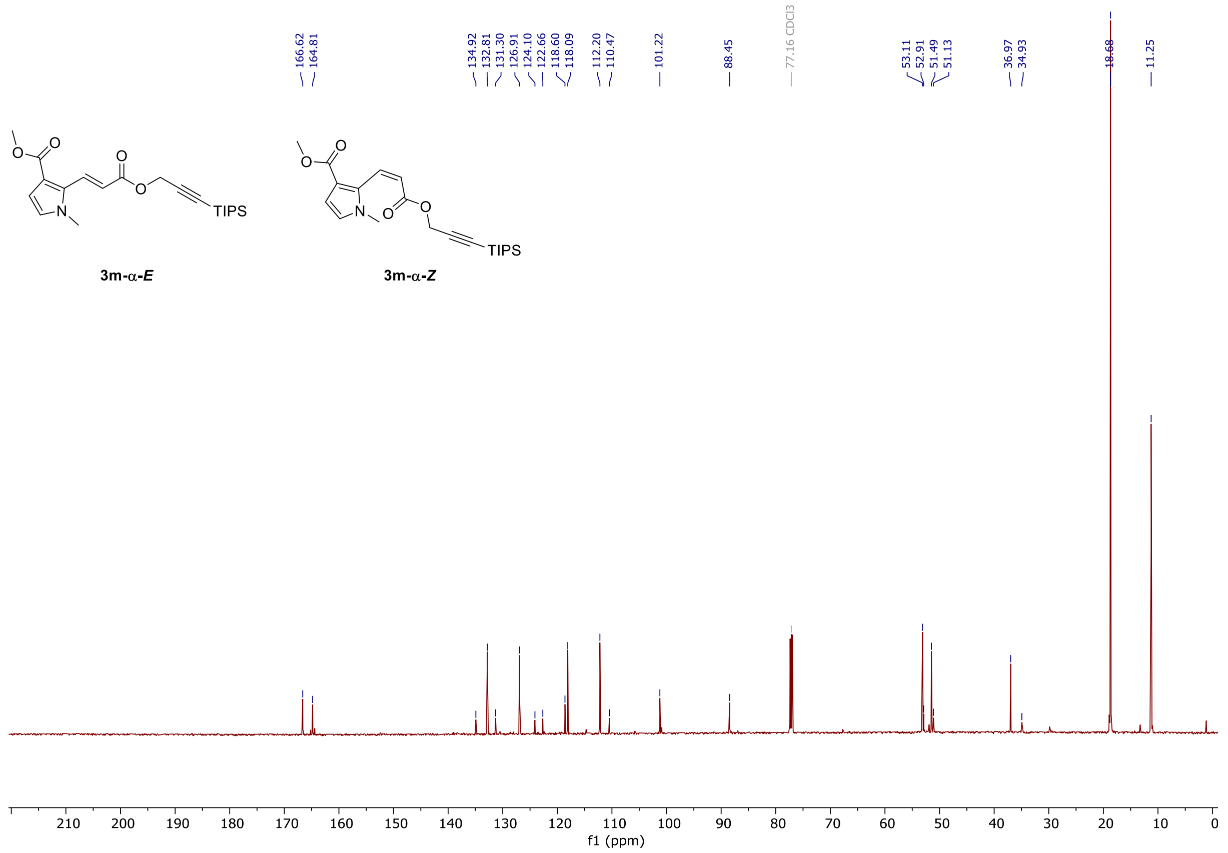

166.62
164.81
134.92
132.81
131.30
126.91
124.10
122.66
118.60
118.09
112.20
110.47
101.22
88.45
77.16 CDCl3
53.11
52.91
51.49
51.13
36.97
34.93
18.68
11.25
TIPS
3m-α-E
3m-α-Z
f1 (ppm)

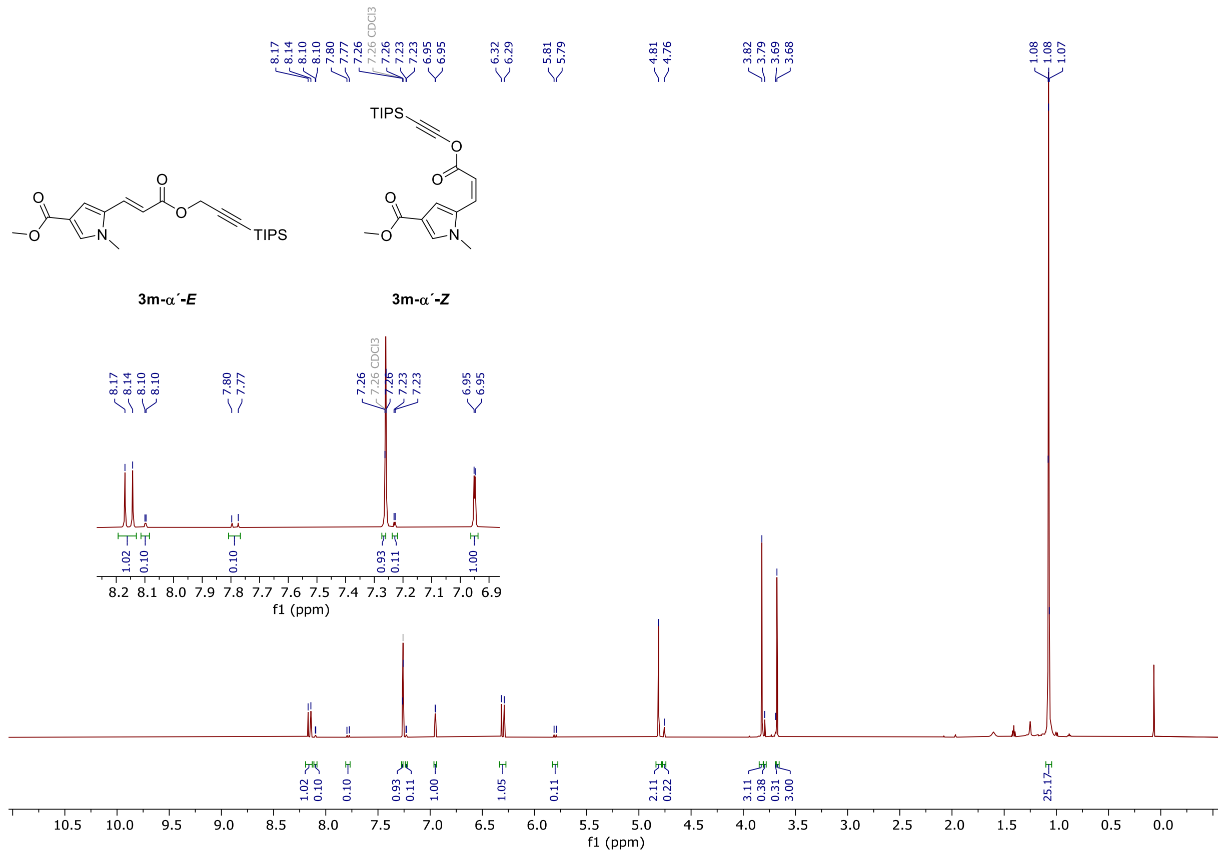

8.17
8.14
8.10
8.10
7.80
7.77
7.26
7.26 CDCl3
7.26
7.23
7.23
6.95
6.95
6.32
6.29
5.81
5.79
4.81
4.76
3.82
3.79
3.69
3.68
1.08
1.08
1.07
TIPS
3m-α´-E
3m-α´-Z
1.02
0.10
0.93
0.11
1.00
1.05
0.11
2.11
0.22
3.11
0.38
0.31
3.00
25.17
f1 (ppm)

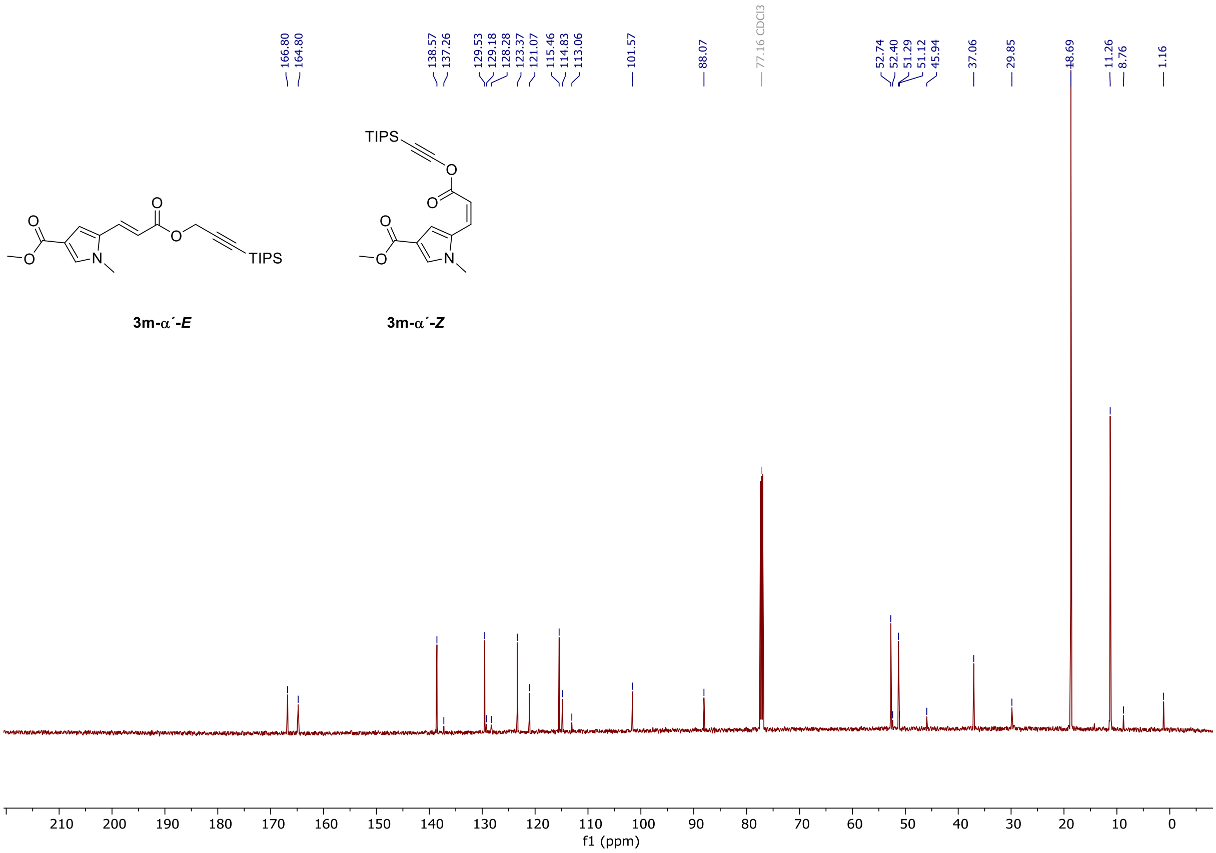
166.80
164.80
138.57
137.26
129.53
129.18
128.28
123.37
121.07
115.46
114.83
113.06
101.57
88.07
77.16 CDCl3
52.74
52.40
51.29
51.12
45.94
37.06
29.85
18.69
11.26
8.76
1.16
TIPS
TIPS
3m-α´-E
3m-α´-Z
f1 (ppm)

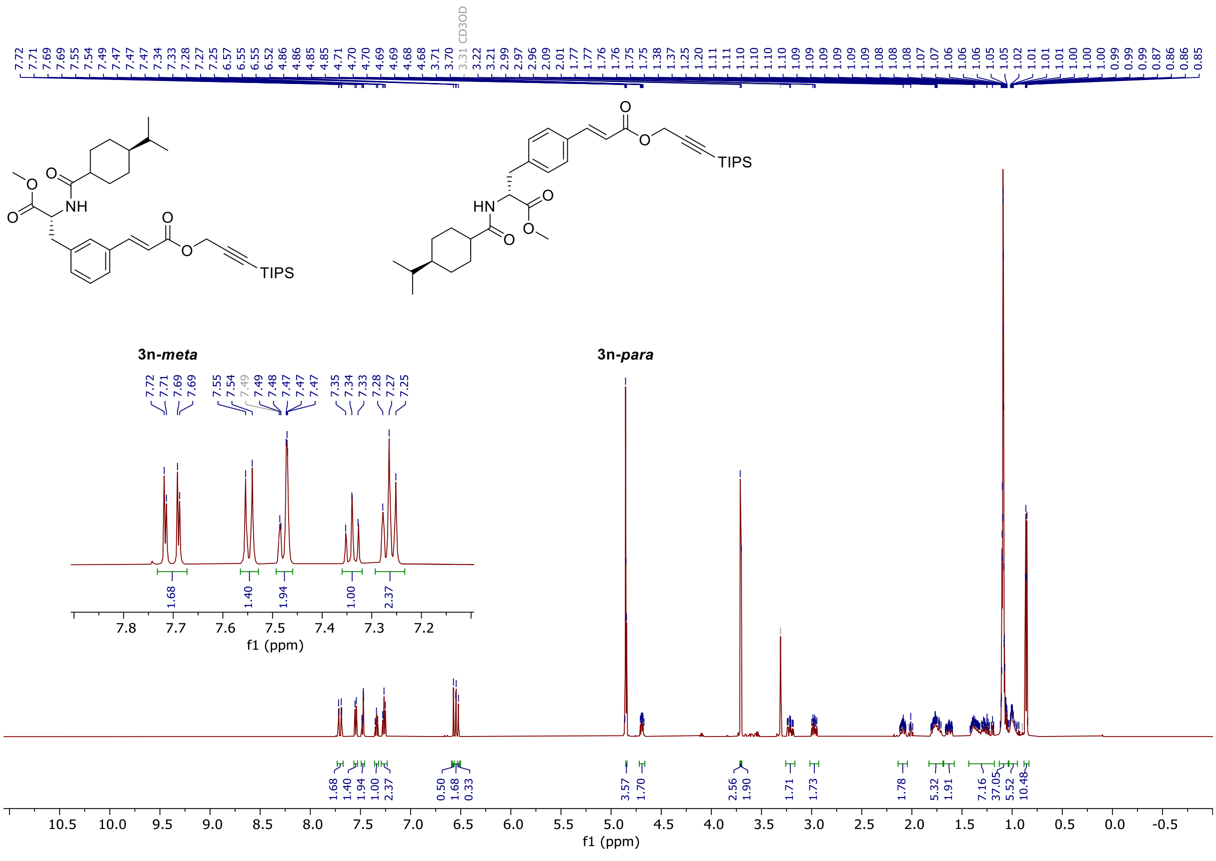
3.31 CD3OD
TIPS
NH
HN
TIPS
3n-meta
3n-para
f1 (ppm)
f1 (ppm)

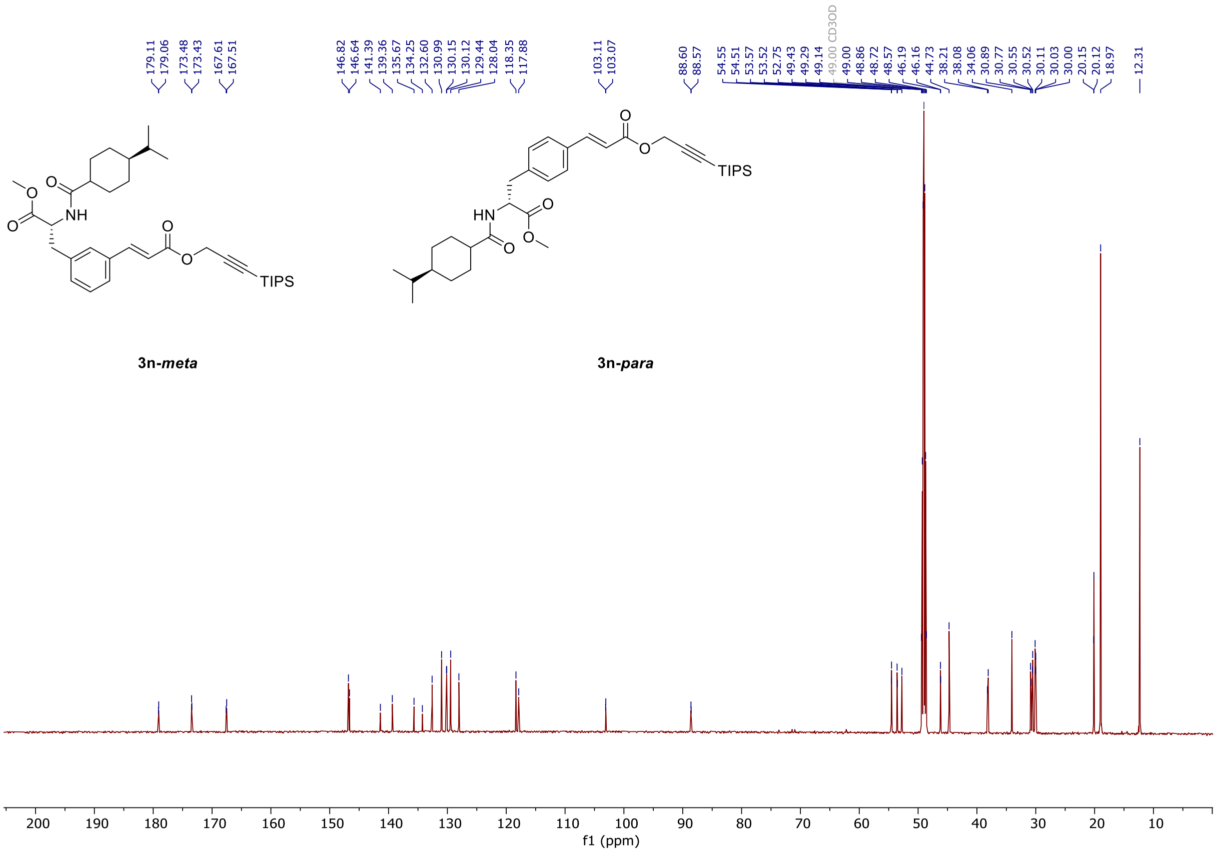
179.11
179.06
173.48
173.43
167.61
167.51
146.82
146.64
141.39
139.36
135.67
134.25
132.60
130.99
130.15
130.12
129.44
128.04
118.35
117.88
103.11
103.07
88.60
88.57
54.55
54.51
53.57
53.52
52.75
49.43
49.29
49.14
49.00 CD3OD
49.00
48.86
48.72
48.57
46.19
46.16
44.73
38.21
38.08
34.06
30.89
30.77
30.55
30.52
30.11
30.03
30.00
20.15
20.12
18.97
12.31
O
NH
O
O
O
O
TIPS
HN
O
O
O
O
O
TIPS
3n-*meta*
3n-*para*
f1 (ppm)

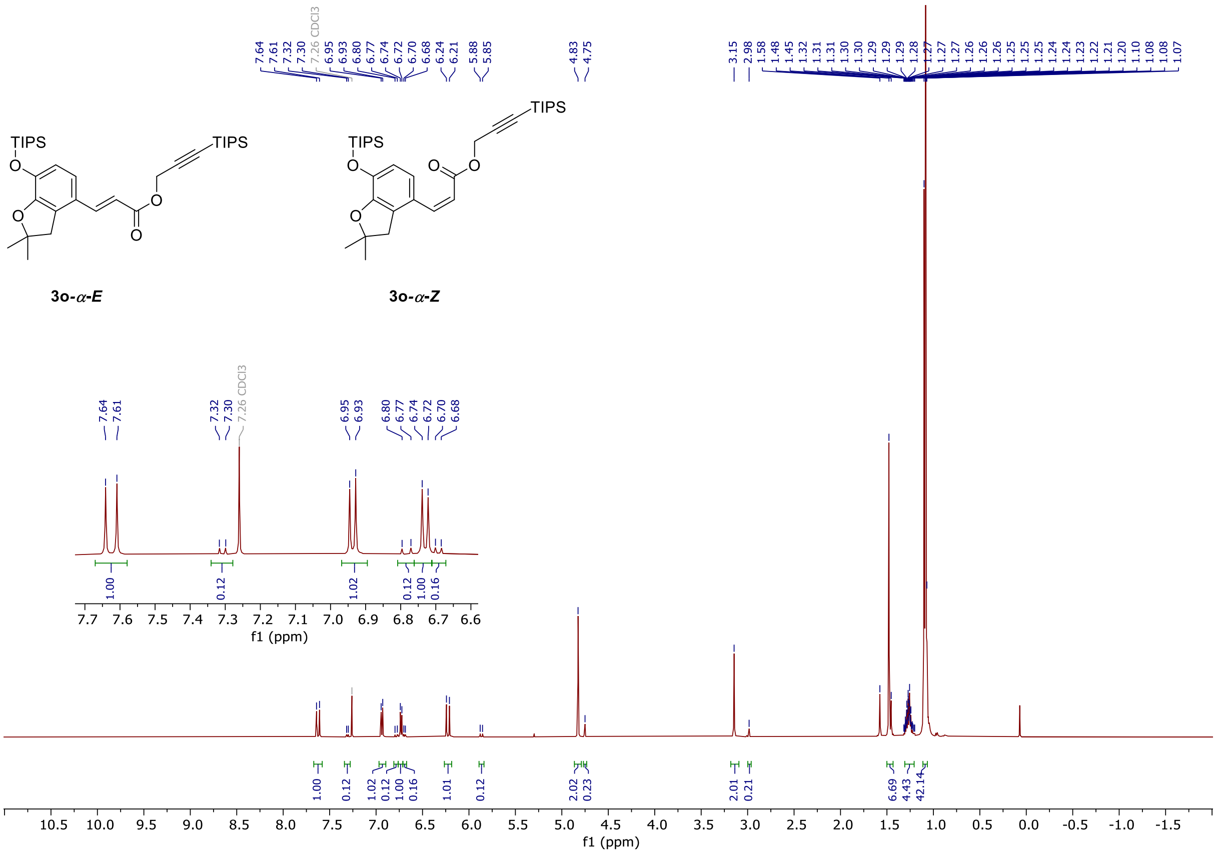
7.64
7.61
7.32
7.30
7.26 CDCl3
6.95
6.93
6.80
6.77
6.74
6.72
6.70
6.68
6.24
6.21
5.88
5.85
4.83
4.75
3.15
2.98
1.58
1.48
1.45
1.32
1.31
1.30
1.30
1.29
1.29
1.28
1.27
1.27
1.26
1.26
1.25
1.25
1.24
1.24
1.23
1.22
1.21
1.20
1.10
1.08
1.08
1.07
TIPS
O
O
O
O
TIPS
TIPS
O
O
O
O
O
TIPS
3o-α-*E*
3o-α-*Z*
1.00
0.12
1.02
0.12
1.00
0.16
1.01
0.12
2.02
0.23
2.01
0.21
6.69
4.43
42.14
f1 (ppm)

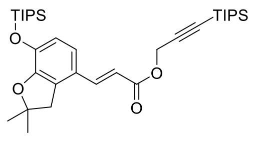

3o-*α*-*E*

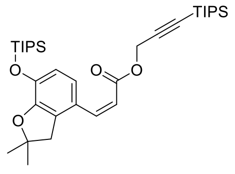

3o-*α*-*Z*

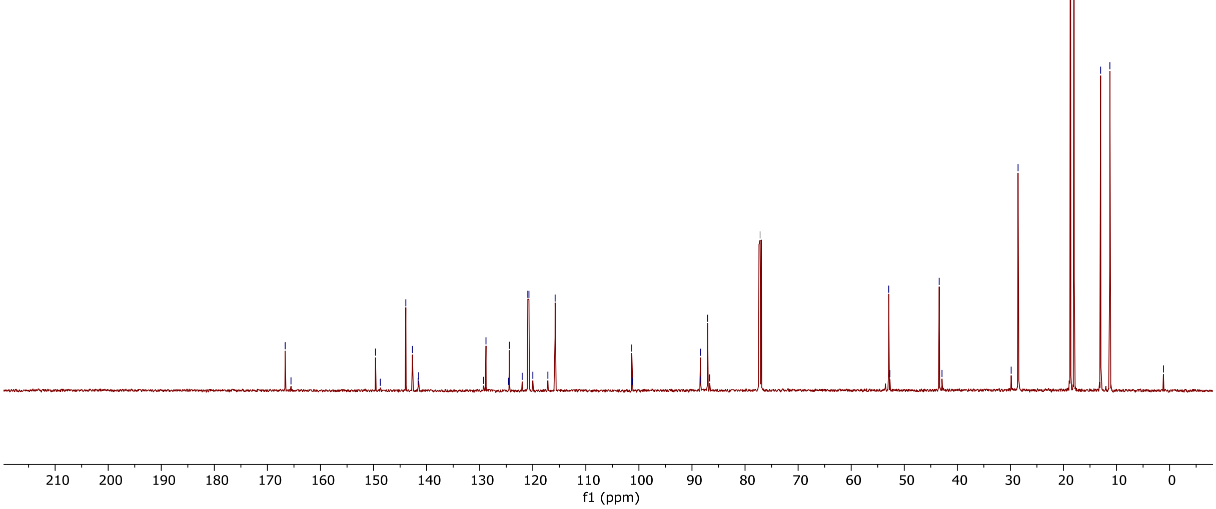

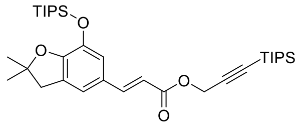

3o-*β*-*E*

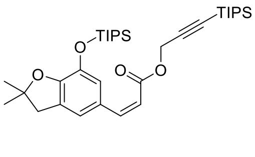

3o-*β*-*Z*

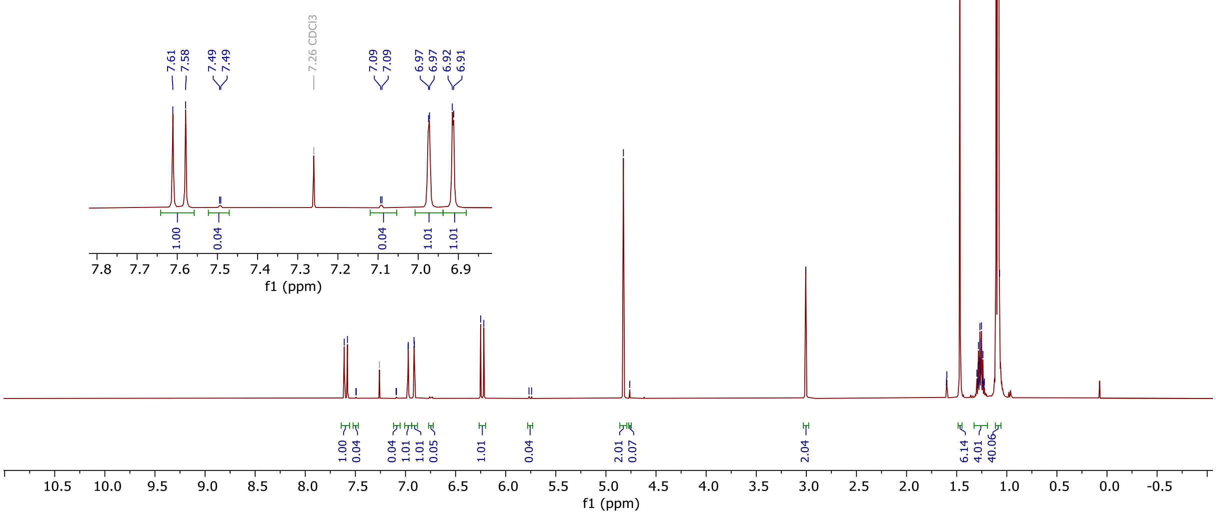

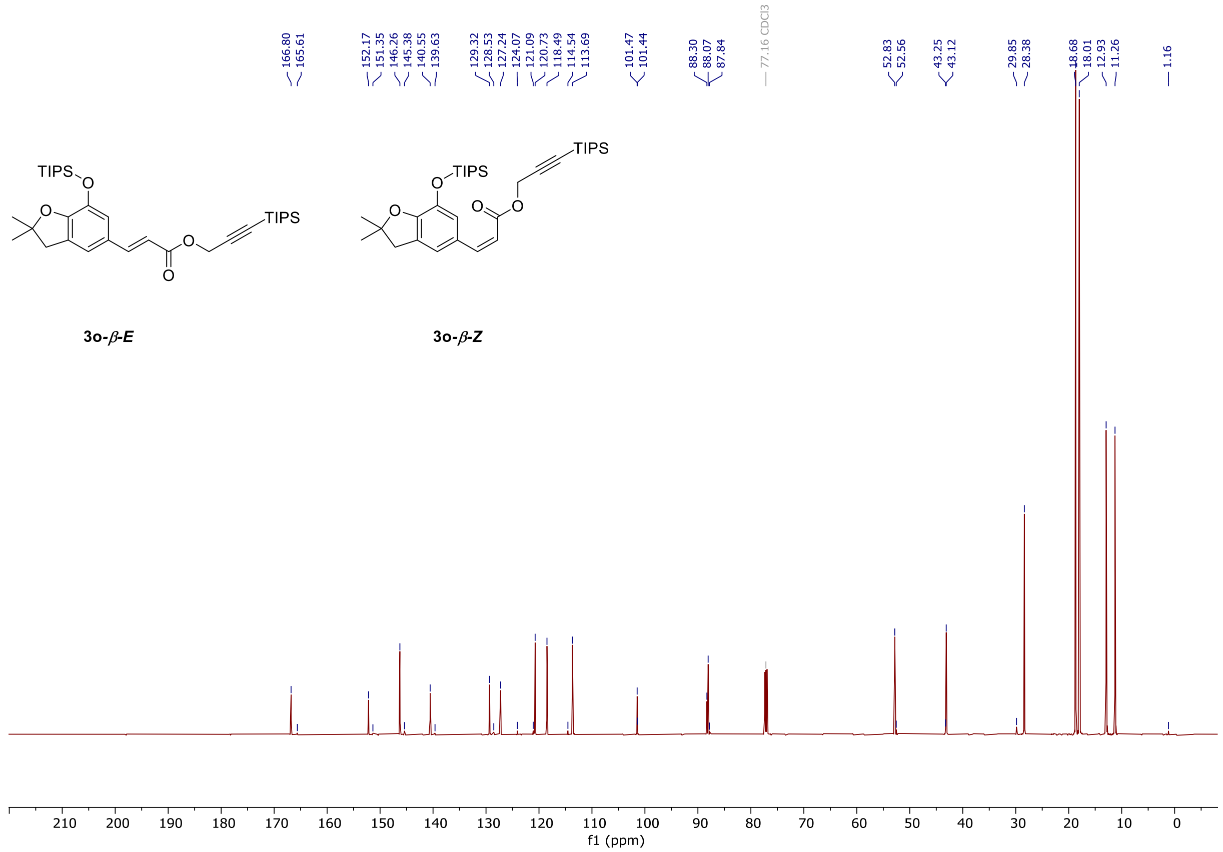
166.80
165.61
152.17
151.35
146.26
145.38
140.55
139.63
129.32
128.53
127.24
124.07
121.09
120.73
118.49
114.54
113.69
101.47
101.44
88.30
88.07
87.84
77.16 CDCl3
52.83
52.56
43.25
43.12
29.85
28.38
18.68
18.01
12.93
11.26
1.16
TIPS
O
TIPS
3o-β-E
3o-β-Z
f1 (ppm)

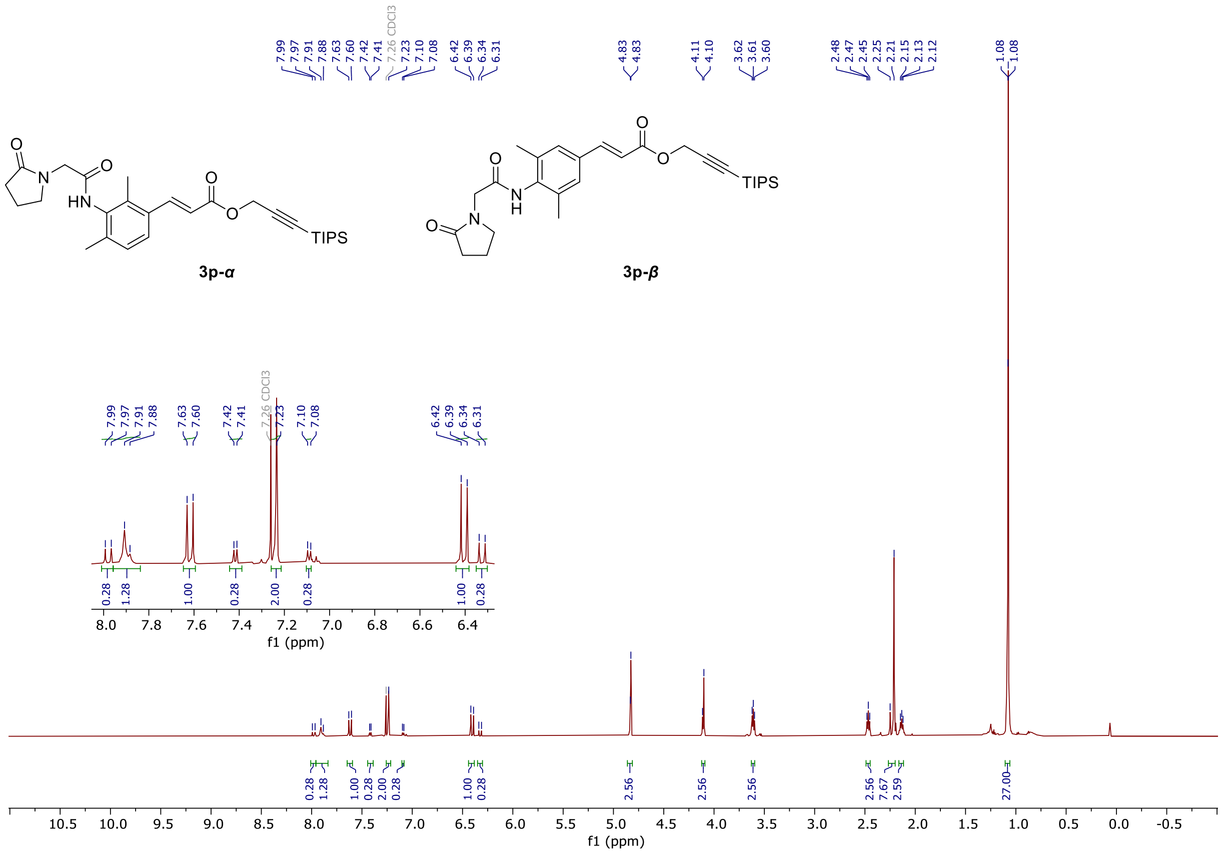
7.99
7.97
7.91
7.88
7.63
7.60
7.42
7.41
7.26 CDCl3
7.23
7.10
7.08
6.42
6.39
6.34
6.31
4.83
4.83
4.11
4.10
3.62
3.61
3.60
2.48
2.47
2.45
2.25
2.21
2.15
2.13
2.12
1.08
1.08
HN
TIPS
3p-α
3p-β
0.28
1.28
1.00
0.28
2.00
0.28
1.00
0.28
2.56
2.56
2.56
2.56
7.67
2.59
27.00
f1 (ppm)

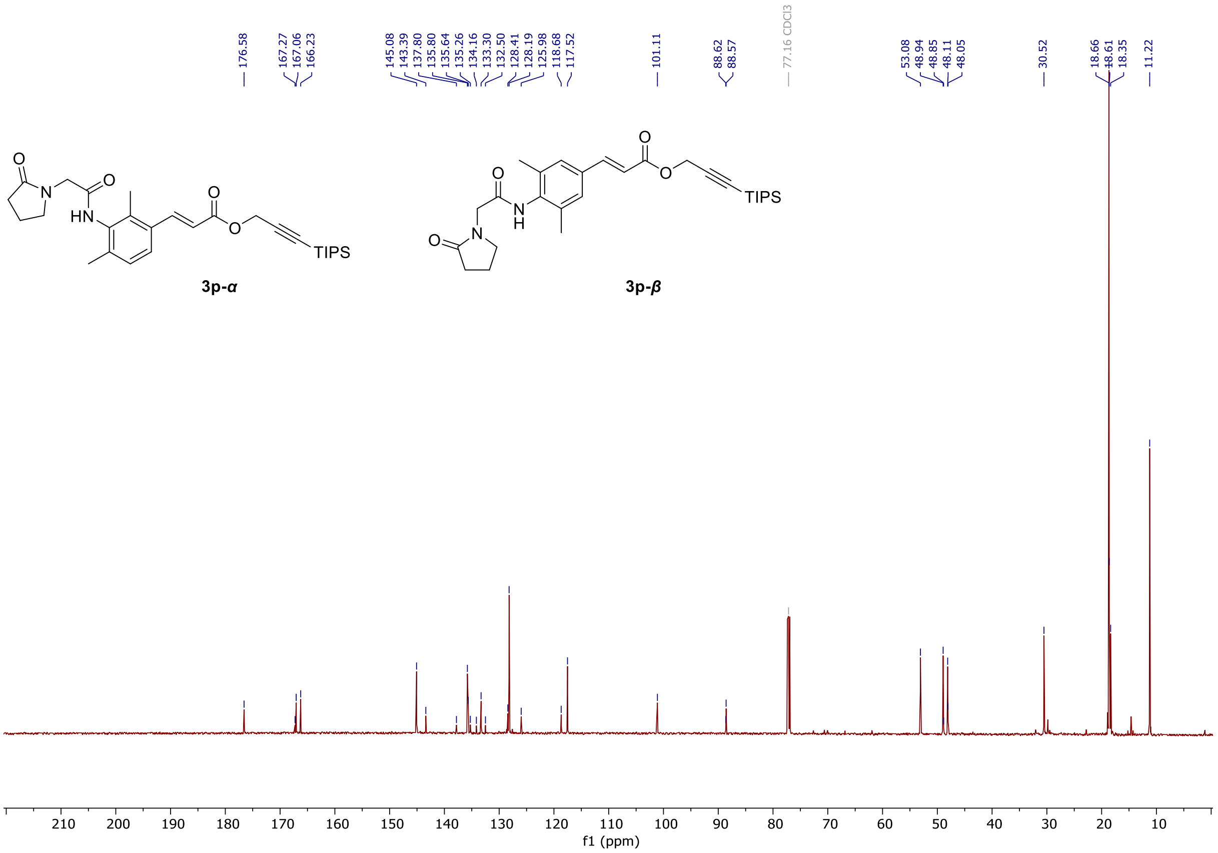
176.58
167.27
167.06
166.23
145.08
143.39
137.80
135.80
135.64
135.26
134.16
133.30
132.50
128.41
128.19
125.98
118.68
117.52
101.11
88.62
88.57
77.16 CDCl3
53.08
48.94
48.85
48.11
48.05
30.52
18.66
18.61
18.35
11.22
O
N
O
HN
O
O
TIPS
3p-α
O
N
H
O
N
O
O
TIPS
3p-β
210
200
190
180
170
160
150
140
130
120
110
100
90
80
70
60
50
40
30
20
10
f1 (ppm)

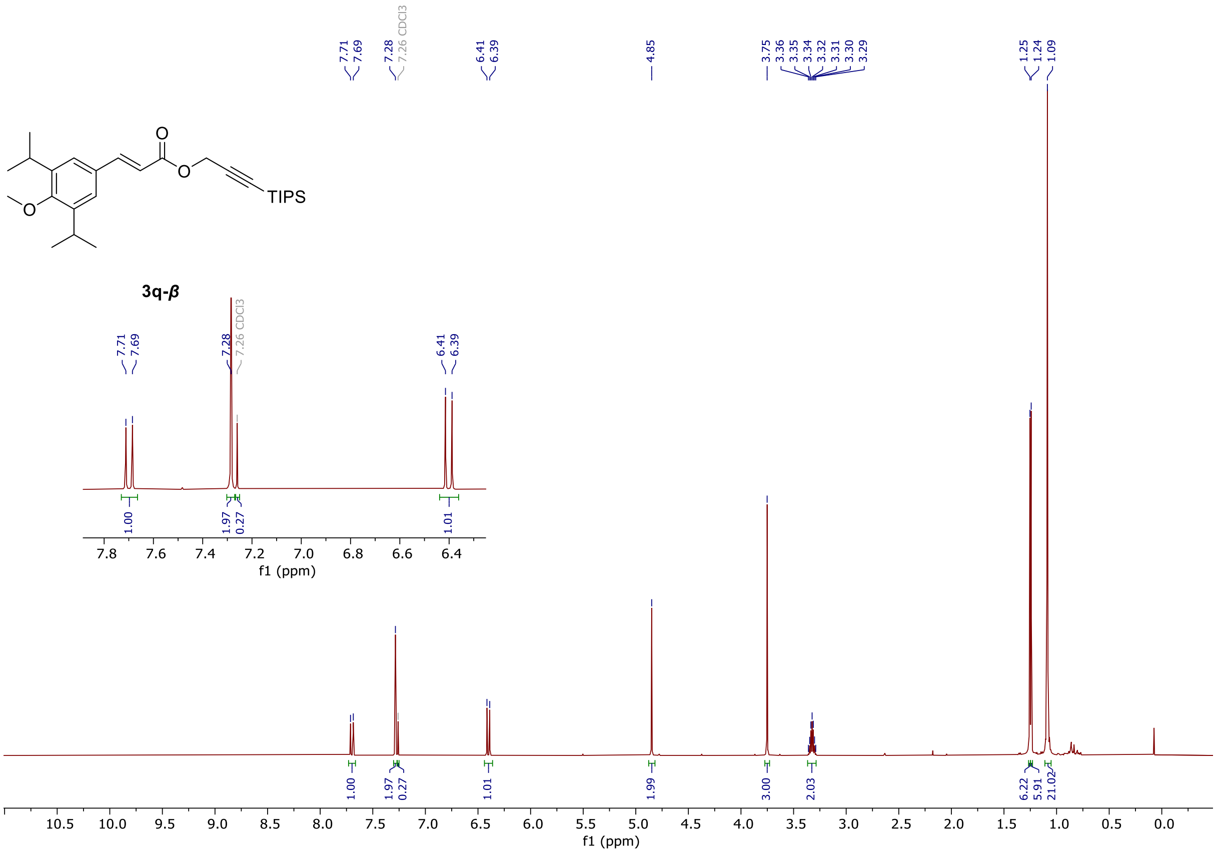
7.71
7.69
7.28
7.26 CDCl3
6.41
6.39
4.85
3.75
3.36
3.35
3.34
3.32
3.31
3.30
3.29
1.25
1.24
1.09
O
O
O
TIPS
3q-β
1.00
1.97
0.27
1.01
1.99
3.00
2.03
6.22
5.91
21.02
f1 (ppm)

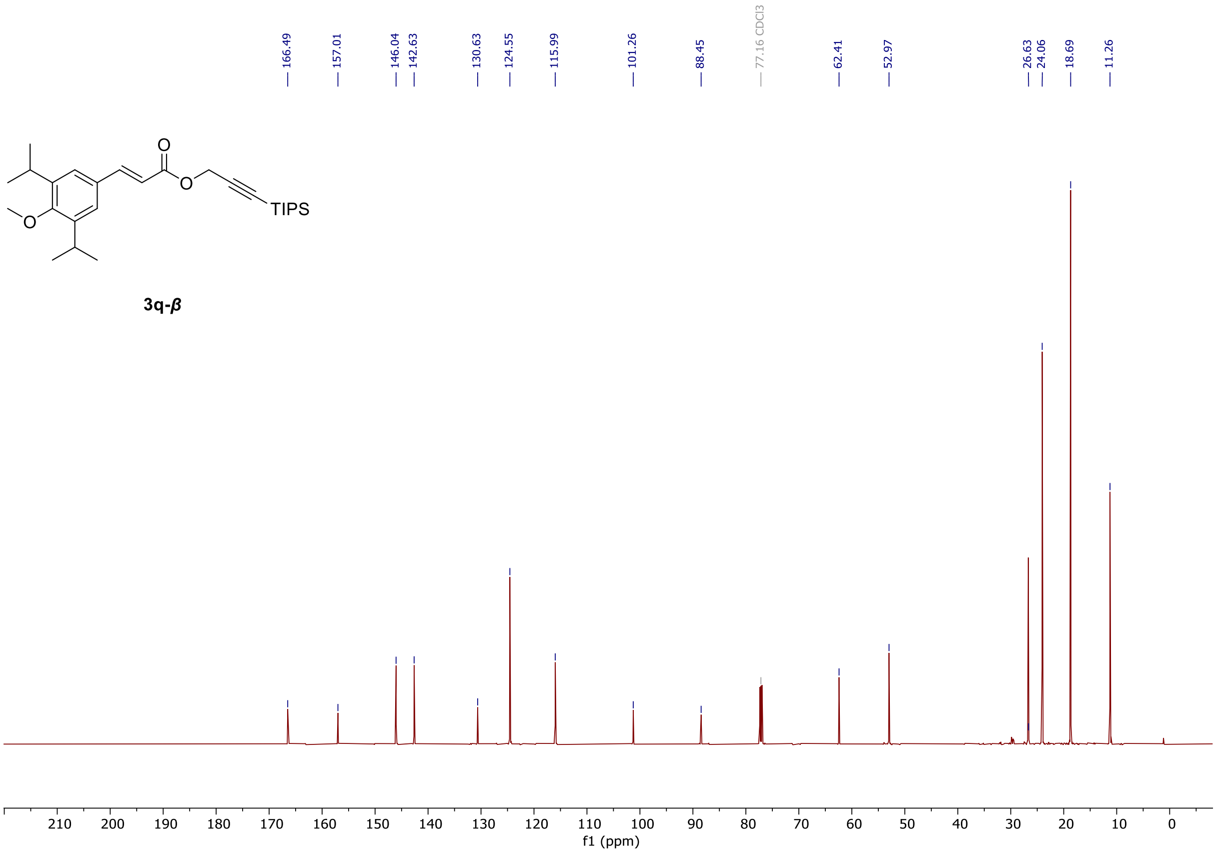
166.49
157.01
146.04
142.63
130.63
124.55
115.99
101.26
88.45
77.16 CDCl3
62.41
52.97
26.63
24.06
18.69
11.26
O
O
O
TIPS
3q-β
f1 (ppm)

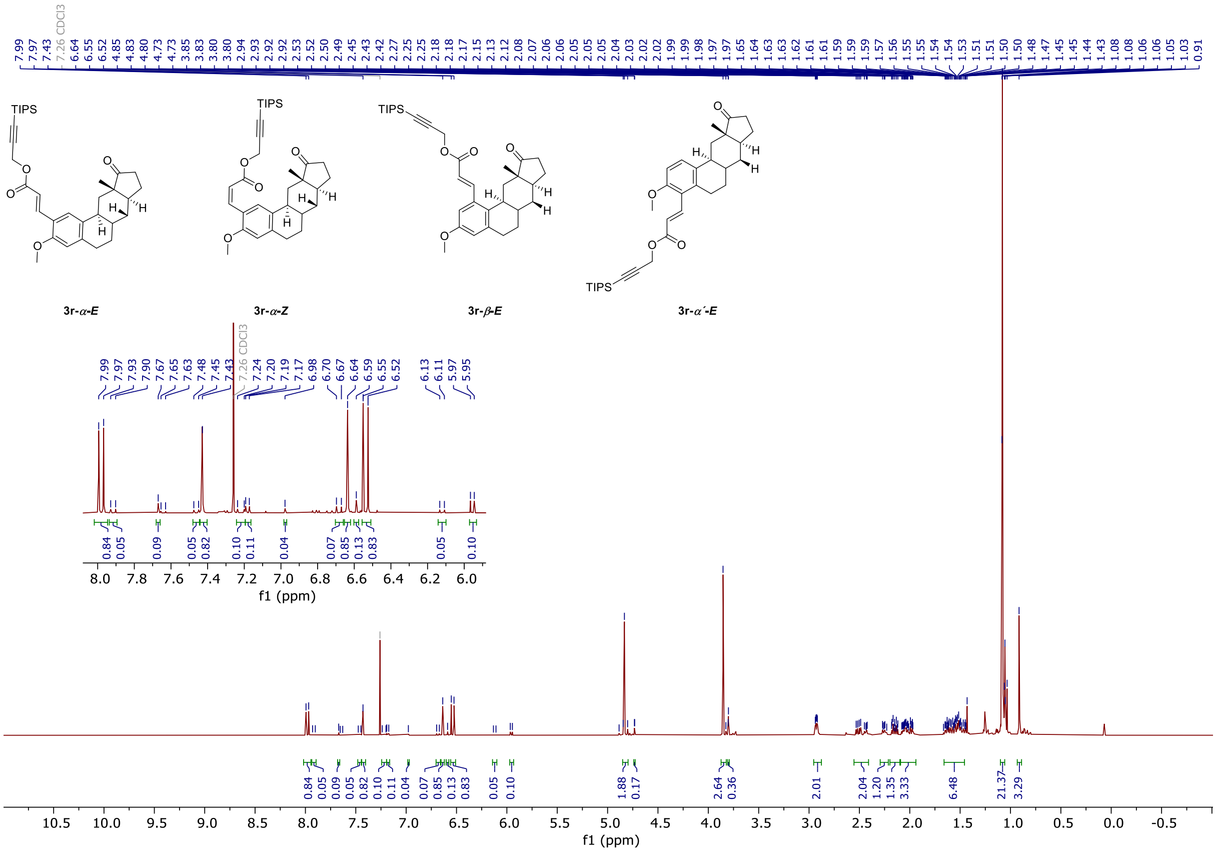
TIPS
3r-α-E
3r-α-Z
3r-β-E
3r-α′-E
f1 (ppm)

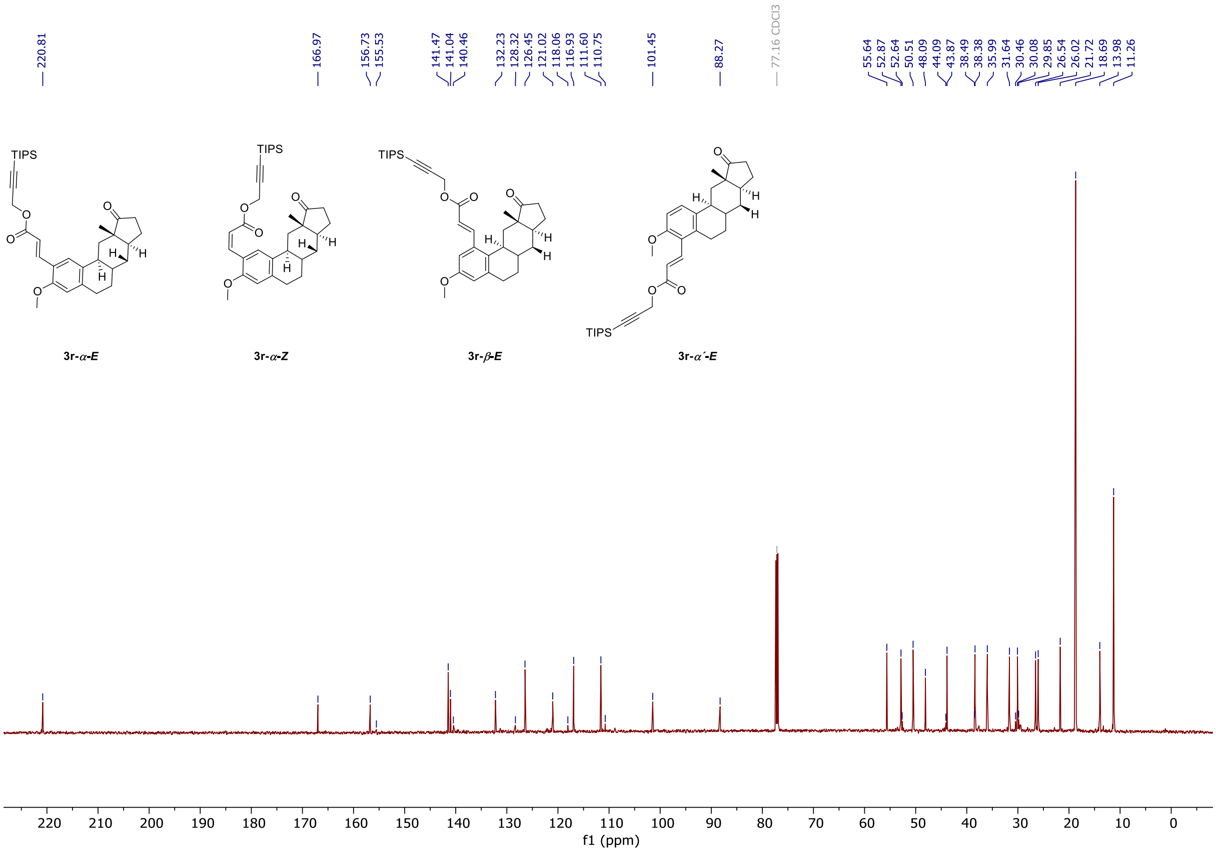
TIPS
3r-α-E
3r-α-Z
3r-β-E
3r-α′-E
f1 (ppm)

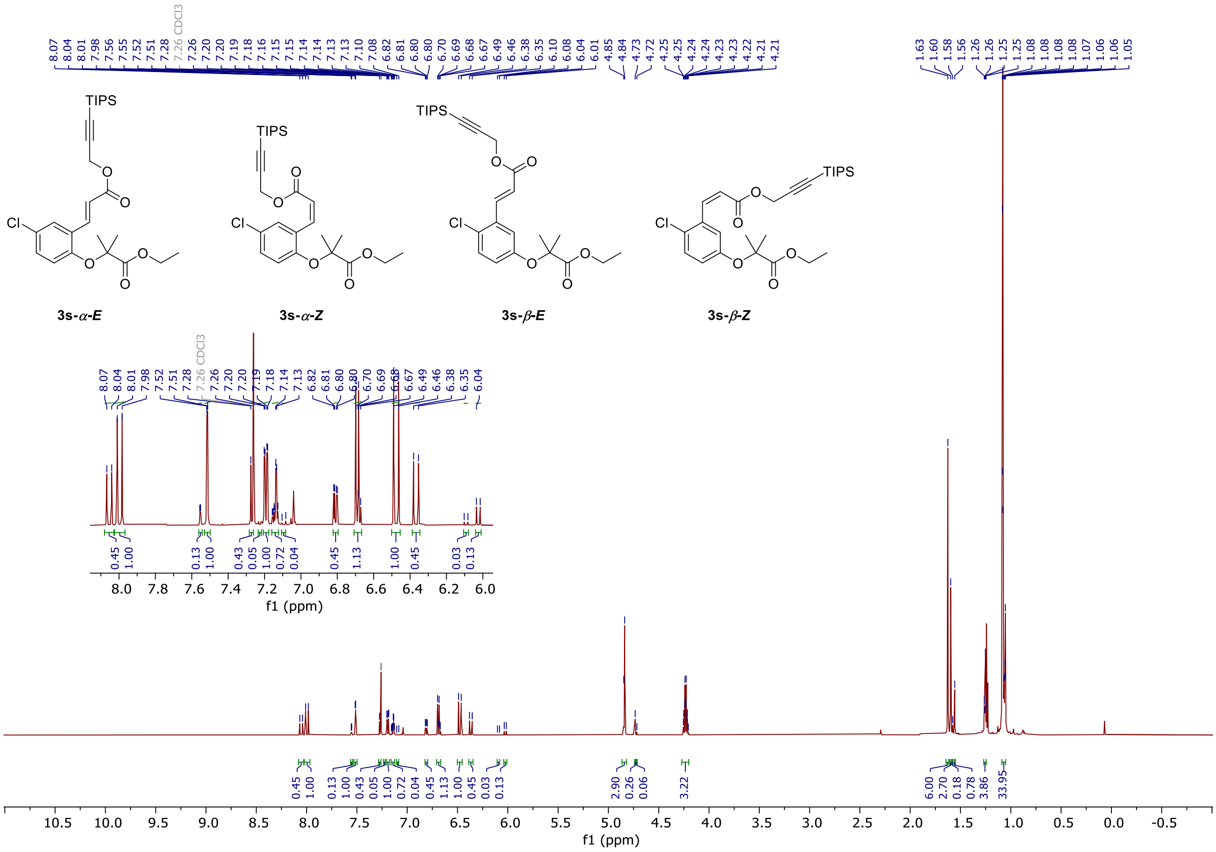
8.07 8.04 8.01 7.98 7.56 7.55 7.52 7.51 7.28 7.26 CDCl3 7.26 7.20 7.20 7.19 7.18 7.16 7.15 7.15 7.14 7.14 7.13 7.13 7.10 7.08 6.82 6.81 6.80 6.80 6.70 6.69 6.68 6.67 6.49 6.46 6.38 6.35 6.10 6.08 6.04 6.01 4.85 4.84 4.73 4.72 4.25 4.25 4.24 4.24 4.23 4.23 4.22 4.21 4.21
1.63 1.60 1.58 1.56 1.26 1.25 1.08 1.08 1.08 1.07 1.06 1.06 1.05
TIPS
Cl
O
3s-α-E
3s-α-Z
3s-β-E
3s-β-Z
0.45 1.00 0.13 1.00 0.43 0.05 1.00 0.72 0.04 0.45 1.13 1.00 0.45 0.03 0.13 2.90 0.26 0.06 3.22 6.00 2.70 0.18 0.78 3.86 33.95
f1 (ppm)

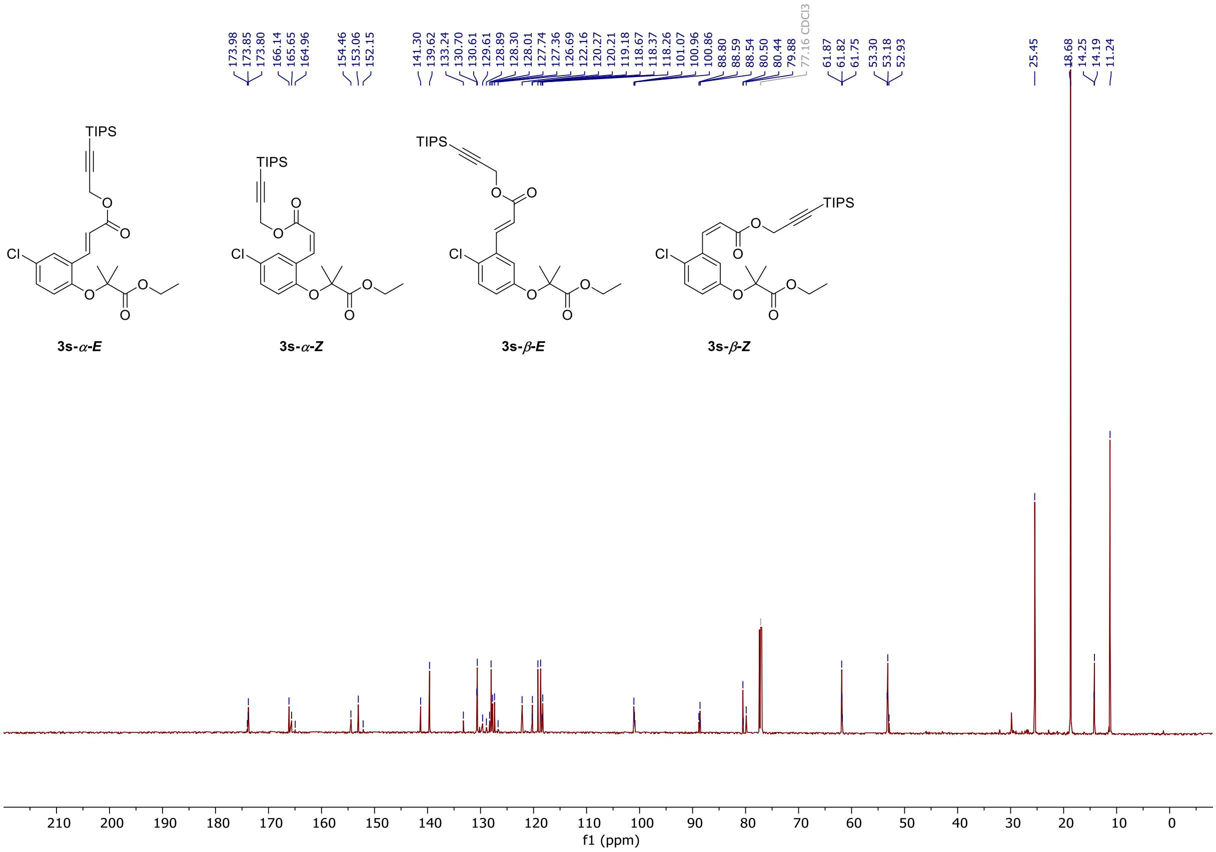
173.98 173.85 173.80 166.14 165.65 164.96 154.46 153.06 152.15 141.30 139.62 133.24 130.70 130.61 129.61 128.89 128.30 128.01 127.74 127.36 126.69 122.16 120.27 120.21 119.18 118.67 118.37 118.26 101.07 100.96 100.86 88.80 88.59 88.54 80.50 80.44 79.88 77.16 CDCl3 61.87 61.82 61.75 53.30 53.18 52.93 25.45 18.68 14.25 14.19 11.24
TIPS
Cl
O
3s-α-E
3s-α-Z
3s-β-E
3s-β-Z
f1 (ppm)

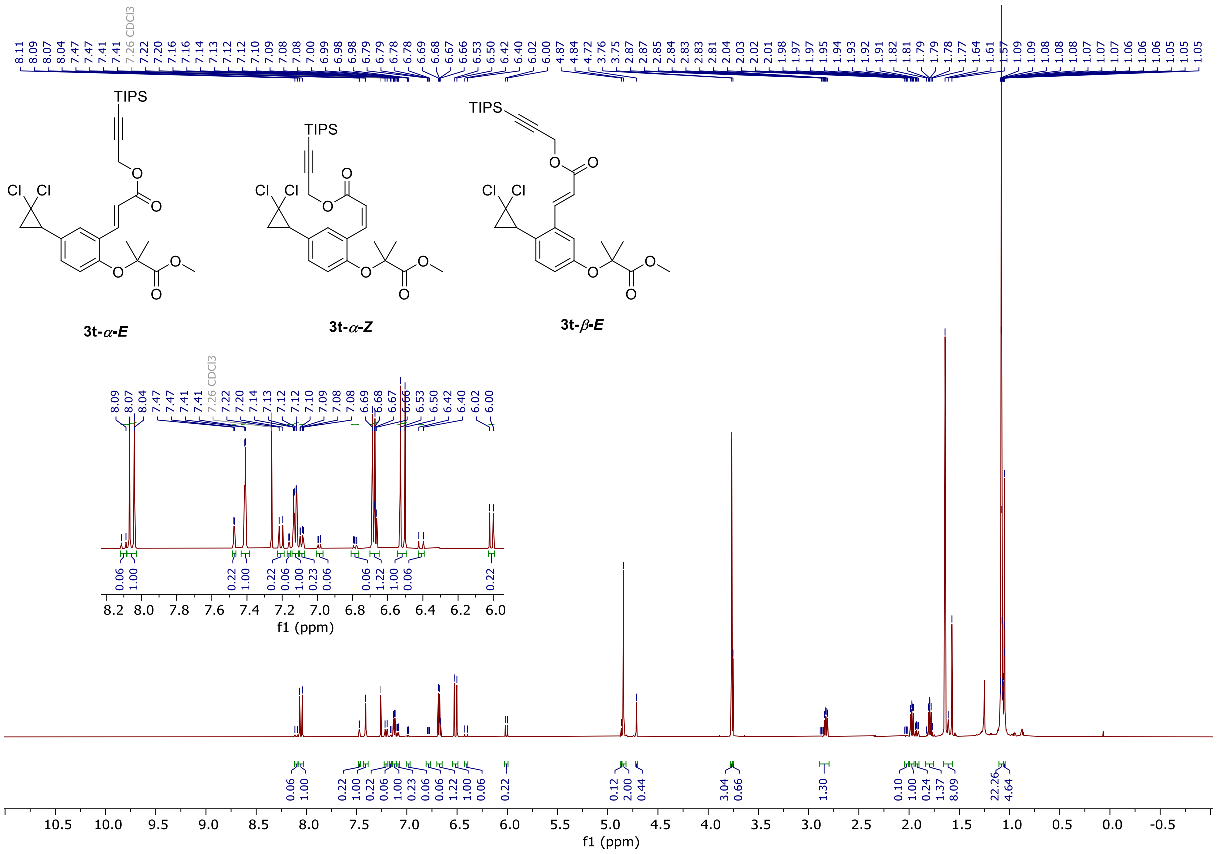
3t-α-E
3t-α-Z
3t-β-E
f1 (ppm)

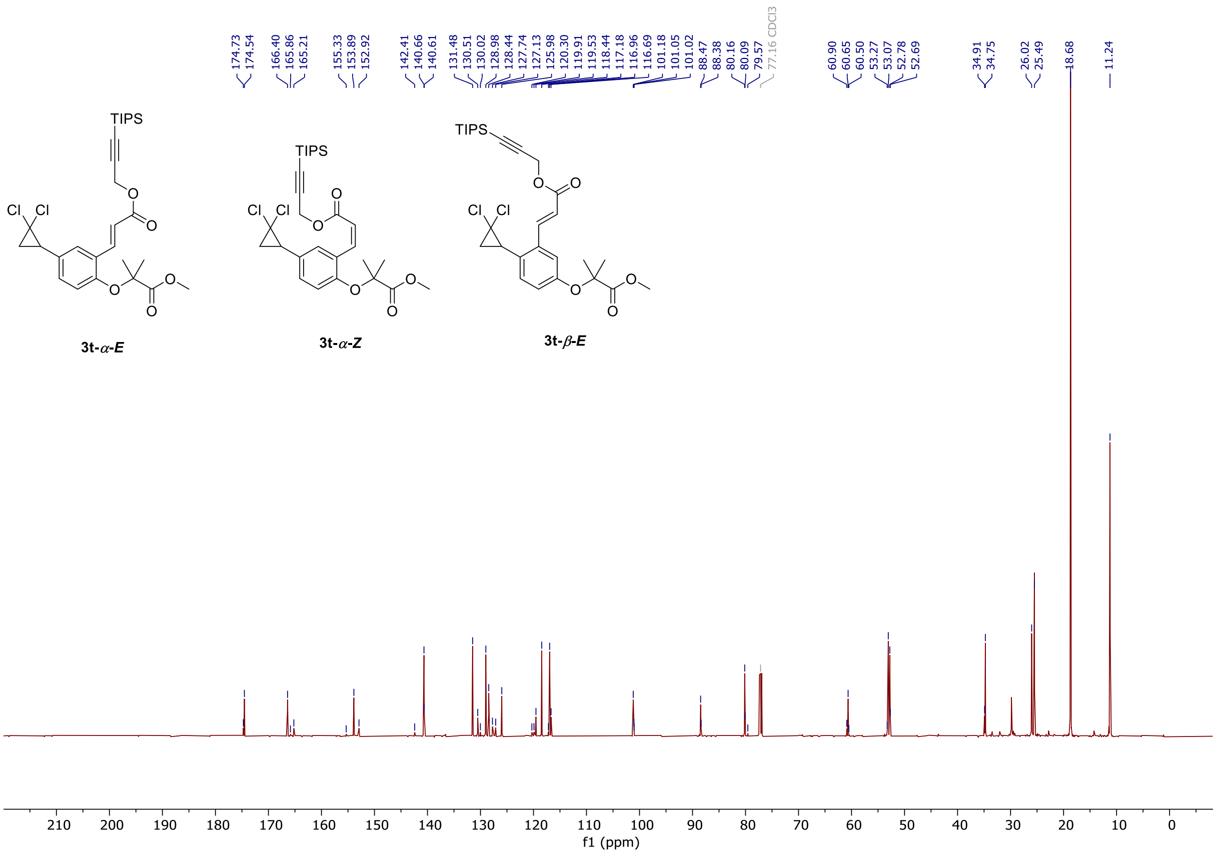
3t-α-E
3t-α-Z
3t-β-E
f1 (ppm)

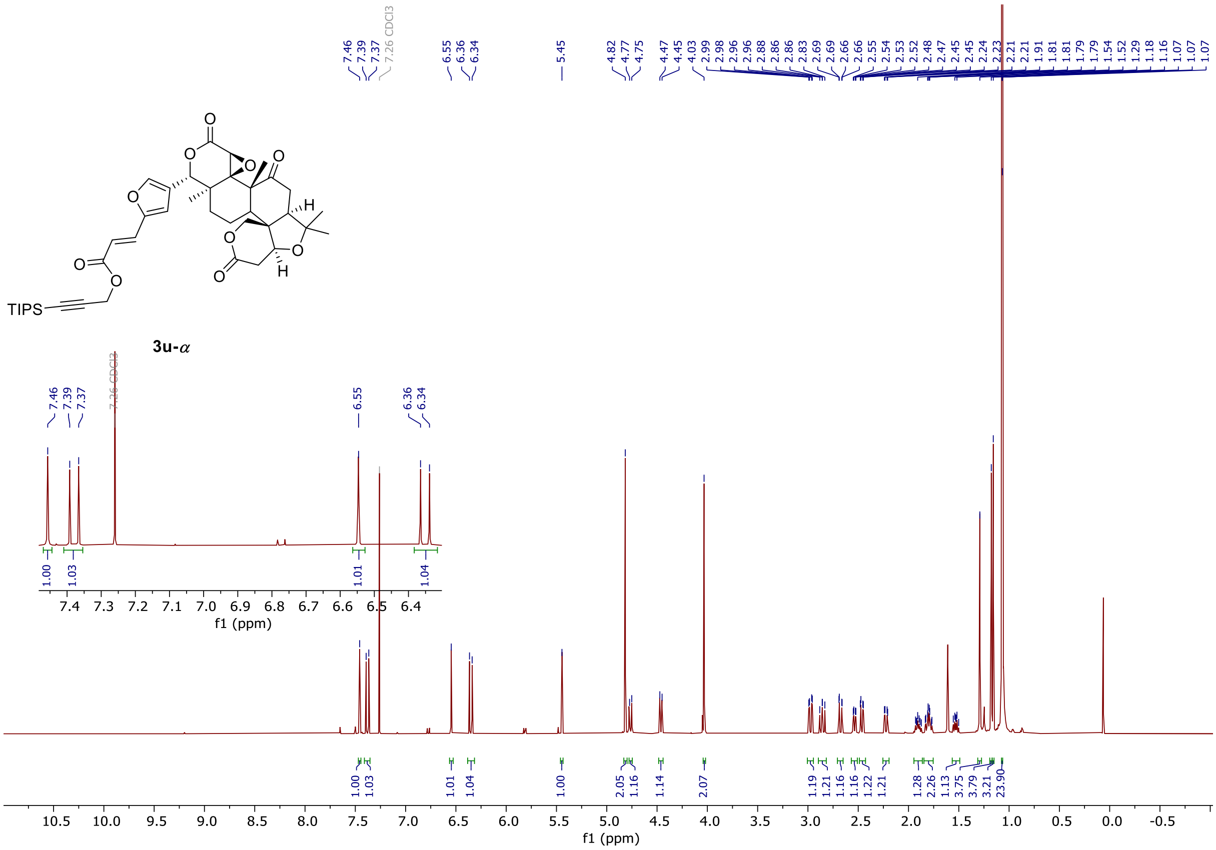
7.46 7.39 7.37 7.26 CDCl3 6.55 6.36 6.34 5.45 4.82 4.77 4.75 4.47 4.45 4.03 2.99 2.98 2.96 2.96 2.88 2.86 2.86 2.83 2.69 2.69 2.66 2.66 2.55 2.54 2.53 2.52 2.48 2.47 2.45 2.45 2.24 2.23 2.21 2.21 1.91 1.81 1.81 1.79 1.54 1.52 1.29 1.18 1.16 1.07 1.07 1.07
TIPS
3u-α
1.00 1.03 1.01 1.04
f1 (ppm)
1.00 1.03 1.01 1.04 1.00 2.05 1.16 1.14 2.07 1.19 1.21 1.16 1.16 1.22 1.21 1.28 2.26 1.13 3.75 3.79 3.21 23.90
f1 (ppm)

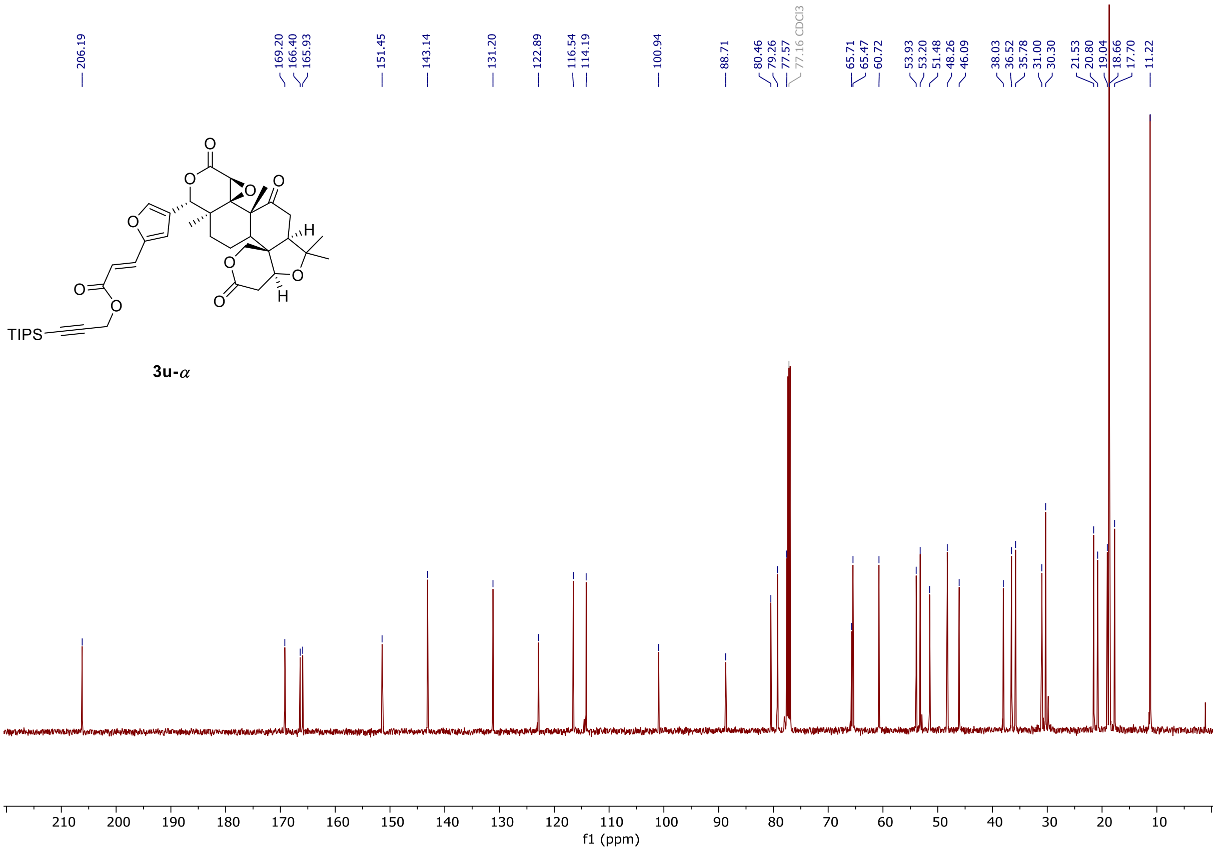
206.19 169.20 166.40 165.93 151.45 143.14 131.20 122.89 116.54 114.19 100.94 88.71 80.46 79.26 77.57 77.16 CDCl3 65.71 65.47 60.72 53.93 53.20 51.48 48.26 46.09 38.03 36.52 35.78 31.00 30.30 21.53 20.80 19.04 18.66 17.70 11.22
TIPS
3u-α
f1 (ppm)

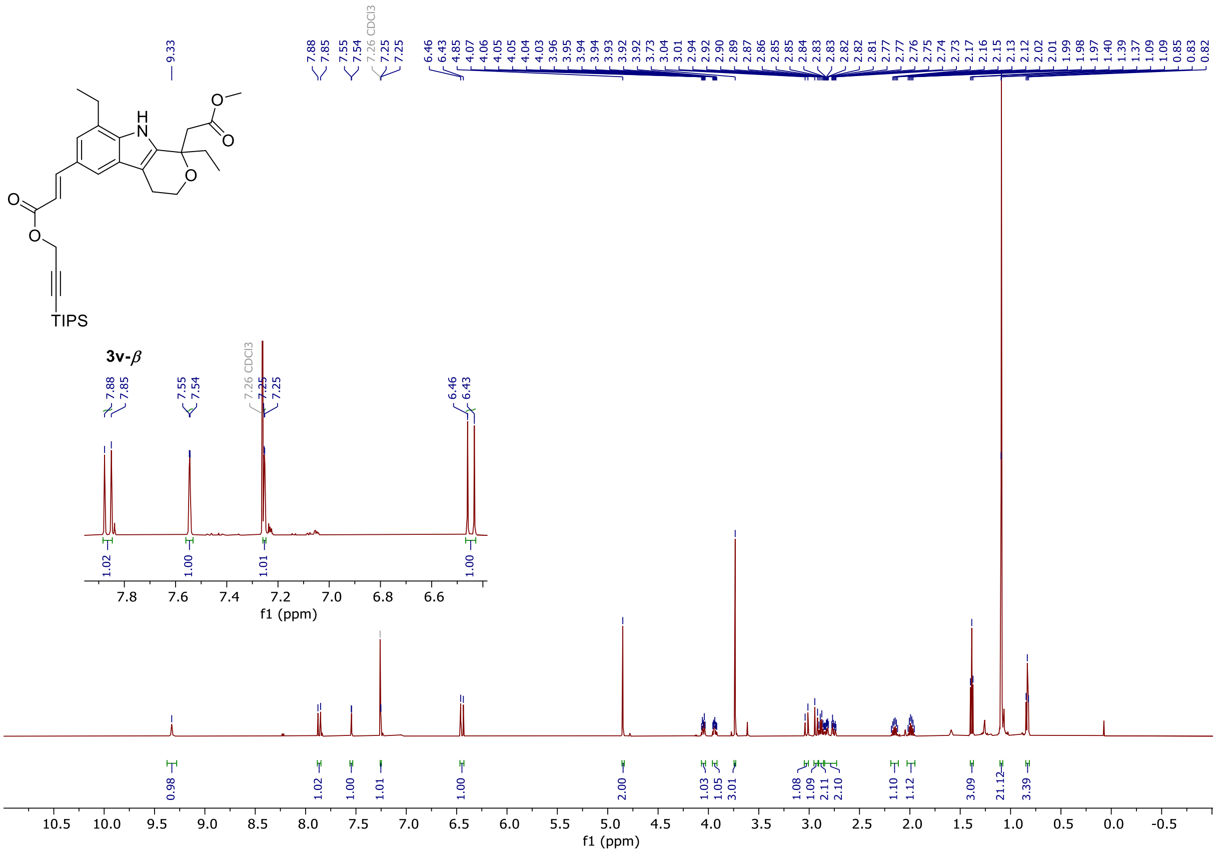
9.33
7.88 7.85 7.55 7.54 7.26 CDCl3 7.25 7.25
6.46 6.43 4.85 4.07 4.06 4.05 4.05 4.04 4.03 3.96 3.95 3.94 3.94 3.93 3.92 3.92 3.73 3.04 3.01 2.94 2.92 2.90 2.89 2.87 2.86 2.85 2.85 2.84 2.83 2.83 2.83 2.82 2.82 2.81 2.77 2.77 2.76 2.75 2.74 2.73 2.17 2.16 2.15 2.13 2.12 2.02 2.01 1.99 1.98 1.97 1.40 1.39 1.37 1.09 1.09 0.85 0.83 0.82
H
O
O
O
O
O
TIPS
3v-β
1.02 1.00 1.01 1.00
f1 (ppm)
0.98 1.02 1.00 1.01 1.00 2.00 1.03 1.05 3.01 1.08 1.09 2.11 2.10 1.10 1.12 3.09 21.12 3.39
f1 (ppm)

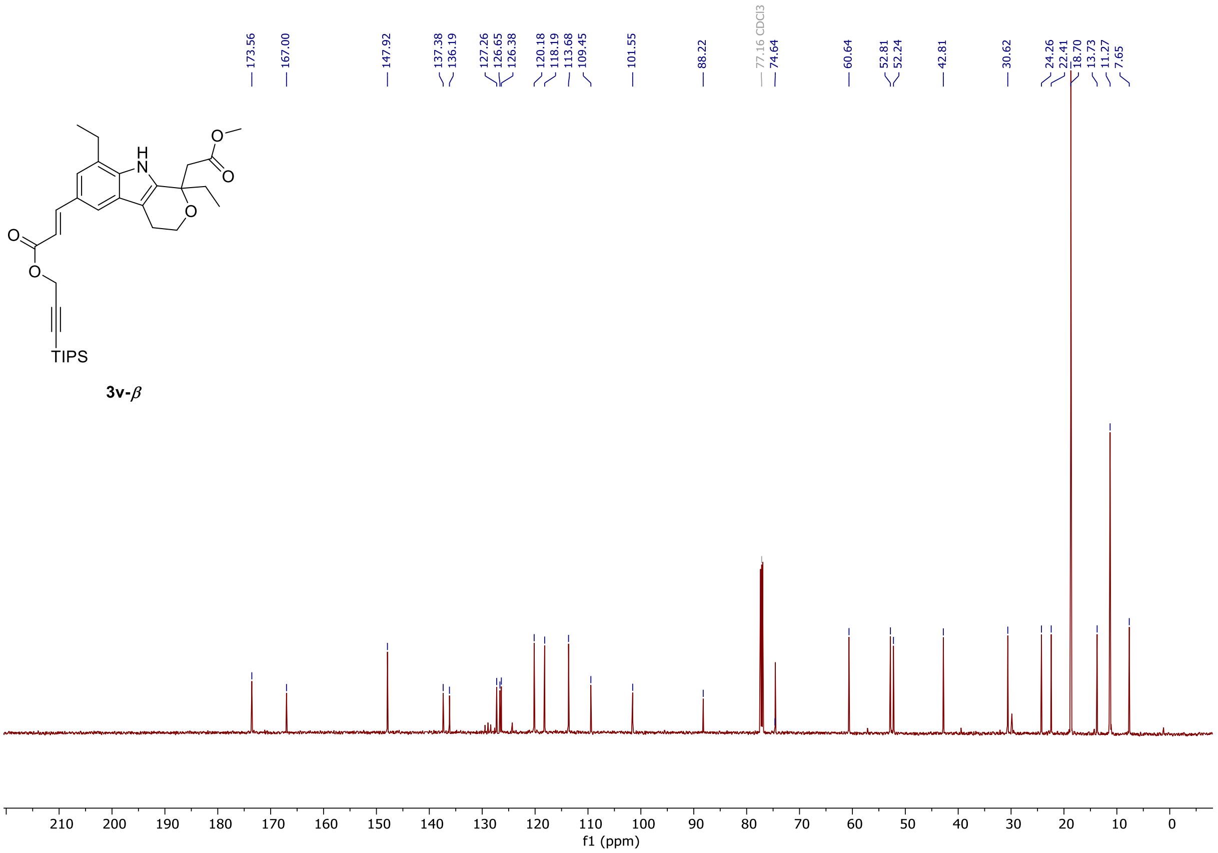
173.56 167.00 147.92 137.38 136.19 127.26 126.65 126.38 120.18 118.19 113.68 109.45 101.55 88.22 77.16 CDCl3 74.64 60.64 52.81 52.24 42.81 30.62 24.26 22.41 18.70 13.73 11.27 7.65
H
O
O
O
O
O
TIPS
3v-β
f1 (ppm)

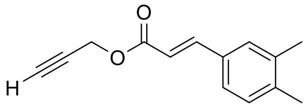

4

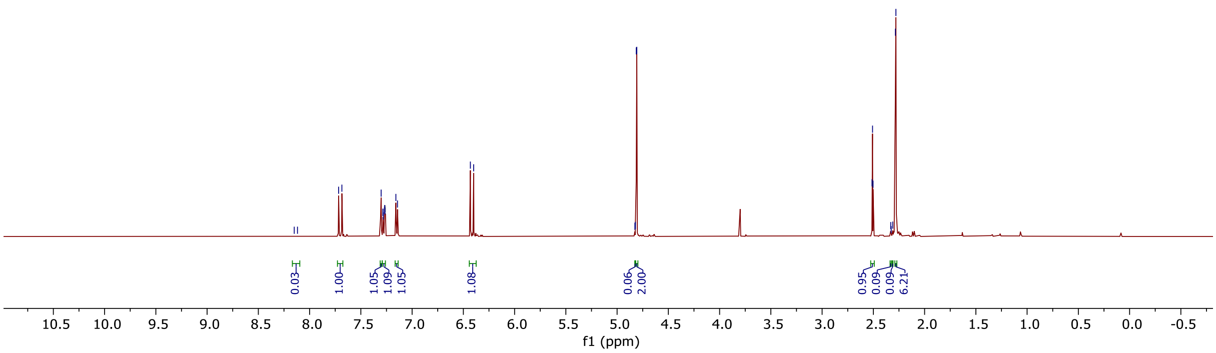
8.15
8.12
7.72
7.68
7.30
7.28
7.28
7.28
7.27
7.27
7.26
7.26 CDCl3
7.16
7.14
6.43
6.40
4.83
4.82
4.81
4.81
2.51
2.51
2.50
2.33
2.33
2.31
2.28
2.28
0.03
1.00
1.05
1.09
1.05
1.08
0.06
2.00
0.95
0.09
0.09
6.21
10.5 10.0 9.5 9.0 8.5 8.0 7.5 7.0 6.5 6.0 5.5 5.0 4.5 4.0 3.5 3.0 2.5 2.0 1.5 1.0 0.5 0.0 -0.5
f1 (ppm)

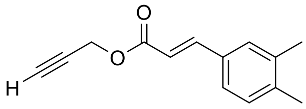

4

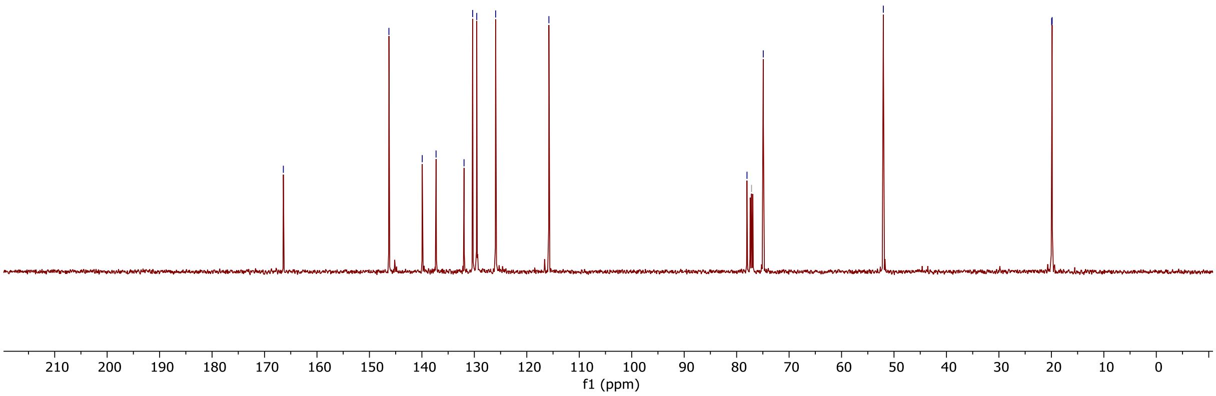
166.41
146.29
139.93
137.30
131.97
130.32
129.54
125.94
115.79
78.03
77.16 CDCl3
74.93
52.04
19.95
19.86
210 200 190 180 170 160 150 140 130 120 110 100 90 80 70 60 50 40 30 20 10 0
f1 (ppm)

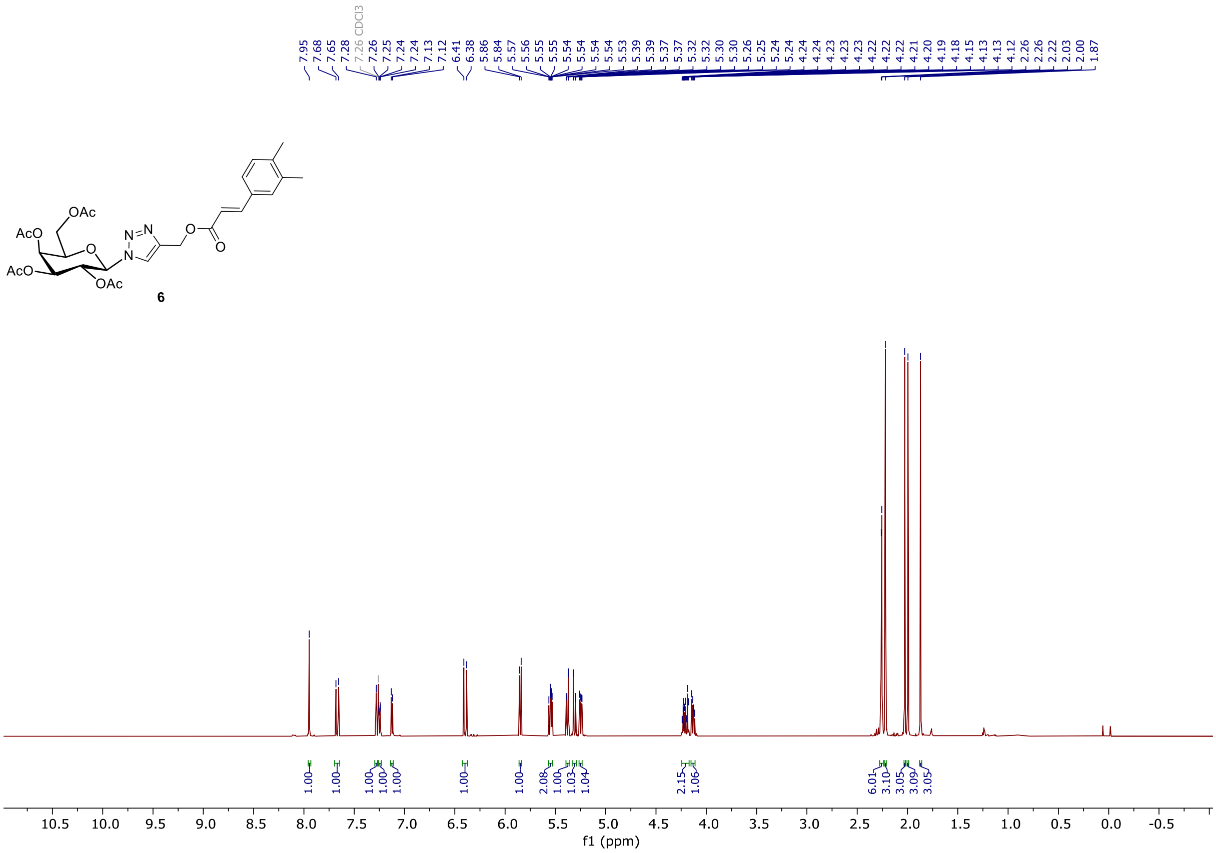
7.95
7.68
7.65
7.28
7.26 CDCl3
7.26
7.25
7.24
7.24
7.13
7.12
6.41
6.38
5.86
5.84
5.57
5.56
5.55
5.55
5.54
5.54
5.54
5.53
5.39
5.39
5.37
5.37
5.32
5.30
5.30
5.26
5.25
5.24
5.24
4.24
4.24
4.23
4.23
4.23
4.22
4.22
4.22
4.21
4.20
4.19
4.18
4.15
4.13
4.13
4.12
2.26
2.26
2.22
2.03
2.00
1.87
AcO
OAc
AcO
OAc
O
N
N=N
O
O
6
1.00
1.00
1.00
1.00
1.00
1.00
1.00
2.08
1.00
1.03
1.04
2.15
1.06
6.01
3.10
3.05
3.09
3.05
f1 (ppm)

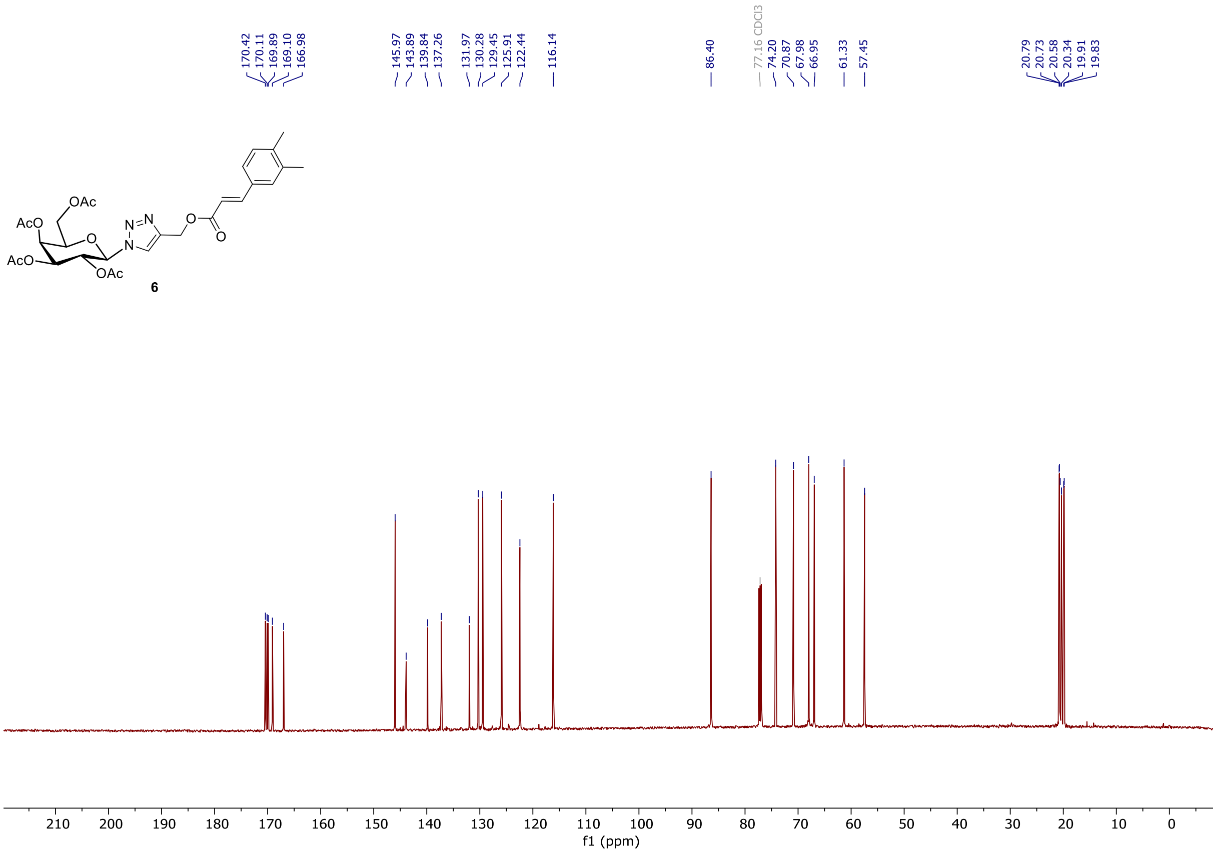
170.42
170.11
169.89
169.10
166.98
145.97
143.89
139.84
137.26
131.97
130.28
129.45
125.91
122.44
116.14
86.40
77.16 CDCl3
74.20
70.87
67.98
66.95
61.33
57.45
20.79
20.73
20.58
20.34
19.91
19.83
AcO
OAc
AcO
OAc
O
N
N=N
O
O
6
f1 (ppm)

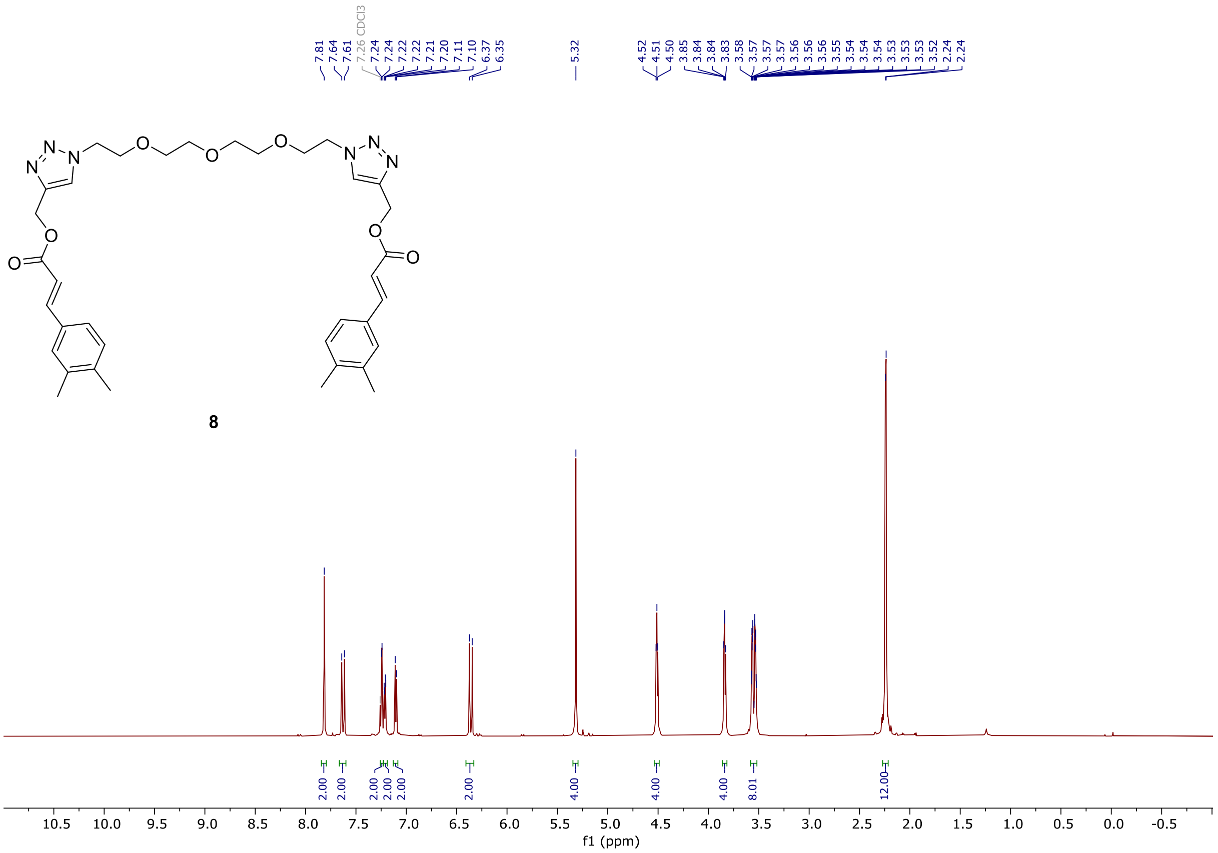
7.81
7.64
7.61
7.26 CDCl3
7.24
7.24
7.22
7.22
7.21
7.20
7.11
7.10
6.37
6.35
5.32
4.52
4.51
4.50
3.85
3.84
3.84
3.83
3.58
3.57
3.57
3.57
3.56
3.56
3.55
3.54
3.54
3.54
3.53
3.53
3.53
3.52
2.24
2.24
8
2.00
2.00
2.00
2.00
2.00
2.00
4.00
4.00
4.00
8.01
12.00
10.5 10.0 9.5 9.0 8.5 8.0 7.5 7.0 6.5 6.0 5.5 5.0 4.5 4.0 3.5 3.0 2.5 2.0 1.5 1.0 0.5 0.0 -0.5
f1 (ppm)

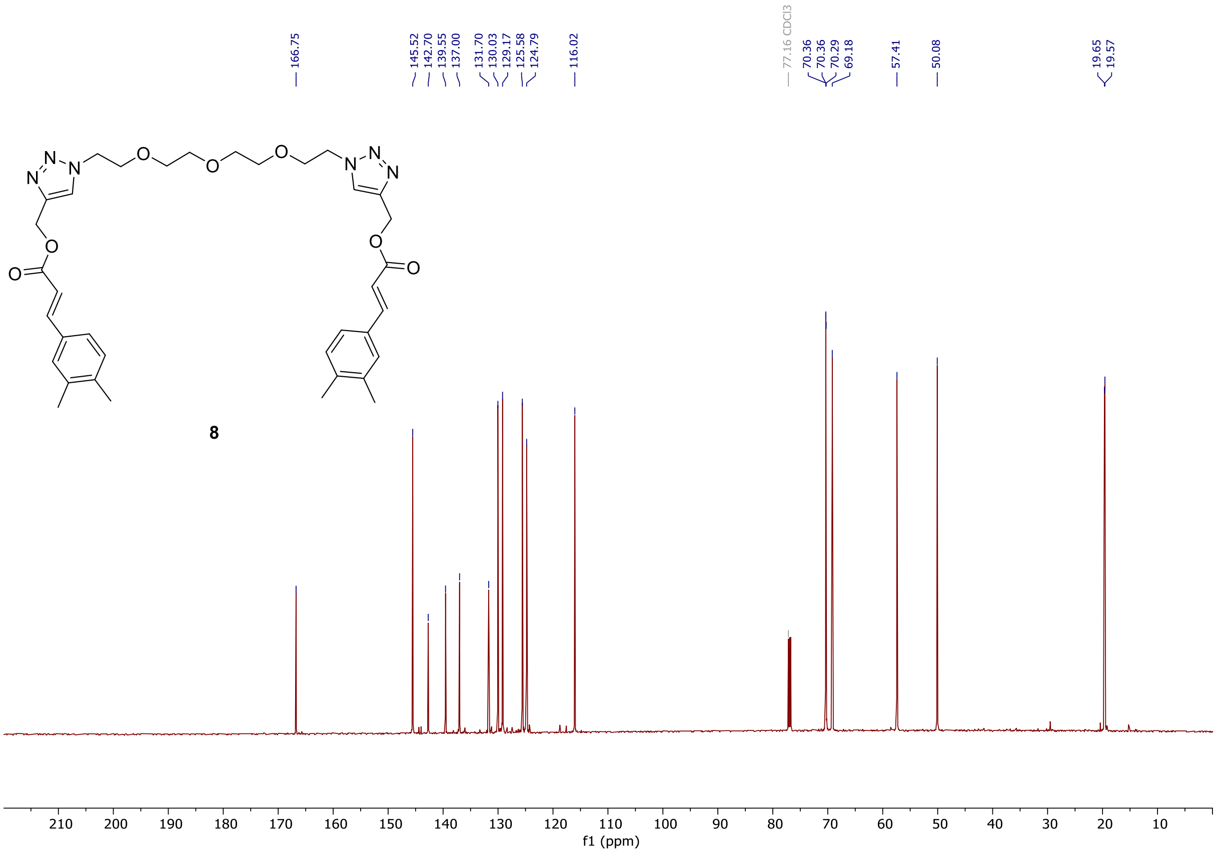
166.75
145.52
142.70
139.55
137.00
131.70
130.03
129.17
125.58
124.79
116.02
77.16 CDCl3
70.36
70.36
70.29
69.18
57.41
50.08
19.65
19.57
8
210 200 190 180 170 160 150 140 130 120 110 100 90 80 70 60 50 40 30 20 10
f1 (ppm)

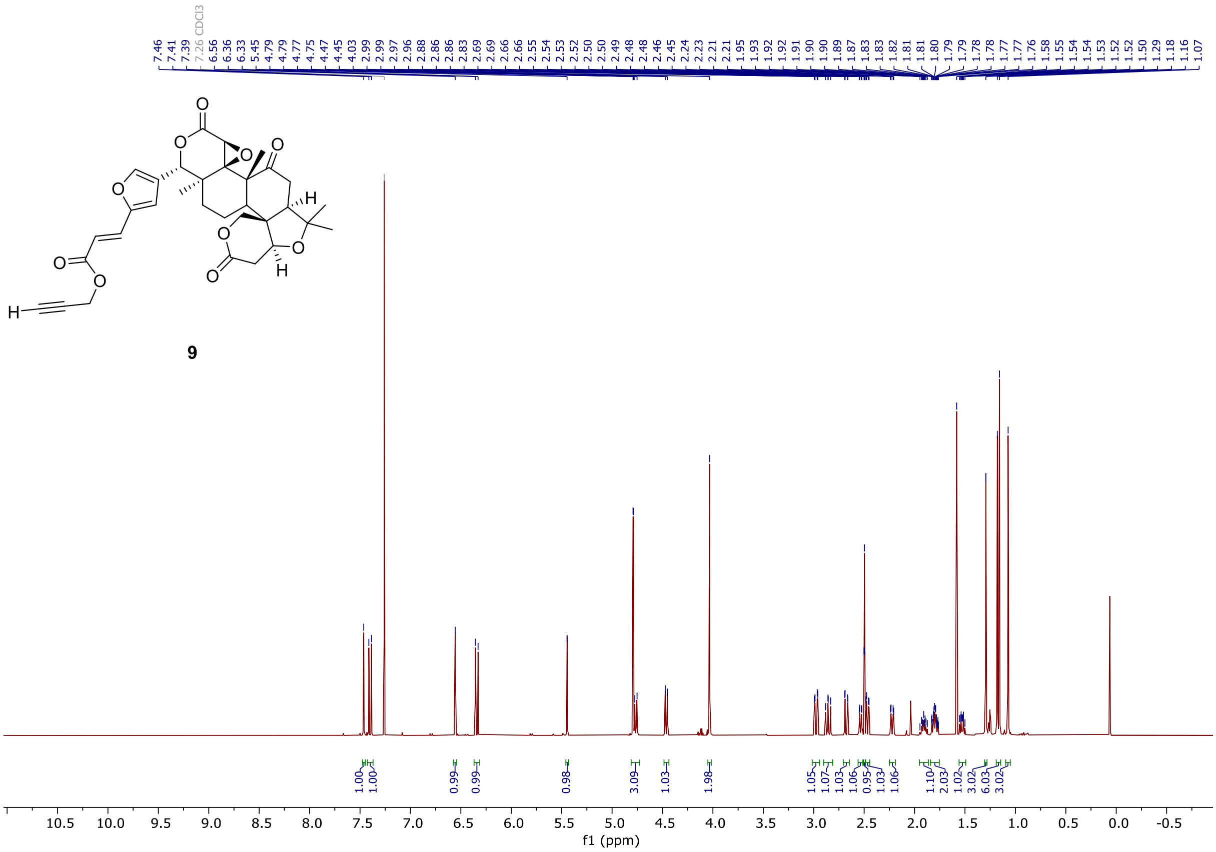
7.46 7.41 7.39 7.26 CDCl3 6.56 6.36 6.33 5.45 4.79 4.79 4.77 4.75 4.47 4.45 4.03 2.99 2.99 2.97 2.96 2.88 2.86 2.86 2.83 2.69 2.69 2.66 2.66 2.55 2.54 2.53 2.52 2.50 2.50 2.49 2.48 2.48 2.46 2.45 2.24 2.23 2.21 2.21 1.95 1.93 1.93 1.92 1.92 1.91 1.90 1.89 1.87 1.83 1.83 1.82 1.81 1.81 1.80 1.79 1.79 1.78 1.78 1.77 1.77 1.76 1.58 1.55 1.54 1.54 1.53 1.52 1.52 1.50 1.29 1.18 1.16 1.07
9
1.00 1.00 0.99 0.99 0.98 3.09 1.03 1.98 1.05 1.07 1.03 1.06 0.95 1.03 1.06 1.10 2.03 1.02 3.02 6.03 3.02
10.5 10.0 9.5 9.0 8.5 8.0 7.5 7.0 6.5 6.0 5.5 5.0 4.5 4.0 3.5 3.0 2.5 2.0 1.5 1.0 0.5 0.0 -0.5
f1 (ppm)

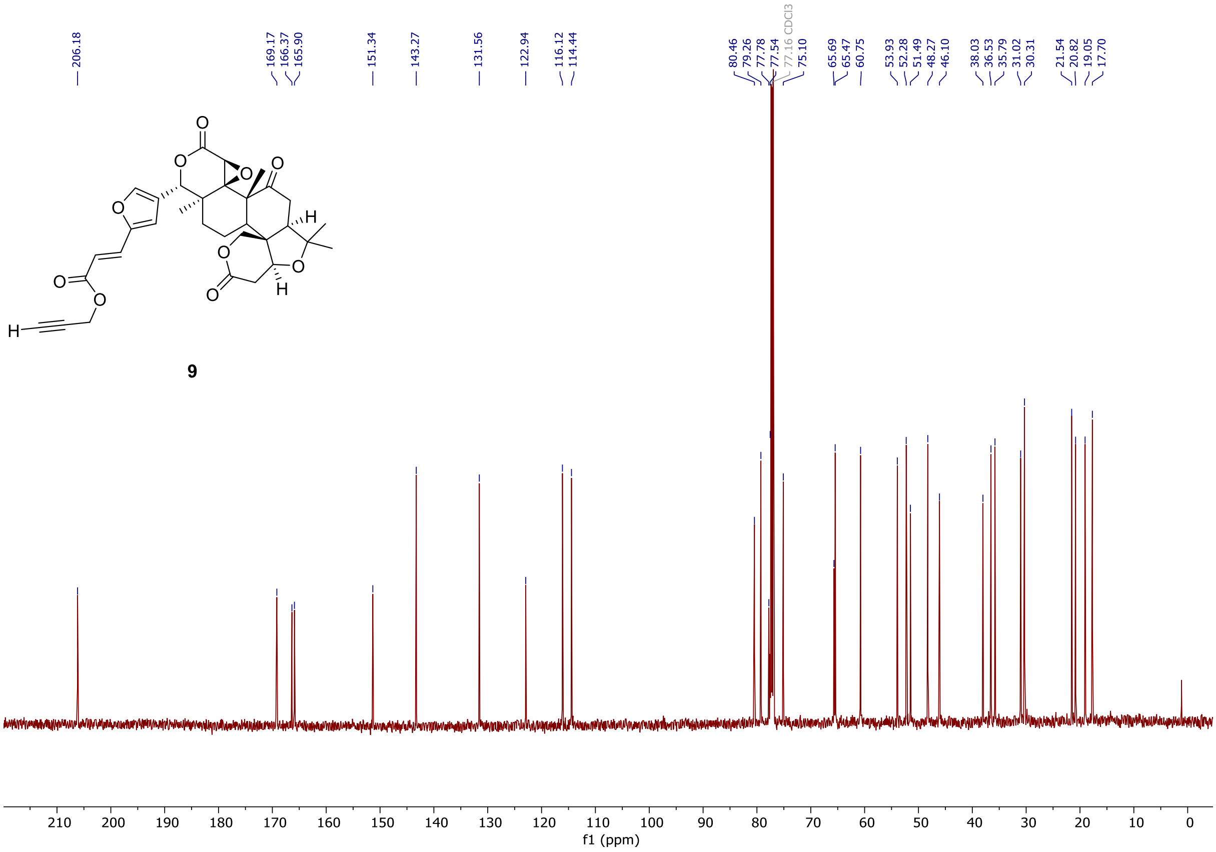
206.18 169.17 166.37 165.90 151.34 143.27 131.56 122.94 116.12 114.44 80.46 79.26 77.78 77.54 77.16 CDCl3 75.10 65.69 65.47 60.75 53.93 52.28 51.49 48.27 46.10 38.03 36.53 35.79 31.02 30.31 21.54 20.82 19.05 17.70
9
210 200 190 180 170 160 150 140 130 120 110 100 90 80 70 60 50 40 30 20 10 0
f1 (ppm)

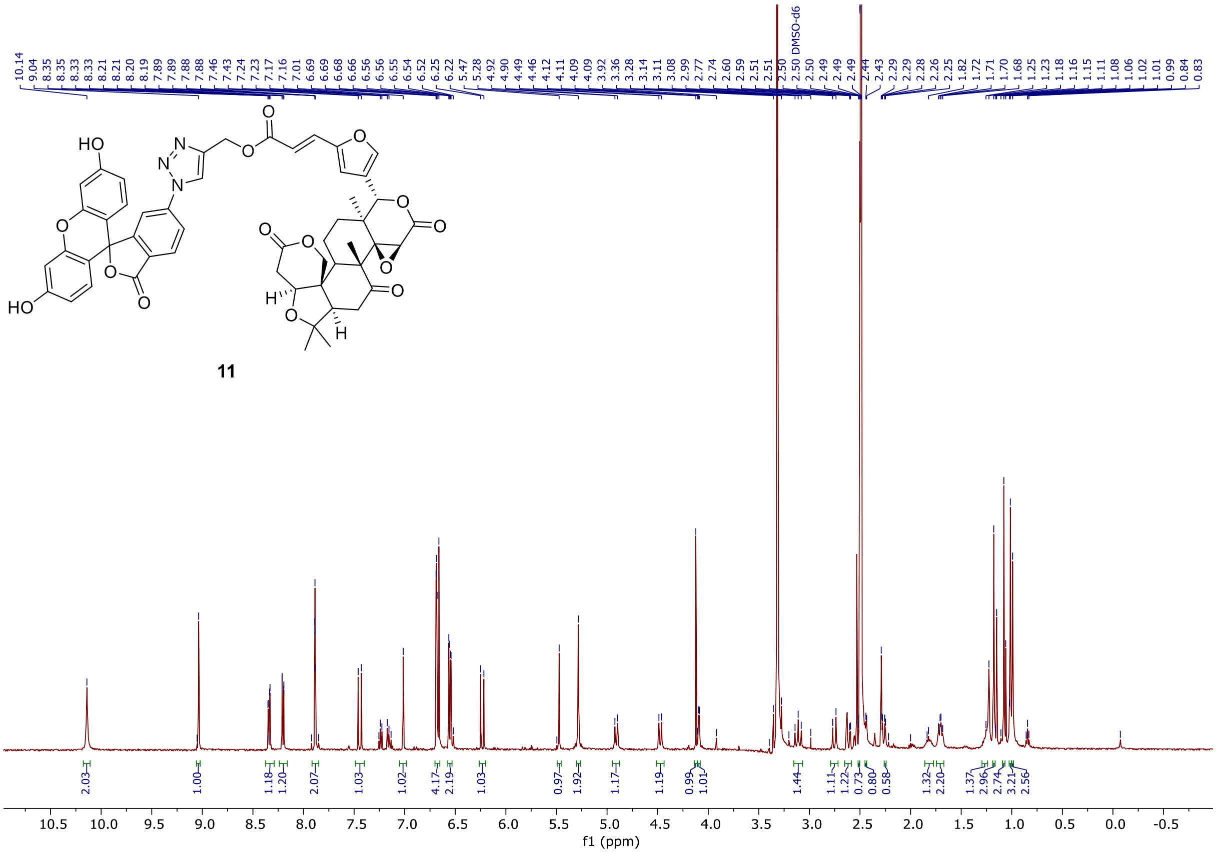
10.14 9.04 8.35 8.35 8.33 8.33 8.21 8.21 8.20 8.19 7.89 7.89 7.88 7.88 7.46 7.43 7.24 7.23 7.17 7.16 7.01 6.69 6.69 6.68 6.66 6.56 6.56 6.55 6.54 6.52 6.25 6.22 5.47 5.28 4.92 4.90 4.49 4.46 4.12 4.11 4.09 4.09 3.92 3.36 3.28 3.14 3.11 3.08 2.99 2.77 2.74 2.60 2.59 2.51 2.51 2.50 2.50 DMSO-d6 2.50 2.49 2.49 2.49 2.44 2.43 2.29 2.28 2.26 2.25 1.82 1.72 1.71 1.70 1.68 1.25 1.23 1.18 1.16 1.15 1.11 1.08 1.06 1.02 1.01 0.99 0.84 0.83
HO
N
N
N
O
O
O
O
HO
O
O
O
O
O
O
O
H
O
H
O
O
11
2.03
1.00
1.18
1.20
2.07
1.03
1.02
4.17
2.19
1.03
0.97
1.92
1.17
1.19
0.99
1.01
1.44
1.11
1.22
0.73
0.80
0.58
1.32
2.20
1.37
2.96
2.74
3.21
2.56
10.5 10.0 9.5 9.0 8.5 8.0 7.5 7.0 6.5 6.0 5.5 5.0 4.5 4.0 3.5 3.0 2.5 2.0 1.5 1.0 0.5 0.0 -0.5
f1 (ppm)

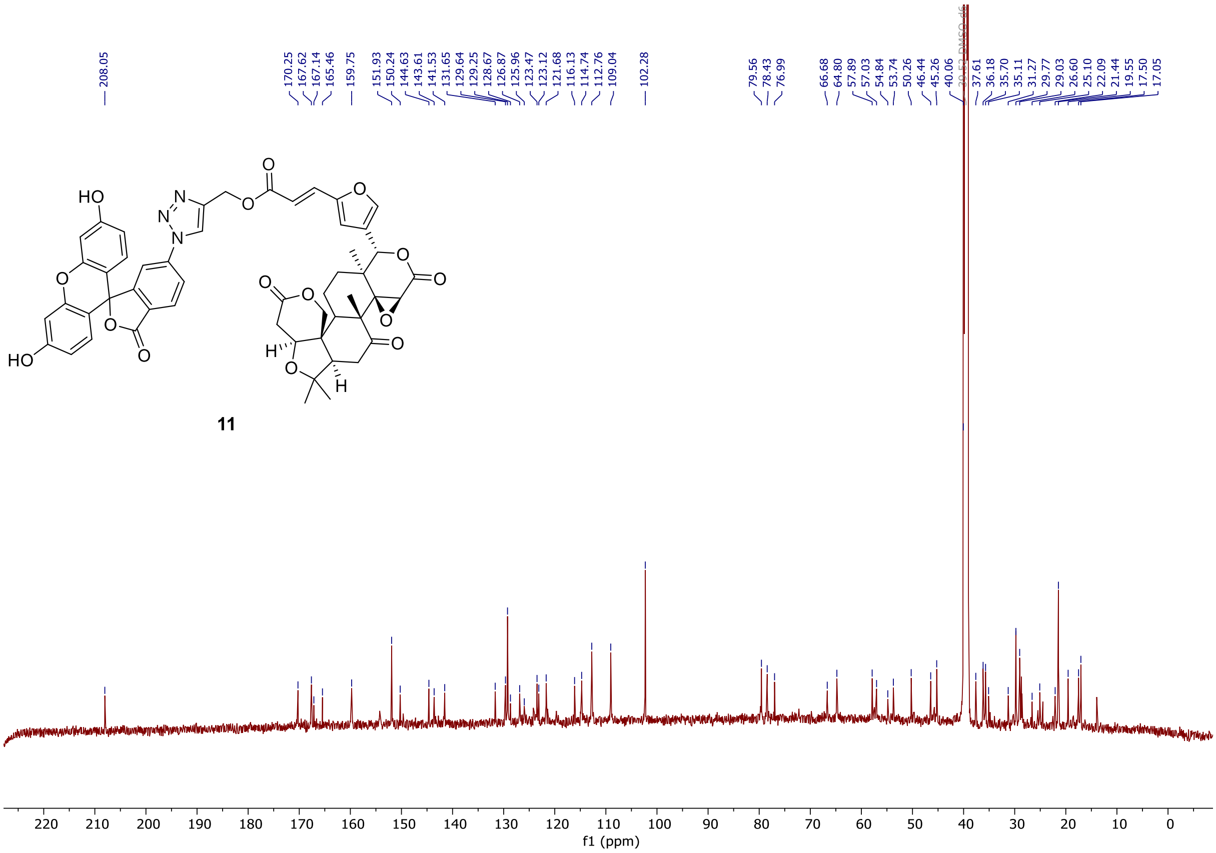
208.05
170.25 167.62 167.14 165.46 159.75 151.93 150.24 144.63 143.61 141.53 131.65 129.64 129.25 128.67 126.87 125.96 123.47 123.12 121.68 116.13 114.74 112.76 109.04 102.28
79.56 78.43 76.99
66.68 64.80 57.89 57.03 54.84 53.74 50.26 46.44 45.26 40.06 39.52 DMSO-d6 37.61 36.18 35.70 35.11 31.27 29.77 29.03 26.60 25.10 22.09 21.44 19.55 17.50 17.05
HO
N
N
N
O
O
O
O
HO
O
O
O
O
O
O
O
H
O
H
O
O
11
220 210 200 190 180 170 160 150 140 130 120 110 100 90 80 70 60 50 40 30 20 10 0
f1 (ppm)

References

(1) G. R. Fulmer, A. J. M. Miller, N. H. Sherden, H. E. Gottlieb, A. Nudelman, B. M. Stoltz, J. E. Bercaw, K. I. Goldberg. NMR Chemical Shifts of Trace Impurities: Common Laboratory Solvents, Organics, and Gases in Deuterated Solvents Relevant to the Organometallic Chemist. *Organometallics* **2010**, *29*, 2176.

(2) H. Chen, P. Wedi, T. Meyer, G. Tavakoli, M. van Gemmeren. Dual Ligand-Enabled Nondirected C-H Olefination of Arenes. *Angew. Chem. Int. Ed*. **2018**, *57*, 2497.

(3) Y.-J. Lim, G. Tang, Z. Ye, C.-J. Zhang, J. Wu, S. Q. Yao. A Late-Stage Aryl C−H Olefination Strategy and Its Application Towards Global Proteome Profiling of Δ^8 -Tetrahydrocannabinol. *Chem. Eur. J.* **2023**, *29,* e202300531.

(4) X.-Y. Chen, Y. Wu, J. Zhou, P. Wang, J.-Q. Yu. Synthesis of β-Arylethenesulfonyl Fluoride via Pd-Catalyzed Nondirected C-H Alkenylation. *Org. Lett.* **2019**, *21*, 1426.

(5) a) D. A. Strassfeld, C.-Y. Chen, H. S. Park, D. Q. Phan, J.-Q. Yu. Hydrogen-bond-acceptor ligands enable distal C(sp^3)-H arylation of free alcohols. *Nature* **2023**, *622,* 7981; b) Z. Zhuang, C.-B. Yu, G. Chen, Q.-F. Wu, Y. Hsiao, C. L. Joe, J. X. Qiao, M. A. Poss, J.-Q. Yu. Ligand-Enabled β-C(sp^3)-H Olefination of Free Carboxylic Acids. *J. Am. Chem. Soc.* **2018**, *140*, 10363; c) T. Xu, S. Mal, M. van Gemmeren. The Direct Pd-Catalyzed γ-Lactonization of Aliphatic Carboxylic Acids. *ACS Catal.* **2025**, *15*, 2735; d) F. Ghiringhelli, A. Uttry, K. K. Ghosh, M. van Gemmeren. Direct β- and γ-C(sp^3)-H Alkynylation of Free Carboxylic Acids. *Angew. Chem. Int. Ed*. **2020**, *59*, 23127; e) J. Dey, S. Kaltenberger, M. van Gemmeren. Palladium(II)-Catalyzed Nondirected Late-Stage C(sp^2)-H Deuteration of Heteroarenes Enabled Through a Multi-Substrate Screening Approach. *Angew. Chem. Int. Ed*. **2024**, *63*, e202404421.

(6) M. Farizyan, A. Mondal, S. Mal, F. Deufel, M. van Gemmeren. Palladium-Catalyzed Nondirected Late-Stage C-H Deuteration of Arenes. *J. Am. Chem. Soc.* **2021**, *143*, 16370.

(7) K. Echizen, T. Taniguchi, T. Nishimura, K. Maeda. Synthesis of Stereoregular Telechelic Poly(phenylacetylene)s: Facile Terminal Chain-End Functionalization of Poly(phenylacetylene)s by Terminative Coupling with Acrylates and Acrylamides in Rhodium-Catalyzed Living Polymerization of Phenylacetylenes. *J. Am. Chem. Soc.* **2021**, *143*, 3604.

(8) M.-R. Ryan, D. Lynch, S. G. Collins, A. R. Maguire. Selective Thermal Deprotection of N-Boc Protected Amines in Continuous Flow. *Org. Process Res. Dev.* **2024**, *28*, 1946.

(9) P. Xu, D. Zhao, F. Berger, A. Hamad, J. Rickmeier, R. Petzold, M. Kondratiuk, K. Bohdan, T. Ritter. Site-Selective Late-Stage Aromatic [^{18}F]Fluorination via Aryl Sulfonium Salts. *Angew. Chem. Int. Ed*. **2020**, *59*, 1956.

(10) K. D. Collins, A. Rühling, F. Lied, F. Glorius. Rapid assessment of protecting-group stability by using a robustness screen. *Chem. Eur. J.* **2014**, *20*, 3800.

(11) E. Wheatley, J. M. Zanghi, S. J. Meek. Diastereo-, Enantio-, and anti-Selective Formation of Secondary Alcohol and Quaternary Carbon Stereocenters by Cu-Catalyzed Additions of B-Substituted Allyl Nucleophiles to Carbonyls. *Org. Lett.* **2020**, *22*, 9269.

(12) Q. Wu, W. Liu, M. Wang, Y. Huang, P. Hu. Iron-catalyzed deconstructive alkylation through chlorine radical induced C-C single bond cleavage under visible light. *Chem. Commun.* **2022**, *58*, 9886.

(13) R. E. Nuttall, T. T. Pham, A. C. Chadwick, I. N. Hungnes, G. Firth, M. A. Heckenast, H. A. Sparkes, M. C. Galan, M. T. Ma, P. G. Pringle. Diphosphine Bioconjugates via Pt(0)-Catalyzed Hydrophosphination. A Versatile Chelator Platform for Technetium-99m and Rhenium-188 Radiolabeling of Biomolecules. *Inorg. Chem.* **2023**, *62*, 20582.

(14) W. J. Reis, P. O. L. Moreira, R. B. Alves, H. H. M. Oliveira, L. M. Silva, F. P. Varotti, R. P. Freitas. Novel Symmetrical 1,4-Disubstituted-bis-1,2,3-Triazoles: Synthesis by Double CuAAC and Cytotoxicity Evaluation. *Curr. Top. Med. Chem.* **2018**, *18*, 1475.

(15) E. H. Christen, R. J. Gübeli, B. Kaufmann, L. Merkel, R. Schoenmakers, N. Budisa, M. Fussenegger, W. Weber, B. Wiltschi. Evaluation of bicinchoninic acid as a ligand for copper(I)-catalyzed azide-alkyne bioconjugations. *Org. Biomol. Chem.* **2012**, *10*, 6629.

(16) M. Domínguez, H. Reissig. New 4-Pyridyl Nonaflates as Precursors for Push–Pull Solvatochromic Dyes. *Synthesis* **2014**, *46,* 1100.
